# Transcriptome sequencing study implicates immune-related genes differentially expressed in schizophrenia: new data and a meta-analysis

**DOI:** 10.1038/tp.2017.47

**Published:** 2017-04-18

**Authors:** A R Sanders, E I Drigalenko, J Duan, W Moy, J Freda, H H H Göring, P V Gejman

**Affiliations:** 1 Department of Psychiatry and Behavioral Sciences, NorthShore University HealthSystem, Evanston, IL, USA; 2 Department of Psychiatry and Behavioral Sciences, University of Chicago, Chicago, IL, USA; 3 Department of Genetics, Texas Biomedical Research Institute, San Antonio, TX, USA; 4 South Texas Diabetes and Obesity Institute, University of Texas Rio Grande Valley School of Medicine, San Antonio, TX, USA; 5 Molecular Genetics of Schizophrenia (MGS) Collaboration, Evanston, IL, USA

## Abstract

We undertook an RNA sequencing (RNAseq)-based transcriptomic profiling study on lymphoblastoid cell lines of a European ancestry sample of 529 schizophrenia cases and 660 controls, and found 1058 genes to be differentially expressed by affection status. These differentially expressed genes were enriched for involvement in immunity, especially the 697 genes with higher expression in cases. Comparing the current RNAseq transcriptomic profiling to our previous findings in an array-based study of 268 schizophrenia cases and 446 controls showed a highly significant positive correlation over all genes. Fifteen (18%) of the 84 genes with significant (false discovery rate<0.05) expression differences between cases and controls in the previous study and analyzed here again were differentially expressed by affection status here at a genome-wide significance level (Bonferroni *P*<0.05 adjusted for 8141 analyzed genes in total, or *P*<~6.1 × 10^−6^), all with the same direction of effect, thus providing corroborative evidence despite each sample of fully independent subjects being studied by different technological approaches. Meta-analysis of the RNAseq and array data sets (797 cases and 1106 controls) showed 169 additional genes (besides those found in the primary RNAseq-based analysis) to be differentially expressed, and provided further evidence of immune gene enrichment. In addition to strengthening our previous array-based gene expression differences in schizophrenia cases versus controls and providing transcriptomic support for some genes implicated by other approaches for schizophrenia, our study detected new genes differentially expressed in schizophrenia. We highlight RNAseq-based differential expression of various genes involved in neurodevelopment and/or neuronal function, and discuss caveats of the approach.

## Introduction

Schizophrenia, a common (~1%) and often severe psychiatric disorder, typically has a late adolescent or early adult onset of overt psychotic symptoms.^1^ A number of etiological contributions have been proposed,^2^ in particular genetic predisposition,^3^ but there is also some evidence for immunological and inflammatory mechanisms.^4, 5^ Genome-wide association studies (GWASs) of schizophrenia and their meta- and mega-analyses with increasing sample sizes have yielded over 100 genome-wide significant loci.^6, 7, 8, 9, 10^ Most common single-nucleotide polymorphism (SNP) GWAS variants lie outside of genes and/or are not in linkage disequilibrium with polymorphisms affecting amino-acid sequence,^9, 10^ and functional consequences of candidate variants remain largely unclear. Disease-associated copy-number variants (CNVs) and trait-associated SNPs from GWAS on disorders with complex genetics (including schizophrenia) have been found to be enriched for regulatory sequences (DNase I hypersensitive sites, ENCODE^11^) and for expression quantitative trait nucleotides,^12, 13, 14, 15^ which suggests a likely importance of gene regulation for such variants. Therefore, we have conducted an RNAseq-based genome-wide gene expression study to investigate genetic mechanisms involved in schizophrenia.

Our previous effort consisted of a transcriptional analysis of lymphoblastoid cell lines (LCLs) from 268 schizophrenia cases and 446 controls from the Molecular Genetics of Schizophrenia (MGS) European ancestry GWAS sample.^16^ In that first study, instead of RNAseq, we used the Illumina HT12-v4 microarray^16^ and found differential expression by affection status for 95 transcripts from 89 genes, among which we noticed immune-related gene enrichment.^16^ Interestingly, the results from a later large GWAS meta-analysis strongly supported immunological activation in schizophrenia, especially in B-lymphocyte lineages involved in acquired immunity.^10^ We present here the transcriptomic profiles of a larger, non-overlapping set of 529 schizophrenia cases and 660 controls, using RNAseq technology. Here we report our primary RNAseq gene expression findings and the results of a combined analysis of both cross-platform data sets (totaling 797 cases and 1106 controls) by the use of a sample size-weighted meta-analysis of *P*-values.

## Materials and methods

### Subjects

The Institutional Review Board from NorthShore University HealthSystem reviewed and approved the protocol, and informed consent was previously been obtained for all subjects. The MGS sample ascertainment, assessment, composition and characteristics have been previously described.^6, 16, 17^ Briefly, the MGS case–control collection is a large data set previously collected at 10 locations to study the genetic etiology of schizophrenia. As with our previous study,^16^ we included only MGS European ancestry GWAS-studied samples. The selected individuals did not overlap with the previously array-studied expression sample,^16^ that is, consisted of entirely non-overlapping subjects. We chose approximately equal proportions of males and females in each group, and roughly matched cases and controls based on 5-year age brackets, to reduce potential confounder effects. Our studied sample is described in Table 1, and the case and control components matched quite well on the measured covariates. Subject (sample) quality control for identity verification included ensuring concordance between known sex (that is, dosages of X and Y chromosomes) and RNAseq expression levels of sex-dimorphic expressed genes on chromosomes X (*XIST*) and Y (*RPS4Y1*, *ZFY*, *USP9Y*, *DDX3Y*, *UTY*, *KDM5D* and *EIF1AY*). We also compared RNAseq-called genotypes (using SAMtools mpileup function,^18^ requiring >8 reads at a called SNP site) with previously determined SNP genotypes (Affymetrix 6.0, Santa Clara, CA, USA) from the MGS GWAS^6^ for informative SNPs (mean 76 per sample), requiring at least 95% concordance (mean 99.5% concordance).

### LCLs

The standardized careful methods for obtaining, reviving, growing, assaying (growth rate and energy status) and harvesting the LCLs for RNA, as well as the use of appropriate (culture/biological and RNA/technical) replicates, RNA isolation and processing are the same as described for the previous microarray-based expression study.^16^ In the current study, all LCLs were transformed at Rutgers University Cell and DNA Repository. As with our previous array-based study, we collected LCL characteristics data that may confound the relationship between schizophrenia status and gene expression levels,^19^ namely, Epstein–Barr virus (EBV) load (copy number), cell count at harvest (LCL growth rate) and LCL energy status (indexed by ATP levels adjusted by cell count).

### Transcriptome sequencing

We shipped samples, always intercalating cases and controls (as was done in the LCL-growing protocol, to minimize any cryptic case–control differences that might still exist besides affection status), in five large batches to the University of Minnesota Genomics Center, which performed next-generation sequencing (RNAseq), using Illumina TruSeq SBS v3 (San Diego, CA, USA) reagents and Illumina HiSeq instruments, and obtaining 50 bp single-end reads, obtaining average read quality (*Q*-score >30) across the entire read length, and achieving >8 million read depth for the analyzed samples. (If more than one run was required, we combined the reads across RNAseq runs, including all runs with >2 million reads. In all such instances, the RNAseq runs were done in the same sequencing batch on the same RNA harvest, that is, we did not regrow LCLs from a study participant multiple times, harvest RNA multiple times and then run RNAseq on these multiple RNA harvests. Similarly, we did not combine RNAseq results across different RNAseq runs.)

### RNAseq data processing

We used TopHat^20^ (v2.0.12, based on Bowtie2,^21^ v2.2.3) for read alignment, and CuffLinks^22^ (v2.2.1) for counting the number of mapped alignments and calculating the measure of gene expression, FPKM (fragments per kilobase of exon per million reads mapped). We normalized the data by square root transformation as a variance-stabilizing transformation, as FPKM may be viewed as following a Poisson distribution, that is, of ‘rare events'. For human genome annotation, we used GenCode v.20 based on human genome assembly GRCh38 (hg38), and program parameters had default values. The total number of reads per sample averaged 14 040 233 (range 9 003 327–64 218 868), excepting 32 LCLs from carriers (23 cases and 9 controls) of known risk CNVs for schizophrenia. These samples were batched together and sequenced at a higher depth (mean=71 758 557, range 11 921 205–107 065 076). Excluding these 32 high-depth CNV carrier samples, there was no difference between the RNAseq coverage between cases and controls (see footnote to Table 1).

### Differential expression analyses

For the primary analysis (RNAseq, full sample), we focused on the 21 146 genes with detectable (that is, non-zero) expression in at least 80% of the samples. The average correlation of square root transformed gene expression levels across these 21 146 genes between 46 technical replicates, 63 culture replicates and all unrelated samples was in the expected order (*r*=0.99, *r*=0.98 and *r*=0.97, respectively). As in the array study before,^16^ we used the same measured covariates (sex, age, age^2^, genotypic principal components, batch (four dummy variables for five RNAseq batches), EBV load, growth rate and ATP) jointly applied (to account for their possible confounding) to a multivariate linear regression model with affection status. For the comparison to the previous array results, we limited the study to genes detected well with both array and RNAseq (detectable expression in ⩾80% of samples for both platforms). The RNAseq sample is a fully independent sample with no overlap of subjects with the array-studied sample. To combine results across the two studies (array and RNAseq), we used extension of Stouffer's *Z* method,^23^ for which we (1) selected the 8141 genes expressed in >80% of samples by both methods, (2) used the *P*-values and beta-coefficients for differential expression by schizophrenia status to generate *Z*-scores, (3) weighted the *Z*-scores by sample sizes (array 714 consisting of 268 cases and 446 controls; RNAseq 1189 consisting of 529 cases and 660 controls), (4) combined the *Z*-scores for the meta-analytic result, and then (5) reverted the combined *Z*-scores back to *P*-values corresponding to the combined *P*-values of RNAseq and array, using an R package.^24^

### Pathway and network analyses

We performed gene ontology-term and Kyoto Encyclopedia of Genes and Genomes pathway enrichment analyses using the Database for Annotation, Visualization and Integrated Discovery tool^25^ for our list 1058 differentially expressed (Bonferroni *P*<0.05) genes, using all of the analyzed genes (21 146 genes with detectable expression in at least 80% of the samples) for the background. We also submitted the same gene list to Disease Association Protein-Protein Link Evaluator^26^ to evaluate their network connectivity, using 5000 permutations to estimate significance. For the 459 input genes (of the 1058) in the same direct network identified by Disease Association Protein-Protein Link Evaluator, we also performed directed gene ontology-term enrichment analysis and Kyoto Encyclopedia of Genes and Genomes pathway analysis using the Database for Annotation, Visualization and Integrated Discovery tool.

### Data sharing

For the results of primary RNAseq expression analyses, we are sharing the data by depositing it into the database of Genotypes and Phenotypes (dbGaP, dbgap.ncbi.nlm.nih.gov). The array expression data have already been deposited into dbGaP (phs000775). GWAS and phenotypic data for all subjects have already been deposited into dbGaP (phs000021 and phs000167), and LCLs (and phenotypic data) are available through the National Institute of Mental Health repository (www.nimhgenetics.org) contractors (rucdr.rutgers.edu and zork.wustl.edu, respectively), with MGS drawing from National Institute of Mental Health repository schizophrenia studies 0 (sites 30–32), 6 (sites 40–49) and 29 (sites 139–150).

## Results

### Sample

We present an outline of the overall study design and data processing steps in Supplementary Figure S1 and present the basic characteristics of the study participants by affection status in Table 1. Cases had chronic, usually unremitting schizophrenia (88%) or schizoaffective disorder (12%) with an average age of onset of 21.6 years old. Controls were screened for psychosis, and both cases and controls were collected under the unified MGS GWAS protocol.^6^ The current RNAseq-studied sample of 529 schizophrenia cases and 660 controls were fully independent of the previous array-studied sample.^16^ Case and control study participants matched well for most primary epidemiological characteristics of study subjects and their LCLs (Table 1). RNA quality was high throughout, and RNAseq quantity and quality scores were comparable between cases and controls (Table 1).

### RNAseq preparative analyses

We aligned RNAseq data to hg38 using TopHat, with 93% of reads being aligned on average, with no significant differences observed between cases and controls (Table 1). We estimated gene-based expression levels as FPKM. We observed positive (non-zero) FPKM expression for 26 337 genes on average (median: 25 920) per sample (range: 22 660–36 325). The number of expressed genes detected increased with the number of reads per sample (Supplementary Figure S2). There is a near-linear relationship between the number of genes with detectable expression and the read count on a logarithmic scale, suggesting that non-detection of expression of genes with low average expression level was primarily due to limited read depth rather than truly absent expression. As expected, the expression levels varied widely between different genes. A histogram of the expression levels (after square root transformation, and averaged across samples) across genes is shown in Supplementary Figure S3. Not all genes were detectably expressed (non-zero FPKM) in all samples. Supplementary Figure S4 is a scatter plot of gene expression levels plotted against the proportion of samples yielding detectable expression. These two quantities are highly positively correlated (*r*=0.72), which indicates again that non-detection of a given gene in a given sample likely is caused by limited read depth rather than actual non-expression. To focus on the genes with reliably estimated expression (that is, moderately to highly expressed), and reduce the multiple testing burden, we retained for analysis genes (*N*=21 146) that had detectable expression in ⩾80% of the 1189 samples. Supplementary Figure S5 shows an overview of the numbers of genes expressed at different FPKM thresholds and the mean correlation of a given sample with all other samples, and shows a lack of any apparent sample outliers.

### Differential expression analyses

**Figure 1:**
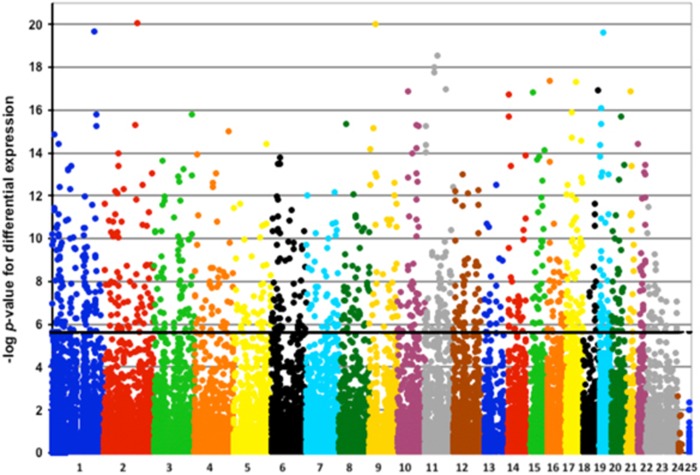
Manhattan plot of differential expression by schizophrenia status. The −log_10_ of the *P*-values for the differential expression by schizophrenia status is plotted against the chromosomal location for the 21 146 genes with detectable expression in at least 80% of the studied samples. The black bar corresponds to Bonferroni *P*⩽0.05.

We performed multiple linear regression analysis to identify genes whose average expression level varied significantly between cases and controls. Although many of the examined characteristics of cases and controls were comparable to one another (Table 1), we nonetheless included as nuisance parameters in our regression model those 16 covariates that we considered to be potential confounders or noise contributors, namely, subject characteristics (sex, age, age^2^ and genotypic principal components 1–5 to index sample ancestry), LCL characteristics (EBV load, growth rate and energy status) and RNAseq main batch (five batches, that is, four dummy variables). A total of 1058 genes (5.0%) were differentially expressed by affection status after Bonferroni *P*<0.05 adjustment for 21 146 separately analyzed genes, or *P<*~2.36 × 10^−6^ (Figure 1). Among these genes, 361 were downregulated and 697 were upregulated in cases compared to controls (Supplementary Tables S1 and S2).

### Gene set enrichment analyses

We conducted gene set enrichment analysis (GSEA) to investigate the overall characteristics of the differentially expressed genes from our RNAseq study. These 1058 differentially expressed genes were enriched (Supplementary Table S1) for genes involved in immunity (as a percentage of analyzed genes: 31% vs 13%, Fisher *P*=5 × 10^−50^). The immune enrichment was more pronounced in the upregulated (697) vs downregulated (361) genes (34% vs 25%, Fisher *P*=1.7 × 10^−3^). As most of the lines of evidence suggest immune contributions to schizophrenia point towards immune activation (see discussions, Schizophrenia Working Group of the Psychiatric Genomics Consortium,^10^ Eaton *et al.*,^27^ Eaton *et al.*,^28^ Brown and Derkits,^29^ and Brown^30^), we performed GSEA on the genes upregulated and downregulated in the schizophrenia cases separately, similar to the directional GSEA approaches used by others, (for example, Lin *et al.*^31^ and Huan *et al.*^32^). GSEA of the 697 upregulated genes using the Database for Annotation, Visualization and Integrated Discovery tool^25^ showed gene ontology-term enrichment (false discovery rate<0.05) for categories including multiple immune categories (response, activation and regulation), apoptosis regulation and cellular components (cell surface and cytosol; Table 2). Pathway analyses of the 361 downregulated genes showed gene ontology-term enrichment for different (not immune or apoptosis categories) and many more categories, including those related to transport/localization (intracellular, vesicular and membrane), multiple protein and glycosylation categories, and cellular components and molecular functions related to the aforementioned biological processes (Supplementary Table S3). In addition, the downregulated genes were enriched for the Kyoto Encyclopedia of Genes and Genomes pathway term of *N*-glycan biosynthesis (hsa00510; major constituents of glycoproteins, which often are involved in cell–cell interactions; Supplementary Table S3). We note that an increasing number of studies are finding alterations in glycan biosynthesis, glycan levels and glycosylation in schizophrenia, both in the brain and blood, suggesting dysregulation of glycosylation in schizophrenia (see review in Kippe *et al.*^33^). Recently, protein levels of important glycosylation enzymes, *B3GNT8* and *MGAT4A*, were found decreased in the prefrontal cortex in schizophrenia (12 case–control pairs),^33^ whereas in our study *B3GNT1*, *B3GNT3* and *MGAT1* transcripts were downregulated in schizophrenia cases (Supplementary Table S1). Protein–protein interaction network analyses using Disease Association Protein-Protein Link Evaluator^26^ of the 697 upregulated genes showed significantly higher network connectivity than expected, both direct (*P*=2.2 × 10^−4^) and indirect (*P*=2.2 × 10^−4^), as it did for the 361 downregulated genes (direct *P*=2.0 × 10^−4^, indirect *P*=2.0 × 10^−4^).

We have performed a number of additional analyses on all 1058 differentially expressed genes (that is, the combination of upregulated and downregulated genes), as follows. As cases were 50% male and controls were 54% male, and as males are affected with schizophrenia more often and more severely than females, we checked for enrichment of sex chromosome genes. We observed no enrichment for chromosome X (3.0% vs 2.9%, Fisher *P*=0.44) or chromosome Y (0% vs 0.09%, Fisher *P*=1.00). Similarly, as cases had a slightly higher average EBV load, we checked results for enrichment of genes known to be associated with EBV copy number,^34^ and found modestly significant statistical evidence for enrichment (1.2% vs 0.6%, *P*=0.01). Genes expressed in the adult brain were not enriched (91% vs 90%, Fisher *P*=0.14), although 84% of differentially expressed immune-related genes are expressed in the brain. More specifically, for the analyzed genes (21 146 genes with detectable expression in at least 80% of the samples), 10 774 (90%) were expressed in the brain and 1156 (10%) were not (via hbatlas.org as for Supplementary Table S1), whereas for the 1058 differentially expressed by affection status genes (Supplementary Table S1) for which there was adult brain expression data, 779 (91%) were expressed in the brain and 73 (9%) were not (via hbatlas.org, the remainder being unlisted which we designated as ‘NA' in Supplementary Table S1).

### Comparison to microarray results

We previously had conducted a gene expression study of LCLs from European ancestry cases and controls from the same MGS cohort, using non-overlapping samples.^16^ The previous microarray study^16^ and the current RNAseq study used the same laboratory methods for growing cells and harvesting RNA, and used consistent criteria for which genes to include in the analysis (expressed in ⩾80% of samples). We therefore wondered whether the findings in both samples, which were studied by different methods (microarray and RNAseq), were consistent with one another overall. Supplementary Figure S6 shows a scatter plot of the results from both studies for the 8141 genes investigated in both studies (on the scale of the sign of the estimated beta regression coefficient multiplied with the −log_10_ of the differential expression *P*-value). We found a highly significant positive correlation over all genes (*r*=0.29, *P*<10^−16^) between the array and the RNAseq findings. The correlation grows stronger among genes whose expression level is significantly associated with schizophrenia. For the *P*-value tails of the array study (array *P*<0.05, *P*<0.005, *P*<0.0005, *P*<0.00005), the correlation increases (*r*=0.47, *r*=0.55, *r*=0.57, *r*=0.68, respectively). We observe a similar pattern of increasing correlation of array and RNAseq findings when moving into such tails of the RNAseq study (RNAseq *P*<0.05: *r*=0.38, *P*<0.005: *r*=0.43, *P*<0.0005, *r*=0.47, *P*<0.00005, *r*=0.50). Thus, despite fully independent samples and different technologies (microarray and RNAseq), we found notable consistency in the detected expression levels and direction over all overlapping genes, which was more pronounced for those genes differentially expressed in schizophrenia.

Of the 89 genes that we reported to be significantly differentially expressed in our previous array study (false discovery rate<0.05),^16^ 84 were analyzed in this RNAseq study. Fifteen (18%) of those 84 were differentially expressed by affection status here at a genome-wide significance level (Bonferroni *P*<0.05 adjusted for 8141 analyzed genes in total, or *P*<~6.1 × 10^−6^), all with the same direction of effect (Supplementary Table S4). At a less stringent significance level applying a Bonferroni correction only for the 84 examined array false discovery rate<0.05 significant genes (that is, *P*<~6.0 × 10^−4^), 29 (35%) were significant in this RNAseq study, with 28 (97%) out of these 29 with the same direction of effect.

### Meta-analysis of RNAseq and array data sets

As we have confirmed differential expression by affection across two different technologies (microarray vs RNAseq) and two different (independent) sample sets, we combined the results across the two studies (array and RNAseq, for the 8141 genes expressed in >80% of samples by both methods) using Stouffer's *Z* method.^23^ Briefly, we used the differential expression *P*-values and beta-coefficients to generate *Z*-scores, which we weighted by the respective sample sizes and combined and then reverted back to differential expression *P*-values and beta-coefficients for the combined result on these 8141 jointly expressed genes. Although the primary RNAseq analysis on 21 146 genes yielded 1058 (5.0%) genes differentially expressed by affection status (Bonferroni *P*<0.05; Supplementary Tables S1 and S2), the meta-analysis of 8141 genes (expressed in >80% of samples by both methods) yielded 647 (7.9%) such genes (Bonferroni *P*<0.05; Supplementary Table S5). There was a great deal of overlap in these two genes lists, with 478 (74%) of the 647 significant genes from the meta-analysis having been in the list of 1058 significant genes from the primary RNAseq analysis. We note that similarly to the main RNAseq analysis, these 647 differentially expressed genes were enriched for genes involved in immunity (as a percentage of analyzed genes: 38% vs 22%, Fisher *P*=1.4 × 10^−18^). Among the 169 new genes from the meta-analysis (that is, not significant in the primary RNAseq analysis), are several previously discussed in our array paper^16^ and several others discussed below.

## Discussion

Using RNAseq to quantify gene expression in LCLs, we have detected 1058 genes differentially expressed by affection status in 529 schizophrenia cases and 660 controls. We have provided further support for some genes detected by other methods (for example, the Psychiatric Genomics Consortium for schizophrenia and GWAS^10^) and platforms (for example, our previous microarray expression study^16^), and we have detected novel genes (Supplementary Tables S1 and S2). These 1058 differentially expressed genes were markedly enriched for genes involved in immunity (Supplementary Table S1). GSEA showed enrichment for categories related to immunity, apoptosis, messenger RNA and protein processing and cell growth (Table 2; Supplementary Table S3). We also provide substantial corroborative evidence for our previous array-based gene profiling study^16^ (Supplementary Table S4; Supplementary Figures S6A and B).

### Genes differentially expressed in RNAseq data set

All 1058 differentially expressed genes are listed in Supplementary Table S1, and ~10% of those genes are highlighted in Supplementary Table S2. We selected to further discuss here four genes involved in immune function (*CR1*, *C3*, *TGFB1* and *TGM2*) with various additional support from the literature (expression, genetic association, human post-mortem brain, pharmacological and so on), and four genes (*PIK3CD*, *PDE4B*, *SHANK2* and *NDE1*) involved in neurodevelopment and/or neuronal function, also with various types of additional support from the literature (expression, genetic association, human post-mortem brain, rodent modeling, pharmacological and so on). Some of the genes discussed below and in Supplementary Tables S1 and S2 (and even Supplementary Table S5 below) have both neuronal and immunological roles and/or expression (for example, *C3*, *TGFB1*, *PIK3CD*, *PDE4B*, *FAM69A*, *IFITM1* and *PPP3CC*). Although we discuss the immune aspects below, these examples also serve here as reminders of various bridges between the two systems (neural and immune) relevant for schizophrenia, such as the general area of neuroinflammation,^35^ immune dysfunction and/interventions sometimes affecting psychotic symptoms (for example, co-administration of antipsychotic and anti-inflammatory drugs augmenting the former's antipsychotic effects^36, 37^), various schizophrenia treatments also having immune effects (for example, clozapine, risperidone and so on, reviewed in Muller and Schwarz^38^ and O'Sullivan *et al.*^39^), and many brain disorders also having immune or inflammatory aspects (for example, schizophrenia as below, but also depression, Alzheimer disease, multiple sclerosis and so on^40, 41, 42^).

### Immune-related differentially expressed gene examples

We found differential expression in components of the complement system, though not for *C4A*, which was recently reported to associate with schizophrenia in proportion to each allele's tendency to generate greater expression of *C4A*^43^ and also resides in the most strongly associated region of the genome (the major histocompatibility complex (MHC) region).^10^ Of the ~50 complement system genes,^44^ 23 were well expressed (in ⩾80% of samples), 4 of which were upregulated in their expression in schizophrenia cases, which was a modest enrichment of differential expression in the complement system genes (0.4% vs 0.1%, *P*=0.026). The four complement components for which we found evidence of upregulation in schizophrenia cases were: *CR1*, *CR2*, *CD55*, and *C3*. *CR1* is expressed on most blood cells and has a high affinity for complement components (C1q, C3 and C4).^45^ CR1 expression on the surface of lymphocytes, monocytes and neutrophils was found to be higher in schizophrenia cases than in controls,^46^ as its messenger RNA was in the current study. In addition, *CR1* has achieved genome-wide significance in GWAS for Alzheimer's disorder.^47, 48^ *C3* plays a central role in the activation of complement system, which is required for both classical and alternative complement activation pathways. *C3* and other complement components have been suggested to influence risk for schizophrenia (see review Mayilyan *et al.*^49^). *C3* also plays roles in neurodevelopment (synaptic remodeling).^50, 51^ *C3* has been reported as being upregulated in blood from schizophrenia cases versus controls,^52^ consistent with our findings of *C3* messenger RNA upregulation. However, a recent study found no significant difference in *C3* expression in blood for drug-naive schizophrenia cases versus controls.^53^

*TGFB1* is a cytokine that regulates proliferation, differentiation, adhesion, migration and other functions in many cell types.^54, 55^ Besides its importance in the maintenance of immune homeostasis (strongly inhibiting the production of pro-inflammatory cytokines),^54, 55^ *TGFB1* also has relevant neurodevelopmental effects (for example, trophic effects on midbrain dopaminergic neurons).^56, 57^ *TGFB1* protein has been reported to be elevated in blood from unmedicated schizophrenic cases compared to controls in Korea, with normalization after antipsychotic treatment.^58^ A small Polish case–control study showed nominal significance (*P*=0.03) for a functional SNP (T29C; Leu10Pro^59^) being associated with schizophrenia.^60^ In the Polish study, TGFB1 protein was also upregulated in schizophrenia cases versus controls,^60^ consistent with our findings of increased messenger RNA expression in schizophrenia cases.

*TGM2* catalyzes the crosslinking of proteins by epsilon-gamma glutamyl lysine isopeptide bonds, appears to be involved in apoptosis and is the autoantigen implicated in celiac disease (gluten-sensitive enteropathy).^61^ *TGM2* SNPs and a haplotype showed nominal association (*P*~0.0004) to schizophrenia in a family-based study of 131 British family trios,^62^ though no association was found in a Chinese case–control study.^63^ *TGM2* has been reported (in a patent, US20070015152) to be upregulated in the brain (anterior cingulate cortex) of schizophrenia cases versus controls. Celiac disease has an epidemiological association with schizophrenia; gluten-free diets occasionally reduce psychotic symptoms; and circulating gliadin antibodies are elevated in schizophrenia cases versus controls (reviewed in Wang *et al.*^63^).

### Neuronal-related differentially expressed gene examples

*PIK3CD* has been shown to be involved in axonal outgrowth during neurodevelopment.^64^ The catalytic subunit of phosphatidylinositol-4,5-bisphosphate 3-kinase (that is, *PIK3CD*)^65^ was previously reported to have higher expression in human LCLs (*β*=0.41),^66^ consistent with our finding (*β*=0.28). Administration of haloperidol to rats led to specific reductions of *PIK3CD* brain expression; and furthermore, specific blocking of *PIK3CD* blocked amphetamine-induced hyperlocomotion (a rodent model of psychosis-like behavior, related to a hyperdopaminergic state) in mice.^66^ *PIK3CD* has also been shown to be important for the biochemical function of the *NRG1*–*ERBB4*–*PI3K* signaling pathway, that is, involving two leading candidate genes for schizophrenia (*NRG1* and *ERBB4*).^66^ Dysregulation of PI3K activity has been implicated in both autism spectrum disorders and schizophrenia.^67, 68, 69^

*PDE4B* plays a role in signal transduction by regulating cellular concentrations of cyclic nucleotides (for example, cyclic AMP), and also has a central role in inflammation.^70, 71, 72^ Studies of *Pde4b* knockout mice demonstrated decreased striatal dopamine and serotonin activity associated with decreased prepulse inhibition, decreased baseline motor activity and an exaggerated locomotor response to amphetamine.^73^ There is also a case report of this gene being disrupted by a balanced t(1:16) translocation in a subject diagnosed with schizophrenia and a cousin with chronic psychiatric illness.^72^ Post-mortem cerebellum showed decreased *PDE4B* expression in schizophrenics versus controls,^74^ though other studies have not found differential brain expression.^75^ Increased expression in peripheral blood has been seen for depression versus controls.^76^ Several studies have reported nominal association of variants at this gene with schizophrenia,^74^ with a meta-analysis (Psychiatric Genomics Consortium for schizophrenia) reporting *P*=~2 × 10^−7^.^10^

*SHANK2* is a component of molecular scaffolds in the postsynaptic density of excitatory glutamatergic synapses, which have important roles in neurodevelopment and the adult brain.^77, 78^ A recent sequencing study of *SHANK2* in 481 schizophrenia cases and 659 controls found an over-representation of rare (minor allele frequency<0.01) missense SNPs in schizophrenia cases.^79^ In addition, variants (CNVs and SNPs) in *SHANK2* have been associated with autism and mental retardation.^78, 80, 81^ Mouse mutant lines for *SHANK2* (as well as *SHANK1* and *SHANK3*) have shown behavioral changes and dysfunction of glutamatergic synapses.^82^ *SHANK2* is upregulated in brains of Alzheimer's disorder cases (whereas *SHANK1* and *SHANK3* are downregulated).^81^

*NDE1* is a member of the nuclear distribution E family of proteins, and plays an essential role in neuronal migration.^83^ Mutations in *NDE1* cause lissencephaly 4, (lissencephaly, severe brain atrophy, microcephaly and severe intellectual deficiency).^84^ *NDE1* interacts with schizophrenia risk genes, most notably, *DISC1*.^85, 86^ *NDE1* is also within a chromosome 16p13.11 CNV reported as associated with schizophrenia (also for autism, attention deficit hyperactivity disorder, seizures and intellectual deficiency).^87, 88, 89^ A rare variant in *NDE1* has also been implicated by re-sequencing for rare coding variants (finding S214F), association testing (nominal *P*=0.04), and functional analyses (finding the mutation affected axonal outgrowth and the interaction between *NDE1* and the neurodevelopmental regulator, *YWHAE*).^90^ In a Finnish schizophrenia family cohort, interaction was reported between *NDE1* genotypes and high birth weight increasing schizophrenia susceptibility.^91^

### Support for differentially expressed genes detected by array

There were 15 genes differentially expressed by affection status in both our previous array study (false discovery rate<0.05)^16^ and the current RNAseq study (Bonferroni *P*<0.05, all with the same direction of effect, Supplementary Table S4). Notable genes among these repeated findings were: *FAM69A*, a member of the *FAM69A*-*EVI*-*RPL5* gene cluster implicated in the autoimmune disorder multiple sclerosis by GWAS^92, 93, 94^ and which also had some association support in the MGS European ancestry GWAS;^6^ *XBP1*, which is a transcription factor known to be a key regulator of MHC class II genes^95^ and has a functional promoter variant reported as nominally associated with schizophrenia in some studies on Asian samples but not in other studies;^96, 97, 98, 99, 100^ and *SYT11*, which is genome-wide significantly associated with Parkinson's disease.^101^ We note that the extended MHC region histones found differentially expressed in schizophrenia previously in our array-based study^16^ were not differentially expressed in our current RNAseq-based transcriptional profiling. This increases the likelihood that the array-based finding for histones was a technical artifact that RNAseq may be better able to avoid, for example, as the sequence similarity within the histone gene family may reduce specificity more for short microarray sequences than on a gene-wide basis as with RNAseq (see discussion in Sanders *et al.*^16^).

### Meta-analysis of RNAseq and array data sets

As is common with a large data set being studied by rapidly developing technologies, different parts of the MGS data set have been studied for gene expression by different technologies, that is, array and RNAseq. Although array expression studies were more prevalent prior to reductions in RNAseq costs, the advantages of RNAseq over array measurements are many,^102, 103^ including that RNAseq: (1) detects transcription of unknown transcripts, exons and transcript isoforms (alternatively spliced variants); (2) measures both gene-wide and exon-specific expression levels; (3) assays allele-specific expression; (4) provides sequence information; (5) provides gene expression levels that are highly correlated with measures of absolute expression level (as assayed by quantitative PCR) across a wide dynamic range; and (6) processing of the obtained read counts is less critical and complicated than with array data. Although a number of methods for meta-analysis of array data sets have been developed (reviewed in Xia *et al.*^104^), due to the technology differences, direct data merging of array vs RNAseq data sets is impractical, leaving approaches such as combination of *P*-values, rank orders or votes as meta-analytic options.^104^ We used an extension of Stouffer's *Z* method^23^ for our meta-analytic approach to combine the RNAseq and array data sets, allowing us to study the 8141 genes expressed in >80% of samples by both methods, and found 647 genes to be differentially expressed by affection status (Supplementary Table S5). Among the 169 new genes from the meta-analysis (but not significant in the primary RNAseq analysis) are several previously discussed in our array paper (*B3GNT2*, *MOXD1*, *DBNDD2* and *S100A10*),^16^ and we also chose to further highlight three additional genes here: *VAMP4*, *PPP3CC* and *IFITM3*.

*VAMP4* is a main component of a protein complex involved in the docking and/or fusion of synaptic vesicles with the presynaptic membrane,^105^ and is critical for neuron outgrowth. A Swedish family-based candidate gene association study found the cSNP rs15655 in the 3′-untranslated region of *VAMP4* to be nominally associated in both their discovery (77 trios, two-tailed *P*=0.004) and replication (190 trios, two-tailed *P*=0.019 for same allele) samples of suicide attempters.^106^

*PPP3CC* is one of the regulatory subunits for calcineurin, which is a calcium-dependent, calmodulin-stimulated protein phosphatase involved in the downstream regulation of dopaminergic signal transduction. *PPP3CC* is localized to presynaptic terminals in hippocampal neurons, and RNA interference-mediated knockdown disrupts synaptic vesicle cycling.^107^ *PPP3CC* has been reported as associated with schizophrenia in families,^108^ but a number of other studies (for example, Sanders *et al.*^109^) have not detected association. Decreased hippocampal expression has been found in 13 schizophrenia cases versus 12 controls.^110^ In two European ancestry samples, variants in *PPP3CC* were found to be nominally associated with treatment-resistant depression, antidepressant treatment response and remission (*P*-values ranging from 0.04 to 0.0002), and some of these effects were suggested to be through the B-cell receptor signaling pathway via pathway analysis (permutated *P*=0.03).^111^

Like *IFITM1* (Supplementary Table S2), *IFITM3* was also found to be upregulated in post-mortem brain (hippocampus and dorsolateral prefrontal cortex) from schizophrenic cases versus controls.^112, 113^ In a larger study (55 cases and 55 controls), prefrontal cortex showed increased *IFITM3* expression via array in schizophrenia (*P*<0.01).^114^ Of relevance for some immunological hypotheses of schizophrenia risk, mouse studies have suggested that *IFITM3* expression is a critical mediator of maternal immune activation.^115, 116^

### Immune-related genes

We continue to study LCLs due to their sample size and quality, tractability and substantial overlap with brain expression, coupled with the enhanced possibility of detecting peripheral biomarkers for schizophrenia and their relevance as a model to study immune contributions to schizophrenia, which has been noted previously.^16^ Briefly, several lines of evidence support a substantial immunological contribution to schizophrenia risk: (1) family history of autoimmune disease is associated with increased schizophrenia risk, and autoimmune disorders modify schizophrenia risk.^27, 28^ (2) Prospective birth cohort studies with serologically documented gestational infection and immune biomarkers show that specific infections increase the risk of schizophrenia in the offspring.^29, 30^ (3) GWAS have shown the strongest association at the extended MHC region,^10^ which is associated with many immune, inflammatory and infectious disorders.^117^ (4) The Psychiatric Genomics Consortium for schizophrenia recently mapped 108 genome-wide significantly associated loci onto sequences with epigenetic markers characteristic of active enhancers and found strong enrichment at enhancers active in tissues with important immune functions, particularly B-lymphocyte lineages involved in acquired immunity (CD19 and CD20 lines), which remained significant even after excluding the extended MHC region and regions containing brain enhancers.^10^ Our GSEA showed an enrichment of immune-related genes in the differentially expressed genes (as a % of analyzed genes: 31% vs 13%), and this enrichment was more pronounced in the upregulated vs downregulated genes (34% vs 25%). Our directional GSEA finding that multiple immune categories (response, activation and regulation) were enriched in the 697 upregulated genes (Table 2), but not the 361 downregulated genes (Supplementary Table S3), is consistent with most of the lines of evidence pointing towards immune activation contributing to schizophrenia (see discussions, Schizophrenia Working Group of the Psychiatric Genomics Consortium,^10^ Eaton *et al.*,^27^ Eaton *et al.*,^28^ Brown and Derkits,^29^ and Brown^30^).

### Caveats and limitations

The tissue to study is always a challenge for psychiatric disorders due to limited access to brain or neural tissue; we chose to study LCLs, that is, B-cells transformed by EBV. The EBV transformation itself may influence gene expression, although we attempted to minimize some (for example, by using LCLs only transformed at one site by a unified protocol) and to adjust for other aspects (such as including EBV load as a nuisance parameter in our regression models). Some genes are regulated differently in LCLs than in brain, and other brain expressed genes are not detectably expressed in LCLs and hence not assayed here. However, many genes expressed in the brain are expressed also in LCLs, and LCLs present other advantages including: availability of large numbers (MGS European ancestry sample, a widely shared repository sample, with GWAS genotypes available), ease of experimental manipulation, living tissue, high-quality RNA, far removed from environmental ‘state' influences (for example, diet, diurnal rhythms, exercise and medications), and arguably a particular suitability for the study of immune/infection hypotheses of schizophrenia. Other limitations of the current study include the exclusive focus on European ancestry samples (though that made it more comparable to the previous array-based study), lack of stronger correlation between array- and RNAseq-based results, analysis limited to gene expression, and focus on the full distribution (means) of expression (though please refer to our separate study focusing on extreme upper and lower tails of the expression distribution, that is, outliers^118^).

## Figures and Tables

**Table 1:** Sample characteristics

*Samples*	*Cases*	*Controls*
Sample size (*N*)	529	660
Sex (% male)	50%	54%
Age (years)	46.5 (45.5–47.5)	43.6 (42.7–44.5)
EBV load (copy number)	1.605 (1.555–1.655)	1.504 (1.465–1.543)
Clonality (% heterozygosity)	80%	78%
Cell count at harvest (growth rate)	0.475 (0.467–0.483)	0.474 (0.468–0.481)
Energy (mean ATP per cell count)	203 741 (198 756–208 726)	203 759 (199 447–208 071)
Aligned reads	15 647 543 (14 575 889–16 719 196)	13 464 505 (12 925 130–14 003 880)
Proportion reads mapped	93.00%	93.10%

Abbreviation: CNV, copy-number variants; EBV, Epstein–Barr virus; LCL, lymphoblastoid cell lines.

Note: unless otherwise indicated, values are means, with 95% confidence intervals (CIs). Cell count at harvest reflects growth rate directly, as all samples were adjusted to 250 000 cells per ml at 24 h prior to harvest. EBV load is calculated by log_10_(2^−mfdCt^). RNA quality indices (A_260_/A_280_, A_260_/A_230_, RNA integrity number, 28s/18s rRNA ratio) were all indicative of high quality, and matched well by affection. Although cases and controls are fairly well matched for these parameters, there are some minor differences: the cases were slightly older (Student's *t*-test *P*=1.8 × 10^−5^). The case LCLs had slightly higher EBV load (Student's *t*-test *P*=1.6 × 10^−3^) and percent heterozygosity (Student's *t*-test *P*=1.4 × 10^−5^). The number of aligned reads is from the Tophat statistic ‘mapped' for single-end reads, and was higher for cases than controls (Student's *t*-test *P*=1.7 × 10^−4^) due to targeted deeper sequencing of a small number (32) of carriers (23 cases and 9 controls) of known schizophrenia-associated CNVs excluding these 32 deeply sequenced CNV carriers, the mean number of aligned reads is not significantly different between cases and controls (13 154 720 vs 12 901 625).

**Table 2:** Pathway analysis findings for 697 upregulated genes differentially expressed by affection status

*Category*	*GO ID*	*GO term*	*Fold enrichment*	*FDR*
Biological process	GO:0006955	Immune response	2.38	2.00E−06
Biological process	GO:0009615	Response to virus	4.59	5.71E−04
Biological process	GO:0006916	Anti-apoptosis	3.24	2.50E−03
Biological process	GO:0050670	Regulation of lymphocyte proliferation	4.69	1.33E−02
Biological process	GO:0070663	Regulation of leukocyte proliferation	4.63	1.52E−02
Biological process	GO:0032944	Regulation of mononuclear cell proliferation	4.63	1.52E−02
Biological process	GO:0050867	Positive regulation of cell activation	4.00	1.74E−02
Biological process	GO:0042129	Regulation of T-cell proliferation	5.38	1.97E−02
Biological process	GO:0042110	T-cell activation	3.75	1.99E−02
Biological process	GO:0042981	Regulation of apoptosis	1.87	2.28E−02
Biological process	GO:0042127	Regulation of cell proliferation	1.87	2.56E−02
Biological process	GO:0002684	Positive regulation of immune system process	2.80	2.86E−02
Biological process	GO:0043067	Regulation of programmed cell death	1.85	2.97E−02
Biological process	GO:0010941	Regulation of cell death	1.84	3.34E−02
Biological process	GO:0050671	Positive regulation of lymphocyte proliferation	5.56	3.95E−02
Biological process	GO:0032946	Positive regulation of mononuclear cell proliferation	5.46	4.66E−02
Biological process	GO:0070665	Positive regulation of leukocyte proliferation	5.46	4.66E−02
Cellular component	GO:0009986	Cell surface	2.43	3.30E−02
Cellular component	GO:0005829	Cytosol	1.62	3.79E−02

Abbreviations: DAVID, Database for Annotation, Visualization and Integrated Discovery; FDR, false discovery rate; GO, gene ontology.

Note: GO terms are tabulated only for those showing FDR<0.05 for fold enrichment. The input into the DAVID tools analysis was the list of 697 genes differentially expressed by affection status (Bonferroni *P*<0.05) that were expressed at higher levels in the schizophrenia cases.
