# Supplementary material for: Transcriptome sequencing study implicates immune-related genes differentially expressed in schizophrenia: new data and a meta-analysis
Source: Transl Psychiatry. 2017 Apr 18;7(4):e1093–. doi: 10.1038/tp.2017.47 (PMC5416689; doi:10.1038/tp.2017.47)

**Supplemental Figures**

**Table of Contents**

**Figure S1. Experimental and Analytical Procedures Flowchart. . . . . . . . . . . . . . . . . . . . .** 2

**Figure S2. Relationship of genes with detectable expression to RNAseq read depth. . .** 3

**Figure S3. Distribution of mean gene expression levels among expressed genes. . . . .**  4

**Figure S4. Relationship of mean expression level and proportion of samples with detectable expression. . . . . . . . . . . . . . . . . . . . . . . . . . . . . . . . . . . . . . . . . . . . . . . . . . . . . . .** 5

**Figure S5. Sample expression overview ordered by RNAseq batch.** . . . . . . . . . . . . . . . . 6

**Figure S6. Comparison of RNAseq and array detected differentially expressed genes in two independent datasets.** . . . . . . . . . . . . . . . . . . . . . . . . . . . . . . . . . . . . . . . . . . . . . . . . . . . 7-8

**Figure S1. Experimental and Analytical Procedures Flowchart.** None

**SAMPLE**

- LCLs from MGS EA: 2 653 controls & 2 681 cases

**LABORATORY**

- Unified lab conditions
- High quality mRNA
- Intercalation of cases and controls
- Culture/biological and technical/RNA replicates

**QUALITY CONTROL**

- Strict threshold of detection
- Completion: samples and transcripts
- Correlations: technical and biological replicates

**ANALYZED SAMPLE**

- 660 controls – RUCDR (Rutgers) transformed LCLs
- 529 cases – RUCDR transformed

**GENES**

- Align reads (TopHat)
- Counted raw reads and calculated FPKM (CuffLinks)
- Normalize data by square root transformation

**DIFFERENTIAL EXPRESSION ANALYSES**

- Measured covariates (viral load, energy level, growth rate, sex, age, age^2^, first 5 genotypic PCs, and RNAseq batch) jointly applied to a multivariate linear regression model with affection status
- Primary analyses: 21 146 genes expressed in at least 80% of RNAseq samples
- Specific analyses:
  - Differential abundance analysis
  - Pathway analysis
- Replication analyses: 8 141 genes also detectably expressed in at least 80% of array samples

**Figure S2. Relationship of genes with detectable expression to RNAseq read depth.** The number of expressed genes (x-axis, thousands) detected increases in a near linear manner with the number of mapped reads per sample (y-axis, millions plotted on logarithmic scale), suggesting that non-detection of expression of genes with low average expression level was primarily due to limited read depth rather than truly absent expression.

**Figure S3. Distribution of mean gene expression levels among expressed genes.** The histogram of the number of expressed genes (in thousands) are plotted for several mean expression level bins (in square root of FPKM).

**Figure S4. Relationship of mean expression level and proportion of samples with detectable expression.**  This is a scatter plot of the average gene expression levels (left y-axis, on a scale of log_10_(mean squareroot(FPKM) + 1)) of all genes plotted against the proportion of samples yielding detectable expression (x-axis). On the right y-axis, the mean of each bin is plotted.


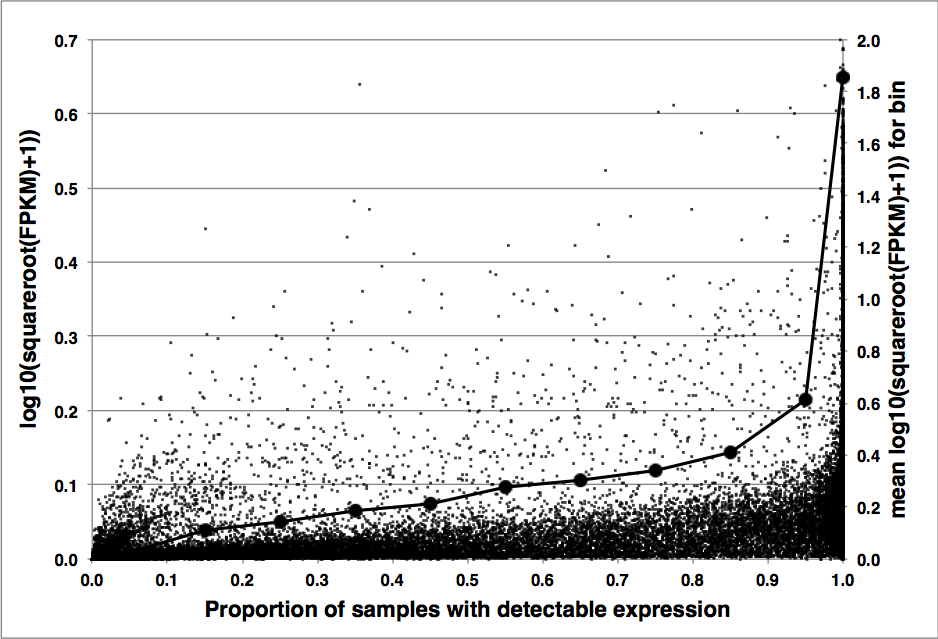


**Figure S5. Sample expression overview ordered by RNAseq batch.** The figure provides an overview of key characteristics of all analyzed 1 189 RNAseq samples, namely the numbers of genes expressed at different FPKM thresholds, and the mean correlation of a given sample with all other samples (across transformed gene expression levels for all analyzed genes). Samples are sorted by RNAseq batch (the 5 batches are demarcated by grey vertical lines) and, within batch, by affection status (controls to left and schizophrenia cases to right of each red vertical line). Note that within batch 2 (13 controls and 32 cases), CNV carriers (9 controls and 23 cases) were sequenced at much higher depth (71 758 557 total reads per sample on average), since these subjects contain CNVs thought to play a role in schizophrenia risk. This is apparent by the larger number of genes with detectable expression (top red horizontal line) for most of batch 2, but there are no apparent differences otherwise between these samples’ RNASeq data and all others.


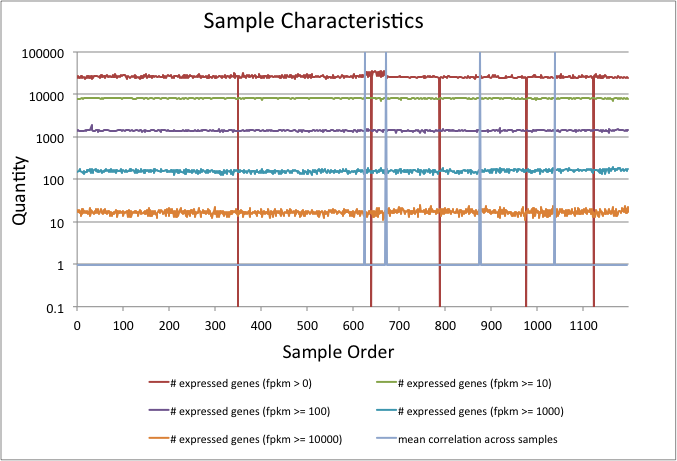


**Figure S6. Comparison of RNAseq and array detected differentially expressed genes in two independent datasets.** The -log_10_ of the *P*-values for the differential expression by schizophrenia status with the sign of the Beta coefficient are plotted for the RNAseq vs. the array experiments. The concentration of points in the top-right and bottom-left corners (i.e., the consistent direction of effect) is more readily seen for the more significant genes (i.e., in Figure S5B). (A) This figure includes all 8 141 genes expressed in >80% of samples by both methods (array & RNAseq). (B) This figure only displays the 616 (of 8 141) genes that had a nominal *P*<0.05 for differential expression by both array and RNAseq.

**A panel:**


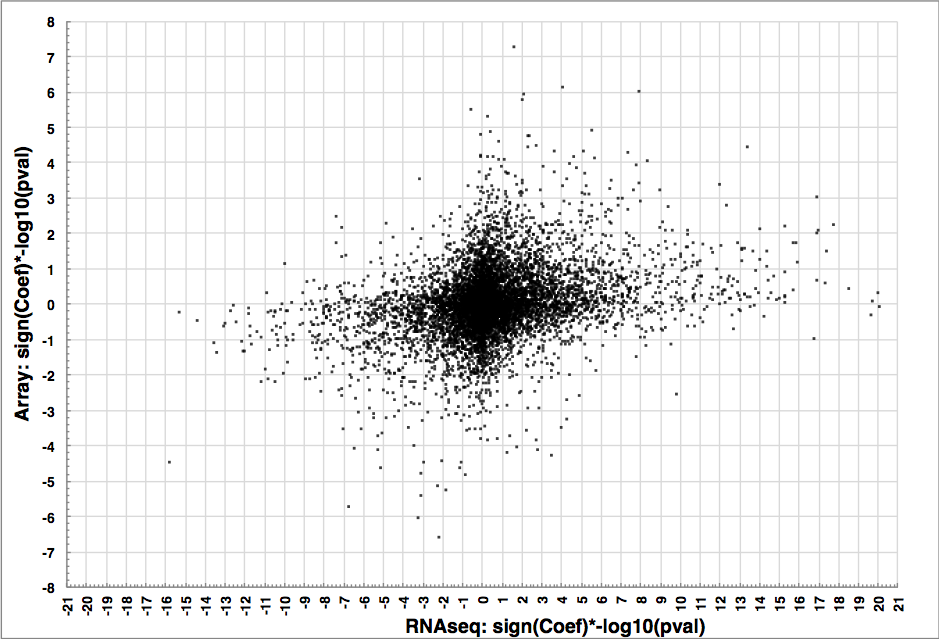


**B panel:**


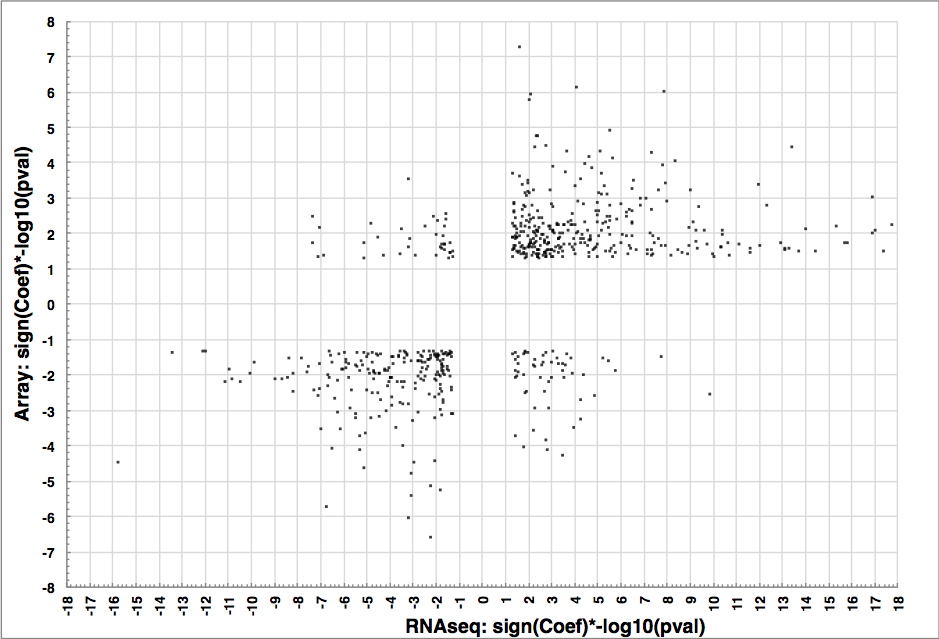

Supplement: Supplementary Information [file tp201747x1.docx]
