# Supplementary material for: Transcriptome sequencing study implicates immune-related genes differentially expressed in schizophrenia: new data and a meta-analysis
Source: Transl Psychiatry. 2017 Apr 18;7(4):e1093–. doi: 10.1038/tp.2017.47 (PMC5416689; doi:10.1038/tp.2017.47)
Supplement: Supplementary Table 1 [file tp201747x2.docx]

| **Table S1. Transcripts Differentially Expressed (Bonferroni *P*≤0.05) by Affection Status** | | | | | | | | |
| --- | --- | --- | --- | --- | --- | --- | --- | --- |
| **ensGene** | **Gene Abbreviation** | **Location (hg38)** | **Brain Expression** | **Immune Function** | ***P*-value** | **Beta coefficient** | **Bonferroni** | **Fold Change** |
| ENSG00000187608.5 | *ISG15* | chr1:1013422-1014540 | yes | yes | 1.96E-10 | 1.154 | 4.13E-06 | 0.078 |
| ENSG00000217801.6 | *RP11-465B22.3* | chr1:1059733-1069355 | NA | no | 6.20E-11 | 0.201 | 1.31E-06 | 0.082 |
| ENSG00000273443.1 | *RP11-54O7.18* | chr1:1062207-1063288 | NA | no | 1.11E-07 | 0.086 | 2.34E-03 | 0.091 |
| ENSG00000189339.8 | *SLC35E2B* | chr1:1659528-1692728 | NA | no | 2.18E-06 | -0.092 | 4.61E-02 | -0.032 |
| ENSG00000198912.7 | *C1orf174* | chr1:3889124-3900293 | yes | no | 1.25E-10 | 0.082 | 2.63E-06 | 0.021 |
| ENSG00000049249.5 | *TNFRSF9* | chr1:7915893-7943165 | no | yes | 3.70E-12 | 0.144 | 7.83E-08 | 0.170 |
| ENSG00000171608.12 | *PIK3CD* | chr1:9651731-9729114 | yes | yes | 1.31E-15 | 0.284 | 2.76E-11 | 0.042 |
| ENSG00000116663.7 | *FBXO6* | chr1:11664123-11674354 | yes | yes | 4.77E-12 | 0.219 | 1.01E-07 | 0.052 |
| ENSG00000142634.9 | *EFHD2* | chr1:15409894-15430343 | yes | yes | 2.30E-06 | 0.178 | 4.87E-02 | 0.019 |
| ENSG00000187144.8 | *SPATA21* | chr1:16387116-16437424 | yes | no | 8.60E-09 | 0.054 | 1.82E-04 | 0.009 |
| ENSG00000077549.14 | *CAPZB* | chr1:19338775-19485539 | yes | no | 8.76E-07 | 0.260 | 1.85E-02 | 0.014 |
| ENSG00000158828.5 | *PINK1* | chr1:20633454-20651511 | yes | yes | 3.05E-11 | -0.059 | 6.44E-07 | -0.062 |
| ENSG00000117242.7 | *PINK1-AS* | chr1:20642656-20652193 | NA | no | 3.49E-10 | -0.059 | 7.38E-06 | -0.030 |
| ENSG00000244038.6 | *DDOST* | chr1:20651766-20661544 | yes | yes | 2.69E-11 | -0.457 | 5.68E-07 | -0.028 |
| ENSG00000231105.1 | *RP5-1071N3.1* | chr1:21293289-21299774 | NA | no | 7.12E-07 | -0.045 | 1.51E-02 | -0.069 |
| ENSG00000169504.11 | *CLIC4* | chr1:24745356-24844324 | yes | no | 5.94E-07 | -0.175 | 1.26E-02 | -0.030 |
| ENSG00000020633.15 | *RUNX3* | chr1:24899510-24965121 | yes | yes | 2.26E-11 | 0.435 | 4.77E-07 | 0.046 |
| ENSG00000157978.8 | *LDLRAP1* | chr1:25543579-25568886 | yes | no | 5.26E-08 | -0.341 | 1.11E-03 | -0.063 |
| ENSG00000117676.10 | *RPS6KA1* | chr1:26529760-26575030 | yes | yes | 4.07E-08 | 0.208 | 8.60E-04 | 0.026 |
| ENSG00000126709.11 | *IFI6* | chr1:27666060-27672218 | yes | yes | 9.70E-10 | 0.739 | 2.05E-05 | 0.084 |
| ENSG00000253304.1 | *TMEM200B* | chr1:29119427-29123935 | yes | no | 2.34E-06 | -0.069 | 4.95E-02 | -0.058 |
| ENSG00000162517.9 | *PEF1* | chr1:31629861-31644896 | yes | no | 5.38E-09 | 0.121 | 1.14E-04 | 0.019 |
| ENSG00000160050.11 | *CCDC28B* | chr1:32200385-32205387 | yes | no | 8.03E-11 | 0.418 | 1.70E-06 | 0.050 |
| ENSG00000224066.1 | *RP4-622L5.7* | chr1:32204768-32206814 | NA | no | 6.37E-11 | 0.183 | 1.35E-06 | 0.056 |
| ENSG00000175130.6 | *MARCKSL1* | chr1:32333831-32336379 | yes | no | 3.76E-15 | 0.901 | 7.95E-11 | 0.085 |
| ENSG00000004455.13 | *AK2* | chr1:33007983-33080996 | yes | no | 6.90E-12 | 0.217 | 1.46E-07 | 0.020 |
| ENSG00000243749.1 | *ZMYM6NB* | chr1:34981534-34985353 | NA | no | 2.89E-10 | 0.166 | 6.12E-06 | 0.049 |
| ENSG00000126067.8 | *PSMB2* | chr1:35599543-35641844 | yes | yes | 4.03E-13 | 0.177 | 8.52E-09 | 0.021 |
| ENSG00000163874.8 | *ZC3H12A* | chr1:37474551-37484379 | no | yes | 6.59E-10 | 0.149 | 1.39E-05 | 0.050 |
| ENSG00000196449.3 | *YRDC* | chr1:37802943-37808185 | NA | no | 8.50E-10 | 0.089 | 1.80E-05 | 0.022 |
| ENSG00000185668.6 | *POU3F1* | chr1:38043826-38046794 | yes | no | 2.88E-09 | 0.131 | 6.10E-05 | 0.200 |
| ENSG00000214114.5 | *MYCBP* | chr1:38862963-38874495 | NA | no | 3.41E-07 | -0.192 | 7.21E-03 | -0.020 |
| ENSG00000090621.10 | *PABPC4* | chr1:39560815-39576790 | yes | yes | 7.89E-08 | -0.586 | 1.67E-03 | -0.030 |
| ENSG00000228060.1 | *RP11-69E11.8* | chr1:39565159-39573203 | NA | no | 6.44E-09 | -0.204 | 1.36E-04 | -0.032 |
| ENSG00000116985.7 | *BMP8B* | chr1:39757181-39788861 | yes | no | 2.27E-10 | -0.229 | 4.81E-06 | -0.162 |
| ENSG00000117016.6 | *RIMS3* | chr1:40620678-40665657 | yes | no | 8.50E-09 | 0.109 | 1.80E-04 | 0.132 |
| ENSG00000171960.7 | *PPIH* | chr1:42658424-42676758 | yes | no | 2.05E-08 | 0.186 | 4.34E-04 | 0.022 |
| ENSG00000229431.1 | *RP1-92O14.6* | chr1:43385112-43389155 | NA | no | 7.20E-07 | -0.070 | 1.52E-02 | -0.026 |
| ENSG00000117419.11 | *ERI3* | chr1:44221069-44355260 | yes | no | 1.50E-10 | 0.135 | 3.17E-06 | 0.025 |
| ENSG00000236624.5 | *CCDC163P* | chr1:45493865-45500079 | NA | NA | 1.02E-08 | -0.107 | 2.15E-04 | -0.053 |
| ENSG00000159596.6 | *TMEM69* | chr1:45687213-45694443 | yes | no | 9.91E-07 | 0.132 | 2.09E-02 | 0.021 |
| ENSG00000132128.13 | *LRRC41* | chr1:46261195-46303608 | yes | yes | 1.51E-07 | -0.141 | 3.20E-03 | -0.021 |
| ENSG00000162385.7 | *MAGOH* | chr1:53226891-53238610 | yes | no | 1.50E-08 | 0.233 | 3.16E-04 | 0.022 |
| ENSG00000116209.8 | *TMEM59* | chr1:54031662-54053504 | yes | no | 1.30E-06 | -0.256 | 2.74E-02 | -0.023 |
| ENSG00000162402.9 | *USP24* | chr1:55066358-55215113 | yes | no | 1.19E-06 | -0.145 | 2.51E-02 | -0.036 |
| ENSG00000134716.6 | *CYP2J2* | chr1:59893307-59926790 | yes | no | 7.57E-07 | 0.074 | 1.60E-02 | 0.155 |
| ENSG00000184588.14 | *PDE4B* | chr1:65792513-66374579 | yes | yes | 1.51E-11 | 0.250 | 3.18E-07 | 0.087 |
| ENSG00000116791.10 | *CRYZ* | chr1:74705481-74733408 | yes | no | 1.35E-11 | 0.510 | 2.85E-07 | 0.076 |
| ENSG00000137959.12 | *IFI44L* | chr1:78619921-78646145 | yes | yes | 6.04E-14 | 1.119 | 1.28E-09 | 0.132 |
| ENSG00000137965.7 | *IFI44* | chr1:78649795-78664078 | yes | no | 5.85E-14 | 0.833 | 1.24E-09 | 0.103 |
| ENSG00000174021.7 | *GNG5* | chr1:84498324-84506565 | yes | yes | 9.83E-08 | 0.172 | 2.08E-03 | 0.015 |
| ENSG00000153898.9 | *MCOLN2* | chr1:84925582-84997113 | no | no | 1.03E-10 | 0.364 | 2.19E-06 | 0.048 |
| ENSG00000117174.7 | *ZNHIT6* | chr1:85649422-85708433 | yes | yes | 2.97E-07 | 0.075 | 6.28E-03 | 0.019 |
| ENSG00000171502.11 | *COL24A1* | chr1:85729232-86156943 | yes | no | 1.66E-06 | -0.121 | 3.51E-02 | -0.060 |
| ENSG00000117228.9 | *GBP1* | chr1:89052318-89065360 | yes | yes | 4.74E-13 | 0.417 | 1.00E-08 | 0.107 |
| ENSG00000162645.9 | *GBP2* | chr1:89106131-89150456 | NA | yes | 1.31E-08 | 0.362 | 2.78E-04 | 0.084 |
| ENSG00000162654.8 | *GBP4* | chr1:89181147-89198932 | yes | yes | 4.13E-14 | 0.654 | 8.73E-10 | 0.264 |
| ENSG00000069702.7 | *TGFBR3* | chr1:91680342-91906335 | yes | yes | 1.80E-06 | -0.161 | 3.81E-02 | -0.109 |
| ENSG00000154511.8 | *FAM69A* | chr1:92832736-92961522 | yes | no | 1.78E-07 | -0.170 | 3.76E-03 | -0.051 |
| ENSG00000067334.10 | *DNTTIP2* | chr1:93866282-93879918 | yes | no | 5.79E-08 | 0.141 | 1.23E-03 | 0.020 |
| ENSG00000079335.14 | *CDC14A* | chr1:100351733-100520277 | yes | no | 8.32E-11 | 0.074 | 1.76E-06 | 0.052 |
| ENSG00000228086.1 | *RP5-837M10.4* | chr1:100462398-100485997 | NA | no | 1.72E-06 | 0.080 | 3.64E-02 | 0.088 |
| ENSG00000198890.7 | *PRMT6* | chr1:107056678-107059294 | yes | yes | 2.74E-10 | 0.093 | 5.79E-06 | 0.029 |
| ENSG00000065135.8 | *GNAI3* | chr1:109548610-109618321 | yes | yes | 2.89E-10 | 0.037 | 6.11E-06 | 0.017 |
| ENSG00000273221.1 | *RP5-1180E21.5* | chr1:111184414-111185061 | NA | no | 5.01E-07 | 0.182 | 1.06E-02 | 0.032 |
| ENSG00000155363.15 | *MOV10* | chr1:112673140-112700746 | yes | yes | 2.28E-09 | 0.283 | 4.82E-05 | 0.044 |
| ENSG00000203865.6 | *ATP1A1-AS1* | chr1:116392246-116418622 | NA | no | 4.05E-08 | 0.158 | 8.56E-04 | 0.023 |
| ENSG00000183508.4 | *FAM46C* | chr1:117605933-117628372 | yes | no | 5.28E-07 | -0.272 | 1.12E-02 | -0.095 |
| ENSG00000265241.3 | *RBM8A* | chr1:145921555-145927495 | yes | no | 6.34E-10 | 0.200 | 1.34E-05 | 0.020 |
| ENSG00000169418.9 | *NPR1* | chr1:153678636-153693992 | yes | yes | 6.82E-11 | 0.127 | 1.44E-06 | 0.177 |
| ENSG00000143515.13 | *ATP8B2* | chr1:154325552-154351307 | yes | no | 3.31E-11 | -0.298 | 6.99E-07 | -0.078 |
| ENSG00000160712.9 | *IL6R* | chr1:154405192-154469450 | yes | yes | 1.89E-09 | -0.318 | 4.00E-05 | -0.110 |
| ENSG00000160714.6 | *UBE2Q1* | chr1:154548576-154559028 | yes | yes | 3.14E-07 | -0.236 | 6.65E-03 | -0.016 |
| ENSG00000160710.12 | *ADAR* | chr1:154582061-154627999 | yes | yes | 6.56E-09 | 0.298 | 1.39E-04 | 0.025 |
| ENSG00000179085.7 | *DPM3* | chr1:155139890-155140595 | yes | no | 4.64E-07 | -0.150 | 9.82E-03 | -0.030 |
| ENSG00000177628.12 | *GBA* | chr1:155234451-155244699 | yes | yes | 6.24E-10 | -0.146 | 1.32E-05 | -0.028 |
| ENSG00000132718.8 | *SYT11* | chr1:155859508-155885199 | yes | no | 1.64E-08 | 0.157 | 3.46E-04 | 0.062 |
| ENSG00000163479.10 | *SSR2* | chr1:156009047-156020959 | yes | yes | 3.24E-11 | -0.479 | 6.85E-07 | -0.030 |
| ENSG00000160803.7 | *UBQLN4* | chr1:156035300-156053794 | NA | no | 5.20E-10 | 0.081 | 1.10E-05 | 0.020 |
| ENSG00000272068.1 | *RP11-284F21.9* | chr1:156637782-156641004 | NA | no | 1.04E-09 | -0.063 | 2.21E-05 | -0.124 |
| ENSG00000132692.15 | *BCAN* | chr1:156641389-156659532 | yes | no | 5.23E-07 | -0.078 | 1.11E-02 | -0.101 |
| ENSG00000272405.1 | *RP11-284F21.10* | chr1:156641665-156644887 | NA | no | 1.07E-08 | -0.155 | 2.25E-04 | -0.097 |
| ENSG00000229953.1 | *RP11-284F21.7* | chr1:156646506-156661424 | NA | no | 9.40E-10 | -0.206 | 1.99E-05 | -0.105 |
| ENSG00000158473.6 | *CD1D* | chr1:158179946-158184896 | yes | yes | 2.60E-07 | 0.106 | 5.49E-03 | 0.135 |
| ENSG00000163565.15 | *IFI16* | chr1:158999967-159055155 | yes | yes | 2.75E-10 | 0.373 | 5.82E-06 | 0.030 |
| ENSG00000158710.11 | *TAGLN2* | chr1:159918106-159925732 | yes | no | 1.36E-07 | 0.353 | 2.87E-03 | 0.021 |
| ENSG00000162734.9 | *PEA15* | chr1:160205336-160215376 | yes | no | 1.08E-12 | 0.388 | 2.29E-08 | 0.052 |
| ENSG00000122218.11 | *COPA* | chr1:160289272-160343400 | yes | no | 6.53E-07 | -0.141 | 1.38E-02 | -0.015 |
| ENSG00000117090.11 | *SLAMF1* | chr1:160608099-160647295 | no | yes | 7.36E-10 | 0.468 | 1.56E-05 | 0.060 |
| ENSG00000122223.9 | *CD244* | chr1:160830159-160862855 | no | yes | 9.86E-07 | 0.171 | 2.09E-02 | 0.171 |
| ENSG00000143256.4 | *PFDN2* | chr1:161100555-161118111 | yes | no | 1.34E-07 | 0.175 | 2.84E-03 | 0.019 |
| ENSG00000158850.11 | *B4GALT3* | chr1:161171309-161177968 | yes | no | 3.50E-09 | -0.305 | 7.39E-05 | -0.037 |
| ENSG00000143179.9 | *UCK2* | chr1:165827530-165911618 | yes | no | 9.26E-08 | 0.161 | 1.96E-03 | 0.025 |
| ENSG00000188859.6 | *FAM78B* | chr1:166057425-166166969 | NA | no | 2.24E-08 | 0.105 | 4.74E-04 | 0.120 |
| ENSG00000143153.9 | *ATP1B1* | chr1:169105696-169132722 | yes | no | 1.95E-07 | 0.480 | 4.12E-03 | 0.100 |
| ENSG00000143156.10 | *NME7* | chr1:169132530-169367967 | yes | no | 1.75E-08 | 0.304 | 3.69E-04 | 0.060 |
| ENSG00000188404.5 | *SELL* | chr1:169690666-169711698 | yes | yes | 2.43E-10 | 0.872 | 5.13E-06 | 0.111 |
| ENSG00000075945.9 | *KIFAP3* | chr1:169921325-170085208 | yes | yes | 3.95E-07 | 0.154 | 8.36E-03 | 0.039 |
| ENSG00000057252.9 | *SOAT1* | chr1:179293713-179358680 | yes | yes | 1.92E-07 | 0.194 | 4.05E-03 | 0.040 |
| ENSG00000230124.3 | *LHX4-AS1* | chr1:180269652-180502954 | NA | no | 3.11E-08 | 0.118 | 6.58E-04 | 0.019 |
| ENSG00000162783.9 | *IER5* | chr1:181088711-181090838 | yes | no | 1.77E-06 | 0.144 | 3.75E-02 | 0.019 |
| ENSG00000143333.6 | *RGS16* | chr1:182598622-182604408 | yes | no | 1.47E-07 | 0.226 | 3.10E-03 | 0.049 |
| ENSG00000135829.13 | *DHX9* | chr1:182839368-182887751 | yes | yes | 2.25E-07 | 0.161 | 4.75E-03 | 0.014 |
| ENSG00000116406.15 | *EDEM3* | chr1:184690230-184754913 | yes | no | 1.88E-06 | -0.166 | 3.97E-02 | -0.042 |
| ENSG00000090104.8 | *RGS1* | chr1:192575726-192580031 | yes | yes | 2.54E-12 | 1.185 | 5.37E-08 | 0.145 |
| ENSG00000133059.13 | *DSTYK* | chr1:205142504-205211566 | yes | no | 1.21E-07 | -0.057 | 2.56E-03 | -0.072 |
| ENSG00000163545.7 | *NUAK2* | chr1:205302058-205321755 | yes | no | 1.38E-07 | -0.079 | 2.92E-03 | -0.140 |
| ENSG00000158715.5 | *SLC45A3* | chr1:205657850-205680459 | yes | no | 2.04E-20 | 0.200 | 4.30E-16 | 0.151 |
| ENSG00000266028.4 | *SRGAP2* | chr1:206203344-206464443 | NA | no | 1.17E-11 | -0.098 | 2.48E-07 | -0.037 |
| ENSG00000263528.4 | *IKBKE* | chr1:206470475-206496889 | yes | yes | 8.95E-08 | 0.120 | 1.89E-03 | 0.032 |
| ENSG00000162888.4 | *C1orf147* | chr1:206491115-206497728 | NA | no | 2.00E-06 | 0.034 | 4.24E-02 | 0.026 |
| ENSG00000196352.10 | *CD55* | chr1:207321507-207360966 | yes | yes | 2.98E-07 | 0.176 | 6.31E-03 | 0.055 |
| ENSG00000117322.13 | *CR2* | chr1:207454229-207489895 | no | yes | 5.70E-10 | 0.449 | 1.21E-05 | 0.130 |
| ENSG00000203710.7 | *CR1* | chr1:207496146-207640647 | no | yes | 7.83E-10 | 0.151 | 1.66E-05 | 0.137 |
| ENSG00000236911.3 | *RP11-78B10.2* | chr1:207551924-207606555 | NA | no | 1.24E-07 | 0.027 | 2.62E-03 | 0.154 |
| ENSG00000009790.11 | *TRAF3IP3* | chr1:209756031-209782320 | no | no | 3.06E-08 | -0.344 | 6.46E-04 | -0.036 |
| ENSG00000162757.4 | *C1orf74* | chr1:209779207-209784559 | yes | no | 6.79E-08 | -0.056 | 1.44E-03 | -0.024 |
| ENSG00000123684.9 | *LPGAT1* | chr1:211743456-211830772 | yes | yes | 2.16E-07 | 0.128 | 4.56E-03 | 0.035 |
| ENSG00000162772.13 | *ATF3* | chr1:212565333-212620777 | yes | yes | 1.55E-16 | 0.351 | 3.28E-12 | 0.118 |
| ENSG00000123685.5 | *BATF3* | chr1:212686417-212699985 | yes | yes | 5.48E-16 | 0.352 | 1.16E-11 | 0.094 |
| ENSG00000143494.12 | *VASH2* | chr1:212950519-212992037 | yes | no | 2.12E-06 | -0.200 | 4.49E-02 | -0.058 |
| ENSG00000136643.8 | *RPS6KC1* | chr1:213051232-213274773 | yes | no | 3.26E-09 | 0.065 | 6.90E-05 | 0.029 |
| ENSG00000135763.6 | *URB2* | chr1:229626233-229660199 | yes | no | 1.39E-08 | 0.064 | 2.94E-04 | 0.022 |
| ENSG00000183780.9 | *SLC35F3* | chr1:233904932-234324516 | yes | no | 2.79E-08 | 0.115 | 5.89E-04 | 0.182 |
| ENSG00000116962.11 | *NID1* | chr1:235975829-236065162 | yes | no | 3.56E-07 | 0.224 | 7.53E-03 | 0.091 |
| ENSG00000134326.8 | *CMPK2* | chr2:6840569-6866635 | yes | no | 2.43E-12 | 0.334 | 5.14E-08 | 0.130 |
| ENSG00000134321.8 | *RSAD2* | chr2:6865805-6898239 | yes | yes | 2.77E-09 | 0.171 | 5.86E-05 | 0.112 |
| ENSG00000143870.9 | *PDIA6* | chr2:10783390-10837977 | yes | no | 1.41E-08 | -0.406 | 2.97E-04 | -0.025 |
| ENSG00000119777.15 | *TMEM214* | chr2:27032909-27041695 | yes | no | 1.40E-11 | -0.235 | 2.97E-07 | -0.030 |
| ENSG00000138074.11 | *SLC5A6* | chr2:27199586-27212958 | yes | no | 1.48E-08 | 0.177 | 3.13E-04 | 0.030 |
| ENSG00000119801.9 | *YPEL5* | chr2:30146940-30160533 | yes | no | 1.14E-06 | -0.163 | 2.42E-02 | -0.032 |
| ENSG00000162959.10 | *MEMO1* | chr2:31865059-32011230 | yes | no | 3.33E-08 | 0.111 | 7.05E-04 | 0.017 |
| ENSG00000008869.8 | *HEATR5B* | chr2:36968382-37084342 | NA | no | 1.06E-08 | -0.104 | 2.25E-04 | -0.033 |
| ENSG00000152133.11 | *GPATCH11* | chr2:37084450-37099244 | NA | no | 7.11E-08 | -0.151 | 1.50E-03 | -0.034 |
| ENSG00000055332.13 | *EIF2AK2* | chr2:37099209-37157065 | yes | yes | 4.73E-11 | 0.263 | 1.00E-06 | 0.041 |
| ENSG00000224891.1 | *AC007899.3* | chr2:37148529-37149304 | NA | no | 3.66E-07 | 0.090 | 7.74E-03 | 0.043 |
| ENSG00000119729.7 | *RHOQ* | chr2:46541805-46583121 | NA | no | 8.11E-11 | -0.375 | 1.72E-06 | -0.059 |
| ENSG00000250116.2 | *RP11-417F21.1* | chr2:46568255-46580238 | NA | no | 1.64E-11 | -0.080 | 3.47E-07 | -0.071 |
| ENSG00000151665.9 | *PIGF* | chr2:46580936-46617119 | no | no | 6.60E-11 | -0.209 | 1.39E-06 | -0.040 |
| ENSG00000068912.10 | *ERLEC1* | chr2:53787043-53818819 | yes | no | 1.07E-08 | -0.222 | 2.25E-04 | -0.037 |
| ENSG00000271615.1 | *CTD-2026C7.1* | chr2:57755427-57766052 | NA | no | 9.80E-08 | 0.197 | 2.07E-03 | 0.270 |
| ENSG00000028116.13 | *VRK2* | chr2:57907650-58159920 | yes | no | 5.76E-13 | 0.303 | 1.22E-08 | 0.050 |
| ENSG00000115392.8 | *FANCL* | chr2:58159242-58241372 | yes | no | 7.47E-12 | 0.185 | 1.58E-07 | 0.031 |
| ENSG00000225889.4 | *AC074289.1* | chr2:64143238-64252859 | NA | no | 3.28E-07 | -0.215 | 6.93E-03 | -0.054 |
| ENSG00000230923.1 | *LINC00309* | chr2:64185077-64205485 | NA | no | 4.73E-11 | -0.025 | 1.00E-06 | -0.086 |
| ENSG00000260101.1 | *RP11-568N6.1* | chr2:64522186-64524093 | NA | no | 3.71E-09 | -0.166 | 7.85E-05 | -0.051 |
| ENSG00000115956.9 | *PLEK* | chr2:68365172-68397453 | yes | yes | 7.41E-13 | 0.540 | 1.57E-08 | 0.041 |
| ENSG00000163219.8 | *ARHGAP25* | chr2:68679600-68826833 | no | no | 1.63E-06 | -0.229 | 3.45E-02 | -0.101 |
| ENSG00000196975.11 | *ANXA4* | chr2:69644424-69826464 | yes | yes | 1.22E-06 | 0.352 | 2.58E-02 | 0.038 |
| ENSG00000169564.6 | *PCBP1* | chr2:70087453-70089203 | yes | no | 1.33E-07 | 0.187 | 2.82E-03 | 0.011 |
| ENSG00000124370.7 | *MCEE* | chr2:71109683-71130239 | yes | no | 5.20E-08 | -0.123 | 1.10E-03 | -0.029 |
| ENSG00000116096.5 | *SPR* | chr2:72887359-72892158 | yes | no | 1.61E-08 | 0.141 | 3.40E-04 | 0.083 |
| ENSG00000135638.10 | *EMX1* | chr2:72916259-72936071 | yes | yes | 1.05E-14 | 0.344 | 2.22E-10 | 0.202 |
| ENSG00000278060.1 | *RP11-466M21.1* | chr2:72932973-72934355 | NA | no | 4.26E-14 | 0.289 | 9.01E-10 | 0.205 |
| ENSG00000187605.12 | *TET3* | chr2:73986403-74108176 | yes | no | 8.67E-11 | 0.084 | 1.83E-06 | 0.034 |
| ENSG00000239779.3 | *WBP1* | chr2:74458328-74460891 | NA | no | 6.20E-09 | -0.171 | 1.31E-04 | -0.035 |
| ENSG00000042493.12 | *CAPG* | chr2:85394747-85418432 | yes | yes | 1.42E-06 | 0.479 | 3.00E-02 | 0.060 |
| ENSG00000239305.3 | *RNF103* | chr2:86603392-86623866 | yes | no | 1.74E-06 | -0.184 | 3.68E-02 | -0.052 |
| ENSG00000172071.8 | *EIF2AK3* | chr2:88556740-88627576 | yes | yes | 1.69E-11 | -0.362 | 3.58E-07 | -0.054 |
| ENSG00000163121.6 | *NEURL3* | chr2:96497642-96508109 | yes | no | 5.05E-13 | 0.231 | 1.07E-08 | 0.257 |
| ENSG00000196843.12 | *ARID5A* | chr2:96536742-96552638 | yes | no | 9.20E-08 | 0.188 | 1.94E-03 | 0.032 |
| ENSG00000071051.10 | *NCK2* | chr2:105744896-105894274 | yes | no | 6.17E-07 | 0.102 | 1.31E-02 | 0.029 |
| ENSG00000125538.8 | *IL1B* | chr2:112829750-112836903 | no | yes | 1.86E-09 | 0.187 | 3.93E-05 | 0.204 |
| ENSG00000125629.11 | *INSIG2* | chr2:118088451-118110997 | yes | no | 1.10E-06 | -0.110 | 2.32E-02 | -0.026 |
| ENSG00000171227.6 | *TMEM37* | chr2:119429900-119438520 | yes | no | 8.64E-08 | 0.116 | 1.83E-03 | 0.169 |
| ENSG00000115109.10 | *EPB41L5* | chr2:120013004-120179119 | yes | no | 1.38E-06 | 0.208 | 2.92E-02 | 0.073 |
| ENSG00000136720.6 | *HS6ST1* | chr2:128236715-128318577 | NA | no | 9.73E-09 | 0.062 | 2.06E-04 | 0.030 |
| ENSG00000115947.10 | *ORC4* | chr2:147930396-148021604 | NA | no | 1.51E-08 | 0.143 | 3.20E-04 | 0.025 |
| ENSG00000123609.7 | *NMI* | chr2:151270464-151290057 | no | no | 4.95E-16 | 0.267 | 1.05E-11 | 0.042 |
| ENSG00000123610.4 | *TNFAIP6* | chr2:151357591-151380048 | yes | yes | 8.56E-07 | 0.103 | 1.81E-02 | 0.152 |
| ENSG00000054219.10 | *LY75* | chr2:159803354-159904749 | no | yes | 1.52E-12 | 0.309 | 3.22E-08 | 0.054 |
| ENSG00000153250.14 | *RBMS1* | chr2:160272150-160493794 | yes | no | 4.66E-09 | 0.105 | 9.86E-05 | 0.130 |
| ENSG00000197635.6 | *DPP4* | chr2:161992240-162074542 | yes | yes | 3.85E-07 | 0.164 | 8.15E-03 | 0.154 |
| ENSG00000115267.5 | *IFIH1* | chr2:162267078-162318703 | yes | yes | 8.66E-21 | 0.408 | 1.83E-16 | 0.087 |
| ENSG00000178662.12 | *CSRNP3* | chr2:165469646-165689407 | yes | no | 2.00E-07 | 0.059 | 4.24E-03 | 0.242 |
| ENSG00000071967.8 | *CYBRD1* | chr2:171522246-171558133 | yes | no | 2.25E-06 | -0.192 | 4.77E-02 | -0.052 |
| ENSG00000152256.10 | *PDK1* | chr2:172555372-172608669 | yes | no | 6.90E-07 | -0.180 | 1.46E-02 | -0.048 |
| ENSG00000138430.12 | *OLA1* | chr2:174072446-174248698 | yes | no | 2.22E-06 | 0.168 | 4.69E-02 | 0.016 |
| ENSG00000116044.12 | *NFE2L2* | chr2:177227594-177392697 | yes | no | 5.26E-08 | 0.153 | 1.11E-03 | 0.026 |
| ENSG00000115232.10 | *ITGA4* | chr2:181457201-181536187 | no | yes | 1.02E-07 | -0.319 | 2.17E-03 | -0.036 |
| ENSG00000115368.6 | *WDR75* | chr2:189441432-189475565 | yes | no | 2.32E-11 | 0.134 | 4.90E-07 | 0.024 |
| ENSG00000128699.10 | *ORMDL1* | chr2:189770322-189784371 | yes | no | 1.57E-07 | -0.146 | 3.32E-03 | -0.019 |
| ENSG00000115415.15 | *STAT1* | chr2:190964357-191020960 | yes | yes | 3.23E-13 | 0.864 | 6.84E-09 | 0.055 |
| ENSG00000081320.7 | *STK17B* | chr2:196133565-196176503 | yes | yes | 9.44E-07 | -0.229 | 2.00E-02 | -0.045 |
| ENSG00000115541.7 | *HSPE1* | chr2:197499993-197503457 | yes | no | 1.68E-08 | 0.476 | 3.55E-04 | 0.031 |
| ENSG00000162971.7 | *TYW5* | chr2:199929974-199955736 | NA | no | 3.06E-07 | 0.049 | 6.47E-03 | 0.024 |
| ENSG00000138442.6 | *WDR12* | chr2:202874781-203014798 | yes | no | 1.80E-09 | 0.290 | 3.81E-05 | 0.025 |
| ENSG00000118246.10 | *FASTKD2* | chr2:206765356-206792509 | yes | no | 2.43E-11 | 0.104 | 5.13E-07 | 0.022 |
| ENSG00000163251.3 | *FZD5* | chr2:207762585-207769563 | yes | no | 1.69E-09 | 0.067 | 3.56E-05 | 0.107 |
| ENSG00000118242.12 | *MREG* | chr2:215942804-216034096 | yes | no | 3.45E-08 | 0.249 | 7.29E-04 | 0.049 |
| ENSG00000123992.15 | *DNPEP* | chr2:219373545-219400022 | yes | no | 6.12E-12 | 0.328 | 1.29E-07 | 0.036 |
| ENSG00000144591.14 | *GMPPA* | chr2:219498866-219506988 | yes | no | 7.58E-09 | -0.304 | 1.60E-04 | -0.037 |
| ENSG00000163082.9 | *SGPP2* | chr2:222424516-222560948 | yes | no | 1.40E-07 | 0.217 | 2.97E-03 | 0.048 |
| ENSG00000173744.14 | *AGFG1* | chr2:227472151-227561214 | yes | no | 1.43E-07 | 0.145 | 3.03E-03 | 0.018 |
| ENSG00000135916.12 | *ITM2C* | chr2:230864638-230879248 | yes | no | 6.48E-08 | -0.721 | 1.37E-03 | -0.044 |
| ENSG00000173692.9 | *PSMD1* | chr2:231056863-231172827 | yes | yes | 1.66E-09 | 0.210 | 3.50E-05 | 0.022 |
| ENSG00000144476.5 | *ACKR3* | chr2:236567786-236582358 | NA | no | 8.44E-14 | 0.318 | 1.78E-09 | 0.200 |
| ENSG00000132330.13 | *SCLY* | chr2:238060888-238099413 | yes | no | 2.26E-09 | 0.215 | 4.79E-05 | 0.037 |
| ENSG00000225493.1 | *LINC01107* | chr2:238510689-238555054 | NA | no | 7.06E-07 | -0.113 | 1.49E-02 | -0.125 |
| ENSG00000260942.1 | *CAPN10-AS1* | chr2:240582699-240586699 | NA | no | 1.04E-07 | -0.036 | 2.19E-03 | -0.039 |
| ENSG00000134121.6 | *CHL1* | chr3:196595-409417 | yes | no | 1.92E-09 | 0.492 | 4.05E-05 | 0.168 |
| ENSG00000234661.2 | *CHL1-AS1* | chr3:363369-385795 | NA | no | 4.64E-07 | 0.111 | 9.82E-03 | 0.163 |
| ENSG00000091181.16 | *IL5RA* | chr3:3066325-3126613 | no | yes | 5.96E-07 | -0.058 | 1.26E-02 | -0.212 |
| ENSG00000134109.7 | *EDEM1* | chr3:5187645-5219957 | yes | no | 1.78E-06 | -0.272 | 3.77E-02 | -0.032 |
| ENSG00000180914.7 | *OXTR* | chr3:8750407-8769628 | yes | no | 4.78E-11 | 0.356 | 1.01E-06 | 0.139 |
| ENSG00000214021.12 | *TTLL3* | chr3:9808085-9855138 | yes | no | 1.20E-07 | -0.154 | 2.54E-03 | -0.041 |
| ENSG00000163704.8 | *PRRT3* | chr3:9945541-9952394 | yes | no | 4.64E-11 | 0.116 | 9.81E-07 | 0.078 |
| ENSG00000125037.9 | *EMC3* | chr3:9962536-10011116 | NA | no | 8.26E-07 | -0.118 | 1.75E-02 | -0.015 |
| ENSG00000157020.14 | *SEC13* | chr3:10293130-10321178 | yes | no | 1.68E-06 | -0.158 | 3.56E-02 | -0.015 |
| ENSG00000206560.7 | *ANKRD28* | chr3:15667235-15859771 | yes | no | 6.34E-09 | -0.180 | 1.34E-04 | -0.033 |
| ENSG00000154822.12 | *PLCL2* | chr3:16802650-17090594 | yes | yes | 2.15E-08 | -0.140 | 4.54E-04 | -0.038 |
| ENSG00000163527.6 | *STT3B* | chr3:31532637-31637622 | yes | yes | 7.12E-08 | -0.282 | 1.50E-03 | -0.027 |
| ENSG00000206557.5 | *TRIM71* | chr3:32818017-32897826 | NA | no | 2.66E-07 | 0.155 | 5.62E-03 | 0.185 |
| ENSG00000172936.9 | *MYD88* | chr3:38138477-38143022 | yes | yes | 5.81E-09 | 0.183 | 1.23E-04 | 0.026 |
| ENSG00000172939.5 | *OXSR1* | chr3:38165088-38255488 | yes | no | 3.54E-09 | 0.088 | 7.49E-05 | 0.019 |
| ENSG00000179934.6 | *CCR8* | chr3:39329705-39333511 | NA | yes | 4.32E-11 | 0.266 | 9.14E-07 | 0.204 |
| ENSG00000228168.1 | *HNRNPA1P21* | chr3:39334978-39335939 | NA | no | 6.33E-10 | 0.267 | 1.34E-05 | 0.184 |
| ENSG00000144791.6 | *LIMD1* | chr3:45555393-45686338 | yes | yes | 2.34E-14 | -0.265 | 4.95E-10 | -0.054 |
| ENSG00000230530.1 | *LIMD1-AS1* | chr3:45679042-45689134 | NA | no | 2.78E-12 | -0.218 | 5.88E-08 | -0.044 |
| ENSG00000163818.13 | *LZTFL1* | chr3:45823315-45916042 | yes | no | 1.73E-06 | -0.187 | 3.67E-02 | -0.062 |
| ENSG00000121807.5 | *CCR2* | chr3:46353733-46360928 | no | yes | 2.26E-09 | -0.189 | 4.78E-05 | -0.161 |
| ENSG00000160791.13 | *CCR5* | chr3:46370853-46376206 | NA | yes | 2.98E-07 | -0.063 | 6.30E-03 | -0.164 |
| ENSG00000173540.9 | *GMPPB* | chr3:49716843-49723951 | yes | no | 7.47E-09 | -0.216 | 1.58E-04 | -0.031 |
| ENSG00000145050.12 | *MANF* | chr3:51385046-51389397 | yes | no | 1.43E-06 | -0.719 | 3.02E-02 | -0.038 |
| ENSG00000114767.6 | *RRP9* | chr3:51933429-51941941 | yes | no | 9.21E-07 | 0.118 | 1.95E-02 | 0.021 |
| ENSG00000016864.13 | *GLT8D1* | chr3:52694484-52706083 | yes | no | 1.01E-12 | -0.277 | 2.14E-08 | -0.041 |
| ENSG00000114902.10 | *SPCS1* | chr3:52704954-52711146 | yes | no | 1.72E-12 | -0.506 | 3.64E-08 | -0.035 |
| ENSG00000184220.7 | *CMSS1* | chr3:99817833-100178603 | NA | no | 1.44E-06 | 0.145 | 3.04E-02 | 0.025 |
| ENSG00000168386.15 | *FILIP1L* | chr3:99830140-100114513 | no | no | 8.06E-07 | 0.108 | 1.71E-02 | 0.086 |
| ENSG00000114021.8 | *NIT2* | chr3:100334700-100356866 | yes | no | 9.82E-12 | 0.246 | 2.08E-07 | 0.029 |
| ENSG00000031081.7 | *ARHGAP31* | chr3:119294372-119420714 | NA | yes | 3.47E-10 | 0.158 | 7.33E-06 | 0.055 |
| ENSG00000176142.9 | *TMEM39A* | chr3:119429499-119468830 | yes | no | 8.02E-11 | -0.210 | 1.70E-06 | -0.035 |
| ENSG00000121594.8 | *CD80* | chr3:119524292-119559602 | no | yes | 1.30E-13 | 0.261 | 2.76E-09 | 0.061 |
| ENSG00000144837.5 | *PLA1A* | chr3:119597841-119629811 | yes | no | 5.80E-10 | 0.315 | 1.23E-05 | 0.109 |
| ENSG00000160124.6 | *CCDC58* | chr3:122359590-122383231 | NA | no | 1.42E-08 | 0.187 | 3.00E-04 | 0.025 |
| ENSG00000138496.13 | *PARP9* | chr3:122527923-122564577 | yes | no | 2.20E-13 | 0.332 | 4.65E-09 | 0.073 |
| ENSG00000163840.6 | *DTX3L* | chr3:122564237-122575203 | yes | yes | 1.10E-09 | 0.221 | 2.33E-05 | 0.056 |
| ENSG00000173193.10 | *PARP14* | chr3:122680617-122730840 | yes | no | 1.60E-10 | 0.273 | 3.38E-06 | 0.047 |
| ENSG00000065485.14 | *PDIA5* | chr3:123067061-123225227 | no | no | 5.43E-07 | -0.179 | 1.15E-02 | -0.038 |
| ENSG00000058262.6 | *SEC61A1* | chr3:128051640-128071683 | yes | yes | 1.61E-12 | -0.465 | 3.40E-08 | -0.038 |
| ENSG00000163902.8 | *RPN1* | chr3:128619969-128681075 | yes | no | 8.77E-09 | -0.281 | 1.85E-04 | -0.026 |
| ENSG00000181789.11 | *COPG1* | chr3:129249605-129277773 | NA | no | 1.23E-10 | -0.300 | 2.59E-06 | -0.028 |
| ENSG00000184897.5 | *H1FX* | chr3:129314770-129316277 | yes | no | 4.89E-08 | -0.169 | 1.03E-03 | -0.057 |
| ENSG00000206417.5 | *H1FX-AS1* | chr3:129315391-129326225 | NA | no | 6.23E-08 | -0.163 | 1.32E-03 | -0.060 |
| ENSG00000114686.5 | *MRPL3* | chr3:131462211-131502983 | yes | no | 2.64E-07 | 0.210 | 5.57E-03 | 0.019 |
| ENSG00000196353.8 | *CPNE4* | chr3:131533554-132285410 | yes | no | 2.86E-09 | 0.103 | 6.04E-05 | 0.255 |
| ENSG00000144868.10 | *TMEM108* | chr3:133038390-133397792 | yes | no | 4.43E-08 | -0.101 | 9.36E-04 | -0.118 |
| ENSG00000249993.1 | *BFSP2-AS1* | chr3:133429268-133455776 | NA | no | 1.90E-10 | -0.114 | 4.02E-06 | -0.123 |
| ENSG00000144867.8 | *SRPRB* | chr3:133784032-133825772 | yes | no | 5.32E-08 | -0.227 | 1.13E-03 | -0.027 |
| ENSG00000066405.9 | *CLDN18* | chr3:137998734-138033655 | yes | no | 2.44E-08 | 0.041 | 5.15E-04 | 0.247 |
| ENSG00000177311.7 | *ZBTB38* | chr3:141324212-141449792 | NA | no | 1.07E-09 | -0.763 | 2.26E-05 | -0.080 |
| ENSG00000175040.4 | *CHST2* | chr3:143119330-143122958 | yes | yes | 3.21E-07 | -0.284 | 6.78E-03 | -0.078 |
| ENSG00000188313.9 | *PLSCR1* | chr3:146515179-146544864 | yes | yes | 5.55E-14 | 0.583 | 1.17E-09 | 0.085 |
| ENSG00000120742.7 | *SERP1* | chr3:150541993-150603228 | yes | no | 1.68E-08 | -0.355 | 3.54E-04 | -0.027 |
| ENSG00000198829.6 | *SUCNR1* | chr3:151873642-151884619 | no | yes | 2.21E-06 | 0.144 | 4.67E-02 | 0.168 |
| ENSG00000169359.10 | *SLC33A1* | chr3:155821023-155854429 | yes | no | 1.64E-06 | -0.115 | 3.47E-02 | -0.028 |
| ENSG00000114850.3 | *SSR3* | chr3:156540139-156555184 | yes | no | 2.02E-07 | -0.415 | 4.26E-03 | -0.032 |
| ENSG00000008952.13 | *SEC62* | chr3:169966634-169998373 | yes | no | 1.13E-10 | -0.333 | 2.40E-06 | -0.031 |
| ENSG00000240373.1 | *SEC62-AS1* | chr3:169978535-169985715 | NA | no | 3.67E-07 | -0.040 | 7.76E-03 | -0.031 |
| ENSG00000173890.13 | *GPR160* | chr3:170037928-170085403 | yes | no | 1.84E-07 | -0.463 | 3.88E-03 | -0.060 |
| ENSG00000121858.7 | *TNFSF10* | chr3:172505507-172523507 | yes | yes | 1.16E-10 | 0.301 | 2.44E-06 | 0.092 |
| ENSG00000136522.10 | *MRPL47* | chr3:179588284-179604654 | yes | no | 1.47E-08 | 0.140 | 3.11E-04 | 0.018 |
| ENSG00000172578.8 | *KLHL6* | chr3:183487530-183555689 | no | yes | 6.48E-11 | -0.196 | 1.37E-06 | -0.104 |
| ENSG00000114796.12 | *KLHL24* | chr3:183635567-183684477 | yes | no | 7.91E-07 | -0.098 | 1.67E-02 | -0.058 |
| ENSG00000163900.7 | *TMEM41A* | chr3:185476495-185499057 | yes | no | 5.73E-09 | -0.086 | 1.21E-04 | -0.023 |
| ENSG00000090520.7 | *DNAJB11* | chr3:186567402-186597203 | yes | no | 1.59E-06 | -0.379 | 3.36E-02 | -0.030 |
| ENSG00000073849.11 | *ST6GAL1* | chr3:186930484-187078553 | yes | yes | 1.59E-16 | -0.528 | 3.37E-12 | -0.051 |
| ENSG00000136514.2 | *RTP4* | chr3:187368331-187372076 | yes | no | 1.13E-13 | 0.152 | 2.40E-09 | 0.086 |
| ENSG00000184203.4 | *PPP1R2* | chr3:195514424-195543386 | yes | no | 3.98E-07 | -0.142 | 8.41E-03 | -0.021 |
| ENSG00000127419.13 | *TMEM175* | chr4:932386-958656 | yes | no | 2.29E-06 | -0.207 | 4.85E-02 | -0.039 |
| ENSG00000123933.11 | *MXD4* | chr4:2247431-2262294 | yes | no | 3.37E-09 | -0.222 | 7.13E-05 | -0.044 |
| ENSG00000145220.10 | *LYAR* | chr4:4267700-4290169 | yes | yes | 4.40E-08 | 0.162 | 9.30E-04 | 0.025 |
| ENSG00000163132.6 | *MSX1* | chr4:4859665-4863936 | yes | no | 4.81E-08 | 0.288 | 1.02E-03 | 0.188 |
| ENSG00000109519.9 | *GRPEL1* | chr4:7058905-7068197 | yes | no | 8.83E-07 | 0.132 | 1.87E-02 | 0.019 |
| ENSG00000109684.11 | *CLNK* | chr4:10486394-10684865 | no | yes | 1.93E-10 | 0.190 | 4.09E-06 | 0.122 |
| ENSG00000002587.6 | *HS3ST1* | chr4:11393149-11429765 | yes | no | 1.20E-14 | 0.249 | 2.53E-10 | 0.322 |
| ENSG00000002549.9 | *LAP3* | chr4:17577191-17607972 | yes | no | 5.91E-07 | 0.337 | 1.25E-02 | 0.027 |
| ENSG00000249502.1 | *AC006160.5* | chr4:17587466-17614571 | NA | no | 7.86E-12 | 0.065 | 1.66E-07 | 0.033 |
| ENSG00000163697.13 | *APBB2* | chr4:40810026-41216714 | yes | no | 1.72E-07 | -0.247 | 3.64E-03 | -0.070 |
| ENSG00000250906.1 | *RP11-632F7.3* | chr4:40812778-40826151 | NA | no | 1.41E-06 | -0.061 | 2.99E-02 | -0.068 |
| ENSG00000188848.12 | *BEND4* | chr4:42110937-42152878 | yes | no | 6.05E-07 | 0.236 | 1.28E-02 | 0.107 |
| ENSG00000174799.7 | *CEP135* | chr4:55948870-56033363 | yes | no | 8.01E-08 | 0.055 | 1.69E-03 | 0.023 |
| ENSG00000138768.11 | *USO1* | chr4:75724592-75814229 | yes | yes | 1.36E-08 | -0.240 | 2.88E-04 | -0.027 |
| ENSG00000138760.5 | *SCARB2* | chr4:76158736-76213893 | yes | no | 9.76E-09 | -0.239 | 2.06E-04 | -0.060 |
| ENSG00000138764.10 | *CCNG2* | chr4:77157150-77433388 | yes | no | 2.39E-09 | -0.134 | 5.06E-05 | -0.045 |
| ENSG00000138670.13 | *RASGEF1B* | chr4:81426392-82044244 | yes | no | 1.85E-06 | 0.070 | 3.92E-02 | 0.179 |
| ENSG00000152795.14 | *HNRNPDL* | chr4:82422563-82430225 | NA | no | 1.46E-07 | 0.227 | 3.09E-03 | 0.015 |
| ENSG00000145293.11 | *ENOPH1* | chr4:82430561-82461091 | yes | no | 1.88E-08 | 0.099 | 3.98E-04 | 0.017 |
| ENSG00000145287.7 | *PLAC8* | chr4:83090047-83137075 | no | no | 1.62E-07 | 1.001 | 3.42E-03 | 0.077 |
| ENSG00000173083.11 | *HPSE* | chr4:83292460-83335153 | no | yes | 3.32E-07 | 0.119 | 7.02E-03 | 0.113 |
| ENSG00000138642.11 | *HERC6* | chr4:88378738-88443111 | yes | no | 2.38E-13 | 0.243 | 5.03E-09 | 0.108 |
| ENSG00000138646.5 | *HERC5* | chr4:88457116-88506163 | yes | yes | 3.97E-13 | 0.354 | 8.39E-09 | 0.087 |
| ENSG00000184305.11 | *CCSER1* | chr4:90127534-91601913 | NA | no | 2.27E-09 | 0.126 | 4.79E-05 | 0.151 |
| ENSG00000109320.8 | *NFKB1* | chr4:102501328-102617302 | yes | yes | 8.44E-14 | 0.271 | 1.78E-09 | 0.037 |
| ENSG00000155016.14 | *CYP2U1* | chr4:107931368-107953457 | yes | no | 1.56E-11 | 0.101 | 3.30E-07 | 0.082 |
| ENSG00000138796.12 | *HADH* | chr4:107989713-108035175 | yes | no | 1.77E-08 | 0.166 | 3.75E-04 | 0.024 |
| ENSG00000178403.3 | *NEUROG2* | chr4:112513515-112516172 | yes | no | 2.30E-07 | 0.154 | 4.86E-03 | 0.144 |
| ENSG00000174607.7 | *UGT8* | chr4:114598454-114678224 | yes | yes | 6.49E-09 | 0.087 | 1.37E-04 | 0.155 |
| ENSG00000150961.11 | *SEC24D* | chr4:118722822-118838683 | yes | yes | 1.21E-08 | -0.265 | 2.57E-04 | -0.037 |
| ENSG00000123737.9 | *EXOSC9* | chr4:121801316-121817021 | yes | yes | 3.02E-09 | 0.162 | 6.39E-05 | 0.022 |
| ENSG00000164070.8 | *HSPA4L* | chr4:127781820-127840733 | yes | no | 1.27E-06 | 0.107 | 2.69E-02 | 0.032 |
| ENSG00000179387.6 | *ELMOD2* | chr4:140524157-140553770 | yes | no | 3.70E-09 | -0.119 | 7.83E-05 | -0.036 |
| ENSG00000164136.13 | *IL15* | chr4:141636598-141733987 | no | yes | 7.19E-07 | 0.149 | 1.52E-02 | 0.053 |
| ENSG00000164161.6 | *HHIP* | chr4:144646020-144745271 | yes | yes | 1.27E-06 | 0.125 | 2.69E-02 | 0.432 |
| ENSG00000164162.9 | *ANAPC10* | chr4:144967111-145098541 | yes | no | 1.62E-06 | 0.100 | 3.43E-02 | 0.024 |
| ENSG00000164164.12 | *OTUD4* | chr4:145110837-145180161 | yes | no | 5.75E-09 | 0.089 | 1.22E-04 | 0.026 |
| ENSG00000121210.12 | *KIAA0922* | chr4:153466345-153636711 | yes | no | 5.79E-09 | 0.160 | 1.23E-04 | 0.029 |
| ENSG00000137462.6 | *TLR2* | chr4:153701499-153705699 | yes | yes | 9.50E-07 | 0.067 | 2.01E-02 | 0.144 |
| ENSG00000171497.4 | *PPID* | chr4:158709133-158723396 | yes | yes | 8.29E-08 | 0.145 | 1.75E-03 | 0.023 |
| ENSG00000137628.13 | *DDX60* | chr4:168216292-168318807 | yes | no | 9.56E-16 | 0.332 | 2.02E-11 | 0.145 |
| ENSG00000181381.10 | *DDX60L* | chr4:168356734-168537786 | NA | no | 1.53E-10 | 0.179 | 3.23E-06 | 0.110 |
| ENSG00000129128.9 | *SPCS3* | chr4:176319963-176332245 | yes | no | 9.26E-08 | -0.279 | 1.96E-03 | -0.029 |
| ENSG00000164305.14 | *CASP3* | chr4:184627695-184649509 | yes | yes | 4.26E-09 | -0.220 | 9.00E-05 | -0.027 |
| ENSG00000249679.1 | *RP11-279O9.4* | chr4:185471515-185472263 | NA | no | 1.74E-08 | -0.228 | 3.69E-04 | -0.103 |
| ENSG00000049656.10 | *CLPTM1L* | chr5:1317743-1345099 | yes | yes | 3.87E-12 | -0.815 | 8.17E-08 | -0.062 |
| ENSG00000271980.1 | *CTD-2256P15.4* | chr5:10264596-10267146 | NA | no | 2.37E-08 | 0.103 | 5.01E-04 | 0.021 |
| ENSG00000272016.1 | *RP11-215G15.5* | chr5:10654219-10657816 | NA | no | 8.48E-10 | 0.156 | 1.79E-05 | 0.032 |
| ENSG00000154122.9 | *ANKH* | chr5:14704803-14871778 | yes | yes | 1.25E-07 | 0.273 | 2.65E-03 | 0.062 |
| ENSG00000154153.10 | *FAM134B* | chr5:16473037-16617058 | yes | no | 2.23E-07 | 0.151 | 4.73E-03 | 0.158 |
| ENSG00000113384.10 | *GOLPH3* | chr5:32124703-32174350 | yes | no | 1.81E-07 | -0.116 | 3.82E-03 | -0.018 |
| ENSG00000113387.8 | *SUB1* | chr5:32531632-32604079 | yes | no | 2.26E-12 | -1.610 | 4.78E-08 | -0.051 |
| ENSG00000113460.9 | *BRIX1* | chr5:34915375-34925996 | NA | no | 1.68E-06 | 0.136 | 3.54E-02 | 0.018 |
| ENSG00000113494.13 | *PRLR* | chr5:35048755-35230589 | yes | yes | 1.22E-06 | 0.107 | 2.57E-02 | 0.162 |
| ENSG00000112972.11 | *HMGCS1* | chr5:43289394-43313512 | yes | no | 2.30E-07 | 0.131 | 4.87E-03 | 0.024 |
| ENSG00000170571.8 | *EMB* | chr5:50396191-50443248 | yes | no | 2.66E-07 | 0.222 | 5.62E-03 | 0.059 |
| ENSG00000151883.13 | *PARP8* | chr5:50665898-50846522 | yes | no | 6.30E-07 | 0.142 | 1.33E-02 | 0.032 |
| ENSG00000213949.5 | *ITGA1* | chr5:52787895-52959210 | yes | yes | 1.53E-07 | 0.199 | 3.23E-03 | 0.146 |
| ENSG00000152684.10 | *PELO* | chr5:52787939-52804046 | NA | no | 6.17E-07 | 0.093 | 1.31E-02 | 0.031 |
| ENSG00000249899.2 | *CTD-2175A23.1* | chr5:52932418-52990278 | NA | no | 1.10E-10 | 0.042 | 2.33E-06 | 0.181 |
| ENSG00000086189.6 | *DIMT1* | chr5:62387253-62403939 | NA | no | 7.10E-07 | 0.098 | 1.50E-02 | 0.020 |
| ENSG00000248664.1 | *CTC-498J12.3* | chr5:69113111-69136394 | NA | no | 3.59E-07 | -0.064 | 7.59E-03 | -0.024 |
| ENSG00000172062.13 | *SMN1* | chr5:70925029-70953942 | NA | no | 7.86E-07 | 0.056 | 1.66E-02 | 0.034 |
| ENSG00000081189.10 | *MEF2C* | chr5:88718157-88904105 | yes | yes | 7.04E-10 | -0.314 | 1.49E-05 | -0.031 |
| ENSG00000118985.11 | *ELL2* | chr5:95885097-95962071 | yes | no | 2.73E-07 | -0.286 | 5.76E-03 | -0.041 |
| ENSG00000113441.12 | *LNPEP* | chr5:96935393-97037515 | yes | yes | 4.70E-08 | 0.140 | 9.94E-04 | 0.041 |
| ENSG00000134970.13 | *TMED7* | chr5:115613507-115632992 | NA | no | 3.37E-07 | -0.128 | 7.12E-03 | -0.027 |
| ENSG00000092421.13 | *SEMA6A* | chr5:116443615-116574934 | yes | no | 9.58E-07 | 0.069 | 2.03E-02 | 0.077 |
| ENSG00000168938.5 | *PPIC* | chr5:123023249-123036741 | yes | yes | 9.37E-07 | 0.119 | 1.98E-02 | 0.158 |
| ENSG00000066583.8 | *ISOC1* | chr5:129094750-129114028 | yes | no | 8.98E-11 | 0.153 | 1.90E-06 | 0.038 |
| ENSG00000125347.10 | *IRF1* | chr5:132481608-132490798 | yes | yes | 1.45E-08 | 0.298 | 3.07E-04 | 0.042 |
| ENSG00000213585.7 | *VDAC1* | chr5:133971914-134005133 | yes | no | 7.32E-07 | 0.195 | 1.55E-02 | 0.015 |
| ENSG00000113558.15 | *SKP1* | chr5:134148934-134177038 | yes | yes | 5.18E-08 | 0.227 | 1.10E-03 | 0.015 |
| ENSG00000043143.17 | *JADE2* | chr5:134524311-134583230 | NA | no | 8.28E-07 | 0.158 | 1.75E-02 | 0.030 |
| ENSG00000113615.9 | *SEC24A* | chr5:134648788-134727823 | yes | no | 6.26E-08 | -0.130 | 1.32E-03 | -0.027 |
| ENSG00000113013.9 | *HSPA9* | chr5:138554881-138575444 | yes | no | 2.35E-06 | 0.293 | 4.97E-02 | 0.013 |
| ENSG00000170476.12 | *MZB1* | chr5:139387479-139390081 | NA | no | 2.54E-11 | -1.080 | 5.37E-07 | -0.061 |
| ENSG00000120306.6 | *CYSTM1* | chr5:140174641-140282052 | NA | no | 2.33E-07 | 0.265 | 4.93E-03 | 0.046 |
| ENSG00000113070.7 | *HBEGF* | chr5:140332842-140346631 | yes | yes | 1.62E-06 | 0.064 | 3.43E-02 | 0.089 |
| ENSG00000240184.3 | *PCDHGC3* | chr5:141475946-141512979 | NA | no | 2.94E-08 | -0.253 | 6.23E-04 | -0.103 |
| ENSG00000187678.8 | *SPRY4* | chr5:142310426-142326455 | yes | no | 1.22E-08 | 0.123 | 2.58E-04 | 0.123 |
| ENSG00000145817.13 | *YIPF5* | chr5:144158158-144170714 | yes | yes | 1.83E-09 | -0.147 | 3.87E-05 | -0.025 |
| ENSG00000171992.9 | *SYNPO* | chr5:150601079-150659220 | yes | no | 8.11E-07 | 0.216 | 1.71E-02 | 0.071 |
| ENSG00000145901.11 | *TNIP1* | chr5:151029944-151093577 | yes | yes | 2.13E-11 | 0.484 | 4.51E-07 | 0.038 |
| ENSG00000251405.2 | *CTB-109A12.1* | chr5:157362614-157460078 | NA | no | 2.07E-11 | 0.022 | 4.37E-07 | 0.027 |
| ENSG00000113282.10 | *CLINT1* | chr5:157785742-157859175 | yes | no | 3.87E-15 | -0.367 | 8.18E-11 | -0.051 |
| ENSG00000253522.3 | *MIR146A* | chr5:160468267-160487426 | NA | no | 1.61E-09 | 0.239 | 3.40E-05 | 0.070 |
| ENSG00000169258.6 | *GPRIN1* | chr5:176595801-176610133 | yes | no | 1.45E-07 | 0.082 | 3.06E-03 | 0.046 |
| ENSG00000169230.6 | *PRELID1* | chr5:177303773-177306959 | NA | yes | 5.18E-09 | 0.287 | 1.10E-04 | 0.022 |
| ENSG00000169220.14 | *RGS14* | chr5:177357836-177372601 | yes | yes | 1.75E-08 | 0.239 | 3.70E-04 | 0.037 |
| ENSG00000146094.10 | *DOK3* | chr5:177501906-177511274 | yes | yes | 1.03E-06 | -0.238 | 2.18E-02 | -0.028 |
| ENSG00000145911.5 | *N4BP3* | chr5:178113442-178126087 | yes | no | 1.01E-06 | 0.055 | 2.13E-02 | 0.031 |
| ENSG00000127022.11 | *CANX* | chr5:179678627-179730925 | yes | yes | 4.47E-08 | -0.524 | 9.45E-04 | -0.024 |
| ENSG00000131446.12 | *MGAT1* | chr5:180790540-180815652 | yes | no | 7.78E-08 | -0.151 | 1.65E-03 | -0.019 |
| ENSG00000188996.4 | *HUS1B* | chr6:655938-656963 | yes | no | 6.83E-07 | 0.060 | 1.44E-02 | 0.197 |
| ENSG00000228170.1 | *RP1-40E16.11* | chr6:3138393-3153062 | NA | no | 4.79E-07 | 0.096 | 1.01E-02 | 0.045 |
| ENSG00000124491.12 | *F13A1* | chr6:6144084-6321013 | yes | no | 5.28E-07 | 0.589 | 1.12E-02 | 0.206 |
| ENSG00000124783.9 | *SSR1* | chr6:7268305-7347446 | yes | no | 4.36E-11 | -0.438 | 9.22E-07 | -0.037 |
| ENSG00000238221.1 | *RP11-69L16.4* | chr6:7276030-7298872 | NA | no | 2.84E-07 | -0.131 | 6.00E-03 | -0.036 |
| ENSG00000239264.5 | *TXNDC5* | chr6:7881516-7910814 | yes | no | 1.14E-11 | -1.569 | 2.42E-07 | -0.081 |
| ENSG00000205269.5 | *TMEM170B* | chr6:11538277-11583524 | NA | no | 1.17E-08 | 0.089 | 2.47E-04 | 0.061 |
| ENSG00000095951.13 | *HIVEP1* | chr6:12008761-12164999 | yes | yes | 7.58E-07 | 0.097 | 1.60E-02 | 0.030 |
| ENSG00000225921.3 | *NOL7* | chr6:13615326-13632739 | yes | no | 7.10E-08 | 0.131 | 1.50E-03 | 0.015 |
| ENSG00000137414.5 | *FAM8A1* | chr6:17600354-17611719 | yes | no | 1.59E-06 | -0.041 | 3.36E-02 | -0.024 |
| ENSG00000124789.8 | *NUP153* | chr6:17615034-17706834 | yes | no | 7.35E-08 | 0.099 | 1.55E-03 | 0.018 |
| ENSG00000213886.3 | *UBD* | chr6:29555514-29559925 | no | no | 1.08E-12 | 0.732 | 2.28E-08 | 0.206 |
| ENSG00000204642.10 | *HLA-F* | chr6:29722774-29738528 | yes | yes | 1.96E-10 | 0.626 | 4.15E-06 | 0.043 |
| ENSG00000225864.1 | *HCG4P11* | chr6:29722980-29723971 | NA | no | 2.18E-08 | 0.247 | 4.61E-04 | 0.039 |
| ENSG00000204619.4 | *PPP1R11* | chr6:30066708-30070333 | yes | no | 1.55E-07 | 0.088 | 3.29E-03 | 0.017 |
| ENSG00000137404.11 | *NRM* | chr6:30688046-30691420 | yes | no | 1.62E-06 | 0.151 | 3.43E-02 | 0.028 |
| ENSG00000137331.11 | *IER3* | chr6:30743198-30744554 | yes | yes | 5.51E-07 | 0.270 | 1.16E-02 | 0.072 |
| ENSG00000204386.7 | *NEU1* | chr6:31857658-31862906 | yes | yes | 4.70E-11 | -0.117 | 9.93E-07 | -0.029 |
| ENSG00000213676.7 | *ATF6B* | chr6:32098175-32128253 | NA | no | 1.28E-06 | -0.179 | 2.70E-02 | -0.018 |
| ENSG00000204310.7 | *AGPAT1* | chr6:32168211-32178096 | yes | no | 1.15E-09 | -0.096 | 2.43E-05 | -0.017 |
| ENSG00000240065.4 | *PSMB9* | chr6:32844135-32859585 | yes | yes | 8.86E-12 | 0.602 | 1.87E-07 | 0.041 |
| ENSG00000168394.10 | *TAP1* | chr6:32845208-32853978 | yes | yes | 3.20E-14 | 0.395 | 6.76E-10 | 0.040 |
| ENSG00000204248.7 | *COL11A2* | chr6:33162680-33192499 | yes | no | 3.57E-11 | 0.125 | 7.55E-07 | 0.179 |
| ENSG00000112473.13 | *SLC39A7* | chr6:33200444-33204439 | yes | no | 1.43E-12 | -0.260 | 3.01E-08 | -0.031 |
| ENSG00000112079.8 | *STK38* | chr6:36493891-36547470 | yes | no | 5.55E-07 | -0.100 | 1.17E-02 | -0.024 |
| ENSG00000137168.7 | *PPIL1* | chr6:36854826-36875024 | yes | no | 1.79E-06 | 0.101 | 3.78E-02 | 0.016 |
| ENSG00000198663.13 | *C6orf89* | chr6:36871869-36928964 | yes | no | 1.37E-07 | -0.086 | 2.90E-03 | -0.019 |
| ENSG00000137200.9 | *CMTR1* | chr6:37433218-37482827 | NA | no | 4.70E-08 | 0.113 | 9.95E-04 | 0.018 |
| ENSG00000124767.6 | *GLO1* | chr6:38675924-38703141 | yes | no | 2.21E-06 | 0.154 | 4.68E-02 | 0.020 |
| ENSG00000112195.8 | *TREML2* | chr6:41190276-41201194 | yes | no | 1.22E-06 | -0.130 | 2.58E-02 | -0.077 |
| ENSG00000112561.14 | *TFEB* | chr6:41683977-41736259 | yes | yes | 1.71E-14 | 0.363 | 3.61E-10 | 0.043 |
| ENSG00000221821.3 | *C6orf226* | chr6:42890264-42890816 | NA | no | 1.91E-06 | -0.093 | 4.04E-02 | -0.030 |
| ENSG00000146215.10 | *CRIP3* | chr6:43299709-43308797 | yes | yes | 5.44E-07 | 0.172 | 1.15E-02 | 0.066 |
| ENSG00000171467.12 | *ZNF318* | chr6:43307133-43369478 | yes | no | 8.02E-10 | 0.231 | 1.70E-05 | 0.048 |
| ENSG00000112759.13 | *SLC29A1* | chr6:44219504-44234151 | yes | no | 1.86E-07 | 0.144 | 3.93E-03 | 0.025 |
| ENSG00000096384.16 | *HSP90AB1* | chr6:44246165-44253888 | yes | yes | 5.71E-11 | 0.533 | 1.21E-06 | 0.022 |
| ENSG00000146232.11 | *NFKBIE* | chr6:44258165-44265788 | yes | yes | 3.04E-14 | 0.438 | 6.43E-10 | 0.060 |
| ENSG00000198087.7 | *CD2AP* | chr6:47477788-47627263 | yes | yes | 8.09E-08 | 0.109 | 1.71E-03 | 0.024 |
| ENSG00000065308.4 | *TRAM2* | chr6:52497401-52576915 | yes | no | 1.25E-06 | -0.139 | 2.65E-02 | -0.042 |
| ENSG00000096092.5 | *TMEM14A* | chr6:52671108-52686588 | yes | no | 1.39E-06 | 0.117 | 2.93E-02 | 0.033 |
| ENSG00000112208.11 | *BAG2* | chr6:57172325-57189833 | yes | no | 1.96E-07 | 0.047 | 4.15E-03 | 0.020 |
| ENSG00000082269.13 | *FAM135A* | chr6:70412940-70561174 | yes | no | 1.07E-09 | 0.108 | 2.26E-05 | 0.048 |
| ENSG00000083097.11 | *DOPEY1* | chr6:83067665-83171350 | yes | no | 5.93E-07 | 0.096 | 1.25E-02 | 0.042 |
| ENSG00000013392.7 | *RWDD2A* | chr6:83193378-83198932 | yes | no | 1.35E-10 | -0.060 | 2.85E-06 | -0.040 |
| ENSG00000146278.10 | *PNRC1* | chr6:89080750-89085160 | yes | no | 8.25E-10 | -0.201 | 1.74E-05 | -0.035 |
| ENSG00000198833.6 | *UBE2J1* | chr6:89326624-89352848 | yes | yes | 1.36E-11 | -0.488 | 2.87E-07 | -0.048 |
| ENSG00000123545.5 | *NDUFAF4* | chr6:96889312-96897881 | yes | no | 4.78E-12 | 0.219 | 1.01E-07 | 0.030 |
| ENSG00000025796.10 | *SEC63* | chr6:107867755-107958189 | yes | yes | 1.46E-10 | -0.274 | 3.08E-06 | -0.028 |
| ENSG00000112365.4 | *ZBTB24* | chr6:109462593-109483237 | NA | no | 2.95E-10 | 0.049 | 6.24E-06 | 0.020 |
| ENSG00000155115.6 | *GTF3C6* | chr6:110958559-110967890 | yes | no | 1.00E-07 | 0.142 | 2.12E-03 | 0.016 |
| ENSG00000197498.9 | *RPF2* | chr6:110982014-111028263 | NA | no | 1.28E-08 | 0.154 | 2.71E-04 | 0.025 |
| ENSG00000187189.10 | *TSPYL4* | chr6:116249960-116254140 | yes | no | 4.18E-08 | 0.076 | 8.83E-04 | 0.028 |
| ENSG00000214338.7 | *SOGA3* | chr6:127472793-127519191 | NA | no | 1.13E-06 | 0.078 | 2.39E-02 | 0.149 |
| ENSG00000152894.11 | *PTPRK* | chr6:127968778-128520674 | yes | yes | 1.59E-06 | 0.286 | 3.37E-02 | 0.138 |
| ENSG00000118515.8 | *SGK1* | chr6:134169245-134318112 | yes | no | 7.35E-07 | -0.213 | 1.55E-02 | -0.089 |
| ENSG00000118503.11 | *TNFAIP3* | chr6:137867187-137883312 | no | yes | 4.96E-08 | 0.378 | 1.05E-03 | 0.043 |
| ENSG00000164442.9 | *CITED2* | chr6:139371806-139374620 | yes | yes | 4.17E-07 | -0.222 | 8.82E-03 | -0.066 |
| ENSG00000118495.15 | *PLAGL1* | chr6:143940299-144064599 | yes | no | 9.17E-09 | 0.137 | 1.94E-04 | 0.054 |
| ENSG00000178199.10 | *ZC3H12D* | chr6:149447657-149485061 | NA | no | 3.02E-10 | 0.203 | 6.39E-06 | 0.071 |
| ENSG00000186625.10 | *KATNA1* | chr6:149594872-149648972 | yes | no | 1.16E-06 | 0.074 | 2.45E-02 | 0.019 |
| ENSG00000120278.11 | *PLEKHG1* | chr6:150599862-150843665 | NA | no | 2.17E-10 | 0.124 | 4.58E-06 | 0.128 |
| ENSG00000146425.7 | *DYNLT1* | chr6:158636473-158644739 | yes | no | 4.32E-11 | 0.279 | 9.14E-07 | 0.050 |
| ENSG00000272841.1 | *RP3-428L16.2* | chr6:160990317-160992342 | NA | no | 2.95E-08 | 0.112 | 6.25E-04 | 0.080 |
| ENSG00000198818.6 | *SFT2D1* | chr6:166319727-166342591 | yes | no | 1.56E-06 | 0.176 | 3.29E-02 | 0.021 |
| ENSG00000112486.11 | *CCR6* | chr6:167122768-167139696 | no | yes | 1.99E-09 | 0.264 | 4.20E-05 | 0.099 |
| ENSG00000198719.7 | *DLL1* | chr6:170282205-170290473 | yes | yes | 9.95E-08 | 0.072 | 2.10E-03 | 0.161 |
| ENSG00000106266.5 | *SNX8* | chr7:2251769-2354318 | yes | no | 1.87E-10 | 0.329 | 3.95E-06 | 0.032 |
| ENSG00000075618.14 | *FSCN1* | chr7:5592822-5606655 | yes | no | 9.20E-13 | 0.899 | 1.95E-08 | 0.081 |
| ENSG00000106305.6 | *AIMP2* | chr7:6009244-6023834 | yes | yes | 6.19E-10 | 0.113 | 1.31E-05 | 0.023 |
| ENSG00000136240.6 | *KDELR2* | chr7:6445952-6484242 | yes | no | 3.69E-08 | -0.383 | 7.80E-04 | -0.036 |
| ENSG00000106415.9 | *GLCCI1* | chr7:7968793-8094272 | yes | no | 2.25E-09 | -0.365 | 4.75E-05 | -0.056 |
| ENSG00000136261.11 | *BZW2* | chr7:16646130-16706523 | yes | no | 1.92E-07 | -0.207 | 4.06E-03 | -0.082 |
| ENSG00000050344.8 | *NFE2L3* | chr7:26152239-26187125 | yes | no | 1.22E-07 | 0.228 | 2.58E-03 | 0.051 |
| ENSG00000086300.12 | *SNX10* | chr7:26291894-26374329 | yes | no | 1.41E-07 | 0.224 | 2.99E-03 | 0.055 |
| ENSG00000225792.1 | *AC004540.4* | chr7:26372143-26376701 | NA | no | 3.05E-09 | 0.142 | 6.45E-05 | 0.062 |
| ENSG00000106588.7 | *PSMA2* | chr7:42916856-42932223 | yes | yes | 1.21E-09 | 0.294 | 2.56E-05 | 0.018 |
| ENSG00000106591.3 | *MRPL32* | chr7:42932199-42948958 | yes | no | 4.08E-07 | 0.127 | 8.63E-03 | 0.018 |
| ENSG00000106605.7 | *BLVRA* | chr7:43758679-43807342 | yes | no | 3.56E-08 | 0.377 | 7.53E-04 | 0.053 |
| ENSG00000158604.11 | *TMED4* | chr7:44577893-44582287 | yes | no | 6.92E-08 | -0.121 | 1.46E-03 | -0.020 |
| ENSG00000122515.11 | *ZMIZ2* | chr7:44748580-44769881 | yes | no | 1.87E-08 | 0.247 | 3.96E-04 | 0.031 |
| ENSG00000183696.10 | *UPP1* | chr7:48088627-48108733 | yes | yes | 5.78E-11 | 0.271 | 1.22E-06 | 0.100 |
| ENSG00000129103.14 | *SUMF2* | chr7:56064001-56080670 | yes | no | 1.85E-08 | -0.160 | 3.91E-04 | -0.019 |
| ENSG00000127951.5 | *FGL2* | chr7:77193370-77199826 | yes | yes | 1.32E-06 | 0.142 | 2.78E-02 | 0.078 |
| ENSG00000005469.8 | *CROT* | chr7:87345680-87399795 | yes | no | 3.37E-09 | 0.102 | 7.12E-05 | 0.039 |
| ENSG00000164715.5 | *LMTK2* | chr7:98106884-98209633 | yes | no | 5.01E-08 | -0.059 | 1.06E-03 | -0.031 |
| ENSG00000180535.3 | *BHLHA15* | chr7:98211426-98212979 | yes | no | 5.54E-09 | -0.322 | 1.17E-04 | -0.101 |
| ENSG00000241685.5 | *ARPC1A* | chr7:99325897-99388164 | yes | yes | 2.77E-10 | 0.146 | 5.85E-06 | 0.023 |
| ENSG00000160868.11 | *CYP3A4* | chr7:99756959-99784265 | no | no | 7.04E-07 | 0.055 | 1.49E-02 | 0.143 |
| ENSG00000085514.12 | *PILRA* | chr7:100367529-100400099 | yes | yes | 1.39E-06 | 0.160 | 2.94E-02 | 0.091 |
| ENSG00000106367.10 | *AP1S1* | chr7:101154396-101161596 | yes | no | 4.08E-08 | 0.155 | 8.63E-04 | 0.024 |
| ENSG00000105835.8 | *NAMPT* | chr7:106248284-106286326 | yes | yes | 9.88E-11 | 0.336 | 2.09E-06 | 0.047 |
| ENSG00000105974.8 | *CAV1* | chr7:116524784-116561184 | yes | yes | 5.10E-07 | -0.194 | 1.08E-02 | -0.106 |
| ENSG00000128595.13 | *CALU* | chr7:128739291-128771807 | yes | no | 1.90E-11 | -0.230 | 4.03E-07 | -0.032 |
| ENSG00000186591.8 | *UBE2H* | chr7:129830731-129952949 | yes | yes | 9.22E-07 | -0.137 | 1.95E-02 | -0.025 |
| ENSG00000106554.8 | *CHCHD3* | chr7:132784867-133082088 | yes | no | 3.09E-07 | 0.135 | 6.54E-03 | 0.016 |
| ENSG00000172331.8 | *BPGM* | chr7:134646807-134679813 | yes | no | 8.45E-08 | 0.106 | 1.79E-03 | 0.033 |
| ENSG00000182158.11 | *CREB3L2* | chr7:137874978-138002067 | yes | no | 7.00E-13 | -0.252 | 1.48E-08 | -0.047 |
| ENSG00000064393.12 | *HIPK2* | chr7:139561569-139777778 | yes | no | 9.76E-08 | -0.190 | 2.06E-03 | -0.050 |
| ENSG00000059378.9 | *PARP12* | chr7:140023743-140063721 | yes | no | 7.05E-11 | 0.277 | 1.49E-06 | 0.072 |
| ENSG00000244701.1 | *RP5-894A10.2* | chr7:141652380-141656810 | NA | no | 7.39E-08 | 0.046 | 1.56E-03 | 0.018 |
| ENSG00000257093.3 | *KIAA1147* | chr7:141656727-141702153 | yes | no | 1.82E-11 | 0.125 | 3.85E-07 | 0.040 |
| ENSG00000106028.7 | *SSBP1* | chr7:141738320-141787922 | yes | no | 1.30E-06 | 0.215 | 2.74E-02 | 0.015 |
| ENSG00000155660.7 | *PDIA4* | chr7:149003061-149028641 | yes | no | 4.15E-11 | -0.554 | 8.78E-07 | -0.043 |
| ENSG00000127399.11 | *LRRC61* | chr7:150322638-150338150 | yes | no | 1.15E-07 | 0.133 | 2.44E-03 | 0.032 |
| ENSG00000106560.7 | *GIMAP2* | chr7:150685696-150693641 | yes | yes | 1.07E-06 | 0.121 | 2.26E-02 | 0.030 |
| ENSG00000013374.12 | *NUB1* | chr7:151341698-151378449 | yes | no | 4.27E-09 | 0.336 | 9.03E-05 | 0.041 |
| ENSG00000187260.12 | *WDR86* | chr7:151375908-151410727 | yes | no | 1.19E-06 | 0.077 | 2.51E-02 | 0.039 |
| ENSG00000133627.14 | *ACTR3B* | chr7:152759748-152855378 | yes | yes | 1.00E-06 | 0.091 | 2.12E-02 | 0.046 |
| ENSG00000236408.1 | *RP11-476H24.1* | chr7:154838387-154865483 | NA | no | 3.54E-07 | 0.081 | 7.49E-03 | 0.324 |
| ENSG00000154328.12 | *NEIL2* | chr8:11769638-11787346 | yes | no | 5.68E-07 | 0.094 | 1.20E-02 | 0.034 |
| ENSG00000104763.14 | *ASAH1* | chr8:18056424-18084985 | yes | no | 1.97E-06 | 0.166 | 4.17E-02 | 0.024 |
| ENSG00000120889.9 | *TNFRSF10B* | chr8:23020132-23069179 | no | yes | 3.53E-07 | 0.228 | 7.47E-03 | 0.032 |
| ENSG00000173530.5 | *TNFRSF10D* | chr8:23135587-23164030 | yes | yes | 1.75E-08 | -0.072 | 3.70E-04 | -0.091 |
| ENSG00000147457.10 | *CHMP7* | chr8:23243636-23262000 | yes | no | 7.94E-08 | -0.118 | 1.68E-03 | -0.026 |
| ENSG00000197217.9 | *ENTPD4* | chr8:23385782-23457695 | yes | no | 2.93E-08 | -0.224 | 6.21E-04 | -0.047 |
| ENSG00000104765.11 | *BNIP3L* | chr8:26382897-26505636 | yes | no | 7.70E-08 | -0.124 | 1.63E-03 | -0.030 |
| ENSG00000189233.8 | *NUGGC* | chr8:28021963-28083871 | NA | no | 5.36E-07 | -0.155 | 1.13E-02 | -0.112 |
| ENSG00000168081.5 | *PNOC* | chr8:28316985-28343355 | yes | yes | 1.75E-09 | -0.264 | 3.70E-05 | -0.036 |
| ENSG00000157110.12 | *RBPMS* | chr8:30384478-30572261 | yes | no | 5.31E-10 | 0.452 | 1.12E-05 | 0.210 |
| ENSG00000133874.1 | *RNF122* | chr8:33547754-33567125 | yes | no | 4.37E-16 | -0.152 | 9.25E-12 | -0.067 |
| ENSG00000147535.13 | *PPAPDC1B* | chr8:38263129-38269243 | yes | no | 1.44E-06 | -0.214 | 3.04E-02 | -0.038 |
| ENSG00000147526.16 | *TACC1* | chr8:38728185-38853028 | yes | no | 1.88E-08 | 0.336 | 3.98E-04 | 0.037 |
| ENSG00000029534.16 | *ANK1* | chr8:41653219-41896762 | yes | yes | 4.07E-08 | 0.265 | 8.60E-04 | 0.154 |
| ENSG00000221869.4 | *CEBPD* | chr8:47736908-47739086 | yes | yes | 6.48E-07 | 0.126 | 1.37E-02 | 0.110 |
| ENSG00000168300.10 | *PCMTD1* | chr8:51817574-51899186 | yes | no | 2.68E-07 | -0.111 | 5.67E-03 | -0.037 |
| ENSG00000180828.2 | *BHLHE22* | chr8:64580366-64583628 | yes | no | 1.76E-09 | 0.526 | 3.72E-05 | 0.130 |
| ENSG00000172817.3 | *CYP7B1* | chr8:64587762-64798761 | yes | yes | 1.64E-07 | 0.351 | 3.47E-03 | 0.123 |
| ENSG00000067167.4 | *TRAM1* | chr8:70573441-70608387 | yes | yes | 1.82E-07 | -0.363 | 3.86E-03 | -0.036 |
| ENSG00000178860.8 | *MSC* | chr8:71841548-71844468 | yes | no | 8.25E-13 | 0.639 | 1.74E-08 | 0.056 |
| ENSG00000164751.11 | *PEX2* | chr8:76980257-77001044 | NA | no | 3.65E-07 | -0.222 | 7.71E-03 | -0.026 |
| ENSG00000104432.9 | *IL7* | chr8:78675742-78805523 | no | yes | 7.91E-12 | 0.237 | 1.67E-07 | 0.109 |
| ENSG00000261618.1 | *RP11-79H23.3* | chr8:78837528-78840522 | NA | no | 1.01E-11 | 0.076 | 2.14E-07 | 0.143 |
| ENSG00000164683.13 | *HEY1* | chr8:79764009-79767863 | yes | no | 6.92E-08 | 0.299 | 1.46E-03 | 0.134 |
| ENSG00000076554.12 | *TPD52* | chr8:80034867-80231232 | yes | yes | 6.46E-09 | -0.370 | 1.37E-04 | -0.032 |
| ENSG00000104312.7 | *RIPK2* | chr8:89757746-89791063 | yes | yes | 2.59E-11 | 0.106 | 5.48E-07 | 0.034 |
| ENSG00000104320.10 | *NBN* | chr8:89933335-90003228 | yes | yes | 2.56E-09 | 0.221 | 5.40E-05 | 0.036 |
| ENSG00000253250.2 | *C8orf88* | chr8:90958636-90985257 | NA | no | 5.08E-07 | 0.229 | 1.07E-02 | 0.078 |
| ENSG00000164938.10 | *TP53INP1* | chr8:94925971-94949411 | yes | yes | 2.57E-08 | -0.160 | 5.43E-04 | -0.063 |
| ENSG00000156469.5 | *MTERFD1* | chr8:96239397-96261610 | NA | no | 2.30E-07 | 0.104 | 4.87E-03 | 0.023 |
| ENSG00000147649.6 | *MTDH* | chr8:97644178-97728770 | yes | no | 2.65E-10 | -0.636 | 5.60E-06 | -0.036 |
| ENSG00000104356.7 | *POP1* | chr8:98117296-98159834 | yes | no | 3.85E-07 | 0.151 | 8.14E-03 | 0.033 |
| ENSG00000104450.9 | *SPAG1* | chr8:100157905-100259278 | yes | no | 2.95E-07 | 0.119 | 6.24E-03 | 0.045 |
| ENSG00000120526.7 | *NUDCD1* | chr8:109240918-109334385 | yes | yes | 4.38E-11 | 0.103 | 9.27E-07 | 0.023 |
| ENSG00000164761.5 | *TNFRSF11B* | chr8:118923556-118952200 | no | yes | 3.12E-11 | 0.274 | 6.60E-07 | 0.267 |
| ENSG00000136960.9 | *ENPP2* | chr8:119557085-119673453 | yes | yes | 1.10E-06 | 0.187 | 2.32E-02 | 0.143 |
| ENSG00000173334.3 | *TRIB1* | chr8:125430320-125438405 | yes | yes | 9.73E-07 | -0.259 | 2.06E-02 | -0.060 |
| ENSG00000104419.11 | *NDRG1* | chr8:133237170-133302022 | yes | yes | 1.66E-08 | -0.192 | 3.50E-04 | -0.125 |
| ENSG00000008513.11 | *ST3GAL1* | chr8:133454847-133571940 | yes | no | 1.09E-08 | -0.156 | 2.30E-04 | -0.058 |
| ENSG00000160932.7 | *LY6E* | chr8:143017981-143023832 | yes | no | 2.12E-08 | 0.631 | 4.49E-04 | 0.046 |
| ENSG00000178685.10 | *PARP10* | chr8:143977152-144012772 | yes | no | 5.03E-11 | 0.305 | 1.06E-06 | 0.043 |
| ENSG00000178719.13 | *GRINA* | chr8:143990057-143993415 | yes | no | 4.09E-09 | 0.321 | 8.64E-05 | 0.028 |
| ENSG00000186583.8 | *SPATC1* | chr8:144012413-144047085 | yes | no | 1.98E-09 | 0.086 | 4.18E-05 | 0.183 |
| ENSG00000204791.5 | *CTD-3065J16.6* | chr8:144049128-144051522 | NA | no | 2.90E-08 | 0.073 | 6.14E-04 | 0.240 |
| ENSG00000080608.9 | *KIAA0020* | chr9:2720468-2844241 | yes | no | 9.69E-07 | 0.146 | 2.05E-02 | 0.023 |
| ENSG00000120217.10 | *CD274* | chr9:5450502-5470566 | no | yes | 6.47E-15 | 0.374 | 1.37E-10 | 0.121 |
| ENSG00000197646.7 | *PDCD1LG2* | chr9:5510569-5571254 | no | yes | 3.22E-13 | 0.152 | 6.81E-09 | 0.135 |
| ENSG00000164975.12 | *SNAPC3* | chr9:15422703-15465953 | yes | no | 1.17E-07 | -0.121 | 2.48E-03 | -0.027 |
| ENSG00000164985.11 | *PSIP1* | chr9:15464065-15511019 | yes | no | 3.89E-07 | 0.260 | 8.22E-03 | 0.027 |
| ENSG00000198642.6 | *KLHL9* | chr9:21329670-21335380 | yes | yes | 3.90E-08 | 0.077 | 8.25E-04 | 0.026 |
| ENSG00000188379.6 | *IFNA2* | chr9:21384253-21385388 | no | yes | 7.06E-16 | 0.209 | 1.49E-11 | 0.392 |
| ENSG00000107201.6 | *DDX58* | chr9:32455704-32526324 | yes | yes | 8.90E-14 | 0.172 | 1.88E-09 | 0.074 |
| ENSG00000086061.12 | *DNAJA1* | chr9:33025210-33039907 | yes | no | 9.97E-21 | 0.479 | 2.11E-16 | 0.039 |
| ENSG00000086065.10 | *CHMP5* | chr9:33264878-33282069 | yes | no | 2.20E-11 | 0.182 | 4.66E-07 | 0.028 |
| ENSG00000164967.6 | *RPP25L* | chr9:34610485-34612104 | NA | no | 9.39E-08 | -0.138 | 1.99E-03 | -0.037 |
| ENSG00000137100.12 | *DCTN3* | chr9:34613544-34620523 | yes | no | 1.27E-08 | -0.146 | 2.68E-04 | -0.019 |
| ENSG00000107175.7 | *CREB3* | chr9:35732319-35737007 | yes | no | 3.64E-09 | -0.119 | 7.69E-05 | -0.023 |
| ENSG00000137106.14 | *GRHPR* | chr9:37422665-37436990 | yes | no | 1.27E-13 | 0.691 | 2.68E-09 | 0.062 |
| ENSG00000119139.13 | *TJP2* | chr9:69121263-69255208 | yes | no | 4.38E-09 | 0.226 | 9.25E-05 | 0.054 |
| ENSG00000107372.9 | *ZFAND5* | chr9:72351424-72365235 | yes | no | 1.27E-06 | 0.134 | 2.68E-02 | 0.020 |
| ENSG00000148053.12 | *NTRK2* | chr9:84668550-85027070 | yes | yes | 1.27E-11 | 0.213 | 2.68E-07 | 0.159 |
| ENSG00000157303.7 | *SUSD3* | chr9:93058687-93085138 | yes | no | 1.67E-08 | 0.229 | 3.52E-04 | 0.046 |
| ENSG00000131669.6 | *NINJ1* | chr9:93121488-93134288 | yes | yes | 1.62E-07 | 0.174 | 3.42E-03 | 0.049 |
| ENSG00000196116.7 | *TDRD7* | chr9:97411949-97496125 | yes | yes | 2.63E-08 | 0.090 | 5.55E-04 | 0.030 |
| ENSG00000023318.7 | *ERP44* | chr9:99979178-100099040 | yes | no | 2.29E-11 | -0.115 | 4.85E-07 | -0.024 |
| ENSG00000136891.10 | *TEX10* | chr9:100302076-100352939 | yes | no | 8.65E-08 | 0.088 | 1.83E-03 | 0.020 |
| ENSG00000106701.8 | *FSD1L* | chr9:105447795-105552433 | yes | no | 1.35E-11 | 0.238 | 2.85E-07 | 0.073 |
| ENSG00000136810.9 | *TXN* | chr9:110243810-110256640 | NA | yes | 1.09E-08 | 0.446 | 2.30E-04 | 0.026 |
| ENSG00000136888.6 | *ATP6V1G1* | chr9:114587745-114598373 | yes | no | 4.49E-07 | -0.132 | 9.50E-03 | -0.021 |
| ENSG00000181634.7 | *TNFSF15* | chr9:114784634-114806126 | no | yes | 1.07E-07 | 0.049 | 2.26E-03 | 0.213 |
| ENSG00000136869.13 | *TLR4* | chr9:117704331-117716871 | yes | yes | 1.19E-07 | 0.093 | 2.52E-03 | 0.121 |
| ENSG00000119403.10 | *PHF19* | chr9:120855651-120894896 | yes | no | 1.77E-07 | -0.231 | 3.73E-03 | -0.024 |
| ENSG00000056558.7 | *TRAF1* | chr9:120902392-120929173 | no | yes | 2.53E-13 | 0.468 | 5.34E-09 | 0.067 |
| ENSG00000236901.4 | *MIR600HG* | chr9:123109493-123115477 | NA | no | 9.86E-13 | -0.132 | 2.08E-08 | -0.069 |
| ENSG00000165209.15 | *STRBP* | chr9:123109499-123268576 | yes | no | 2.61E-09 | -0.217 | 5.53E-05 | -0.029 |
| ENSG00000119487.13 | *MAPKAP1* | chr9:125437392-125707234 | yes | no | 5.70E-08 | 0.271 | 1.20E-03 | 0.019 |
| ENSG00000095370.16 | *SH2D3C* | chr9:127738316-127778741 | yes | yes | 4.08E-07 | -0.110 | 8.62E-03 | -0.027 |
| ENSG00000125485.14 | *DDX31* | chr9:132592996-132670401 | yes | no | 2.25E-12 | 0.079 | 4.75E-08 | 0.024 |
| ENSG00000125484.8 | *GTF3C4* | chr9:132670034-132694955 | yes | no | 2.84E-11 | 0.060 | 6.00E-07 | 0.023 |
| ENSG00000148248.10 | *SURF4* | chr9:133361448-133376166 | NA | yes | 1.42E-09 | -0.321 | 3.01E-05 | -0.030 |
| ENSG00000238227.4 | *C9orf69* | chr9:136114580-136118863 | NA | no | 4.19E-07 | 0.076 | 8.86E-03 | 0.021 |
| ENSG00000107937.15 | *GTPBP4* | chr10:988018-1019936 | yes | yes | 1.83E-06 | 0.182 | 3.88E-02 | 0.018 |
| ENSG00000047056.11 | *WDR37* | chr10:1049537-1132297 | yes | no | 8.50E-08 | -0.047 | 1.80E-03 | -0.020 |
| ENSG00000134452.16 | *FBXO18* | chr10:5890202-5937594 | yes | no | 1.65E-06 | -0.168 | 3.49E-02 | -0.023 |
| ENSG00000148484.14 | *RSU1* | chr10:16590610-16817528 | yes | no | 1.29E-07 | 0.157 | 2.73E-03 | 0.022 |
| ENSG00000165996.10 | *PTPLA* | chr10:17589031-17617377 | yes | no | 3.11E-08 | 0.201 | 6.57E-04 | 0.160 |
| ENSG00000185875.9 | *THNSL1* | chr10:25016657-25026664 | yes | no | 1.97E-06 | 0.068 | 4.17E-02 | 0.043 |
| ENSG00000107551.17 | *RASSF4* | chr10:44959406-44995891 | yes | no | 1.29E-07 | 0.332 | 2.73E-03 | 0.086 |
| ENSG00000128815.14 | *WDFY4* | chr10:48684875-48982956 | no | no | 1.36E-17 | 0.320 | 2.88E-13 | 0.065 |
| ENSG00000241577.1 | *RP11-523O18.7* | chr10:48883954-48935213 | NA | no | 1.83E-09 | 0.030 | 3.86E-05 | 0.082 |
| ENSG00000233665.5 | *RP11-523O18.5* | chr10:48976553-48993046 | NA | no | 1.33E-13 | 0.145 | 2.82E-09 | 0.064 |
| ENSG00000177613.8 | *CSTF2T* | chr10:51695486-51699591 | yes | no | 3.04E-09 | 0.050 | 6.42E-05 | 0.014 |
| ENSG00000122873.8 | *CISD1* | chr10:58269057-58289586 | yes | no | 9.47E-12 | 0.201 | 2.00E-07 | 0.032 |
| ENSG00000072401.11 | *UBE2D1* | chr10:58334974-58370753 | yes | no | 9.45E-08 | 0.098 | 2.00E-03 | 0.027 |
| ENSG00000165449.8 | *SLC16A9* | chr10:59650760-59736002 | yes | no | 3.89E-07 | 0.092 | 8.23E-03 | 0.082 |
| ENSG00000108091.10 | *CCDC6* | chr10:59788762-59906656 | yes | no | 1.25E-06 | 0.095 | 2.64E-02 | 0.027 |
| ENSG00000150347.11 | *ARID5B* | chr10:61901299-62096944 | NA | yes | 1.50E-08 | -0.122 | 3.18E-04 | -0.053 |
| ENSG00000165732.9 | *DDX21* | chr10:68956127-68985073 | yes | no | 2.23E-08 | 0.192 | 4.71E-04 | 0.022 |
| ENSG00000122862.4 | *SRGN* | chr10:69088105-69104811 | yes | no | 9.84E-09 | 1.055 | 2.08E-04 | 0.056 |
| ENSG00000042286.11 | *AIFM2* | chr10:70098222-70132934 | no | no | 1.19E-06 | 0.107 | 2.51E-02 | 0.024 |
| ENSG00000180817.8 | *PPA1* | chr10:70202829-70233911 | yes | no | 1.95E-06 | 0.236 | 4.12E-02 | 0.018 |
| ENSG00000107738.16 | *C10orf54* | chr10:71747558-71773498 | yes | no | 3.98E-07 | -0.209 | 8.42E-03 | -0.056 |
| ENSG00000138279.12 | *ANXA7* | chr10:73375100-73414076 | yes | yes | 1.03E-14 | 0.226 | 2.17E-10 | 0.023 |
| ENSG00000176986.11 | *SEC24C* | chr10:73744383-73772161 | yes | yes | 1.24E-08 | -0.116 | 2.61E-04 | -0.015 |
| ENSG00000222047.5 | *C10orf55* | chr10:73909968-73922777 | no | no | 1.41E-09 | 0.031 | 2.97E-05 | 0.131 |
| ENSG00000108219.11 | *TSPAN14* | chr10:80454165-80533123 | yes | no | 1.00E-06 | -0.106 | 2.12E-02 | -0.031 |
| ENSG00000273413.1 | *RP11-96C23.15* | chr10:86970236-86970826 | NA | no | 8.55E-08 | 0.136 | 1.81E-03 | 0.108 |
| ENSG00000152766.5 | *ANKRD22* | chr10:88822131-88851818 | no | no | 7.10E-08 | 0.107 | 1.50E-03 | 0.207 |
| ENSG00000026103.16 | *FAS* | chr10:88990530-89015785 | yes | yes | 6.08E-15 | 0.367 | 1.29E-10 | 0.053 |
| ENSG00000261438.1 | *RP11-399O19.9* | chr10:89015835-89017059 | NA | no | 1.29E-11 | 0.088 | 2.73E-07 | 0.079 |
| ENSG00000119922.8 | *IFIT2* | chr10:89301954-89309276 | yes | yes | 8.42E-14 | 0.273 | 1.78E-09 | 0.119 |
| ENSG00000119917.10 | *IFIT3* | chr10:89327893-89340971 | yes | yes | 4.85E-16 | 0.775 | 1.02E-11 | 0.143 |
| ENSG00000185745.9 | *IFIT1* | chr10:89392545-89406486 | yes | no | 7.77E-11 | 0.460 | 1.64E-06 | 0.114 |
| ENSG00000152778.8 | *IFIT5* | chr10:89414585-89421001 | yes | no | 1.58E-12 | 0.186 | 3.34E-08 | 0.075 |
| ENSG00000180628.11 | *PCGF5* | chr10:91163011-91284331 | yes | no | 3.35E-08 | 0.127 | 7.09E-04 | 0.022 |
| ENSG00000107864.11 | *CPEB3* | chr10:92046691-92291087 | yes | no | 1.98E-07 | -0.053 | 4.18E-03 | -0.062 |
| ENSG00000173145.8 | *NOC3L* | chr10:94333225-94362959 | yes | yes | 2.09E-13 | 0.140 | 4.42E-09 | 0.031 |
| ENSG00000235823.1 | *LINC00263* | chr10:100373614-100383368 | NA | no | 2.70E-07 | -0.045 | 5.70E-03 | -0.052 |
| ENSG00000148840.7 | *PPRC1* | chr10:102132993-102150331 | yes | no | 3.70E-08 | 0.108 | 7.83E-04 | 0.022 |
| ENSG00000077150.14 | *NFKB2* | chr10:102394109-102402529 | yes | yes | 5.66E-16 | 0.493 | 1.20E-11 | 0.064 |
| ENSG00000171206.10 | *TRIM8* | chr10:102644495-102658407 | yes | no | 1.15E-07 | -0.145 | 2.44E-03 | -0.024 |
| ENSG00000138175.8 | *ARL3* | chr10:102673730-102714407 | yes | yes | 2.99E-11 | 0.080 | 6.31E-07 | 0.031 |
| ENSG00000165806.16 | *CASP7* | chr10:113679161-113730907 | yes | yes | 2.94E-07 | 0.127 | 6.21E-03 | 0.025 |
| ENSG00000068383.15 | *INPP5A* | chr10:132537819-132783480 | yes | no | 9.77E-08 | 0.178 | 2.07E-03 | 0.065 |
| ENSG00000151651.12 | *ADAM8* | chr10:133262402-133276868 | yes | yes | 2.24E-07 | 0.267 | 4.74E-03 | 0.040 |
| ENSG00000185885.12 | *IFITM1* | chr11:313505-315272 | yes | yes | 5.59E-07 | 0.631 | 1.18E-02 | 0.045 |
| ENSG00000185507.16 | *IRF7* | chr11:612552-615999 | yes | yes | 1.85E-06 | 0.264 | 3.91E-02 | 0.034 |
| ENSG00000110628.10 | *SLC22A18* | chr11:2899720-2925246 | yes | no | 8.76E-07 | -0.113 | 1.85E-02 | -0.057 |
| ENSG00000177105.9 | *RHOG* | chr11:3826977-3840983 | yes | yes | 9.45E-15 | 0.293 | 2.00E-10 | 0.032 |
| ENSG00000132109.9 | *TRIM21* | chr11:4384896-4393696 | no | yes | 5.39E-16 | 0.216 | 1.14E-11 | 0.042 |
| ENSG00000132274.12 | *TRIM22* | chr11:5689688-5737089 | yes | yes | 3.98E-15 | 0.782 | 8.41E-11 | 0.060 |
| ENSG00000166311.6 | *SMPD1* | chr11:6390430-6394998 | yes | yes | 2.71E-07 | -0.148 | 5.74E-03 | -0.039 |
| ENSG00000166333.10 | *ILK* | chr11:6603707-6610874 | yes | yes | 1.11E-06 | 0.116 | 2.34E-02 | 0.019 |
| ENSG00000166471.7 | *TMEM41B* | chr11:9280653-9314780 | NA | no | 3.78E-07 | -0.087 | 8.00E-03 | -0.028 |
| ENSG00000148926.6 | *ADM* | chr11:10304679-10307397 | yes | yes | 5.37E-07 | -0.254 | 1.14E-02 | -0.179 |
| ENSG00000129084.14 | *PSMA1* | chr11:14504873-14643635 | yes | yes | 5.90E-08 | 0.277 | 1.25E-03 | 0.019 |
| ENSG00000135378.3 | *PRRG4* | chr11:32829942-32858123 | no | no | 5.90E-10 | 0.108 | 1.25E-05 | 0.087 |
| ENSG00000166016.5 | *ABTB2* | chr11:34150987-34358008 | yes | no | 1.71E-06 | 0.118 | 3.62E-02 | 0.079 |
| ENSG00000179431.6 | *FJX1* | chr11:35618418-35620868 | yes | no | 4.06E-10 | 0.163 | 8.59E-06 | 0.075 |
| ENSG00000085117.8 | *CD82* | chr11:44564426-44620363 | yes | no | 1.76E-18 | 1.294 | 3.73E-14 | 0.099 |
| ENSG00000254693.1 | *RP11-58K22.5* | chr11:44604507-44605337 | NA | no | 9.49E-19 | 0.451 | 2.01E-14 | 0.108 |
| ENSG00000025434.15 | *NR1H3* | chr11:47248299-47269032 | yes | yes | 1.98E-06 | -0.091 | 4.18E-02 | -0.037 |
| ENSG00000156587.12 | *UBE2L6* | chr11:57551655-57568284 | yes | yes | 2.93E-19 | 0.669 | 6.20E-15 | 0.069 |
| ENSG00000110048.8 | *OSBP* | chr11:59574397-59616144 | yes | no | 7.78E-10 | -0.107 | 1.65E-05 | -0.020 |
| ENSG00000166889.13 | *PATL1* | chr11:59636715-59668980 | yes | no | 5.18E-08 | 0.091 | 1.09E-03 | 0.019 |
| ENSG00000256813.1 | *RP11-804A23.4* | chr11:60841805-60851081 | NA | no | 4.44E-07 | 0.109 | 9.38E-03 | 0.044 |
| ENSG00000149476.11 | *DAK* | chr11:61333209-61353295 | yes | yes | 6.46E-07 | -0.146 | 1.37E-02 | -0.020 |
| ENSG00000255126.1 | *CTD-2531D15.5* | chr11:62391515-62393372 | NA | no | 3.86E-07 | 0.196 | 8.16E-03 | 0.119 |
| ENSG00000089597.13 | *GANAB* | chr11:62624825-62646726 | yes | no | 1.91E-06 | -0.237 | 4.04E-02 | -0.021 |
| ENSG00000133321.7 | *RARRES3* | chr11:63536808-63546462 | yes | yes | 9.83E-07 | 0.205 | 2.08E-02 | 0.063 |
| ENSG00000162298.13 | *SYVN1* | chr11:65121779-65134533 | yes | yes | 4.15E-09 | -0.354 | 8.78E-05 | -0.037 |
| ENSG00000149798.4 | *CDC42EP2* | chr11:65314817-65322429 | yes | no | 9.19E-07 | 0.067 | 1.94E-02 | 0.093 |
| ENSG00000175550.4 | *DRAP1* | chr11:65919256-65921561 | yes | no | 2.58E-07 | 0.253 | 5.45E-03 | 0.022 |
| ENSG00000174684.6 | *B3GNT1* | chr11:66345371-66347692 | yes | yes | 8.27E-08 | -0.051 | 1.75E-03 | -0.031 |
| ENSG00000175463.8 | *TBC1D10C* | chr11:67403914-67410089 | NA | no | 7.24E-09 | -0.218 | 1.53E-04 | -0.033 |
| ENSG00000175634.11 | *RPS6KB2* | chr11:67428459-67435408 | yes | no | 2.02E-08 | -0.158 | 4.27E-04 | -0.021 |
| ENSG00000162105.13 | *SHANK2* | chr11:70467855-71252577 | yes | no | 7.65E-09 | 0.165 | 1.62E-04 | 0.172 |
| ENSG00000214530.4 | *STARD10* | chr11:72754728-72793681 | yes | no | 1.90E-09 | 0.276 | 4.01E-05 | 0.064 |
| ENSG00000256928.1 | *RP11-809N8.2* | chr11:73395558-73396436 | NA | no | 1.51E-07 | 0.085 | 3.19E-03 | 0.022 |
| ENSG00000175567.5 | *UCP2* | chr11:73974666-73983307 | yes | yes | 6.77E-09 | -0.476 | 1.43E-04 | -0.032 |
| ENSG00000214517.5 | *PPME1* | chr11:74171098-74254703 | yes | no | 4.13E-07 | 0.129 | 8.73E-03 | 0.031 |
| ENSG00000118363.8 | *SPCS2* | chr11:74949246-74979031 | NA | no | 1.32E-08 | -0.584 | 2.78E-04 | -0.031 |
| ENSG00000137491.11 | *SLCO2B1* | chr11:75100562-75206549 | yes | no | 7.56E-08 | 0.302 | 1.60E-03 | 0.130 |
| ENSG00000085741.9 | *WNT11* | chr11:76186324-76210736 | no | no | 1.09E-08 | 0.111 | 2.31E-04 | 0.221 |
| ENSG00000074266.14 | *EED* | chr11:86244543-86278813 | yes | no | 7.89E-10 | 0.156 | 1.67E-05 | 0.022 |
| ENSG00000255241.1 | *RP11-164N3.3* | chr11:88098590-88115835 | NA | no | 2.25E-06 | 0.020 | 4.76E-02 | 0.146 |
| ENSG00000123892.8 | *RAB38* | chr11:88113241-88175467 | no | yes | 2.23E-08 | 0.292 | 4.72E-04 | 0.136 |
| ENSG00000109861.12 | *CTSC* | chr11:88293591-88337787 | yes | yes | 3.45E-07 | 0.859 | 7.30E-03 | 0.053 |
| ENSG00000134627.8 | *PIWIL4* | chr11:94543839-94621421 | yes | no | 1.16E-07 | 0.064 | 2.45E-03 | 0.146 |
| ENSG00000255929.2 | *RP11-867G2.8* | chr11:94545329-94740355 | NA | no | 2.68E-07 | 0.181 | 5.66E-03 | 0.082 |
| ENSG00000149218.4 | *ENDOD1* | chr11:95089809-95132645 | yes | no | 1.38E-10 | 0.195 | 2.93E-06 | 0.059 |
| ENSG00000087053.15 | *MTMR2* | chr11:95832881-95925315 | yes | no | 1.55E-06 | 0.082 | 3.27E-02 | 0.019 |
| ENSG00000184384.10 | *MAML2* | chr11:95976597-96343180 | yes | no | 6.72E-10 | 0.125 | 1.42E-05 | 0.069 |
| ENSG00000023445.10 | *BIRC3* | chr11:102317449-102339403 | no | yes | 1.02E-17 | 0.518 | 2.16E-13 | 0.074 |
| ENSG00000152558.11 | *TMEM123* | chr11:102396331-102470384 | yes | no | 5.28E-07 | -0.343 | 1.12E-02 | -0.035 |
| ENSG00000137673.5 | *MMP7* | chr11:102520507-102530753 | no | yes | 4.18E-10 | 0.569 | 8.85E-06 | 0.252 |
| ENSG00000137752.19 | *CASP1* | chr11:105025442-105035250 | no | yes | 2.96E-07 | 0.371 | 6.26E-03 | 0.086 |
| ENSG00000110777.8 | *POU2AF1* | chr11:111352251-111455630 | no | yes | 7.26E-07 | -0.412 | 1.54E-02 | -0.026 |
| ENSG00000109846.4 | *CRYAB* | chr11:111908564-111923722 | NA | yes | 1.22E-06 | 0.166 | 2.58E-02 | 0.209 |
| ENSG00000095139.10 | *ARCN1* | chr11:118572389-118603033 | yes | no | 3.44E-08 | -0.144 | 7.28E-04 | -0.014 |
| ENSG00000172269.13 | *DPAGT1* | chr11:119096502-119108331 | yes | no | 4.48E-09 | -0.178 | 9.48E-05 | -0.028 |
| ENSG00000172375.9 | *C2CD2L* | chr11:119102197-119118544 | yes | no | 1.15E-08 | -0.094 | 2.43E-04 | -0.029 |
| ENSG00000110400.7 | *PVRL1* | chr11:119623407-119729084 | yes | yes | 3.11E-09 | 0.201 | 6.57E-05 | 0.077 |
| ENSG00000109971.10 | *HSPA8* | chr11:123057488-123063230 | yes | no | 1.84E-06 | 0.738 | 3.89E-02 | 0.017 |
| ENSG00000134910.9 | *STT3A* | chr11:125591711-125625215 | yes | yes | 5.09E-09 | -0.496 | 1.08E-04 | -0.036 |
| ENSG00000182934.8 | *SRPR* | chr11:126262918-126269144 | yes | no | 3.82E-11 | -0.267 | 8.09E-07 | -0.027 |
| ENSG00000151502.7 | *VPS26B* | chr11:134224644-134247792 | yes | no | 3.86E-13 | -0.123 | 8.16E-09 | -0.033 |
| ENSG00000171823.6 | *FBXL14* | chr12:1565992-1594165 | yes | no | 1.59E-06 | 0.097 | 3.37E-02 | 0.039 |
| ENSG00000272173.1 | *RNU7-1* | chr12:6943507-6944604 | NA | no | 2.17E-06 | -0.134 | 4.59E-02 | -0.024 |
| ENSG00000111732.7 | *AICDA* | chr12:8602165-8612871 | no | yes | 1.64E-08 | -0.570 | 3.47E-04 | -0.071 |
| ENSG00000110848.5 | *CD69* | chr12:9752485-9760901 | no | yes | 1.21E-10 | 0.261 | 2.56E-06 | 0.094 |
| ENSG00000245648.1 | *RP11-277P12.20* | chr12:10363768-10398506 | NA | no | 2.81E-07 | 0.069 | 5.95E-03 | 0.176 |
| ENSG00000060138.9 | *YBX3* | chr12:10699088-10723312 | NA | no | 5.44E-08 | 0.473 | 1.15E-03 | 0.040 |
| ENSG00000111266.5 | *DUSP16* | chr12:12474209-12562383 | yes | no | 5.63E-07 | 0.206 | 1.19E-02 | 0.053 |
| ENSG00000111276.7 | *CDKN1B* | chr12:12715057-12722371 | yes | yes | 1.68E-12 | -0.223 | 3.56E-08 | -0.040 |
| ENSG00000213782.4 | *DDX47* | chr12:12813315-12829981 | yes | no | 6.02E-13 | 0.203 | 1.27E-08 | 0.028 |
| ENSG00000023734.7 | *STRAP* | chr12:15882390-15903478 | yes | no | 7.48E-07 | 0.163 | 1.58E-02 | 0.015 |
| ENSG00000226397.4 | *C12orf77* | chr12:24993423-24997519 | NA | no | 2.68E-07 | -0.163 | 5.66E-03 | -0.095 |
| ENSG00000247903.1 | *RP11-421F16.3* | chr12:26971585-26979582 | NA | no | 3.82E-07 | 0.072 | 8.08E-03 | 0.057 |
| ENSG00000029153.11 | *ARNTL2* | chr12:27332853-27425289 | yes | no | 7.46E-07 | 0.207 | 1.58E-02 | 0.042 |
| ENSG00000170456.11 | *DENND5B* | chr12:31382222-31591097 | yes | no | 1.44E-07 | -0.192 | 3.05E-03 | -0.056 |
| ENSG00000139131.9 | *YARS2* | chr12:32727489-32755902 | yes | no | 1.11E-06 | 0.095 | 2.34E-02 | 0.018 |
| ENSG00000061273.14 | *HDAC7* | chr12:47782721-47833132 | yes | yes | 3.06E-12 | -0.287 | 6.46E-08 | -0.086 |
| ENSG00000268069.2 | *RP5-1057I20.4* | chr12:47784922-47786002 | NA | no | 5.98E-09 | -0.104 | 1.26E-04 | -0.089 |
| ENSG00000111424.7 | *VDR* | chr12:47841536-47943048 | no | yes | 9.80E-14 | -0.249 | 2.07E-09 | -0.113 |
| ENSG00000205537.2 | *RP11-89H19.1* | chr12:47882648-47901525 | NA | no | 1.58E-07 | -0.033 | 3.34E-03 | -0.149 |
| ENSG00000134291.8 | *TMEM106C* | chr12:47963568-47968878 | yes | no | 1.24E-06 | -0.260 | 2.61E-02 | -0.022 |
| ENSG00000134285.7 | *FKBP11* | chr12:48921517-48926474 | yes | yes | 1.32E-06 | -0.485 | 2.80E-02 | -0.048 |
| ENSG00000167550.7 | *RHEBL1* | chr12:49064684-49070025 | yes | no | 2.37E-07 | 0.127 | 5.02E-03 | 0.049 |
| ENSG00000161791.10 | *FMNL3* | chr12:49636498-49708165 | yes | no | 1.49E-08 | 0.170 | 3.16E-04 | 0.036 |
| ENSG00000185432.11 | *METTL7A* | chr12:50923471-50932517 | yes | no | 1.89E-06 | -0.241 | 4.00E-02 | -0.076 |
| ENSG00000257671.1 | *RP3-416H24.1* | chr12:52245047-52247448 | NA | no | 8.46E-07 | 0.082 | 1.79E-02 | 0.100 |
| ENSG00000139626.12 | *ITGB7* | chr12:53191317-53207307 | yes | yes | 3.53E-08 | -0.586 | 7.46E-04 | -0.040 |
| ENSG00000012822.12 | *CALCOCO1* | chr12:53708516-53727745 | yes | no | 1.20E-08 | -0.153 | 2.53E-04 | -0.040 |
| ENSG00000139572.3 | *GPR84* | chr12:54362444-54364487 | no | yes | 9.64E-08 | 0.095 | 2.04E-03 | 0.139 |
| ENSG00000135426.11 | *TESPA1* | chr12:54948017-54984746 | NA | no | 1.56E-09 | 0.216 | 3.30E-05 | 0.047 |
| ENSG00000135473.11 | *PAN2* | chr12:56316222-56334053 | yes | no | 9.25E-07 | -0.176 | 1.96E-02 | -0.028 |
| ENSG00000110958.12 | *PTGES3* | chr12:56663340-56688408 | NA | no | 8.67E-09 | 0.267 | 1.83E-04 | 0.017 |
| ENSG00000135446.13 | *CDK4* | chr12:57747726-57756013 | yes | yes | 1.09E-09 | 0.336 | 2.30E-05 | 0.023 |
| ENSG00000175215.6 | *CTDSP2* | chr12:57819926-57846739 | yes | no | 8.05E-07 | -0.169 | 1.70E-02 | -0.021 |
| ENSG00000166226.9 | *CCT2* | chr12:69585333-69601570 | yes | no | 4.32E-09 | 0.273 | 9.13E-05 | 0.020 |
| ENSG00000127328.18 | *RAB3IP* | chr12:69738680-69823204 | yes | no | 7.50E-13 | 0.215 | 1.58E-08 | 0.045 |
| ENSG00000139291.10 | *TMEM19* | chr12:71686086-71705046 | yes | no | 8.86E-10 | -0.236 | 1.87E-05 | -0.036 |
| ENSG00000120800.4 | *UTP20* | chr12:101280108-101386616 | yes | no | 7.59E-07 | 0.057 | 1.60E-02 | 0.020 |
| ENSG00000136010.10 | *ALDH1L2* | chr12:105019783-105084577 | NA | no | 1.78E-06 | -0.184 | 3.77E-02 | -0.083 |
| ENSG00000136026.10 | *CKAP4* | chr12:106237876-106304279 | yes | no | 1.95E-09 | -0.320 | 4.11E-05 | -0.067 |
| ENSG00000151135.6 | *TMEM263* | chr12:106955718-106978778 | NA | no | 2.51E-07 | -0.114 | 5.31E-03 | -0.021 |
| ENSG00000136003.12 | *ISCU* | chr12:108562581-108569384 | yes | no | 1.46E-06 | 0.360 | 3.09E-02 | 0.032 |
| ENSG00000084112.11 | *SSH1* | chr12:108782693-108857590 | yes | no | 3.94E-08 | 0.116 | 8.34E-04 | 0.038 |
| ENSG00000135148.8 | *TRAFD1* | chr12:112125500-112153609 | yes | yes | 1.21E-09 | 0.205 | 2.56E-05 | 0.033 |
| ENSG00000111331.9 | *OAS3* | chr12:112938351-112973249 | yes | yes | 4.54E-09 | 0.540 | 9.59E-05 | 0.098 |
| ENSG00000111335.9 | *OAS2* | chr12:112978394-113011723 | yes | yes | 1.70E-06 | 0.422 | 3.59E-02 | 0.041 |
| ENSG00000135127.8 | *CCDC64* | chr12:119989868-120094494 | yes | no | 1.61E-07 | 0.224 | 3.40E-03 | 0.077 |
| ENSG00000135114.9 | *OASL* | chr12:121020291-121039242 | no | yes | 5.71E-11 | 0.145 | 1.21E-06 | 0.097 |
| ENSG00000089094.13 | *KDM2B* | chr12:121429096-121581015 | yes | no | 1.75E-09 | 0.212 | 3.70E-05 | 0.032 |
| ENSG00000139725.4 | *RHOF* | chr12:121777753-121803403 | NA | no | 5.58E-13 | 0.246 | 1.18E-08 | 0.042 |
| ENSG00000150977.10 | *RILPL2* | chr12:123410682-123436717 | NA | no | 2.60E-12 | 0.146 | 5.51E-08 | 0.038 |
| ENSG00000086598.7 | *TMED2* | chr12:123584530-123598577 | yes | no | 2.14E-08 | -0.365 | 4.52E-04 | -0.028 |
| ENSG00000073060.12 | *SCARB1* | chr12:124776855-124882668 | yes | yes | 8.12E-07 | 0.231 | 1.72E-02 | 0.035 |
| ENSG00000111450.10 | *STX2* | chr12:130789599-130839266 | yes | yes | 7.56E-08 | 0.109 | 1.60E-03 | 0.047 |
| ENSG00000176915.11 | *ANKLE2* | chr12:132725502-132761888 | NA | no | 7.47E-07 | 0.142 | 1.58E-02 | 0.020 |
| ENSG00000121743.3 | *GJA3* | chr13:20138254-20161049 | yes | no | 3.45E-07 | 0.098 | 7.30E-03 | 0.161 |
| ENSG00000165474.5 | *GJB2* | chr13:20187469-20192898 | yes | no | 7.23E-08 | 0.149 | 1.53E-03 | 0.151 |
| ENSG00000102699.5 | *PARP4* | chr13:24420925-24512810 | yes | yes | 2.20E-06 | -0.093 | 4.65E-02 | -0.018 |
| ENSG00000102755.7 | *FLT1* | chr13:28300343-28495128 | yes | yes | 2.31E-06 | 0.122 | 4.87E-02 | 0.102 |
| ENSG00000132963.7 | *POMP* | chr13:28659103-28678925 | yes | yes | 1.97E-11 | 0.267 | 4.17E-07 | 0.026 |
| ENSG00000139597.13 | *N4BP2L1* | chr13:32400722-32428178 | yes | no | 3.71E-07 | 0.098 | 7.84E-03 | 0.030 |
| ENSG00000120688.8 | *WBP4* | chr13:41061273-41084006 | yes | no | 8.14E-08 | 0.064 | 1.72E-03 | 0.019 |
| ENSG00000133106.11 | *EPSTI1* | chr13:42886387-42992271 | yes | no | 2.82E-11 | 0.370 | 5.96E-07 | 0.075 |
| ENSG00000083635.7 | *NUFIP1* | chr13:44939248-44989483 | yes | no | 3.77E-08 | 0.042 | 7.98E-04 | 0.020 |
| ENSG00000188342.8 | *GTF2F2* | chr13:45120514-45284909 | yes | no | 2.43E-07 | 0.071 | 5.15E-03 | 0.019 |
| ENSG00000136152.11 | *COG3* | chr13:45464897-45536630 | yes | no | 9.52E-07 | -0.146 | 2.01E-02 | -0.029 |
| ENSG00000136159.3 | *NUDT15* | chr13:48037566-48047222 | yes | no | 1.09E-06 | 0.064 | 2.30E-02 | 0.019 |
| ENSG00000136147.13 | *PHF11* | chr13:49495609-49528987 | yes | yes | 5.67E-09 | 0.182 | 1.20E-04 | 0.031 |
| ENSG00000118939.14 | *UCHL3* | chr13:75549479-75606020 | yes | yes | 9.74E-07 | 0.128 | 2.06E-02 | 0.021 |
| ENSG00000005810.14 | *MYCBP2* | chr13:77044654-77327050 | yes | no | 3.22E-13 | 0.227 | 6.82E-09 | 0.034 |
| ENSG00000152192.7 | *POU4F1* | chr13:78598361-78603560 | yes | yes | 1.62E-08 | -0.039 | 3.43E-04 | -0.100 |
| ENSG00000152193.7 | *RNF219* | chr13:78614290-78659179 | yes | no | 8.32E-08 | 0.062 | 1.76E-03 | 0.019 |
| ENSG00000179399.10 | *GPC5* | chr13:91398674-92873682 | yes | no | 8.27E-10 | 0.160 | 1.75E-05 | 0.150 |
| ENSG00000102580.11 | *DNAJC3* | chr13:95677138-95794989 | yes | no | 1.55E-07 | -0.180 | 3.28E-03 | -0.037 |
| ENSG00000125304.8 | *TM9SF2* | chr13:99501416-99564006 | yes | yes | 5.92E-07 | -0.137 | 1.25E-02 | -0.019 |
| ENSG00000102524.8 | *TNFSF13B* | chr13:108251239-108308484 | yes | yes | 1.02E-09 | 0.294 | 2.16E-05 | 0.098 |
| ENSG00000165801.6 | *ARHGEF40* | chr14:21070269-21090240 | NA | no | 1.32E-06 | 0.056 | 2.78E-02 | 0.195 |
| ENSG00000251002.4 | *AE000661.37* | chr14:22380679-22482959 | NA | no | 2.11E-07 | 0.127 | 4.47E-03 | 0.090 |
| ENSG00000100804.15 | *PSMB5* | chr14:23016542-23035230 | yes | yes | 7.05E-08 | 0.215 | 1.49E-03 | 0.019 |
| ENSG00000136367.13 | *ZFHX2* | chr14:23520854-23556192 | yes | no | 1.20E-06 | 0.073 | 2.54E-02 | 0.101 |
| ENSG00000092051.13 | *JPH4* | chr14:23568034-23578800 | yes | yes | 1.35E-06 | 0.116 | 2.86E-02 | 0.089 |
| ENSG00000092010.11 | *PSME1* | chr14:24136157-24138967 | yes | yes | 1.91E-17 | 0.610 | 4.03E-13 | 0.036 |
| ENSG00000100911.10 | *PSME2* | chr14:24143361-24147570 | yes | yes | 1.92E-16 | 0.775 | 4.06E-12 | 0.030 |
| ENSG00000213928.5 | *IRF9* | chr14:24161052-24166565 | yes | yes | 2.59E-10 | 0.307 | 5.48E-06 | 0.028 |
| ENSG00000139899.7 | *CBLN3* | chr14:24426531-24430954 | yes | no | 3.01E-07 | 0.104 | 6.36E-03 | 0.072 |
| ENSG00000129515.15 | *SNX6* | chr14:34561093-34630183 | yes | no | 6.28E-09 | 0.132 | 1.33E-04 | 0.020 |
| ENSG00000258738.1 | *RP11-73E17.2* | chr14:34874342-34876459 | NA | no | 3.90E-07 | 0.047 | 8.24E-03 | 0.022 |
| ENSG00000100906.7 | *NFKBIA* | chr14:35401510-35404749 | yes | yes | 4.08E-14 | 0.890 | 8.64E-10 | 0.063 |
| ENSG00000165355.7 | *FBXO33* | chr14:39397668-39432500 | yes | no | 1.00E-08 | -0.126 | 2.12E-04 | -0.032 |
| ENSG00000165527.6 | *ARF6* | chr14:49893091-49897054 | yes | yes | 1.58E-06 | 0.085 | 3.33E-02 | 0.012 |
| ENSG00000186469.5 | *GNG2* | chr14:51826194-51979342 | yes | yes | 8.58E-08 | 0.263 | 1.81E-03 | 0.038 |
| ENSG00000259007.1 | *RP11-463J10.3* | chr14:51967002-51969800 | NA | no | 8.41E-08 | 0.221 | 1.78E-03 | 0.041 |
| ENSG00000087302.5 | *C14orf166* | chr14:51989474-52010691 | yes | no | 8.99E-08 | 0.214 | 1.90E-03 | 0.013 |
| ENSG00000126777.14 | *KTN1* | chr14:55559071-55701526 | yes | no | 1.62E-06 | 0.270 | 3.43E-02 | 0.028 |
| ENSG00000139998.11 | *RAB15* | chr14:64945813-64972776 | yes | yes | 3.97E-07 | 0.110 | 8.40E-03 | 0.089 |
| ENSG00000134001.9 | *EIF2S1* | chr14:67359996-67386516 | yes | yes | 1.58E-07 | 0.220 | 3.35E-03 | 0.015 |
| ENSG00000100632.7 | *ERH* | chr14:69380122-69398627 | yes | no | 4.76E-08 | 0.521 | 1.01E-03 | 0.022 |
| ENSG00000213463.4 | *SYNJ2BP* | chr14:70366495-70417061 | yes | no | 2.59E-07 | 0.095 | 5.47E-03 | 0.042 |
| ENSG00000170348.5 | *TMED10* | chr14:75131469-75176631 | yes | no | 1.15E-07 | -0.298 | 2.44E-03 | -0.025 |
| ENSG00000119669.4 | *IRF2BPL* | chr14:77024542-77028699 | NA | yes | 7.39E-08 | 0.118 | 1.56E-03 | 0.038 |
| ENSG00000100603.10 | *SNW1* | chr14:77717598-77761207 | yes | no | 6.91E-07 | 0.110 | 1.46E-02 | 0.013 |
| ENSG00000053254.12 | *FOXN3* | chr14:89124870-89619149 | yes | no | 2.02E-06 | -0.141 | 4.27E-02 | -0.031 |
| ENSG00000277801.1 | *RP11-681H18.2* | chr14:89156742-89157574 | NA | no | 4.86E-07 | -0.127 | 1.03E-02 | -0.031 |
| ENSG00000165929.9 | *TC2N* | chr14:91779750-91867536 | yes | no | 1.09E-10 | 0.331 | 2.31E-06 | 0.179 |
| ENSG00000100599.12 | *RIN3* | chr14:92513773-92688994 | yes | no | 4.05E-11 | 0.283 | 8.56E-07 | 0.089 |
| ENSG00000185215.5 | *TNFAIP2* | chr14:103123441-103137439 | yes | no | 1.35E-14 | 0.702 | 2.87E-10 | 0.142 |
| ENSG00000166166.9 | *TRMT61A* | chr14:103529183-103537073 | yes | no | 7.60E-08 | 0.074 | 1.61E-03 | 0.026 |
| ENSG00000183484.8 | *GPR132* | chr14:105049388-105065445 | no | yes | 1.11E-11 | 0.192 | 2.35E-07 | 0.045 |
| ENSG00000259448.2 | *RP11-16E12.1* | chr15:31216019-31224445 | NA | no | 5.94E-08 | -0.054 | 1.26E-03 | -0.067 |
| ENSG00000259772.3 | *RP11-16E12.2* | chr15:31221998-31230838 | NA | no | 1.51E-17 | -0.512 | 3.20E-13 | -0.098 |
| ENSG00000021776.7 | *AQR* | chr15:34851781-34969839 | yes | no | 7.88E-07 | 0.075 | 1.67E-02 | 0.016 |
| ENSG00000104140.6 | *RHOV* | chr15:40872213-40874289 | yes | no | 3.78E-09 | 0.160 | 8.00E-05 | 0.105 |
| ENSG00000166946.10 | *CCNDBP1* | chr15:43185117-43197176 | yes | no | 9.53E-07 | -0.097 | 2.02E-02 | -0.015 |
| ENSG00000104164.7 | *BLOC1S6* | chr15:45587122-45615999 | NA | no | 4.62E-08 | -0.124 | 9.78E-04 | -0.023 |
| ENSG00000140280.10 | *LYSMD2* | chr15:51723010-51751585 | yes | no | 2.12E-14 | 0.353 | 4.48E-10 | 0.072 |
| ENSG00000128872.6 | *TMOD2* | chr15:51751560-51816368 | yes | no | 1.82E-08 | 0.117 | 3.84E-04 | 0.072 |
| ENSG00000069956.8 | *MAPK6* | chr15:51952105-52067372 | yes | no | 1.48E-07 | 0.176 | 3.14E-03 | 0.034 |
| ENSG00000047346.9 | *FAM214A* | chr15:52581316-52709817 | NA | no | 4.79E-10 | -0.254 | 1.01E-05 | -0.047 |
| ENSG00000247982.3 | *LINC00926* | chr15:57300364-57307769 | NA | no | 5.91E-12 | 0.266 | 1.25E-07 | 0.081 |
| ENSG00000103569.6 | *AQP9* | chr15:58138168-58185911 | no | yes | 1.89E-07 | 0.111 | 3.99E-03 | 0.163 |
| ENSG00000137845.11 | *ADAM10* | chr15:58588806-58749978 | yes | yes | 1.81E-07 | -0.196 | 3.83E-03 | -0.023 |
| ENSG00000103642.8 | *LACTB* | chr15:63121799-63142061 | yes | yes | 2.47E-08 | 0.116 | 5.22E-04 | 0.053 |
| ENSG00000166794.4 | *PPIB* | chr15:64155811-64163205 | yes | no | 6.61E-08 | -0.975 | 1.40E-03 | -0.033 |
| ENSG00000241839.6 | *PLEKHO2* | chr15:64841882-64868007 | yes | no | 8.66E-07 | 0.078 | 1.83E-02 | 0.020 |
| ENSG00000103769.6 | *RAB11A* | chr15:65726053-65891991 | yes | no | 1.53E-14 | 0.675 | 3.23E-10 | 0.048 |
| ENSG00000140350.12 | *ANP32A* | chr15:68778534-68820897 | yes | no | 8.31E-07 | 0.210 | 1.76E-02 | 0.014 |
| ENSG00000138623.6 | *SEMA7A* | chr15:74409288-74434467 | yes | yes | 1.18E-12 | 0.477 | 2.50E-08 | 0.052 |
| ENSG00000140497.13 | *SCAMP2* | chr15:74843729-74873365 | yes | no | 1.24E-10 | -0.303 | 2.62E-06 | -0.026 |
| ENSG00000178802.14 | *MPI* | chr15:74890004-74902219 | yes | no | 2.42E-10 | -0.198 | 5.12E-06 | -0.033 |
| ENSG00000167196.10 | *FBXO22* | chr15:75903858-75942510 | yes | no | 1.21E-08 | 0.217 | 2.55E-04 | 0.027 |
| ENSG00000117906.10 | *RCN2* | chr15:76931618-76954392 | yes | no | 1.93E-13 | -0.198 | 4.08E-09 | -0.044 |
| ENSG00000103740.6 | *ACSBG1* | chr15:78167467-78245688 | yes | no | 1.81E-09 | 0.076 | 3.82E-05 | 0.021 |
| ENSG00000041357.12 | *PSMA4* | chr15:78540404-78552419 | yes | yes | 1.44E-10 | 0.373 | 3.05E-06 | 0.023 |
| ENSG00000140379.7 | *BCL2A1* | chr15:79960888-79971446 | no | yes | 2.90E-12 | 0.524 | 6.13E-08 | 0.065 |
| ENSG00000086666.15 | *ZFAND6* | chr15:80059567-80138393 | yes | no | 1.17E-10 | -0.424 | 2.47E-06 | -0.052 |
| ENSG00000140612.10 | *SEC11A* | chr15:84669537-84716716 | yes | no | 3.39E-11 | -0.212 | 7.17E-07 | -0.020 |
| ENSG00000140511.8 | *HAPLN3* | chr15:88877287-88895626 | yes | no | 7.67E-15 | 0.241 | 1.62E-10 | 0.232 |
| ENSG00000185033.11 | *SEMA4B* | chr15:90160603-90229679 | yes | yes | 1.95E-06 | -0.187 | 4.13E-02 | -0.053 |
| ENSG00000182768.8 | *NGRN* | chr15:90265658-90278141 | NA | no | 4.79E-08 | 0.075 | 1.01E-03 | 0.021 |
| ENSG00000007520.3 | *TSR3* | chr16:1349239-1351911 | NA | no | 1.60E-06 | 0.149 | 3.38E-02 | 0.021 |
| ENSG00000131650.10 | *KREMEN2* | chr16:2963943-2968383 | yes | no | 9.99E-08 | 0.088 | 2.11E-03 | 0.089 |
| ENSG00000185338.4 | *SOCS1* | chr16:11254404-11256179 | no | yes | 8.98E-10 | 0.267 | 1.90E-05 | 0.078 |
| ENSG00000184602.5 | *SNN* | chr16:11668413-11679159 | yes | no | 4.24E-18 | 0.341 | 8.96E-14 | 0.096 |
| ENSG00000153066.9 | *TXNDC11* | chr16:11679079-11742878 | yes | no | 2.93E-08 | -0.422 | 6.20E-04 | -0.048 |
| ENSG00000262420.3 | *RP11-490O6.2* | chr16:11741909-11744506 | NA | no | 1.60E-10 | -0.092 | 3.38E-06 | -0.051 |
| ENSG00000234719.5 | *RP11-166B2.1* | chr16:11927372-11976643 | NA | no | 8.97E-09 | -0.117 | 1.90E-04 | -0.086 |
| ENSG00000048462.7 | *TNFRSF17* | chr16:11965106-11968068 | no | yes | 2.64E-14 | -0.864 | 5.58E-10 | -0.066 |
| ENSG00000072864.9 | *NDE1* | chr16:15643266-15726353 | yes | no | 8.73E-08 | 0.247 | 1.85E-03 | 0.025 |
| ENSG00000103319.8 | *EEF2K* | chr16:22206281-22288732 | yes | yes | 1.75E-06 | 0.089 | 3.71E-02 | 0.029 |
| ENSG00000197272.2 | *IL27* | chr16:28499361-28512051 | NA | yes | 4.22E-07 | 0.092 | 8.93E-03 | 0.273 |
| ENSG00000260719.1 | *AC009133.17* | chr16:29745246-29748299 | NA | no | 2.34E-06 | -0.104 | 4.94E-02 | -0.081 |
| ENSG00000090238.8 | *YPEL3* | chr16:30092313-30096915 | yes | no | 2.45E-07 | -0.208 | 5.18E-03 | -0.045 |
| ENSG00000005844.14 | *ITGAL* | chr16:30472657-30523185 | no | yes | 3.54E-09 | -0.549 | 7.50E-05 | -0.057 |
| ENSG00000261346.1 | *RP11-297C4.2* | chr16:30477179-30489353 | NA | no | 3.64E-09 | -0.189 | 7.69E-05 | -0.062 |
| ENSG00000261332.1 | *RP11-297C4.1* | chr16:30498765-30499554 | NA | no | 1.78E-08 | -0.213 | 3.76E-04 | -0.057 |
| ENSG00000089280.15 | *FUS* | chr16:31180109-31194871 | yes | yes | 1.00E-08 | 0.337 | 2.12E-04 | 0.017 |
| ENSG00000185947.11 | *ZNF267* | chr16:31873757-31917357 | yes | no | 1.95E-11 | 0.146 | 4.12E-07 | 0.039 |
| ENSG00000069345.8 | *DNAJA2* | chr16:46955361-46973788 | yes | no | 9.11E-10 | 0.107 | 1.93E-05 | 0.015 |
| ENSG00000259283.2 | *RP11-26L20.3* | chr16:55259968-55333588 | NA | no | 8.48E-08 | 0.120 | 1.79E-03 | 0.349 |
| ENSG00000125148.6 | *MT2A* | chr16:56608198-56609497 | NA | yes | 1.23E-09 | 0.698 | 2.60E-05 | 0.116 |
| ENSG00000102900.9 | *NUP93* | chr16:56730104-56850286 | yes | no | 8.48E-08 | 0.213 | 1.79E-03 | 0.027 |
| ENSG00000051108.11 | *HERPUD1* | chr16:56932047-56944863 | yes | no | 7.97E-08 | -0.724 | 1.69E-03 | -0.049 |
| ENSG00000102962.4 | *CCL22* | chr16:57358771-57366190 | no | yes | 1.14E-08 | 1.398 | 2.42E-04 | 0.094 |
| ENSG00000102970.7 | *CCL17* | chr16:57404766-57416062 | yes | yes | 3.78E-09 | 0.837 | 7.98E-05 | 0.161 |
| ENSG00000237172.3 | *B3GNT9* | chr16:67148104-67151214 | yes | no | 3.56E-08 | -0.083 | 7.54E-04 | -0.041 |
| ENSG00000124074.8 | *ENKD1* | chr16:67662944-67667265 | NA | no | 5.61E-07 | 0.150 | 1.19E-02 | 0.058 |
| ENSG00000132603.10 | *NIP7* | chr16:69339429-69343111 | yes | no | 8.75E-07 | 0.159 | 1.85E-02 | 0.020 |
| ENSG00000103018.13 | *CYB5B* | chr16:69424524-69466266 | yes | no | 3.30E-07 | 0.192 | 6.98E-03 | 0.015 |
| ENSG00000141101.9 | *NOB1* | chr16:69741866-69754940 | yes | no | 9.45E-07 | 0.125 | 2.00E-02 | 0.017 |
| ENSG00000090863.8 | *GLG1* | chr16:74451957-74607114 | yes | no | 5.66E-09 | -0.139 | 1.20E-04 | -0.027 |
| ENSG00000065427.11 | *KARS* | chr16:75627473-75648643 | yes | yes | 4.18E-08 | 0.171 | 8.83E-04 | 0.014 |
| ENSG00000230989.3 | *HSBP1* | chr16:83807842-83819737 | yes | no | 1.22E-06 | 0.140 | 2.59E-02 | 0.021 |
| ENSG00000153789.9 | *FAM92B* | chr16:85098357-85112508 | yes | no | 4.86E-07 | -0.098 | 1.03E-02 | -0.183 |
| ENSG00000225614.2 | *ZNF469* | chr16:88427470-88440757 | NA | no | 5.78E-09 | 0.081 | 1.22E-04 | 0.115 |
| ENSG00000003249.10 | *DBNDD1* | chr16:90004864-90020128 | yes | no | 3.79E-08 | 0.321 | 8.02E-04 | 0.154 |
| ENSG00000167740.6 | *CYB5D2* | chr17:4143167-4187310 | yes | no | 1.23E-07 | -0.094 | 2.60E-03 | -0.032 |
| ENSG00000132388.9 | *UBE2G1* | chr17:4269258-4366628 | yes | no | 8.18E-13 | -0.403 | 1.73E-08 | -0.042 |
| ENSG00000108518.7 | *PFN1* | chr17:4945651-4949061 | yes | yes | 1.26E-09 | 0.643 | 2.66E-05 | 0.020 |
| ENSG00000108515.14 | *ENO3* | chr17:4948091-4957131 | no | no | 4.24E-08 | 0.540 | 8.96E-04 | 0.020 |
| ENSG00000129250.8 | *KIF1C* | chr17:4997947-5028401 | yes | yes | 3.06E-07 | 0.160 | 6.46E-03 | 0.026 |
| ENSG00000132530.13 | *XAF1* | chr17:6755446-6775647 | yes | yes | 2.94E-13 | 0.545 | 6.22E-09 | 0.102 |
| ENSG00000258315.2 | *C17orf49* | chr17:7014494-7017525 | yes | no | 7.64E-09 | 0.271 | 1.62E-04 | 0.033 |
| ENSG00000267532.3 | *MIR497HG* | chr17:7015817-7019659 | NA | no | 3.23E-08 | 0.087 | 6.83E-04 | 0.029 |
| ENSG00000132507.14 | *EIF5A* | chr17:7306998-7312463 | yes | no | 2.33E-09 | 0.452 | 4.92E-05 | 0.020 |
| ENSG00000132522.12 | *GPS2* | chr17:7311323-7315564 | NA | no | 1.83E-06 | 0.131 | 3.88E-02 | 0.012 |
| ENSG00000196544.7 | *C17orf59* | chr17:8188332-8190907 | yes | no | 7.69E-07 | -0.057 | 1.63E-02 | -0.020 |
| ENSG00000178977.3 | *LINC00324* | chr17:8220641-8224043 | NA | no | 2.85E-08 | -0.066 | 6.02E-04 | -0.064 |
| ENSG00000109016.14 | *DHRS7B* | chr17:21123363-21193265 | yes | no | 4.78E-07 | 0.094 | 1.01E-02 | 0.026 |
| ENSG00000274180.1 | *NATD1* | chr17:21238869-21253410 | NA | no | 1.34E-07 | -0.042 | 2.84E-03 | -0.062 |
| ENSG00000184185.6 | *KCNJ12* | chr17:21376196-21419872 | yes | no | 6.19E-08 | 0.117 | 1.31E-03 | 0.287 |
| ENSG00000141068.10 | *KSR1* | chr17:27456713-27626438 | NA | yes | 1.38E-10 | 0.443 | 2.92E-06 | 0.069 |
| ENSG00000266728.2 | *AC015688.3* | chr17:27623363-27640777 | NA | no | 6.68E-07 | 0.199 | 1.41E-02 | 0.069 |
| ENSG00000266872.1 | *RP11-19P22.8* | chr17:27625483-27626438 | NA | no | 1.00E-12 | 0.274 | 2.12E-08 | 0.076 |
| ENSG00000109103.8 | *UNC119* | chr17:28546706-28552668 | yes | no | 1.21E-16 | 0.469 | 2.56E-12 | 0.068 |
| ENSG00000076604.11 | *TRAF4* | chr17:28743983-28750958 | yes | yes | 1.84E-15 | 0.486 | 3.89E-11 | 0.042 |
| ENSG00000265474.1 | *AC010761.9* | chr17:28745568-28747652 | NA | no | 3.73E-07 | 0.114 | 7.88E-03 | 0.042 |
| ENSG00000265840.1 | *AC010761.10* | chr17:28749730-28750079 | NA | no | 2.27E-11 | 0.325 | 4.81E-07 | 0.045 |
| ENSG00000132589.12 | *FLOT2* | chr17:28879334-28897679 | yes | no | 4.72E-09 | -0.481 | 9.97E-05 | -0.072 |
| ENSG00000167536.10 | *DHRS13* | chr17:28897780-28903071 | yes | no | 2.13E-06 | -0.118 | 4.50E-02 | -0.055 |
| ENSG00000167543.12 | *TP53I13* | chr17:29566051-29573157 | yes | no | 9.86E-07 | -0.210 | 2.09E-02 | -0.041 |
| ENSG00000092871.13 | *RFFL* | chr17:35005989-35089319 | yes | no | 2.80E-07 | 0.242 | 5.92E-03 | 0.041 |
| ENSG00000271503.2 | *CCL5* | chr17:35871490-35880793 | yes | yes | 1.04E-10 | 0.281 | 2.21E-06 | 0.078 |
| ENSG00000126351.9 | *THRA* | chr17:40058289-40093867 | yes | yes | 1.59E-06 | -0.057 | 3.36E-02 | -0.069 |
| ENSG00000126368.5 | *NR1D1* | chr17:40092786-40100725 | yes | no | 1.33E-06 | 0.036 | 2.82E-02 | 0.030 |
| ENSG00000131759.14 | *RARA* | chr17:40309191-40357643 | yes | yes | 1.37E-11 | 0.162 | 2.89E-07 | 0.035 |
| ENSG00000126561.13 | *STAT5A* | chr17:42287546-42311943 | yes | yes | 2.50E-10 | 0.302 | 5.29E-06 | 0.054 |
| ENSG00000068079.4 | *IFI35* | chr17:43006724-43014456 | yes | yes | 4.79E-07 | 0.349 | 1.01E-02 | 0.037 |
| ENSG00000175832.9 | *ETV4* | chr17:43527843-43579620 | no | no | 6.83E-09 | 0.196 | 1.44E-04 | 0.100 |
| ENSG00000002919.11 | *SNX11* | chr17:48103356-48123074 | yes | no | 4.98E-18 | 0.357 | 1.05E-13 | 0.059 |
| ENSG00000159202.14 | *UBE2Z* | chr17:48908368-48929056 | yes | yes | 1.08E-11 | 0.168 | 2.28E-07 | 0.025 |
| ENSG00000108798.5 | *ABI3* | chr17:49210226-49223225 | yes | no | 1.44E-12 | 0.255 | 3.04E-08 | 0.041 |
| ENSG00000167085.8 | *PHB* | chr17:49404048-49414905 | yes | yes | 5.30E-10 | 0.336 | 1.12E-05 | 0.022 |
| ENSG00000250186.3 | *RP11-1079K10.4* | chr17:49404080-49405197 | NA | no | 1.43E-06 | 0.156 | 3.03E-02 | 0.020 |
| ENSG00000121104.4 | *FAM117A* | chr17:49710331-49789180 | yes | no | 1.55E-06 | -0.157 | 3.28E-02 | -0.029 |
| ENSG00000108821.10 | *COL1A1* | chr17:50183288-50201632 | yes | yes | 5.19E-07 | 0.108 | 1.10E-02 | 0.098 |
| ENSG00000108829.9 | *LRRC59* | chr17:50375058-50397553 | yes | no | 8.16E-08 | -0.174 | 1.73E-03 | -0.022 |
| ENSG00000108960.4 | *MMD* | chr17:55392612-55421992 | yes | no | 4.16E-11 | 0.202 | 8.79E-07 | 0.053 |
| ENSG00000262112.1 | *RP11-670E13.5* | chr17:56888879-56891841 | NA | no | 3.54E-09 | 0.132 | 7.50E-05 | 0.023 |
| ENSG00000263004.1 | *RP11-166P13.3* | chr17:57078297-57085024 | NA | no | 7.19E-07 | -0.096 | 1.52E-02 | -0.056 |
| ENSG00000108389.6 | *MTMR4* | chr17:58489528-58517905 | yes | no | 1.78E-06 | 0.136 | 3.76E-02 | 0.022 |
| ENSG00000008283.12 | *CYB561* | chr17:63432303-63446378 | yes | no | 3.13E-07 | 0.122 | 6.61E-03 | 0.073 |
| ENSG00000178607.12 | *ERN1* | chr17:64039141-64130819 | yes | yes | 2.90E-10 | -0.157 | 6.14E-06 | -0.062 |
| ENSG00000108370.12 | *RGS9* | chr17:65137430-65227703 | yes | no | 1.40E-07 | -0.193 | 2.95E-03 | -0.107 |
| ENSG00000070540.9 | *WIPI1* | chr17:68420947-68457513 | yes | no | 9.60E-11 | -0.282 | 2.03E-06 | -0.054 |
| ENSG00000125398.5 | *SOX9* | chr17:72121019-72126420 | yes | yes | 1.53E-10 | 0.229 | 3.24E-06 | 0.119 |
| ENSG00000180616.5 | *SSTR2* | chr17:73165011-73171046 | yes | no | 2.72E-15 | 0.211 | 5.75E-11 | 0.191 |
| ENSG00000260248.1 | *RP11-143K11.1* | chr17:73175482-73176633 | NA | NA | 1.45E-13 | 0.157 | 3.07E-09 | 0.214 |
| ENSG00000129657.11 | *SEC14L1* | chr17:77086715-77217101 | yes | no | 1.35E-10 | -0.317 | 2.85E-06 | -0.040 |
| ENSG00000141582.11 | *CBX4* | chr17:79833155-79839429 | yes | no | 2.35E-07 | -0.099 | 4.96E-03 | -0.028 |
| ENSG00000181523.9 | *SGSH* | chr17:80206715-80220923 | yes | yes | 5.59E-08 | -0.149 | 1.18E-03 | -0.042 |
| ENSG00000157637.9 | *SLC38A10* | chr17:81244999-81295547 | yes | no | 2.35E-06 | -0.148 | 4.96E-02 | -0.023 |
| ENSG00000225663.4 | *FAM195B* | chr17:81822360-81833302 | NA | no | 1.04E-07 | 0.175 | 2.20E-03 | 0.031 |
| ENSG00000185624.11 | *P4HB* | chr17:81843158-81860694 | NA | yes | 2.43E-13 | -1.481 | 5.14E-09 | -0.039 |
| ENSG00000262831.1 | *RP11-498C9.2* | chr17:81843164-81843958 | NA | no | 2.04E-10 | -0.656 | 4.32E-06 | -0.035 |
| ENSG00000197063.7 | *MAFG* | chr17:81918269-81927714 | yes | yes | 1.25E-06 | 0.069 | 2.64E-02 | 0.024 |
| ENSG00000178927.13 | *C17orf62* | chr17:82442588-82450829 | yes | no | 1.81E-06 | -0.247 | 3.82E-02 | -0.017 |
| ENSG00000118276.8 | *B4GALT6* | chr18:31622246-31685836 | yes | no | 3.14E-08 | 0.241 | 6.63E-04 | 0.056 |
| ENSG00000259985.1 | *RP11-549B18.1* | chr18:31685654-31686823 | NA | no | 5.13E-08 | 0.086 | 1.09E-03 | 0.052 |
| ENSG00000141644.14 | *MBD1* | chr18:50266881-50281774 | yes | no | 1.98E-09 | -0.236 | 4.18E-05 | -0.033 |
| ENSG00000154832.11 | *CXXC1* | chr18:50282342-50288304 | yes | no | 1.78E-08 | -0.389 | 3.75E-04 | -0.036 |
| ENSG00000091164.9 | *TXNL1* | chr18:56597207-56651600 | yes | no | 2.47E-07 | 0.101 | 5.22E-03 | 0.013 |
| ENSG00000172175.9 | *MALT1* | chr18:58671385-58750139 | yes | yes | 2.42E-11 | 0.131 | 5.13E-07 | 0.042 |
| ENSG00000267476.1 | *RP11-126O1.4* | chr18:58672612-58752730 | NA | no | 7.45E-12 | 0.046 | 1.58E-07 | 0.040 |
| ENSG00000267705.1 | *RP11-108P20.3* | chr18:58752178-58753898 | NA | no | 6.88E-07 | 0.078 | 1.46E-02 | 0.041 |
| ENSG00000074695.5 | *LMAN1* | chr18:59327822-59359962 | yes | yes | 1.30E-07 | -0.211 | 2.75E-03 | -0.030 |
| ENSG00000141682.11 | *PMAIP1* | chr18:59899947-59904306 | no | yes | 2.47E-12 | 0.345 | 5.22E-08 | 0.048 |
| ENSG00000141655.12 | *TNFRSF11A* | chr18:62325286-62391292 | yes | yes | 5.22E-07 | 0.181 | 1.10E-02 | 0.113 |
| ENSG00000141664.6 | *ZCCHC2* | chr18:62523006-62587709 | yes | no | 1.87E-06 | 0.136 | 3.96E-02 | 0.037 |
| ENSG00000171791.11 | *BCL2* | chr18:63123345-63320128 | yes | yes | 9.52E-09 | 0.158 | 2.01E-04 | 0.033 |
| ENSG00000166342.15 | *NETO1* | chr18:72742313-72868146 | yes | no | 1.36E-07 | 0.403 | 2.88E-03 | 0.215 |
| ENSG00000166347.15 | *CYB5A* | chr18:74250846-74292016 | yes | no | 1.19E-17 | 0.419 | 2.51E-13 | 0.068 |
| ENSG00000178184.12 | *PARD6G* | chr18:80157231-80247546 | yes | no | 1.40E-06 | 0.142 | 2.96E-02 | 0.100 |
| ENSG00000116017.7 | *ARID3A* | chr19:925780-975934 | yes | yes | 3.41E-07 | -0.149 | 7.20E-03 | -0.023 |
| ENSG00000167468.13 | *GPX4* | chr19:1103925-1106791 | yes | yes | 1.27E-06 | 0.350 | 2.68E-02 | 0.025 |
| ENSG00000071564.11 | *TCF3* | chr19:1609289-1652605 | yes | no | 2.75E-09 | -0.524 | 5.82E-05 | -0.033 |
| ENSG00000099875.11 | *MKNK2* | chr19:2037464-2051244 | yes | no | 4.28E-15 | 0.931 | 9.06E-11 | 0.074 |
| ENSG00000099840.10 | *IZUMO4* | chr19:2096428-2099593 | NA | no | 1.65E-08 | 0.189 | 3.49E-04 | 0.059 |
| ENSG00000104885.14 | *DOT1L* | chr19:2164148-2232578 | yes | no | 4.61E-07 | 0.167 | 9.74E-03 | 0.028 |
| ENSG00000176533.9 | *GNG7* | chr19:2511218-2702709 | yes | yes | 9.41E-08 | -0.213 | 1.99E-03 | -0.057 |
| ENSG00000105246.5 | *EBI3* | chr19:4229497-4237531 | yes | yes | 1.48E-14 | 0.762 | 3.13E-10 | 0.087 |
| ENSG00000074842.4 | *C19orf10* | chr19:4641373-4670370 | yes | no | 2.98E-07 | -0.376 | 6.31E-03 | -0.035 |
| ENSG00000125657.4 | *TNFSF9* | chr19:6530998-6535928 | yes | yes | 4.86E-08 | 0.186 | 1.03E-03 | 0.033 |
| ENSG00000125730.13 | *C3* | chr19:6677703-6730562 | yes | yes | 4.39E-08 | 0.184 | 9.29E-04 | 0.203 |
| ENSG00000125733.14 | *TRIP10* | chr19:6737924-6751526 | yes | yes | 7.92E-10 | 0.327 | 1.67E-05 | 0.053 |
| ENSG00000269680.1 | *CTD-3128G10.6* | chr19:6748292-6751467 | NA | no | 4.38E-16 | 0.123 | 9.26E-12 | 0.060 |
| ENSG00000090661.8 | *CERS4* | chr19:8206735-8262421 | NA | no | 3.83E-10 | 0.174 | 8.09E-06 | 0.040 |
| ENSG00000130813.14 | *C19orf66* | chr19:10086121-10093252 | yes | no | 1.14E-11 | 0.263 | 2.41E-07 | 0.042 |
| ENSG00000267387.1 | *CTD-2240E14.4* | chr19:10089031-10090377 | NA | no | 3.83E-07 | 0.070 | 8.09E-03 | 0.035 |
| ENSG00000130810.16 | *PPAN* | chr19:10106288-10111634 | yes | no | 4.64E-07 | 0.248 | 9.82E-03 | 0.026 |
| ENSG00000090339.5 | *ICAM1* | chr19:10270834-10286615 | yes | yes | 1.19E-13 | 0.496 | 2.53E-09 | 0.063 |
| ENSG00000267607.1 | *CTD-2369P2.8* | chr19:10285800-10289019 | NA | no | 7.93E-17 | 0.198 | 1.68E-12 | 0.074 |
| ENSG00000105371.8 | *ICAM4* | chr19:10286966-10288522 | no | yes | 2.20E-06 | -0.079 | 4.66E-02 | -0.072 |
| ENSG00000142453.8 | *CARM1* | chr19:10871512-10923070 | yes | yes | 7.04E-07 | 0.167 | 1.49E-02 | 0.020 |
| ENSG00000130175.6 | *PRKCSH* | chr19:11435287-11450968 | yes | yes | 1.48E-07 | -0.182 | 3.13E-03 | -0.015 |
| ENSG00000171223.5 | *JUNB* | chr19:12791495-12793315 | yes | yes | 8.22E-14 | 0.282 | 1.74E-09 | 0.043 |
| ENSG00000105607.9 | *GCDH* | chr19:12891025-12914207 | yes | no | 1.43E-07 | 0.191 | 3.03E-03 | 0.026 |
| ENSG00000187912.8 | *CLEC17A* | chr19:14583083-14611157 | yes | no | 3.39E-07 | 0.212 | 7.17E-03 | 0.039 |
| ENSG00000127528.5 | *KLF2* | chr19:16324816-16327874 | yes | no | 2.11E-07 | -0.607 | 4.46E-03 | -0.097 |
| ENSG00000074855.7 | *ANO8* | chr19:17323222-17334829 | NA | no | 5.20E-09 | -0.058 | 1.10E-04 | -0.050 |
| ENSG00000105642.12 | *KCNN1* | chr19:17951292-18000080 | yes | no | 4.95E-07 | 0.170 | 1.05E-02 | 0.219 |
| ENSG00000216490.3 | *IFI30* | chr19:18173161-18178117 | yes | yes | 2.32E-20 | 1.285 | 4.91E-16 | 0.075 |
| ENSG00000105656.9 | *ELL* | chr19:18442662-18522127 | yes | no | 1.81E-07 | -0.102 | 3.84E-03 | -0.025 |
| ENSG00000105669.9 | *COPE* | chr19:18899513-18919397 | yes | no | 1.66E-06 | -0.281 | 3.52E-02 | -0.019 |
| ENSG00000181035.10 | *SLC25A42* | chr19:19063998-19112888 | yes | no | 2.00E-06 | -0.162 | 4.24E-02 | -0.042 |
| ENSG00000166289.5 | *PLEKHF1* | chr19:29665055-29675457 | yes | no | 1.79E-11 | 0.126 | 3.78E-07 | 0.052 |
| ENSG00000167604.10 | *NFKBID* | chr19:35887652-35902303 | yes | yes | 5.89E-07 | 0.211 | 1.25E-02 | 0.044 |
| ENSG00000167645.13 | *YIF1B* | chr19:38305103-38317273 | yes | no | 1.48E-06 | -0.165 | 3.14E-02 | -0.023 |
| ENSG00000130755.9 | *GMFG* | chr19:39328352-39342372 | yes | no | 9.47E-09 | -0.446 | 2.00E-04 | -0.029 |
| ENSG00000105223.15 | *PLD3* | chr19:40348455-40380439 | yes | no | 7.76E-08 | -0.331 | 1.64E-03 | -0.033 |
| ENSG00000105329.6 | *TGFB1* | chr19:41301586-41353911 | yes | yes | 1.19E-06 | 0.222 | 2.51E-02 | 0.018 |
| ENSG00000105404.7 | *RABAC1* | chr19:41956680-41959390 | yes | no | 6.96E-12 | -0.405 | 1.47E-07 | -0.050 |
| ENSG00000104856.10 | *RELB* | chr19:45001429-45038194 | yes | yes | 1.03E-13 | 0.239 | 2.18E-09 | 0.056 |
| ENSG00000125753.10 | *VASP* | chr19:45506578-45526983 | yes | yes | 3.00E-07 | 0.395 | 6.34E-03 | 0.027 |
| ENSG00000160013.5 | *PTGIR* | chr19:46620467-46625118 | yes | yes | 1.06E-06 | 0.137 | 2.24E-02 | 0.079 |
| ENSG00000105438.5 | *KDELR1* | chr19:48382569-48391553 | yes | no | 1.79E-06 | -0.166 | 3.80E-02 | -0.019 |
| ENSG00000126457.17 | *PRMT1* | chr19:49675785-49689029 | yes | yes | 1.07E-09 | 0.330 | 2.26E-05 | 0.020 |
| ENSG00000125826.16 | *RBCK1* | chr20:407497-430966 | yes | yes | 4.20E-07 | 0.158 | 8.89E-03 | 0.019 |
| ENSG00000088812.14 | *ATRN* | chr20:3471039-3651122 | yes | yes | 9.54E-09 | -0.048 | 2.02E-04 | -0.023 |
| ENSG00000125843.7 | *AP5S1* | chr20:3820523-3828837 | NA | no | 1.73E-06 | -0.063 | 3.66E-02 | -0.022 |
| ENSG00000088888.14 | *MAVS* | chr20:3846798-3876123 | yes | yes | 2.94E-08 | -0.045 | 6.21E-04 | -0.015 |
| ENSG00000132646.7 | *PCNA* | chr20:5114952-5126626 | yes | yes | 4.60E-11 | 0.332 | 9.72E-07 | 0.020 |
| ENSG00000125845.6 | *BMP2* | chr20:6767663-6780280 | no | no | 7.18E-07 | 0.144 | 1.52E-02 | 0.229 |
| ENSG00000125844.12 | *RRBP1* | chr20:17613677-17682295 | yes | no | 9.61E-11 | -0.394 | 2.03E-06 | -0.048 |
| ENSG00000089050.11 | *RBBP9* | chr20:18486539-18497243 | yes | no | 8.13E-12 | 0.164 | 1.72E-07 | 0.050 |
| ENSG00000101310.11 | *SEC23B* | chr20:18507492-18561415 | yes | no | 5.36E-07 | -0.154 | 1.13E-02 | -0.018 |
| ENSG00000173418.8 | *NAA20* | chr20:20017115-20033655 | NA | no | 5.87E-08 | 0.112 | 1.24E-03 | 0.016 |
| ENSG00000154930.11 | *ACSS1* | chr20:25006229-25058980 | yes | no | 2.13E-06 | -0.117 | 4.50E-02 | -0.026 |
| ENSG00000101294.13 | *HM13* | chr20:31514427-31577923 | yes | yes | 4.62E-09 | -0.346 | 9.77E-05 | -0.034 |
| ENSG00000230613.1 | *HM13-AS1* | chr20:31567706-31573263 | NA | no | 2.70E-09 | -0.081 | 5.72E-05 | -0.037 |
| ENSG00000171552.9 | *BCL2L1* | chr20:31664451-31723989 | yes | yes | 8.59E-07 | 0.157 | 1.82E-02 | 0.028 |
| ENSG00000126003.6 | *PLAGL2* | chr20:32192502-32207791 | yes | no | 2.59E-09 | -0.071 | 5.49E-05 | -0.021 |
| ENSG00000101421.3 | *CHMP4B* | chr20:33811303-33854366 | yes | no | 2.13E-06 | 0.100 | 4.50E-02 | 0.011 |
| ENSG00000131069.16 | *ACSS2* | chr20:34872145-34927962 | yes | no | 1.13E-07 | -0.341 | 2.40E-03 | -0.101 |
| ENSG00000088298.9 | *EDEM2* | chr20:35115356-35147364 | yes | no | 6.86E-08 | -0.123 | 1.45E-03 | -0.031 |
| ENSG00000126005.12 | *MMP24-AS1* | chr20:35216461-35278131 | NA | no | 1.29E-10 | -0.145 | 2.72E-06 | -0.032 |
| ENSG00000080845.14 | *DLGAP4* | chr20:36306335-36528637 | yes | no | 7.00E-07 | 0.156 | 1.48E-02 | 0.028 |
| ENSG00000118707.6 | *TGIF2* | chr20:36573487-36593950 | no | no | 8.87E-08 | 0.101 | 1.88E-03 | 0.024 |
| ENSG00000118705.13 | *RPN2* | chr20:37178409-37241623 | yes | no | 3.14E-09 | -0.426 | 6.64E-05 | -0.026 |
| ENSG00000198959.8 | *TGM2* | chr20:38127386-38166578 | yes | yes | 1.79E-13 | 0.200 | 3.78E-09 | 0.164 |
| ENSG00000132824.10 | *SERINC3* | chr20:44496220-44522109 | yes | no | 6.01E-07 | -0.095 | 1.27E-02 | -0.018 |
| ENSG00000124145.6 | *SDC4* | chr20:45325287-45348424 | yes | no | 3.24E-10 | 0.213 | 6.85E-06 | 0.161 |
| ENSG00000101017.10 | *CD40* | chr20:46118271-46129863 | no | yes | 1.98E-16 | 0.630 | 4.18E-12 | 0.072 |
| ENSG00000149654.6 | *CDH22* | chr20:46173732-46308498 | yes | no | 9.23E-07 | 0.038 | 1.95E-02 | 0.234 |
| ENSG00000124207.13 | *CSE1L* | chr20:49046245-49096960 | yes | no | 1.98E-06 | 0.135 | 4.19E-02 | 0.016 |
| ENSG00000124214.16 | *STAU1* | chr20:49113338-49188367 | yes | yes | 2.33E-07 | 0.095 | 4.93E-03 | 0.016 |
| ENSG00000124201.11 | *ZNFX1* | chr20:49237945-49278426 | yes | no | 2.56E-08 | 0.092 | 5.41E-04 | 0.021 |
| ENSG00000158470.5 | *B4GALT5* | chr20:49632944-49713878 | yes | no | 2.95E-08 | 0.101 | 6.23E-04 | 0.032 |
| ENSG00000171940.10 | *ZNF217* | chr20:53567064-53609907 | yes | no | 1.99E-08 | -0.143 | 4.21E-04 | -0.031 |
| ENSG00000130589.13 | *HELZ2* | chr20:63558085-63574239 | NA | no | 3.41E-14 | 0.277 | 7.21E-10 | 0.082 |
| ENSG00000154640.11 | *BTG3* | chr21:17593652-17612947 | yes | no | 5.05E-07 | 0.114 | 1.07E-02 | 0.024 |
| ENSG00000185433.5 | *LINC00158* | chr21:25385819-25431701 | NA | no | 7.96E-12 | 0.224 | 1.68E-07 | 0.091 |
| ENSG00000234883.3 | *MIR155HG* | chr21:25561908-25575168 | NA | no | 1.32E-17 | 0.908 | 2.79E-13 | 0.102 |
| ENSG00000154719.10 | *MRPL39* | chr21:25585655-25607517 | yes | no | 1.86E-07 | 0.143 | 3.93E-03 | 0.018 |
| ENSG00000156239.8 | *N6AMT1* | chr21:28872190-28885371 | yes | no | 7.76E-07 | 0.051 | 1.64E-02 | 0.026 |
| ENSG00000159110.16 | *IFNAR2* | chr21:33229900-33265675 | yes | yes | 3.87E-14 | -0.312 | 8.18E-10 | -0.042 |
| ENSG00000223799.1 | *IL10RB-AS1* | chr21:33263872-33266260 | NA | no | 3.95E-11 | -0.146 | 8.35E-07 | -0.036 |
| ENSG00000142166.9 | *IFNAR1* | chr21:33324476-33359862 | yes | yes | 6.65E-12 | -0.235 | 1.41E-07 | -0.041 |
| ENSG00000182670.10 | *TTC3* | chr21:37073225-37203112 | yes | no | 4.09E-08 | -0.163 | 8.66E-04 | -0.027 |
| ENSG00000183486.9 | *MX2* | chr21:41361942-41409390 | yes | no | 1.94E-10 | 0.385 | 4.10E-06 | 0.072 |
| ENSG00000157601.10 | *MX1* | chr21:41420303-41459214 | yes | yes | 9.81E-10 | 0.702 | 2.07E-05 | 0.052 |
| ENSG00000228318.2 | *AP001610.5* | chr21:41441393-41445708 | NA | no | 3.12E-09 | 0.342 | 6.60E-05 | 0.052 |
| ENSG00000160216.15 | *AGPAT3* | chr21:43865185-43986536 | yes | no | 2.69E-07 | 0.144 | 5.68E-03 | 0.021 |
| ENSG00000184979.9 | *USP18* | chr22:18149898-18177397 | yes | yes | 3.76E-15 | 0.351 | 7.94E-11 | 0.133 |
| ENSG00000133460.16 | *SLC2A11* | chr22:23856702-23886309 | yes | yes | 2.72E-08 | -0.146 | 5.76E-04 | -0.048 |
| ENSG00000100024.11 | *UPB1* | chr22:24494106-24528390 | no | no | 2.32E-09 | 0.118 | 4.90E-05 | 0.139 |
| ENSG00000100219.13 | *XBP1* | chr22:28794554-28800597 | yes | yes | 3.21E-07 | -0.870 | 6.80E-03 | -0.056 |
| ENSG00000186998.12 | *EMID1* | chr22:29205850-29259597 | yes | no | 3.14E-09 | 0.425 | 6.64E-05 | 0.113 |
| ENSG00000100280.13 | *AP1B1* | chr22:29327679-29423179 | yes | no | 1.11E-06 | 0.122 | 2.35E-02 | 0.016 |
| ENSG00000185339.5 | *TCN2* | chr22:30606837-30627278 | yes | no | 8.61E-07 | 0.296 | 1.82E-02 | 0.090 |
| ENSG00000100220.8 | *RTCB* | chr22:32387581-32412255 | NA | no | 4.72E-10 | 0.175 | 9.97E-06 | 0.021 |
| ENSG00000100284.17 | *TOM1* | chr22:35299274-35347994 | yes | no | 1.22E-08 | -0.257 | 2.59E-04 | -0.060 |
| ENSG00000221963.5 | *APOL6* | chr22:35648394-35668409 | yes | yes | 1.90E-09 | 0.125 | 4.02E-05 | 0.056 |
| ENSG00000189060.5 | *H1F0* | chr22:37805092-37807436 | yes | yes | 1.02E-06 | -0.111 | 2.16E-02 | -0.087 |
| ENSG00000185022.8 | *MAFF* | chr22:38201881-38216511 | no | no | 1.29E-12 | 0.142 | 2.73E-08 | 0.117 |
| ENSG00000100393.9 | *EP300* | chr22:41091785-41180077 | yes | yes | 2.03E-07 | -0.070 | 4.30E-03 | -0.026 |
| ENSG00000232754.1 | *RP1-85F18.6* | chr22:41169189-41183144 | NA | no | 1.79E-07 | -0.051 | 3.78E-03 | -0.022 |
| ENSG00000100403.11 | *ZC3H7B* | chr22:41301521-41360147 | yes | no | 1.15E-06 | 0.059 | 2.43E-02 | 0.012 |
| ENSG00000184208.10 | *C22orf46* | chr22:41688938-41698136 | NA | no | 2.37E-08 | -0.081 | 5.00E-04 | -0.029 |
| ENSG00000167077.9 | *MEI1* | chr22:41699498-41799456 | no | no | 1.17E-07 | -0.376 | 2.48E-03 | -0.059 |
| ENSG00000100207.15 | *TCF20* | chr22:42160012-42343616 | yes | no | 5.11E-09 | 0.074 | 1.08E-04 | 0.023 |
| ENSG00000100422.10 | *CERK* | chr22:46684410-46738261 | yes | yes | 8.38E-14 | -0.191 | 1.77E-09 | -0.034 |
| ENSG00000198355.4 | *PIM3* | chr22:49960512-49964080 | NA | yes | 1.03E-09 | 0.140 | 2.17E-05 | 0.030 |
| ENSG00000188130.10 | *MAPK12* | chr22:50245449-50261825 | yes | yes | 1.93E-06 | 0.134 | 4.09E-02 | 0.033 |
| ENSG00000185386.11 | *MAPK11* | chr22:50263712-50270767 | yes | yes | 1.23E-12 | 0.142 | 2.60E-08 | 0.066 |
| ENSG00000025708.9 | *TYMP* | chr22:50525751-50530085 | yes | yes | 9.58E-14 | 0.534 | 2.03E-09 | 0.088 |
| ENSG00000177989.10 | *ODF3B* | chr22:50529709-50532580 | NA | no | 2.30E-11 | 0.307 | 4.87E-07 | 0.082 |
| ENSG00000272666.1 | *CTA-384D8.35* | chr22:50542304-50542906 | NA | no | 3.77E-14 | 0.166 | 7.96E-10 | 0.172 |
| ENSG00000273272.1 | *CTA-384D8.34* | chr22:50542649-50543011 | NA | no | 1.36E-09 | 0.309 | 2.87E-05 | 0.120 |
| ENSG00000123595.6 | *RAB9A* | chrX:13689120-13710506 | yes | no | 5.66E-11 | 0.359 | 1.20E-06 | 0.062 |
| ENSG00000046651.11 | *OFD1* | chrX:13734744-13769353 | yes | no | 3.41E-12 | 0.178 | 7.22E-08 | 0.050 |
| ENSG00000046647.10 | *GEMIN8* | chrX:14008278-14029893 | yes | no | 1.69E-08 | 0.109 | 3.57E-04 | 0.030 |
| ENSG00000102048.12 | *ASB9* | chrX:15235287-15270467 | no | yes | 1.56E-06 | 0.130 | 3.30E-02 | 0.216 |
| ENSG00000149970.11 | *CNKSR2* | chrX:21374417-21654695 | yes | no | 3.70E-07 | 0.158 | 7.82E-03 | 0.139 |
| ENSG00000102172.12 | *SMS* | chrX:21940572-21994835 | NA | no | 4.05E-08 | 0.148 | 8.56E-04 | 0.019 |
| ENSG00000123130.13 | *ACOT9* | chrX:23702252-23766475 | yes | no | 4.37E-07 | 0.168 | 9.24E-03 | 0.038 |
| ENSG00000198947.11 | *DMD* | chrX:31097676-33339441 | yes | no | 2.79E-07 | 0.134 | 5.90E-03 | 0.059 |
| ENSG00000165169.7 | *DYNLT3* | chrX:37836756-37847637 | yes | no | 5.86E-07 | 0.059 | 1.24E-02 | 0.025 |
| ENSG00000185753.9 | *CXorf38* | chrX:40626920-40647554 | yes | no | 1.86E-06 | 0.065 | 3.94E-02 | 0.020 |
| ENSG00000180182.7 | *MED14* | chrX:40648305-40735858 | yes | no | 5.26E-07 | 0.151 | 1.11E-02 | 0.028 |
| ENSG00000270069.1 | *MIR222HG* | chrX:45745210-45770274 | NA | no | 5.09E-10 | 0.090 | 1.08E-05 | 0.113 |
| ENSG00000008056.9 | *SYN1* | chrX:47571897-47619853 | yes | no | 3.37E-08 | 0.047 | 7.12E-04 | 0.163 |
| ENSG00000102100.11 | *SLC35A2* | chrX:48903181-48911958 | yes | no | 1.62E-08 | -0.085 | 3.43E-04 | -0.022 |
| ENSG00000068308.10 | *OTUD5* | chrX:48922027-48958386 | yes | yes | 1.34E-06 | -0.086 | 2.84E-02 | -0.015 |
| ENSG00000196998.12 | *WDR45* | chrX:49074432-49101170 | yes | no | 4.73E-07 | -0.206 | 1.00E-02 | -0.033 |
| ENSG00000179222.14 | *MAGED1* | chrX:51803006-51902357 | yes | yes | 4.09E-08 | -0.308 | 8.64E-04 | -0.032 |
| ENSG00000184205.11 | *TSPYL2* | chrX:53082366-53088540 | yes | no | 2.87E-09 | -0.152 | 6.07E-05 | -0.038 |
| ENSG00000158526.7 | *TSR2* | chrX:54440400-54445487 | yes | no | 1.47E-09 | 0.098 | 3.11E-05 | 0.017 |
| ENSG00000188021.8 | *UBQLN2* | chrX:56563638-56567868 | yes | no | 2.14E-06 | 0.051 | 4.52E-02 | 0.015 |
| ENSG00000184675.6 | *AMER1* | chrX:64185116-64205744 | NA | no | 2.61E-08 | 0.044 | 5.52E-04 | 0.031 |
| ENSG00000067177.11 | *PHKA1* | chrX:72578813-72714319 | yes | no | 6.06E-07 | 0.087 | 1.28E-02 | 0.184 |
| ENSG00000102158.16 | *MAGT1* | chrX:77826363-77895593 | yes | no | 7.56E-07 | -0.144 | 1.60E-02 | -0.024 |
| ENSG00000173198.5 | *CYSLTR1* | chrX:78271463-78327691 | no | yes | 6.86E-08 | -0.144 | 1.45E-03 | -0.050 |
| ENSG00000072133.7 | *RPS6KA6* | chrX:84058345-84187925 | yes | no | 2.63E-07 | 0.129 | 5.56E-03 | 0.176 |
| ENSG00000147202.14 | *DIAPH2* | chrX:96684662-97604997 | yes | no | 1.86E-06 | 0.047 | 3.94E-02 | 0.024 |
| ENSG00000126945.8 | *HNRNPH2* | chrX:101408294-101414133 | yes | no | 1.12E-06 | 0.093 | 2.36E-02 | 0.015 |
| ENSG00000102401.16 | *ARMCX3* | chrX:101622796-101627843 | yes | no | 8.16E-08 | -0.123 | 1.73E-03 | -0.027 |
| ENSG00000063587.13 | *ZNF275* | chrX:153334154-153360110 | NA | no | 1.82E-06 | -0.062 | 3.84E-02 | -0.024 |
| ENSG00000259886.2 | *U82695.10* | chrX:153423751-153426481 | NA | no | 1.53E-06 | 0.057 | 3.23E-02 | 0.060 |
| ENSG00000180879.10 | *SSR4* | chrX:153793515-153798505 | yes | no | 2.85E-07 | -0.521 | 6.03E-03 | -0.032 |
| ENSG00000196924.11 | *FLNA* | chrX:154348523-154374638 | yes | no | 8.40E-08 | 0.586 | 1.78E-03 | 0.033 |
| The analyses tabulated here are for the 1 058 transcripts with differential expression by schizophrenia status from the study of 529 cases and 660 controls using the 12 measured covariates, and restricted to the 21 146 genes detected (i.e., a non-zero FPKM) in 80% or more subjects (Bonferroni *P*-values are adjusted for 21 146 genes analyzed). Immune-related, i.e., protein-coding gene function containing “immune” from genecards.org, is noted (yes, no, or NA for those unlisted). Genes are also tabulated for brain expression (yes, no, or NA for those unlisted) in the adult (hbatlas.org, ^1^). The regression coefficient refers to cases compared to controls, i.e. a value of 1.0 means that the average expression level in cases exceeds that of controls by that amount (when analyzing square root of FPKM values and when simultaneously accounting for confounder variables). Fold change (FC) of the mean expression level by affection status is also tabulated, with positive FC indicating higher expression in cases. | | | | | | | | |

**References.**

1. Kang HJ, Kawasawa YI, Cheng F, Zhu Y, Xu X, Li M*, et al*. Spatio-temporal transcriptome of the human brain. *Nature* 2011; **478**(7370)**:** 483-489.
