# Supplementary material for: Transcriptome sequencing study implicates immune-related genes differentially expressed in schizophrenia: new data and a meta-analysis
Source: Transl Psychiatry. 2017 Apr 18;7(4):e1093–. doi: 10.1038/tp.2017.47 (PMC5416689; doi:10.1038/tp.2017.47)
Supplement: Supplementary Table 2 [file tp201747x3.docx]

| **Table S2. Selected examples of genes differentially expressed (Bonferroni *P*≤0.05) in schizophrenia.** | | |
| --- | --- | --- |
| **Gene(s)** | **Comments** | **References** |
| *PIK3CD* | See discussion text. | ^1-6^ |
| *PINK1* | Schizophrenia spectrum disorders were enriched in a large family with monogenic (*PINK1*) Parkinson's Disease, but no support was found for common variants at *PINK1* increasing risk for schizophrenia in an Asian sample. | ^7-9^ |
| *PINK1-AS* | See entry for *PINK1*. | See entry for *PINK1*. |
| *RPS6KA1* | This gene, which has been implicated in controlling cell growth and differentiation, was reduced in postmortem frontal cortex of schizophrenics versus controls, with less pronounced decreases noted in subject groups with depression and bipolar disorder. | ^10, 11^ |
| *POU3F1* | The brain-expressed transcription factor, *POU3F1*, was noted to be more highly expressed in the frontal and temporal cortex of schizophrenics versus controls in one study, but it was later unreplicated. A SNP (rs1344706) reported as associated in the schizophrenia candidate gene, *ZNF804A*, lies in a predicted binding site of *POU3F1*. | ^12-14^ |
| *RIMS3* | Increased expression of *RIMS3* (which regulates synaptic membrane exocytosis) has been reported in the amygdala in schizophrenia cases. The gene has also been implicated in an autism case report of a de novo 1p34.2 microdeletion. LCLs from autism patients with either maternal duplications of 15q11q13 or fragile X syndrome show lowered expression of *RIMS3* compared to controls. | ^15-18^ |
| *PDE4B* | See discussion text. | ^19-26^ |
| *GBP2* | These proinflammatory guanylate binding proteins (both upregulated in case LCLs) are induced by interferon and important for host defense against intracellular pathogens. | ^27, 28^ |
| *GBP4* | See entry for *GBP2*. | ^27, 28^ |
| *FAM69A* | See discussion text. | ^29-32^ |
| *RBM8A* | *RBM8A* is one of the genes within the 1q21.1 CNV associated with schizophrenia (as well as intellectual disability, autism, and microcephaly), and plays important neurodevelopmental roles. In mice, haploinsufficiency causes severe microcephaly and defective neurogenesis. *RBM8A* overexpression has been reported to stimulate embryonic neural progenitor cells (NPC) proliferation and suppress neuronal differentiation, with *RBM8A* knockdown reducing NPC proliferation and promoting premature neuronal differentiation. | ^33-37^ |
| *IL6R* | Nominal association of this gene with schizophrenia has been reported in an Asian sample (Han Chinese). Pro-inflammatory cytokines, especially IL-6, have been shown to be elevated in rodent models of schizophrenia and autism, as well as in peripheral blood of schizophrenics. | ^38, 39^ |
| *SYT11* | See discussion text. | ^40^ |
| *SLAMF1* | SNPs ~10kb downstream of *SLAMF1* (a signaling lymphocyte activation molecule) have been reported as nominally associated (*P*~2E-07) to schizophrenia in an Asian sample (Han Chinese) GWAS. | ^41^ |
| *SRGAP2* | *SRGAP2* plays important roles in cortical neuronal development (e.g., neuronal migration), and has been implicated in seizures (in association with a severe neurodevelopmental syndrome, and as seen in *SRGAP2* knockout mice). | ^42-44^ |
| *CR1* | See discussion text. | ^45-48^ |
| *ATF3* | The transcription factor, *ATF3*, has been reported to be upregulated in monocytes of schizophrenia and bipolar disorder cases. | ^49^ |
| *VRK2* | *VRK2* acts as an effector of signaling pathways that regulate apoptosis (has been found to be anti-apoptotic when over-expressed), and this gene has been reported as having genome-wide significant association in schizophrenia GWAS. *VRK2* has been reported to be upregulated in schizophrenia cases than in controls. Differences in white matter connectivity were found for different rs2312147 (a *VRK2* GWAS risk variant) genotypes in schizophrenia cases versus controls. | ^50-53^ |
| *IL1B* | This cytokine is involved in cell proliferation, differentiation, and apoptosis. DBA/2J mice have less social interaction and reduced pre-pulse inhibition compared to C57BL/6N mice, and also show higher expression of *Il1b* in the cortex and hypothalamus. Meta-analyses have shown association of *IL1B* with schizophrenia especially in EA samples. | ^54-57^ |
| *INSIG2* | *INSIG1* variants have been reported to be associated with various aspects (e.g., weight gain, glucose levels) of metabolic syndrome in schizophrenic cases treated with antipsychotics, though some studies are negative. | ^58-60^ |
| *STAT1* | One study has reported that antipsychotics (aripiprazole and minocycline) reduce phosphorylation of *STAT1* leading to reduction in the proinflammatory cytokine *TNF* in microglial cells, thus inhibiting oligodendrocyte damage by suppressing *IFNG*-activated microglial cells. | ^61^ |
| *CHL1* | *CHL1* is a neural cell adhesion molecule that may be involved in signal transduction pathways, e.g., by regulating SNARE complexes. A 3p26.3 locus (with *CHL1* being the nearest gene) has been implicated in schizophrenia (CNV deletion), intellectual disability (translocation breakpoint in *CHL1*), and autism (CNV duplication). Allelic associations to schizophrenia have been reported for *CHL1*. *CHL1* has also been reported as a putative SSRI response biomarker. | ^62-67^ |
| *CHL1-AS1* | See entry for *CHL1*. | See entry for *CHL1*. |
| *OXTR* | *OXTR* is the G-protein coupled receptor for oxytocin. Variants in *OXTR* have been reported associated with aspects of social impairment in schizophrenia. Nominal association has been reported for schizophrenia itself, but without replication. | ^68-72^ |
| *OXSR1* | *OXSR1* was reported to have increased expression in prefrontal cortex in schizophrenia cases versus controls. | ^73^ |
| *CCR5* | *CCR5* is a G protein-coupled chemokine receptor expressed by T cells. Defective alleles of *CCR5* are associated with resistance to HIV infection. The 32-bp deletion allele of *CCR5* has been suggested to be associated with age of onset for schizophrenia. | ^74^ |
| *CD80* | Activation of the *CD80* receptor induces T-cell proliferation and cytokine production. The antipsychotic, haloperidol, has been shown to downregulate *CD80* expression, and to suppress immune responses mediated by dendritc (antigen-presenting) cells. | ^75^ |
| *NFKB1* | GWAS in an Asian sample found *NFKB1* variants to be nominally associated (P~2E-07) with treatment-refractory schizophrenia, including an indel in the promoter region (*P*~5E-06). | ^41^ |
| *NEUROG2* | *NEUROG2* is a transcription factor that is involved in neurodevelopment, including playing a role in the differentiation and survival of midbrain dopaminergic neurons. Knockdown of TCF4 (known to be genome-wide significantly associated with schizophrenia from GWAS) in SH-SY5Y neuroblastoma cells led to downregulation of *NEUROG2*. | ^76-78^ |
| *UGT8* | *UGT8* is involved in the biosynthesis of galactocerebrosides (abundant sphingolipids of the myelin membrane in the CNS). *UGT8* has been reported to be downregulated in prefrontal cortex of schizophrenia cases, and to be downregulated in temporal cortex of depression cases. | ^79, 80^ |
| *CASP3* | *CASP3* is involved in apoptosis, and is the predominant caspase involved in the cleavage of amyloid-beta 4A precursor protein (which is associated with neuronal death in Alzheimer's disease). Primary fibroblast cultures from antipsychotic-naïve patients with first-episode schizophrenia showed elevated *CASP3* activity. However, schizophrenic brains (internal capsule) showed downregulation of *CASP3*. | ^81, 82^ |
| *HMGCS1* | *HMGCS1* is involved in lipogenesis, and has been reported to be upregulated by treatment with clozapine and haloperidol in cultured human glioma cells. | ^83^ |
| *MEF2C* | *MEF2C* has roles neurodevelopment and hippocampal memory functions, especially facilitating context-dependent fear conditioning. *MEF2C* has been shown to undergone significant positive selection in humans, and has been proposed to be involved in schizophrenia pathogenesis. Knockdown of *TCF4* (known to be genome-wide significantly associated with schizophrenia from GWAS) in SH-SY5Y neuroblastoma cells led to upregulation of *MEF2C*. Haploinsufficiency of *MEF2C* causes an intellectual disability syndrome that includes seizures and stereotypical movements. | ^77, 84-87^ |
| *SEMA6A* | *SEMA6A* plays important neurodevelopment roles, e.g., axon guidance, laminar connectivity, neuronal migration, dendrite development. Mice with mutations for this gene displayed subtle defects in limbic and cortical cellular organization, lamination, and connectivity, as well as behavioral changes (e.g., exploratory behavior, social interaction) characteristic of animal models of psychosis and reversible by antipsychotic treatment. | ^88, 89^ |
| *VDAC1* | *VDAC1* is a voltage-dependent anion channel located in the outer mitochondrial membrane (also found on the the plasma membrane). The protein expression of *Vdac1* was downregulated in the medial prefrontal cortex of rats treated with phencyclidine (a psychotogenic drug). | ^90^ |
| *HSPA9* | *HSPA9,* is a heat-shock cognate gene that plays a role in cell proliferation, stress response, and maintenance of the mitochondria. *HSPA9*, also called *40-kDa Catecholamine Regulated Protein* (*CRP40*), has been reported to be downregulated in post-mortem brain of schizophrenia cases. *HSPA9* was also noted to be downregulated in blood of both first episode schizophrenia subjects and chronic schizophrenia subjects, compared to controls. | ^91, 92^ |
| *MZB1* | One of the haploinsufficient genes in 22q11.2 deletion syndrome patients is *MIR185*, which regulates T cell development, in part through *MZB1* (which is an endoplasmic reticulum calcium regulator). Levels of *MZB1* were noted to be elevated in thymocyte extracts from several 22q11.2 deletion syndrome patients. | ^93^ |
| *HBEGF* | *HBEGF* contributes in several ways to neurodevelopment (neurogenesis, proliferation, differentiation, synaptic plasticity), including promoting the survival of dopaminergic neurons. *HBEGF* knockout mice showed behavioral abnormalities that were reduced by antipsychotics, altered brain dopamine levels, and other abnormalities, which suggest possible involvement in schizophrenia pathogenesis. | ^94-96^ |
| *SYNPO* | *SYNPO* plays a role in actin-based cell shape and motility, has been found in postsynaptic densities and associated dendritic spine, and is an N-methyl-D-aspartate (NMDA)-interacting protein. A proteomic study of anterior cingulate cortex found downregulation of *SYNPO* in schizophrenia brains. | ^97, 98^ |
| *CLINT1* | *CLINT1* interacts with clathrin, and may be involved in the formation of clathrin coated vesicles and protein trafficking. *CLINT1* has been associated with schizophrenia in some samples, but not in others. Clozapine treatment of SH-SY5Y human neuroblastoma cells has been shown to increase expression of *CLINT1* (also found in treated mice). | ^99-107^ |
| *GPRIN1* | *GPRIN1* has been suggested to play roles in neuronal migration and differentiation, as well as maintenance of specific neural circuitries in adulthood. In particular, *GPRIN1* has been reported to help regulate axon growth in hippocampal neurons. | ^108-110^ |
| *HLA-F* | *HLA-F* is found in the xMHC, the strongest associated region reported in schizophrenia GWAS. | See text. |
| *GLO1* | This enzyme protects against glycation by catalyzing the conversion of reactive, acyclic alpha-oxoaldehydes. It has been previously implicated in autism and schizophrenia in some studies of carbonyl stress, and shown to be protective in a mouse model of Parkinson’s disease. | ^111-118^ |
| *SLC29A1* | *SLC29A1* facilitates transport of nucleosides such as adenosine across membranes until the concentration is equalized on each side of the membrane (it is also called *equilibrative nucleoside transporter 1*, or *ENT1*). Recently it has been suggested that *SLC29A1* may also affect glutamate levels by regulating excitatory amino acid transporter expression and activity. Protein expression of SLC29A1was reduced in the brain (superior temporal gyrus, anterior cingulate cortex) in elderly schizophrenia cases. | ^119-121^ |
| *SGK1* | This gene participates in the regulation of neuroexcitability, inflammation, cell proliferation and apoptosis. | ^122^ |
| *FSCN1* | *FSCN1* is an actin-binding protein, which plays a critical role in cell migration, motility, adhesion, and cellular interactions. The protein expression of *Fscn1* was upregulated in the medial prefrontal cortex of rats treated with phencyclidine (a psychotogenic drug). *FSCN1* has been found to be upregulated in a mouse model of Down's syndrome. | ^90, 123^ |
| *CAV1* | *CAV1* is a caveolin, a group of genes which plays roles in vesicular trafficking, lipid homeostasis, and signal transduction through their functions as scaffolding proteins. Knockout mice for *Cav-1* are sensitized to the psychotogenic effects of phencyclidine. Disruption of *CAV1* has been reported in a childhood-onset schizophrenia sample. | ^124-127^ |
| *ASAH1* | *ASAH1* (also called *acid ceramidase*, *AC*) catalyzes the synthesis and degradation of ceramide into sphingosine and fatty acid, and has been implicated in a lysosomal storage disorder known as Farber disease . A small study of Asian samples found decreased *ASAH1* expression in peripheral blood in 14 family history positive schizophrenia cases compared to 30 controls. The same study also found nominal association (*P*=0.026) with schizophrenia in 191 parent-offspring trios. Another study found decreased expression of *ASAH1* in prefrontal cortex of schizophrenia subjects. | ^79, 128-130^ |
| *ENTPD4* | *ENTPD4* plays a role in salvaging nucleotides from lysosomes. After giving the psychotogenic drugs methamphetamine or phencyclidine to rats, the homologous gene was one of three rat genes (1) with a human homolog in a region implicated from schizophrenia linkage studies, (2) found to be downregulated upon drug treatment, and (3) and advanced into a candidate gene association study in an Asian sample. However, no association was detected between schizophrenia and *ENTPD4* SNPs. | ^131-133^ |
| *HEY1* | *HEY1* is a transcriptional repressor involved in several aspects of neurodevelopment, and for which expression is induced by the Notch and c-Jun signal transduction pathways. *HEY1* was one of two genes with SNPs that were identified as genome-wide significant QTLs (quantitative trait loci) for working memory in a Hispanic family sample. | ^134-138^ |
| *TNFRSF11B* | *TNFRSF11B* (also called *osteoprotegerin*, *OPG*) is an osteoblast-secreted decoy receptor that functions as a negative regulator of bone resorption. *TNFRSF11B* plasma protein levels were elevated in 312 cases (bipolar disorder and schizophrenia) compared to 239 controls. In a sample of 192 schizophrenia cases, there was a weak positive correlation of *TNFRSF11B* plasma protein levels with the number of psychiatric hospitalizations. | ^139, 140^ |
| *ENPP2* | *ENPP2* is also known as *autotaxin* (*ATX*), and is known to be important in a number of neurodevelopmental processes as well as in inflammation. *ENPP2* has also been reported as a putative SSRI response biomarker, downregulated in temporal cortex of depression cases. | ^67, 80, 141^ |
| *ST3GAL1* | *ST3GAL1* catalyzes the transfer of sialic acid from CMP-sialic acid to galactose-containing substrates. SNPs in *ST3GAL1* have been reported as nominally associated (*P*=1.75E-05) with negative symptoms of schizophrenia. *ST3GAL1* SNPs have also been reported to be nominally associated with bipolar disorder. | ^142-144^ |
| *GRINA* | *GRINA*, a glutmate receptor, was among the genes downregulated after 8 weeks of topiramate treament (in a drug trial for methamphetamine dependence), as measured in RNA from blood. Another study on postmortem brain found upregulation of *GRINA* in the prefrontal cortex of major depression cases versus controls. | ^145, 146^ |
| *IFNA2* | *IFNA2* is a cytokine produced in response to viral infection. Treatment with interferons (e.g., for chronic hepatitis C virus infection) is known to produce a high rate of psychiatric side effects (e.g., depression, irritability, mania, psychosis, delirium). | ^147^ |
| *NTRK2* | *NTRK2* is the high affinity receptor for *brain-derived neurotrophic factor* (*BDNF*). Variants in *NTRK2* have been reported to be nominally associated with clozapine response. *NTRK2* has been noted to be downregulated in brains (e.g., prefrontal cortex, hippocampus) of schizophrenic cases versus controls in several studies. Interaction of variants in *NTRK2* and *BDNF* has been proposed to increase risk for schizophrenia. | ^148-152^ |
| *TLR4* | *TLR4* is a member of the Toll-like receptor (TLR) family which plays a fundamental role in pathogen recognition and activation of innate immunity. A recent review postulated involvement of TLRs (especially *TLR4*) in development of schizophrenia through prenatal infection leading to immune effects on neurodevelopment. | ^153, 154^ |
| *ARID5B* | Variants near *ARID5B* were associated with treatment response for haloperidol (*P*=1.36E-06) and perphenazine (*P*=0.015), however, the direction of effect was opposite in the two studies (which is speculated to possibly represent a "flip-flop" effect). | ^155, 156^ |
| *ANXA7* | *ANXA7* is a membrane binding protein with diverse properties, including voltage-sensitive calcium channel activity, ion selectivity and membrane fusion. In a study of schizophrenic cases and controls from Taiwan, *ANXA7* expression was noted to be downregulated in LCLs. | ^157^ |
| *SEC24C* | *SEC24C* is involved in vesicle trafficking. IN a study of protein-protein interaction network of abnormally expressed genes in postmortem brain samples of schizophrenia, bipolar disorder, and major depression patients (versus controls), *SEC24C* was the gene most different between the bipolar disorder and control groups. | ^158^ |
| *FAS* | *FAS* plays a central role in the physiological regulation of programmed cell death. *FAS* has been found to be upregulated in blood as well as the brain (dorsolateral prefrontal cortex) of schizophrenic cases versus controls. Nominal association with treatment resistant schizophrenia has been reported. | ^159-163^ |
| *IFIT2* | *IFIT1*, *IFIT2*, *IFIT3*, and *IFIT5* are interferon-induced proteins with tetratricopeptide repeats located in a chromosome 10q23.31 gene cluster; among other roles, these genes are responsive to viral infections, especially in the central nervous system. Peripubertal infection mimicry in rats (peripheral administration of polyriboinosinic-polyribocytidylic acid) resulted in increased hippocampal expression of *IFIT2* accompanied by an emergent deficit in prepulse inhibition. | ^164-166^ |
| *IFIT3* | see entry for *IFIT2*. | ^166^ |
| *IFIT1* | see entry for *IFIT2*. | ^166^ |
| *IFIT5* | see entry for *IFIT2*. | ^166^ |
| *ADAM8* | *ADAM8* is a member of the ADAM (a disintegrin and metalloprotease domain) family, which includes membrane-anchored proteins involved in a variety of roles including neurogenesis. *ADAM8* may be involved in cell adhesion during neurodegeneration. | ^167-169^ |
| *IFITM1* | See discussion text. | ^170-172^ |
| *IRF7* | Like *IFIT2* (above), *IRF7* was upregulated by peripubertal infection mimicry in rat hippocampus. | ^164^ |
| *ADM* | *ADM* (*adrenomedullin*) is a peptide with several functions, including vasodilation, regulation of hormone secretion, promotion of angiogenesis, and antimicrobial activity. *ADM* has been found to be upregulated in LCLs and in blood from schizophrenic cases, as well as in blood from bipolar disorder cases, versus controls. In a cross-disorder GWAS, the strongest association (*P*~4E−08) was near *ADM*, with the best-fitting model indicating some specificity to bipolar disorder, type 2. Conditonal ADM-knockout mice show abnormalities in activity and anxiety behaviors. | ^173-176^ |
| *SHANK2* | See discussion text. | ^177-182^ |
| *UCP2* | *UCP2* facilitates the transfer of anions from the inner to the outer mitochondrial membrane and the return transfer of protons from the outer to the inner mitochondrial membrane. UCPs have been suggested to exert a neuroprotective effect against increased oxidative stress. *UCP2* was found to be downregulated in prefrontal cortex of bipolar disorder cases and schizophrenia cases compared to controls. | ^183, 184^ |
| *STT3A* | *STT3A* was nominally associated (*P*~1.5E−05) with schizophrenia in a sample from India. | ^185^ |
| *VDR* | *VDR* is the nuclear hormone receptor for vitamin D3 (cholecalciferol). In cell lines, vitamin D3 was found to regulate tyrosine hydroxylase expression, and in other studies to be important in the development of dopaminergic neurons. | ^186^ |
| *PTGES3* | *PTGES3* is a pivotal cofactor of the glucocorticoid receptor. Following up on abnormalities in brain expression of the glucocorticoid receptor (responsive to stress-induced cortisol secretion), *PTGES3* was found to be upregulated in the prefrontal cortex in schizophrenia cases relative to controls. | ^187-189^ |
| *SSH1* | *SSH1* plays a role in actin dynamics by reactivating cofilin proteins. *SSH1* has been shown to be necessary for dopamine D4 receptor regulation of glutamatergic transmission. | ^190, 191^ |
| *FLT1* | *FLT1* is a member of the vascular endothelial growth factor receptor family, and plays an important role in angiogenesis and vasculogenesis. *FLT1* has also been reported as a putative SSRI response biomarker, and has been noted to be involved in hippocampal neurogenesis. | ^67, 192^ |
| *TNFSF13B* | *TNFSF13B* is a cytokine belonging to the tumor necrosis factor ligand family, and plays an important role in the proliferation and differentiation of B cells. DBA/2J mice have less social interaction and reduced pre-pulse inhibition compared to C57BL/6N mice, and also show higher expression of *Tnfsf13b* in the cortex and hypothalamus. *TNFSF13B* was noted to be downregulated in the blood of drug-free acute schizophrenia cases versus controls. | ^57, 193^ |
| *PSMB5* | *PSMB5* is part of a modified proteasome, the immunoproteasome, that processes class I MHC peptides. Lithium treatment was found to downregulate *PSMB5* in the mouse brain. | ^194, 195^ |
| *PSME1* | *PSME1* is part of a modified proteasome, the immunoproteasome, that processes class I MHC peptides, and is also protective against apoptosis induced by oxidative stress. The protein expression of *Psme1* was downregulated in the medial prefrontal cortex of rats treated with phencyclidine (a psychotogenic drug). | ^90, 195, 196^ |
| *ADAM10* | *ADAM10* is a member of the ADAM (a disintegrin and metalloprotease domain) family, which includes membrane-anchored proteins involved in a variety of roles including neurite branching and neurite outgrowth. *ADAM10* contributes to regulation of neural cell adhesion molecule (NCAM) shedding, and NCAM in turn influences axon growth and synaptic plasticity, and has been implicated in schizophrenia. | ^197-200^ |
| *PSMA4* | *PSMA4* is part of a modified proteasome, the immunoproteasome, that processes class I MHC peptides; *PSMA4* is an interaction partner of *dysbindin* (*dystrobrevin-binding protein 1*, or *DTNBP1*) via proteomics analyses. *PSMA4* proteasomal activity was noted to be significantly reduced in brains of the *dysbindin*-null mutant mice (*dysbindin* being extensively studied as a schizophrenia risk gene). | ^195, 201^ |
| *NGRN* | *NGRN* (*neugrin, neurite outgrowth associated*) has been shown to be upregulated with the neurite outgrowth associated with neuronal differentiation in neuroblastoma cell lines. | ^202^ |
| *NDE1* | See discussion text. | ^203-211^ |
| *FUS* | *FUS* is a multifunctional protein component of the heterogeneous nuclear ribonucleoprotein (hnRNP) complex, which is involved in pre-mRNA splicing and the export of fully processed mRNA to the cytoplasm. *FUS* mutations are implicated in familial MND (motor neuron disease) / ALS (amyotrophic lateral sclerosis). A review found that frontotemporal dementia with *FUS* pathology had an increased prevalence of psychosis. | ^212, 213^ |
| *MT2A* | *MT2A* is a member of the metallothioneins, which are reported to be involved in aspects of neuroprotection, regeneration, and cognitive function. *MT2A* was found to be upregulated in postmortem brain (hippocampus, dorsolateral prefrontal cortex) from schizophrenic cases versus controls. | ^172, 214, 215^ |
| *TRAF4* | *TRAF4* is a member of the TRAF family, which mediates signal transduction from members of the TNF receptor superfamily. *TRAF4* plays important roles in neural tube closure and neurodevelopment. *TRAF4* was reported as downregulated in temporal cortex of schizophrenia cases versus controls. | ^216-220^ |
| *CCL5* | *CCL5* belongs to a superfamily of secreted proteins involved in immunoregulatory and inflammatory processes. *CCL5* has also been reported as a putative SSRI response biomarker. *CCL5* may also play various central nervous system roles: neuronal survival, chemotactic activity, neural plasticity, modulation of glutamatergic neurotransmission, and astrocyte-mediated neuronal differentiation. Expression in blood of *CCL5* has been separately reported to be upregulated or downregulated in schizophrenia cases versus controls. More consistently reported in Alzheimer's Disorder cases versus controls, *CCL5* expression is upregulated. | ^67, 221-225^ |
| *RGS9* | *RGS9* is a member of the RGS family of GTPase activating proteins that function in various signaling pathways by accelerating the deactivation of G proteins. *RGS9* plays a role in modulating dopaminergic receptor-mediated signaling cascades. *RGS9* expression is downregulated in an amphetamine-sensitized rat model, as well as in postmortem brain (hippocampus) from schizophrenia cases versus controls. | ^226, 227^ |
| *SSTR2* | *SSTR2* is a G protein-coupled receptor highly expressed in the brain, and which is a receptor for somatostatin, which acts at many sites to inhibit the release of various hormones and other secretory proteins. *SSTR2* expression was upregulated in the amygdala of rats treated with the N-methyl-D-aspartate (NMDA) antagonist, MK-801, (a suggested animal model of schizophrenia), along with the typically noted decreased social interaction and increased locomotor activity. Chronic haloperidol treatment of macaque monkeys led to downregulation of *SSTR2* in the brain (cortex). | ^228, 229^ |
| *B4GALT6* | *B4GALT6* is a lactosylceramide synthase important for glycolipid biosynthesis. *B4GALT6* has been reported to be downregulated in prefrontal cortex of schizophrenia cases compared to controls. | ^79^ |
| *BCL2* | *BCL2* is an integral outer mitochondrial membrane protein that blocks the apoptotic death of lymphocytes. *BCL2* was downregulated in lymphocytes from schizophrenia cases versus controls. Primary fibroblast cultures from antipsychotic-naïve patients with first-episode schizophrenia showed *BCL2* downregulation versus controls. | ^81, 230^ |
| *NETO1* | *NETO1* is a transmembrane protein containing two extracellular CUB domains followed by a low-density lipoprotein class A (LDLa) domain; a similar gene in mice encodes a protein that plays a critical role in spatial learning and memory by regulating the function of synaptic N-methyl-D-aspartic acid receptor complexes in the hippocampus. Deletion of *NETO1* in mice leads to deficits in synaptic plasticity. A SNP near *NETO1* was reported to have nominally significant association (*P*~9E-06) to schizophrenia in a family-based GWAS of 107 Jewish-Israeli families. A variant in an intron of *NETO1* showed nominal association (*P*~1.5E-04) in a large combined (European ancestry and African Americn) GWAS of schizophrenia. Along with other 18q genes (*TCF4*, *FBXO15*), hemizygosity of *NETO1* has been reported to be associated with a autistic-like behaviors. | ^29, 231-234^ |
| *C3* | See discussion text. | ^235-239^ |
| *TGFB1* | See discussion text. | ^240-245^ |
| *BCL2L1* | *BCL2L1* forms hetero- or homodimers and acts as an anti-apoptotic (longer isoform) or pro-apoptotic (shorter isoform) regulator. *BCL2L1* was upregulated in rats (frontal cortex) after treatment with clozapine or haloperidol compared to control rats. Phencyclidine (PCP, a psychotomimetic drug) impaired spatial memory in rats and decreased the ratio of the longer (anti-apoptotic) to the shorter (pro-apoptotic) isoform in the posterior cingulate cortex, effects which were reversed with quetiapine treatment. | ^246-249^ |
| *RPN2* | *RPN2* encodes a type I integral membrane protein found only in the rough endoplasmic reticulum, and is part of an N-oligosaccharyl transferase complex that links high mannose oligosaccharides to asparagine residues found in the Asn-X-Ser/Thr consensus motif of nascent polypeptide chains. *NRG1*-alpha (*NRG1*, *neuregulin*, a schizophrenia risk gene) treatment of LCLs showed a slower chemotactic response for LCLs from schizophrenia cases than from controls, thus prompting a study of which genes *NRG1*-alpha treatment affected: *RPN2* was downregulated in LCLs from schizophrenia cases treated (versus untreated) with NRG1-alpha. | ^250, 251^ |
| *TGM2* | See discussion text. | ^252-254^ |
| *CD40* | *CD40* is a member of the TNF-receptor superfamily, a receptor on antigen-presenting cells of the immune system, and is essential for mediating a broad variety of immune and inflammatory responses (*CD40* is expressed on microglia). The interaction of *CD40* and its ligand is found to be necessary for amyloid-beta-induced microglial activation, which is thought to be an early event in Alzheimer disease pathogenesis. The ligand for *CD40* (*CD40LG*) was negatively associated with general cognitive abilities in bipolar disorder cases. | ^255-257^ |
| *MX2* | *MX2* is a member of both the dynamin family and the family of large GTPases, and is upregulated by interferon-alpha. Peripubertal infection mimicry in rats (peripheral administration of polyriboinosinic-polyribocytidylic acid) resulted in increased hippocampal expression of *Mx2* accompanied by an emergent deficit in prepulse inhibition. | ^164, 258, 259^ |
| *MX1* | *MX1* is a guanosine triphosphate (GTP)-metabolizing protein that participates in the cellular antiviral response, and is induced by type I and type II interferons. *MX1* (and *MX2*) are key mediators of the interferon-induced innate immune response. | ^258-260^ |
| *XBP1* | See discussion text. | ^261-266^ |
| *TCN2* | *TCN2* encodes a member of the vitamin B12-binding protein family, and transports vitamin B12 (cobalamin) to peripheral tissues. Vitamin B12 (along with other B vitamins, e.g., folic acid, vitamin B6) is essential for neuronal function, and severe deficiencies of these vitamins have been linked to increased risk of various psychiatric disorders. Low blood levels of various B vitamins (including B12) have been reported in patients with schizophrenia (including in drug naive first-episode patients), and B12 supplementation (often with other B vitamins) has sometimes shown to help as an add-on treatment of schizophrenia. | ^267, 268^ |
| *SYN1* | *SYN1* (*synapsin I*) encodes a neuronal phosphoprotein, which associates with the cytoplasmic surface of synaptic vesicles. *SYN1* plays a role in regulation of axonogenesis and synaptogenesis. A synonymous SNP (rs1142636) in *SYN1* was reported as associated (*P*~6E-05 in full sample; *P*~1E-04 in females, NS in males) in a candidate gene study of 286 (99 female) schizophrenia cases and 304 (159 female) controls from Korea. A *SynI* knockout mouse model showed evidence of social impairments possibly consistent with those seen in schizophrenia or autism spectrum disorders (ASD). *SYN1* nonsense mutations (W356X, Q555X) have been found to cosegregate in families with epilepsy and ASD phenotypes. A proteomic study of rat brain (frontal cortex and hippocampus) showed decreased *SYN1* after treatment with ketamine. *SYN1* was a node gene in a network analysis of genes whose brain (prefrontal cortex) expression was altered by phencyclidine (PCP) administration (*SYN1* was downregulated) and normalized by olanzapine treatment in macaques. | ^269-276^ |
| Genes above are a highlighted (for potential relevance to schizophrenia) subset of the 1 058 genes with differential expression by schizophrenia status (Bonferroni *P*≤0.05); the full list is given in Table S1. Brief description of gene functions are from the cited references, sometimes supplemented by RefSeq descriptions. | | |

**References.**

1. Vanhaesebroeck B, Welham MJ, Kotani K, Stein R, Warne PH, Zvelebil MJ*, et al*. P110delta, a novel phosphoinositide 3-kinase in leukocytes. *Proc Natl Acad Sci U S A* 1997; **94**(9)**:** 4330-4335.

2. Law AJ, Wang Y, Sei Y, O'Donnell P, Piantadosi P, Papaleo F*, et al*. Neuregulin 1-ErbB4-PI3K signaling in schizophrenia and phosphoinositide 3-kinase-p110delta inhibition as a potential therapeutic strategy. *Proc Natl Acad Sci U S A* 2012; **109**(30)**:** 12165-12170.

3. Eickholt BJ, Ahmed AI, Davies M, Papakonstanti EA, Pearce W, Starkey ML*, et al*. Control of axonal growth and regeneration of sensory neurons by the p110delta PI 3-kinase. *PLoS One* 2007; **2**(9)**:** e869.

4. Levitt P, Campbell DB. The genetic and neurobiologic compass points toward common signaling dysfunctions in autism spectrum disorders. *J Clin Invest* 2009; **119**(4)**:** 747-754.

5. Waite K, Eickholt BJ. The neurodevelopmental implications of PI3K signaling. *Curr Top Microbiol Immunol* 2010; **346:** 245-265.

6. Gross C, Bassell GJ. Neuron-specific regulation of class I PI3K catalytic subunits and their dysfunction in brain disorders. *Front Mol Neurosci* 2014; **7:** 12.

7. Steinlechner S, Stahlberg J, Volkel B, Djarmati A, Hagenah J, Hiller A*, et al*. Co-occurrence of affective and schizophrenia spectrum disorders with PINK1 mutations. *J Neurol Neurosurg Psychiatry* 2007; **78**(5)**:** 532-535.

8. Funayama M, Li Y, Tsoi TH, Lam CW, Ohi T, Yazawa S*, et al*. Familial Parkinsonism with digenic parkin and PINK1 mutations. *Mov Disord* 2008; **23**(10)**:** 1461-1465.

9. Li X, Zhang W, Zhang C, Yi Z, Zhang DF, Gong W*, et al*. Common variants of the PINK1 and PARL genes do not confer genetic susceptibility to schizophrenia in Han Chinese. *Mol Genet Genomics* 2015; **290**(2)**:** 585-592.

10. Yuan P, Zhou R, Wang Y, Li X, Li J, Chen G*, et al*. Altered levels of extracellular signal-regulated kinase signaling proteins in postmortem frontal cortex of individuals with mood disorders and schizophrenia. *J Affect Disord* 2010; **124**(1-2)**:** 164-169.

11. Wong EV, Schaefer AW, Landreth G, Lemmon V. Involvement of p90rsk in neurite outgrowth mediated by the cell adhesion molecule L1. *J Biol Chem* 1996; **271**(30)**:** 18217-18223.

12. Ilia M, Beasley C, Meijer D, Kerwin R, Cotter D, Everall I*, et al*. Expression of Oct-6, a POU III domain transcription factor, in schizophrenia. *Am J Psychiatry* 2002; **159**(7)**:** 1174-1182.

13. Ubhi K, Price J. Expression of POU-domain transcription factor, Oct-6, in schizophrenia, bipolar disorder and major depression. *BMC Psychiatry* 2005; **5:** 38.

14. Riley B, Thiselton D, Maher BS, Bigdeli T, Wormley B, McMichael GO*, et al*. Replication of association between schizophrenia and ZNF804A in the Irish Case-Control Study of Schizophrenia sample. *Mol Psychiatry* 2010; **15**(1)**:** 29-37.

15. Weidenhofer J, Scott RJ, Tooney PA. Investigation of the expression of genes affecting cytomatrix active zone function in the amygdala in schizophrenia: effects of antipsychotic drugs. *J Psychiatr Res* 2009; **43**(3)**:** 282-290.

16. Kumar RA, Sudi J, Babatz TD, Brune CW, Oswald D, Yen M*, et al*. A de novo 1p34.2 microdeletion identifies the synaptic vesicle gene RIMS3 as a novel candidate for autism. *J Med Genet* 2010; **47**(2)**:** 81-90.

17. Nishimura Y, Martin CL, Vazquez-Lopez A, Spence SJ, Alvarez-Retuerto AI, Sigman M*, et al*. Genome-wide expression profiling of lymphoblastoid cell lines distinguishes different forms of autism and reveals shared pathways. *Hum Mol Genet* 2007; **16**(14)**:** 1682-1698.

18. Weidenhofer J, Bowden NA, Scott RJ, Tooney PA. Altered gene expression in the amygdala in schizophrenia: up-regulation of genes located in the cytomatrix active zone. *Mol Cell Neurosci* 2006; **31**(2)**:** 243-250.

19. Wang P, Wu P, Ohleth KM, Egan RW, Billah MM. Phosphodiesterase 4B2 is the predominant phosphodiesterase species and undergoes differential regulation of gene expression in human monocytes and neutrophils. *Mol Pharmacol* 1999; **56**(1)**:** 170-174.

20. Siuciak JA, McCarthy SA, Chapin DS, Martin AN. Behavioral and neurochemical characterization of mice deficient in the phosphodiesterase-4B (PDE4B) enzyme. *Psychopharmacology (Berl)* 2008; **197**(1)**:** 115-126.

21. Azam MA, Tripuraneni NS. Selective Phosphodiesterase 4B Inhibitors: A Review. *Sci Pharm* 2014; **82**(3)**:** 453-481.

22. Millar JK, Pickard BS, Mackie S, James R, Christie S, Buchanan SR*, et al*. DISC1 and PDE4B are interacting genetic factors in schizophrenia that regulate cAMP signaling. *Science* 2005; **310**(5751)**:** 1187-1191.

23. Fatemi SH, King DP, Reutiman TJ, Folsom TD, Laurence JA, Lee S*, et al*. PDE4B polymorphisms and decreased PDE4B expression are associated with schizophrenia. *Schizophr Res* 2008; **101**(1-3)**:** 36-49.

24. Rastogi A, Zai C, Likhodi O, Kennedy JL, Wong AH. Genetic association and post-mortem brain mRNA analysis of DISC1 and related genes in schizophrenia. *Schizophr Res* 2009; **114**(1-3)**:** 39-49.

25. Numata S, Iga J, Nakataki M, Tayoshi S, Taniguchi K, Sumitani S*, et al*. Gene expression and association analyses of the phosphodiesterase 4B (PDE4B) gene in major depressive disorder in the Japanese population. *Am J Med Genet B Neuropsychiatr Genet* 2009; **150B**(4)**:** 527-534.

26. Consortium SWGotPG. Biological insights from 108 schizophrenia-associated genetic loci. *Nature* 2014; **511**(7510)**:** 421-427.

27. Vestal DJ, Jeyaratnam JA. The guanylate-binding proteins: emerging insights into the biochemical properties and functions of this family of large interferon-induced guanosine triphosphatase. *J Interferon Cytokine Res* 2011; **31**(1)**:** 89-97.

28. Hu Y, Wang J, Yang B, Zheng N, Qin M, Ji Y*, et al*. Guanylate binding protein 4 negatively regulates virus-induced type I IFN and antiviral response by targeting IFN regulatory factor 7. *J Immunol* 2011; **187**(12)**:** 6456-6462.

29. Shi J, Levinson DF, Duan J, Sanders AR, Zheng Y, Pe'er I*, et al*. Common variants on chromosome 6p22.1 are associated with schizophrenia. *Nature* 2009; **460**(7256)**:** 753-757.

30. Oksenberg JR, Baranzini SE, Sawcer S, Hauser SL. The genetics of multiple sclerosis: SNPs to pathways to pathogenesis. *Nat Rev Genet* 2008; **9**(7)**:** 516-526.

31. Hafler DA, Compston A, Sawcer S, Lander ES, Daly MJ, De Jager PL*, et al*. Risk alleles for multiple sclerosis identified by a genomewide study. *N Engl J Med* 2007; **357**(9)**:** 851-862.

32. Hoppenbrouwers IA, Aulchenko YS, Ebers GC, Ramagopalan SV, Oostra BA, van Duijn CM*, et al*. EVI5 is a risk gene for multiple sclerosis. *Genes Immun* 2008; **9**(4)**:** 334-337.

33. Mao H, Pilaz LJ, McMahon JJ, Golzio C, Wu D, Shi L*, et al*. Rbm8a haploinsufficiency disrupts embryonic cortical development resulting in microcephaly. *J Neurosci* 2015; **35**(18)**:** 7003-7018.

34. Zou D, McSweeney C, Sebastian A, Reynolds DJ, Dong F, Zhou Y*, et al*. A critical role of RBM8a in proliferation and differentiation of embryonic neural progenitors. *Neural Dev* 2015; **10**(1)**:** 18.

35. Alachkar A, Jiang D, Harrison M, Zhou Y, Chen G, Mao Y. An EJC factor RBM8a regulates anxiety behaviors. *Curr Mol Med* 2013; **13**(6)**:** 887-899.

36. Stefansson H, Rujescu D, Cichon S, Pietilainen OP, Ingason A, Steinberg S*, et al*. Large recurrent microdeletions associated with schizophrenia. *Nature* 2008; **455**(7210)**:** 232-236.

37. Levinson DF, Duan J, Oh S, Wang K, Sanders AR, Shi J*, et al*. Copy Number Variants in Schizophrenia: Confirmation of Five Previous Findings and New Evidence for 3q29 Microdeletions and VIPR2 Duplications. *Am J Psychiatry* 2011; **168**(3)**:** 302-316.

38. Sun S, Wang F, Wei J, Cao LY, Qi LY, Xiu MH*, et al*. Association between interleukin-6 receptor polymorphism and patients with schizophrenia. *Schizophr Res* 2008; **102**(1-3)**:** 346-347.

39. Hsiao EY, Patterson PH. Activation of the maternal immune system induces endocrine changes in the placenta via IL-6. *Brain Behav Immun* 2011; **25**(4)**:** 604-615.

40. Lill CM, Roehr JT, McQueen MB, Kavvoura FK, Bagade S, Schjeide BM*, et al*. Comprehensive research synopsis and systematic meta-analyses in Parkinson's disease genetics: The PDGene database. *PLoS Genet* 2012; **8**(3)**:** e1002548.

41. Liou YJ, Wang HH, Lee MT, Wang SC, Chiang HL, Chen CC*, et al*. Genome-wide association study of treatment refractory schizophrenia in Han Chinese. *PLoS One* 2012; **7**(3)**:** e33598.

42. Ma Y, Mi YJ, Dai YK, Fu HL, Cui DX, Jin WL. The inverse F-BAR domain protein srGAP2 acts through srGAP3 to modulate neuronal differentiation and neurite outgrowth of mouse neuroblastoma cells. *PLoS One* 2013; **8**(3)**:** e57865.

43. Charrier C, Joshi K, Coutinho-Budd J, Kim JE, Lambert N, de Marchena J*, et al*. Inhibition of SRGAP2 function by its human-specific paralogs induces neoteny during spine maturation. *Cell* 2012; **149**(4)**:** 923-935.

44. Saitsu H, Osaka H, Sugiyama S, Kurosawa K, Mizuguchi T, Nishiyama K*, et al*. Early infantile epileptic encephalopathy associated with the disrupted gene encoding Slit-Robo Rho GTPase activating protein 2 (SRGAP2). *Am J Med Genet A* 2012; **158A**(1)**:** 199-205.

45. Arakelyan A, Zakharyan R, Khoyetsyan A, Poghosyan D, Aroutiounian R, Mrazek F*, et al*. Functional characterization of the complement receptor type 1 and its circulating ligands in patients with schizophrenia. *BMC Clin Pathol* 2011; **11:** 10.

46. Liu D, Niu ZX. The structure, genetic polymorphisms, expression and biological functions of complement receptor type 1 (CR1/CD35). *Immunopharmacol Immunotoxicol* 2009; **31**(4)**:** 524-535.

47. Lambert JC, Heath S, Even G, Campion D, Sleegers K, Hiltunen M*, et al*. Genome-wide association study identifies variants at CLU and CR1 associated with Alzheimer's disease. *Nat Genet* 2009; **41**(10)**:** 1094-1099.

48. Lambert JC, Ibrahim-Verbaas CA, Harold D, Naj AC, Sims R, Bellenguez C*, et al*. Meta-analysis of 74,046 individuals identifies 11 new susceptibility loci for Alzheimer's disease. *Nat Genet* 2013; **45**(12)**:** 1452-1458.

49. Drexhage RC, van der Heul-Nieuwenhuijsen L, Padmos RC, van Beveren N, Cohen D, Versnel MA*, et al*. Inflammatory gene expression in monocytes of patients with schizophrenia: overlap and difference with bipolar disorder. A study in naturalistically treated patients. *Int J Neuropsychopharmacol* 2010; **13**(10)**:** 1369-1381.

50. Steinberg S, de Jong S, Andreassen OA, Werge T, Borglum AD, Mors O*, et al*. Common variants at VRK2 and TCF4 conferring risk of schizophrenia. *Hum Mol Genet* 2011; **20**(20)**:** 4076-4081.

51. Li LY, Liu MY, Shih HM, Tsai CH, Chen JY. Human cellular protein VRK2 interacts specifically with Epstein-Barr virus BHRF1, a homologue of Bcl-2, and enhances cell survival. *J Gen Virol* 2006; **87**(Pt 10)**:** 2869-2878.

52. Li M, Wang Y, Zheng XB, Ikeda M, Iwata N, Luo XJ*, et al*. Meta-analysis and brain imaging data support the involvement of VRK2 (rs2312147) in schizophrenia susceptibility. *Schizophr Res* 2012; **142**(1-3)**:** 200-205.

53. Sohn H, Kim B, Kim KH, Kim MK, Choi TK, Lee SH. Effects of VRK2 (rs2312147) on white matter connectivity in patients with schizophrenia. *PLoS One* 2014; **9**(7)**:** e103519.

54. Shi J, Gershon ES, Liu C. Genetic associations with schizophrenia: meta-analyses of 12 candidate genes. *Schizophr Res* 2008; **104**(1-3)**:** 96-107.

55. Xu M, He L. Convergent evidence shows a positive association of interleukin-1 gene complex locus with susceptibility to schizophrenia in the Caucasian population. *Schizophr Res* 2010; **120**(1-3)**:** 131-142.

56. Shirts BH, Wood J, Yolken RH, Nimgaonkar VL. Association study of IL10, IL1beta, and IL1RN and schizophrenia using tag SNPs from a comprehensive database: suggestive association with rs16944 at IL1beta. *Schizophr Res* 2006; **88**(1-3)**:** 235-244.

57. Ma L, Kulesskaya N, Voikar V, Tian L. Differential expression of brain immune genes and schizophrenia-related behavior in C57BL/6N and DBA/2J female mice. *Psychiatry Res* 2015; **226**(1)**:** 211-216.

58. Liou YJ, Bai YM, Lin E, Chen JY, Chen TT, Hong CJ*, et al*. Gene-gene interactions of the INSIG1 and INSIG2 in metabolic syndrome in schizophrenic patients treated with atypical antipsychotics. *Pharmacogenomics J* 2012; **12**(1)**:** 54-61.

59. Lett TA, Wallace TJ, Chowdhury NI, Tiwari AK, Kennedy JL, Muller DJ. Pharmacogenetics of antipsychotic-induced weight gain: review and clinical implications. *Mol Psychiatry* 2012; **17**(3)**:** 242-266.

60. Kuo PH, Kao CF, Chen PY, Chen CH, Tsai YS, Lu ML*, et al*. Polymorphisms of INSIG2, MC4R, and LEP are associated with obesity- and metabolic-related traits in schizophrenic patients. *J Clin Psychopharmacol* 2011; **31**(6)**:** 705-711.

61. Seki Y, Kato TA, Monji A, Mizoguchi Y, Horikawa H, Sato-Kasai M*, et al*. Pretreatment of aripiprazole and minocycline, but not haloperidol, suppresses oligodendrocyte damage from interferon-gamma-stimulated microglia in co-culture model. *Schizophr Res* 2013; **151**(1-3)**:** 20-28.

62. Shaltout TE, Alali KA, Bushra S, Alkaseri AM, Jose ED, Al-Khainji M*, et al*. Significant association of close homologue of L1 gene polymorphism rs2272522 with schizophrenia in Qatar. *Asia Pac Psychiatry* 2013; **5**(1)**:** 17-23.

63. Andreyeva A, Leshchyns'ka I, Knepper M, Betzel C, Redecke L, Sytnyk V*, et al*. CHL1 is a selective organizer of the presynaptic machinery chaperoning the SNARE complex. *PLoS One* 2010; **5**(8)**:** e12018.

64. Salyakina D, Cukier HN, Lee JM, Sacharow S, Nations LD, Ma D*, et al*. Copy number variants in extended autism spectrum disorder families reveal candidates potentially involved in autism risk. *PLoS One* 2011; **6**(10)**:** e26049.

65. Frints SG, Marynen P, Hartmann D, Fryns JP, Steyaert J, Schachner M*, et al*. CALL interrupted in a patient with non-specific mental retardation: gene dosage-dependent alteration of murine brain development and behavior. *Hum Mol Genet* 2003; **12**(13)**:** 1463-1474.

66. Tam GW, van de Lagemaat LN, Redon R, Strathdee KE, Croning MD, Malloy MP*, et al*. Confirmed rare copy number variants implicate novel genes in schizophrenia. *Biochem Soc Trans* 2010; **38**(2)**:** 445-451.

67. Morag A, Pasmanik-Chor M, Oron-Karni V, Rehavi M, Stingl JC, Gurwitz D. Genome-wide expression profiling of human lymphoblastoid cell lines identifies CHL1 as a putative SSRI antidepressant response biomarker. *Pharmacogenomics* 2011; **12**(2)**:** 171-184.

68. Haram M, Tesli M, Bettella F, Djurovic S, Andreassen OA, Melle I. Association between Genetic Variation in the Oxytocin Receptor Gene and Emotional Withdrawal, but not between Oxytocin Pathway Genes and Diagnosis in Psychotic Disorders. *Front Hum Neurosci* 2015; **9:** 9.

69. Davis MC, Horan WP, Nurmi EL, Rizzo S, Li W, Sugar CA*, et al*. Associations between oxytocin receptor genotypes and social cognitive performance in individuals with schizophrenia. *Schizophr Res* 2014; **159**(2-3)**:** 353-357.

70. Montag C, Brockmann EM, Lehmann A, Muller DJ, Rujescu D, Gallinat J. Association between oxytocin receptor gene polymorphisms and self-rated 'empathic concern' in schizophrenia. *PLoS One* 2012; **7**(12)**:** e51882.

71. Montag C, Brockmann EM, Bayerl M, Rujescu D, Muller DJ, Gallinat J. Oxytocin and oxytocin receptor gene polymorphisms and risk for schizophrenia: a case-control study. *World J Biol Psychiatry* 2013; **14**(7)**:** 500-508.

72. Watanabe Y, Kaneko N, Nunokawa A, Shibuya M, Egawa J, Someya T. Oxytocin receptor (OXTR) gene and risk of schizophrenia: case-control and family-based analyses and meta-analysis in a Japanese population. *Psychiatry Clin Neurosci* 2012; **66**(7)**:** 622.

73. Arion D, Lewis DA. Altered expression of regulators of the cortical chloride transporters NKCC1 and KCC2 in schizophrenia. *Arch Gen Psychiatry* 2011; **68**(1)**:** 21-31.

74. Rasmussen HB, Timm S, Wang AG, Soeby K, Lublin H, Fenger M*, et al*. Association between the CCR5 32-bp deletion allele and late onset of schizophrenia. *Am J Psychiatry* 2006; **163**(3)**:** 507-511.

75. Matsumoto A, Ohta N, Goto Y, Kashiwa Y, Yamamoto S, Fujino Y. Haloperidol suppresses murine dendritic cell maturation and priming of the T helper 1-type immune response. *Anesth Analg* 2015; **120**(4)**:** 895-902.

76. Park CH, Kang JS, Yoon EH, Shim JW, Suh-Kim H, Lee SH. Proneural bHLH neurogenin 2 differentially regulates Nurr1-induced dopamine neuron differentiation in rat and mouse neural precursor cells in vitro. *FEBS Lett* 2008; **582**(5)**:** 537-542.

77. Forrest MP, Waite AJ, Martin-Rendon E, Blake DJ. Knockdown of human TCF4 affects multiple signaling pathways involved in cell survival, epithelial to mesenchymal transition and neuronal differentiation. *PLoS One* 2013; **8**(8)**:** e73169.

78. Stefansson H, Ophoff RA, Steinberg S, Andreassen OA, Cichon S, Rujescu D*, et al*. Common variants conferring risk of schizophrenia. *Nature* 2009; **460**(7256)**:** 744-747.

79. Narayan S, Head SR, Gilmartin TJ, Dean B, Thomas EA. Evidence for disruption of sphingolipid metabolism in schizophrenia. *J Neurosci Res* 2009; **87**(1)**:** 278-288.

80. Aston C, Jiang L, Sokolov BP. Transcriptional profiling reveals evidence for signaling and oligodendroglial abnormalities in the temporal cortex from patients with major depressive disorder. *Mol Psychiatry* 2005; **10**(3)**:** 309-322.

81. Gasso P, Mas S, Molina O, Lafuente A, Bernardo M, Parellada E. Increased susceptibility to apoptosis in cultured fibroblasts from antipsychotic-naive first-episode schizophrenia patients. *J Psychiatr Res* 2014; **48**(1)**:** 94-101.

82. Kerns D, Vong GS, Barley K, Dracheva S, Katsel P, Casaccia P*, et al*. Gene expression abnormalities and oligodendrocyte deficits in the internal capsule in schizophrenia. *Schizophr Res* 2010; **120**(1-3)**:** 150-158.

83. Ferno J, Raeder MB, Vik-Mo AO, Skrede S, Glambek M, Tronstad KJ*, et al*. Antipsychotic drugs activate SREBP-regulated expression of lipid biosynthetic genes in cultured human glioma cells: a novel mechanism of action? *Pharmacogenomics J* 2005; **5**(5)**:** 298-304.

84. Barbosa AC, Kim MS, Ertunc M, Adachi M, Nelson ED, McAnally J*, et al*. MEF2C, a transcription factor that facilitates learning and memory by negative regulation of synapse numbers and function. *Proc Natl Acad Sci U S A* 2008; **105**(27)**:** 9391-9396.

85. Kalmady SV, Venkatasubramanian G, Arasappa R, Rao NP. Evolutionary genetic analyses of MEF2C gene: implications for learning and memory in Homo sapiens. *Asian J Psychiatr* 2013; **6**(1)**:** 56-59.

86. Le Meur N, Holder-Espinasse M, Jaillard S, Goldenberg A, Joriot S, Amati-Bonneau P*, et al*. MEF2C haploinsufficiency caused by either microdeletion of the 5q14.3 region or mutation is responsible for severe mental retardation with stereotypic movements, epilepsy and/or cerebral malformations. *J Med Genet* 2010; **47**(1)**:** 22-29.

87. Zweier M, Rauch A. The MEF2C-Related and 5q14.3q15 Microdeletion Syndrome. *Mol Syndromol* 2012; **2**(3-5)**:** 164-170.

88. Leighton PA, Mitchell KJ, Goodrich LV, Lu X, Pinson K, Scherz P*, et al*. Defining brain wiring patterns and mechanisms through gene trapping in mice. *Nature* 2001; **410**(6825)**:** 174-179.

89. Runker AE, O'Tuathaigh C, Dunleavy M, Morris DW, Little GE, Corvin AP*, et al*. Mutation of Semaphorin-6A disrupts limbic and cortical connectivity and models neurodevelopmental psychopathology. *PLoS One* 2011; **6**(11)**:** e26488.

90. Pickering C, Ericson M, Soderpalm B. Chronic phencyclidine increases synapsin-1 and synaptic adaptation proteins in the medial prefrontal cortex. *ISRN Psychiatry* 2013; **2013:** 620361.

91. Groleau SE, Lubarda J, Thomas N, Ferro MA, Pristupa ZB, Mishra RK*, et al*. Human blood analysis reveals differences in gene expression of catecholamine-regulated protein 40 (CRP40) in schizophrenia. *Schizophr Res* 2013; **143**(1)**:** 203-206.

92. Gabriele JP, Chong VZ, Pontoriero GF, Mishra RK. Decreased expression of a 40-kDa catecholamine-regulated protein in the ventral striatum of schizophrenic brain specimens from the Stanley Foundation Neuropathology Consortium. *Schizophr Res* 2005; **74**(1)**:** 111-119.

93. Belkaya S, Murray SE, Eitson JL, de la Morena MT, Forman JA, van Oers NS. Transgenic expression of microRNA-185 causes a developmental arrest of T cells by targeting multiple genes including Mzb1. *J Biol Chem* 2013; **288**(42)**:** 30752-30762.

94. Oyagi A, Oida Y, Kakefuda K, Shimazawa M, Shioda N, Moriguchi S*, et al*. Generation and characterization of conditional heparin-binding EGF-like growth factor knockout mice. *PLoS One* 2009; **4**(10)**:** e7461.

95. Farkas LM, Krieglstein K. Heparin-binding epidermal growth factor-like growth factor (HB-EGF) regulates survival of midbrain dopaminergic neurons. *J Neural Transm* 2002; **109**(3)**:** 267-277.

96. Kornblum HI, Zurcher SD, Werb Z, Derynck R, Seroogy KB. Multiple trophic actions of heparin-binding epidermal growth factor (HB-EGF) in the central nervous system. *Eur J Neurosci* 1999; **11**(9)**:** 3236-3246.

97. Mundel P, Heid HW, Mundel TM, Kruger M, Reiser J, Kriz W. Synaptopodin: an actin-associated protein in telencephalic dendrites and renal podocytes. *J Cell Biol* 1997; **139**(1)**:** 193-204.

98. Focking M, Lopez LM, English JA, Dicker P, Wolff A, Brindley E*, et al*. Proteomic and genomic evidence implicates the postsynaptic density in schizophrenia. *Mol Psychiatry* 2015; **20**(4)**:** 424-432.

99. Schubert KO, Focking M, Prehn JH, Cotter DR. Hypothesis review: are clathrin-mediated endocytosis and clathrin-dependent membrane and protein trafficking core pathophysiological processes in schizophrenia and bipolar disorder? *Mol Psychiatry* 2012; **17**(7)**:** 669-681.

100. Pimm J, McQuillin A, Thirumalai S, Lawrence J, Quested D, Bass N*, et al*. The Epsin 4 gene on chromosome 5q, which encodes the clathrin-associated protein enthoprotin, is involved in the genetic susceptibility to schizophrenia. *Am J Hum Genet* 2005; **76**(5)**:** 902-907.

101. Tang RQ, Zhao XZ, Shi YY, Tang W, Gu NF, Feng GY*, et al*. Family-based association study of Epsin 4 and Schizophrenia. *Mol Psychiatry* 2006; **11**(4)**:** 395-399.

102. Escamilla M, Lee BD, Ontiveros A, Raventos H, Nicolini H, Mendoza R*, et al*. The epsin 4 gene is associated with psychotic disorders in families of Latin American origin. *Schizophr Res* 2008; **106**(2-3)**:** 253-257.

103. Liou YJ, Lai IC, Wang YC, Bai YM, Lin CC, Lin CY*, et al*. Genetic analysis of the human ENTH (Epsin 4) gene and schizophrenia. *Schizophr Res* 2006; **84**(2-3)**:** 236-243.

104. Richards M, Iijima Y, Shizuno T, Kamegaya Y, Hori H, Omori M*, et al*. Failure to confirm an association between Epsin 4 and schizophrenia in a Japanese population. *J Neural Transm* 2008; **115**(9)**:** 1347-1354.

105. Gurling H, Pimm J, McQuillin A. Replication of genetic association studies between markers at the Epsin 4 gene locus and schizophrenia in two Han Chinese samples. *Schizophr Res* 2007; **89**(1-3)**:** 357-359.

106. Leon CA, Schumacher J, Kluck N, Herold C, Schulze TG, Propping P*, et al*. Association study of the GRIA1 and CLINT1 (Epsin 4) genes in a German schizophrenia sample. *Psychiatr Genet* 2011; **21**(2)**:** 114.

107. Sharp SI, Hu Y, Weymer JF, Rizig M, McQuillin A, Hunt SP*, et al*. The effect of clozapine on mRNA expression for genes encoding G protein-coupled receptors and the protein components of clathrin-mediated endocytosis. *Psychiatr Genet* 2013; **23**(4)**:** 153-162.

108. Masuho I, Mototani Y, Sahara Y, Asami J, Nakamura S, Kozasa T*, et al*. Dynamic expression patterns of G protein-regulated inducer of neurite outgrowth 1 (GRIN1) and its colocalization with Galphao implicate significant roles of Galphao-GRIN1 signaling in nervous system. *Dev Dyn* 2008; **237**(9)**:** 2415-2429.

109. Nakata H, Kozasa T. Functional characterization of Galphao signaling through G protein-regulated inducer of neurite outgrowth 1. *Mol Pharmacol* 2005; **67**(3)**:** 695-702.

110. Nordman JC, Phillips WS, Kodama N, Clark SG, Del Negro CA, Kabbani N. Axon targeting of the alpha 7 nicotinic receptor in developing hippocampal neurons by Gprin1 regulates growth. *J Neurochem* 2014; **129**(4)**:** 649-662.

111. Thornalley PJ. Glyoxalase I--structure, function and a critical role in the enzymatic defence against glycation. *Biochem Soc Trans* 2003; **31**(Pt 6)**:** 1343-1348.

112. Toyosima M, Maekawa M, Toyota T, Iwayama Y, Arai M, Ichikawa T*, et al*. Schizophrenia with the 22q11.2 deletion and additional genetic defects: case history. *Br J Psychiatry* 2011; **199**(3)**:** 245-246.

113. Barua M, Jenkins EC, Chen W, Kuizon S, Pullarkat RK, Junaid MA. Glyoxalase I polymorphism rs2736654 causing the Ala111Glu substitution modulates enzyme activity--implications for autism. *Autism Res* 2011; **4**(4)**:** 262-270.

114. Kurz A, Rabbani N, Walter M, Bonin M, Thornalley P, Auburger G*, et al*. Alpha-synuclein deficiency leads to increased glyoxalase I expression and glycation stress. *Cell Mol Life Sci* 2011; **68**(4)**:** 721-733.

115. Bangel FN, Yamada K, Arai M, Iwayama Y, Balan S, Toyota T*, et al*. Genetic analysis of the glyoxalase system in schizophrenia. *Prog Neuropsychopharmacol Biol Psychiatry* 2015; **59:** 105-110.

116. Itokawa M, Miyashita M, Arai M, Miyata T. Carbonyl stress in schizophrenia. *Biochem Soc Trans* 2014; **42**(2)**:** 468-472.

117. Distler MG, Palmer AA. Role of Glyoxalase 1 (Glo1) and methylglyoxal (MG) in behavior: recent advances and mechanistic insights. *Front Genet* 2012; **3:** 250.

118. Arai M, Yuzawa H, Nohara I, Ohnishi T, Obata N, Iwayama Y*, et al*. Enhanced carbonyl stress in a subpopulation of schizophrenia. *Arch Gen Psychiatry* 2010; **67**(6)**:** 589-597.

119. Shan D, Haroutunian V, Meador-Woodruff JH, McCullumsmith RE. Expression of equilibrative nucleoside transporter type 1 protein in elderly patients with schizophrenia. *Neuroreport* 2012; **23**(4)**:** 224-227.

120. Baldwin SA, Beal PR, Yao SY, King AE, Cass CE, Young JD. The equilibrative nucleoside transporter family, SLC29. *Pflugers Arch* 2004; **447**(5)**:** 735-743.

121. Wu J, Lee MR, Choi S, Kim T, Choi DS. ENT1 regulates ethanol-sensitive EAAT2 expression and function in astrocytes. *Alcohol Clin Exp Res* 2010; **34**(6)**:** 1110-1117.

122. Lang F, Strutz-Seebohm N, Seebohm G, Lang UE. Significance of SGK1 in the regulation of neuronal function. *J Physiol* 2010; **588**(Pt 18)**:** 3349-3354.

123. Shin JH, Guedj F, Delabar JM, Lubec G. Dysregulation of growth factor receptor-bound protein 2 and fascin in hippocampus of mice polytransgenic for chromosome 21 structures. *Hippocampus* 2007; **17**(12)**:** 1180-1192.

124. Allen JA, Yadav PN, Setola V, Farrell M, Roth BL. Schizophrenia risk gene CAV1 is both pro-psychotic and required for atypical antipsychotic drug actions in vivo. *Transl Psychiatry* 2011; **1:** e33.

125. Ostrom RS. New determinants of receptor-effector coupling: trafficking and compartmentation in membrane microdomains. *Mol Pharmacol* 2002; **61**(3)**:** 473-476.

126. Allen JA, Halverson-Tamboli RA, Rasenick MM. Lipid raft microdomains and neurotransmitter signalling. *Nat Rev Neurosci* 2007; **8**(2)**:** 128-140.

127. Walsh T, McClellan JM, McCarthy SE, Addington AM, Pierce SB, Cooper GM*, et al*. Rare structural variants disrupt multiple genes in neurodevelopmental pathways in schizophrenia. *Science* 2008; **320**(5875)**:** 539-543.

128. Zhang H, Li D, Su Y, Jiang S, Xu Y, Jiang K*, et al*. Identification of the N-acylsphingosine amidohydrolase 1 gene (ASAH1) for susceptibility to schizophrenia in a Han Chinese population. *World J Biol Psychiatry* 2012; **13**(2)**:** 106-113.

129. Schwarz A, Futerman AH. Distinct roles for ceramide and glucosylceramide at different stages of neuronal growth. *J Neurosci* 1997; **17**(9)**:** 2929-2938.

130. Zhang Z, Mandal AK, Mital A, Popescu N, Zimonjic D, Moser A*, et al*. Human acid ceramidase gene: novel mutations in Farber disease. *Mol Genet Metab* 2000; **70**(4)**:** 301-309.

131. Saito A, Fujikura-Ouchi Y, Ito C, Matsuoka H, Shimoda K, Akiyama K. An association study on polymorphisms in the PEA15, ENTPD4, and GAS2L1 genes and schizophrenia. *Psychiatry Res* 2011; **185**(1-2)**:** 9-15.

132. Biederbick A, Rose S, Elsasser HP. A human intracellular apyrase-like protein, LALP70, localizes to lysosomal/autophagic vacuoles. *J Cell Sci* 1999; **112 ( Pt 15):** 2473-2484.

133. Ouchi Y, Kubota Y, Kuramasu A, Watanabe T, Ito C. Gene expression profiling in whole cerebral cortices of phencyclidine- or methamphetamine-treated rats. *Brain Res Mol Brain Res* 2005; **140**(1-2)**:** 142-149.

134. Knowles EE, Carless MA, de Almeida MA, Curran JE, McKay DR, Sprooten E*, et al*. Genome-wide significant localization for working and spatial memory: Identifying genes for psychosis using models of cognition. *Am J Med Genet B Neuropsychiatr Genet* 2014; **165B**(1)**:** 84-95.

135. Bray S, Bernard F. Notch targets and their regulation. *Curr Top Dev Biol* 2010; **92:** 253-275.

136. Kokubo H, Lun Y, Johnson RL. Identification and expression of a novel family of bHLH cDNAs related to Drosophila hairy and enhancer of split. *Biochem Biophys Res Commun* 1999; **260**(2)**:** 459-465.

137. Leimeister C, Externbrink A, Klamt B, Gessler M. Hey genes: a novel subfamily of hairy- and Enhancer of split related genes specifically expressed during mouse embryogenesis. *Mech Dev* 1999; **85**(1-2)**:** 173-177.

138. Sakamoto M, Hirata H, Ohtsuka T, Bessho Y, Kageyama R. The basic helix-loop-helix genes Hesr1/Hey1 and Hesr2/Hey2 regulate maintenance of neural precursor cells in the brain. *J Biol Chem* 2003; **278**(45)**:** 44808-44815.

139. Hope S, Melle I, Aukrust P, Agartz I, Lorentzen S, Steen NE*, et al*. Osteoprotegerin levels in patients with severe mental disorders. *J Psychiatry Neurosci* 2010; **35**(5)**:** 304-310.

140. Hope S, Ueland T, Steen NE, Dieset I, Lorentzen S, Berg AO*, et al*. Interleukin 1 receptor antagonist and soluble tumor necrosis factor receptor 1 are associated with general severity and psychotic symptoms in schizophrenia and bipolar disorder. *Schizophr Res* 2013; **145**(1-3)**:** 36-42.

141. Benesch MG, Ko YM, McMullen TP, Brindley DN. Autotaxin in the crosshairs: taking aim at cancer and other inflammatory conditions. *FEBS Lett* 2014; **588**(16)**:** 2712-2727.

142. Xu C, Aragam N, Li X, Villla EC, Wang L, Briones D*, et al*. BCL9 and C9orf5 are associated with negative symptoms in schizophrenia: meta-analysis of two genome-wide association studies. *PLoS One* 2013; **8**(1)**:** e51674.

143. Zhang P, Xiang N, Chen Y, Sliwerska E, McInnis MG, Burmeister M*, et al*. Family-based association analysis to finemap bipolar linkage peak on chromosome 8q24 using 2,500 genotyped SNPs and 15,000 imputed SNPs. *Bipolar Disord* 2010; **12**(8)**:** 786-792.

144. Perlis RH, Purcell S, Fagerness J, Kirby A, Petryshen TL, Fan J*, et al*. Family-based association study of lithium-related and other candidate genes in bipolar disorder. *Arch Gen Psychiatry* 2008; **65**(1)**:** 53-61.

145. Li MD, Wang J, Niu T, Ma JZ, Seneviratne C, Ait-Daoud N*, et al*. Transcriptome profiling and pathway analysis of genes expressed differentially in participants with or without a positive response to topiramate treatment for methamphetamine addiction. *BMC Med Genomics* 2014; **7:** 65.

146. Goswami DB, Jernigan CS, Chandran A, Iyo AH, May WL, Austin MC*, et al*. Gene expression analysis of novel genes in the prefrontal cortex of major depressive disorder subjects. *Prog Neuropsychopharmacol Biol Psychiatry* 2013; **43:** 126-133.

147. Raison CL, Demetrashvili M, Capuron L, Miller AH. Neuropsychiatric adverse effects of interferon-alpha: recognition and management. *CNS Drugs* 2005; **19**(2)**:** 105-123.

148. Mitjans M, Catalan R, Vazquez M, Gonzalez-Rodriguez A, Penades R, Pons A*, et al*. Hypothalamic-pituitary-adrenal system, neurotrophic factors and clozapine response: association with FKBP5 and NTRK2 genes. *Pharmacogenet Genomics* 2015; **25**(5)**:** 274-277.

149. Weickert CS, Ligons DL, Romanczyk T, Ungaro G, Hyde TM, Herman MM*, et al*. Reductions in neurotrophin receptor mRNAs in the prefrontal cortex of patients with schizophrenia. *Mol Psychiatry* 2005; **10**(7)**:** 637-650.

150. Hashimoto T, Bergen SE, Nguyen QL, Xu B, Monteggia LM, Pierri JN*, et al*. Relationship of brain-derived neurotrophic factor and its receptor TrkB to altered inhibitory prefrontal circuitry in schizophrenia. *J Neurosci* 2005; **25**(2)**:** 372-383.

151. Thompson Ray M, Weickert CS, Wyatt E, Webster MJ. Decreased BDNF, trkB-TK+ and GAD67 mRNA expression in the hippocampus of individuals with schizophrenia and mood disorders. *J Psychiatry Neurosci* 2011; **36**(3)**:** 195-203.

152. Lin Z, Su Y, Zhang C, Xing M, Ding W, Liao L*, et al*. The interaction of BDNF and NTRK2 gene increases the susceptibility of paranoid schizophrenia. *PLoS One* 2013; **8**(9)**:** e74264.

153. Venkatasubramanian G, Debnath M. The TRIPS (Toll-like receptors in immuno-inflammatory pathogenesis) Hypothesis: a novel postulate to understand schizophrenia. *Prog Neuropsychopharmacol Biol Psychiatry* 2013; **44:** 301-311.

154. Lucas K, Maes M. Role of the Toll Like receptor (TLR) radical cycle in chronic inflammation: possible treatments targeting the TLR4 pathway. *Mol Neurobiol* 2013; **48**(1)**:** 190-204.

155. Drago A, Giegling I, Schafer M, Hartmann AM, Konte B, Friedl M*, et al*. Genome-wide association study supports the role of the immunological system and of the neurodevelopmental processes in response to haloperidol treatment. *Pharmacogenet Genomics* 2014; **24**(6)**:** 314-319.

156. Lin PI, Vance JM, Pericak-Vance MA, Martin ER. No gene is an island: the flip-flop phenomenon. *Am J Hum Genet* 2007; **80**(3)**:** 531-538.

157. Liu CM, Fann CS, Chen CY, Liu YL, Oyang YJ, Yang WC*, et al*. ANXA7, PPP3CB, DNAJC9, and ZMYND17 genes at chromosome 10q22 associated with the subgroup of schizophrenia with deficits in attention and executive function. *Biol Psychiatry* 2011; **70**(1)**:** 51-58.

158. Lee SA, Tsao TT, Yang KC, Lin H, Kuo YL, Hsu CH*, et al*. Construction and analysis of the protein-protein interaction networks for schizophrenia, bipolar disorder, and major depression. *BMC Bioinformatics* 2011; **12 Suppl 13:** S20.

159. Djordjevic VV, Ristic T, Lazarevic D, Cosic V, Vlahovic P, Djordjevic VB. Schizophrenia is associated with increased levels of serum Fas and FasL. *Clin Chem Lab Med* 2012; **50**(6)**:** 1049-1054.

160. Catts VS, Shannon Weickert C. Gene expression analysis implicates a death receptor pathway in schizophrenia pathology. *PLoS ONE* 2012; **7**(4)**:** e35511.

161. Zuliani C, Kleber S, Klussmann S, Wenger T, Kenzelmann M, Schreglmann N*, et al*. Control of neuronal branching by the death receptor CD95 (Fas/Apo-1). *Cell Death Differ* 2006; **13**(1)**:** 31-40.

162. Reich A, Spering C, Schulz JB. Death receptor Fas (CD95) signaling in the central nervous system: tuning neuroplasticity? *Trends Neurosci* 2008; **31**(9)**:** 478-486.

163. Jia P, Jayathilake K, Zhao Z, Meltzer HY. Association of FAS, a TNF-alpha receptor gene, with treatment resistant schizophrenia. *Schizophr Res* 2011; **129**(2-3)**:** 211-212.

164. Lukasz B, O'Sullivan NC, Loscher JS, Pickering M, Regan CM, Murphy KJ. Peripubertal viral-like challenge and social isolation mediate overlapping but distinct effects on behaviour and brain interferon regulatory factor 7 expression in the adult Wistar rat. *Brain Behav Immun* 2013; **27**(1)**:** 71-79.

165. Jiang L, Saetre P, Radomska KJ, Jazin E, Lindholm Carlstrom E. QKI-7 regulates expression of interferon-related genes in human astrocyte glioma cells. *PLoS One* 2010; **5**(9).

166. Wacher C, Muller M, Hofer MJ, Getts DR, Zabaras R, Ousman SS*, et al*. Coordinated regulation and widespread cellular expression of interferon-stimulated genes (ISG) ISG-49, ISG-54, and ISG-56 in the central nervous system after infection with distinct viruses. *J Virol* 2007; **81**(2)**:** 860-871.

167. White JM. ADAMs: modulators of cell-cell and cell-matrix interactions. *Curr Opin Cell Biol* 2003; **15**(5)**:** 598-606.

168. Naus S, Richter M, Wildeboer D, Moss M, Schachner M, Bartsch JW. Ectodomain shedding of the neural recognition molecule CHL1 by the metalloprotease-disintegrin ADAM8 promotes neurite outgrowth and suppresses neuronal cell death. *J Biol Chem* 2004; **279**(16)**:** 16083-16090.

169. Schlomann U, Rathke-Hartlieb S, Yamamoto S, Jockusch H, Bartsch JW. Tumor necrosis factor alpha induces a metalloprotease-disintegrin, ADAM8 (CD 156): implications for neuron-glia interactions during neurodegeneration. *J Neurosci* 2000; **20**(21)**:** 7964-7971.

170. Hwang Y, Kim J, Shin JY, Kim JI, Seo JS, Webster MJ*, et al*. Gene expression profiling by mRNA sequencing reveals increased expression of immune/inflammation-related genes in the hippocampus of individuals with schizophrenia. *Transl Psychiatry* 2013; **3:** e321.

171. Diamond MS, Farzan M. The broad-spectrum antiviral functions of IFIT and IFITM proteins. *Nat Rev Immunol* 2013; **13**(1)**:** 46-57.

172. Arion D, Unger T, Lewis DA, Levitt P, Mirnics K. Molecular evidence for increased expression of genes related to immune and chaperone function in the prefrontal cortex in schizophrenia. *Biol Psychiatry* 2007; **62**(7)**:** 711-721.

173. Huang CH, Chen ML, Tsai YL, Tsai MT, Chen CH. Elevated adrenomedullin mRNA in lymphoblastoid cells from schizophrenic patients. *Neuroreport* 2004; **15**(9)**:** 1443-1446.

174. Yilmaz N, Herken H, Cicek HK, Celik A, Yurekli M, Akyol O. Increased levels of nitric oxide, cortisol and adrenomedullin in patients with chronic schizophrenia. *Med Princ Pract* 2007; **16**(2)**:** 137-141.

175. Fernandez AP, Serrano J, Tessarollo L, Cuttitta F, Martinez A. Lack of adrenomedullin in the mouse brain results in behavioral changes, anxiety, and lower survival under stress conditions. *Proc Natl Acad Sci U S A* 2008; **105**(34)**:** 12581-12586.

176. Savas HA, Herken H, Yurekli M, Uz E, Tutkun H, Zoroglu SS*, et al*. Possible role of nitric oxide and adrenomedullin in bipolar affective disorder. *Neuropsychobiology* 2002; **45**(2)**:** 57-61.

177. Sheng M, Kim E. The Shank family of scaffold proteins. *J Cell Sci* 2000; **113 ( Pt 11):** 1851-1856.

178. Boeckers TM, Bockmann J, Kreutz MR, Gundelfinger ED. ProSAP/Shank proteins - a family of higher order organizing molecules of the postsynaptic density with an emerging role in human neurological disease. *J Neurochem* 2002; **81**(5)**:** 903-910.

179. Berkel S, Marshall CR, Weiss B, Howe J, Roeth R, Moog U*, et al*. Mutations in the SHANK2 synaptic scaffolding gene in autism spectrum disorder and mental retardation. *Nat Genet* 2010; **42**(6)**:** 489-491.

180. Peykov S, Berkel S, Schoen M, Weiss K, Degenhardt F, Strohmaier J*, et al*. Identification and functional characterization of rare SHANK2 variants in schizophrenia. *Mol Psychiatry* 2015; **20**(12)**:** 1489-1498.

181. Schmeisser MJ. Translational neurobiology in Shank mutant mice--model systems for neuropsychiatric disorders. *Ann Anat* 2015; **200:** 115-117.

182. Guilmatre A, Huguet G, Delorme R, Bourgeron T. The emerging role of SHANK genes in neuropsychiatric disorders. *Dev Neurobiol* 2014; **74**(2)**:** 113-122.

183. Gigante AD, Andreazza AC, Lafer B, Yatham LN, Beasley CL, Young LT. Decreased mRNA expression of uncoupling protein 2, a mitochondrial proton transporter, in post-mortem prefrontal cortex from patients with bipolar disorder and schizophrenia. *Neurosci Lett* 2011; **505**(1)**:** 47-51.

184. Yasuno K, Ando S, Misumi S, Makino S, Kulski JK, Muratake T*, et al*. Synergistic association of mitochondrial uncoupling protein (UCP) genes with schizophrenia. *Am J Med Genet B Neuropsychiatr Genet* 2007; **144B**(2)**:** 250-253.

185. Jajodia A, Kaur H, Kumari K, Gupta M, Baghel R, Srivastava A*, et al*. Evidence for schizophrenia susceptibility alleles in the Indian population: An association of neurodevelopmental genes in case-control and familial samples. *Schizophr Res* 2015; **162**(1-3)**:** 112-117.

186. Cui X, Pertile R, Liu P, Eyles DW. Vitamin D regulates tyrosine hydroxylase expression: N-cadherin a possible mediator. *Neuroscience* 2015; **304:** 90-100.

187. Sinclair D, Fillman SG, Webster MJ, Weickert CS. Dysregulation of glucocorticoid receptor co-factors FKBP5, BAG1 and PTGES3 in prefrontal cortex in psychotic illness. *Sci Rep* 2013; **3:** 3539.

188. Dittmar KD, Demady DR, Stancato LF, Krishna P, Pratt WB. Folding of the glucocorticoid receptor by the heat shock protein (hsp) 90-based chaperone machinery. The role of p23 is to stabilize receptor.hsp90 heterocomplexes formed by hsp90.p60.hsp70. *J Biol Chem* 1997; **272**(34)**:** 21213-21220.

189. Morishima Y, Kanelakis KC, Murphy PJ, Lowe ER, Jenkins GJ, Osawa Y*, et al*. The hsp90 cochaperone p23 is the limiting component of the multiprotein hsp90/hsp70-based chaperone system in vivo where it acts to stabilize the client protein: hsp90 complex. *J Biol Chem* 2003; **278**(49)**:** 48754-48763.

190. Wang Y, Shibasaki F, Mizuno K. Calcium signal-induced cofilin dephosphorylation is mediated by Slingshot via calcineurin. *J Biol Chem* 2005; **280**(13)**:** 12683-12689.

191. Yuen EY, Yan Z. Dopamine D4 receptors regulate AMPA receptor trafficking and glutamatergic transmission in GABAergic interneurons of prefrontal cortex. *J Neurosci* 2009; **29**(2)**:** 550-562.

192. Cao L, Jiao X, Zuzga DS, Liu Y, Fong DM, Young D*, et al*. VEGF links hippocampal activity with neurogenesis, learning and memory. *Nat Genet* 2004; **36**(8)**:** 827-835.

193. El Kissi Y, Samoud S, Mtiraoui A, Letaief L, Hannachi N, Ayachi M*, et al*. Increased Interleukin-17 and decreased BAFF serum levels in drug-free acute schizophrenia. *Psychiatry Res* 2015; **225**(1-2)**:** 58-63.

194. Chetcuti A, Adams LJ, Mitchell PB, Schofield PR. Microarray gene expression profiling of mouse brain mRNA in a model of lithium treatment. *Psychiatr Genet* 2008; **18**(2)**:** 64-72.

195. de Graaf N, van Helden MJ, Textoris-Taube K, Chiba T, Topham DJ, Kloetzel PM*, et al*. PA28 and the proteasome immunosubunits play a central and independent role in the production of MHC class I-binding peptides in vivo. *Eur J Immunol* 2011; **41**(4)**:** 926-935.

196. Li J, Powell SR, Wang X. Enhancement of proteasome function by PA28&alpha; overexpression protects against oxidative stress. *FASEB J* 2011; **25**(3)**:** 883-893.

197. Hinkle CL, Diestel S, Lieberman J, Maness PF. Metalloprotease-induced ectodomain shedding of neural cell adhesion molecule (NCAM). *J Neurobiol* 2006; **66**(12)**:** 1378-1395.

198. Brennaman LH, Moss ML, Maness PF. EphrinA/EphA-induced ectodomain shedding of neural cell adhesion molecule regulates growth cone repulsion through ADAM10 metalloprotease. *J Neurochem* 2014; **128**(2)**:** 267-279.

199. Brennaman LH, Maness PF. NCAM in neuropsychiatric and neurodegenerative disorders. *Adv Exp Med Biol* 2010; **663:** 299-317.

200. Bukalo O, Dityatev A. Synaptic cell adhesion molecules. *Adv Exp Med Biol* 2012; **970:** 97-128.

201. Han MH, Hu Z, Chen CY, Chen Y, Gucek M, Li Z*, et al*. Dysbindin-associated proteome in the p2 synaptosome fraction of mouse brain. *J Proteome Res* 2014; **13**(11)**:** 4567-4580.

202. Ishigaki S, Niwa J, Yoshihara T, Mitsuma N, Doyu M, Sobue G. Two novel genes, human neugrin and mouse m-neugrin, are upregulated with neuronal differentiation in neuroblastoma cells. *Biochem Biophys Res Commun* 2000; **279**(2)**:** 526-533.

203. Alkuraya FS, Cai X, Emery C, Mochida GH, Al-Dosari MS, Felie JM*, et al*. Human mutations in NDE1 cause extreme microcephaly with lissencephaly [corrected]. *Am J Hum Genet* 2011; **88**(5)**:** 536-547.

204. Feng Y, Walsh CA. Mitotic spindle regulation by Nde1 controls cerebral cortical size. *Neuron* 2004; **44**(2)**:** 279-293.

205. Hennah W, Tomppo L, Hiekkalinna T, Palo OM, Kilpinen H, Ekelund J*, et al*. Families with the risk allele of DISC1 reveal a link between schizophrenia and another component of the same molecular pathway, NDE1. *Hum Mol Genet* 2007; **16**(5)**:** 453-462.

206. Bradshaw NJ, Christie S, Soares DC, Carlyle BC, Porteous DJ, Millar JK. NDE1 and NDEL1: multimerisation, alternate splicing and DISC1 interaction. *Neurosci Lett* 2009; **449**(3)**:** 228-233.

207. Ingason A, Rujescu D, Cichon S, Sigurdsson E, Sigmundsson T, Pietilainen OP*, et al*. Copy number variations of chromosome 16p13.1 region associated with schizophrenia. *Mol Psychiatry* 2011; **16**(1)**:** 17-25.

208. Liu JY, Kasperaviciute D, Martinian L, Thom M, Sisodiya SM. Neuropathology of 16p13.11 deletion in epilepsy. *PLoS One* 2012; **7**(4)**:** e34813.

209. Tropeano M, Ahn JW, Dobson RJ, Breen G, Rucker J, Dixit A*, et al*. Male-biased autosomal effect of 16p13.11 copy number variation in neurodevelopmental disorders. *PLoS One* 2013; **8**(4)**:** e61365.

210. Kimura H, Tsuboi D, Wang C, Kushima I, Koide T, Ikeda M*, et al*. Identification of Rare, Single-Nucleotide Mutations in NDE1 and Their Contributions to Schizophrenia Susceptibility. *Schizophr Bull* 2015; **41**(3)**:** 744-753.

211. Wegelius A, Pankakoski M, Tomppo L, Lehto U, Lonnqvist J, Suvisaari J*, et al*. An interaction between NDE1 and high birth weight increases schizophrenia susceptibility. *Psychiatry Res* 2015; **230**(2)**:** 194-199.

212. Lashley T, Rohrer JD, Mead S, Revesz T. Review: An update on clinical, genetic and pathological aspects of frontotemporal lobar degenerations. *Neuropathol Appl Neurobiol* 2015; **41**(7)**:** 858-881.

213. Shinagawa S, Nakajima S, Plitman E, Graff-Guerrero A, Mimura M, Nakayama K*, et al*. Psychosis in frontotemporal dementia. *J Alzheimers Dis* 2014; **42**(2)**:** 485-499.

214. Choi KH, Elashoff M, Higgs BW, Song J, Kim S, Sabunciyan S*, et al*. Putative psychosis genes in the prefrontal cortex: combined analysis of gene expression microarrays. *BMC Psychiatry* 2008; **8:** 87.

215. West AK, Hidalgo J, Eddins D, Levin ED, Aschner M. Metallothionein in the central nervous system: Roles in protection, regeneration and cognition. *Neurotoxicology* 2008; **29**(3)**:** 489-503.

216. Aston C, Jiang L, Sokolov BP. Microarray analysis of postmortem temporal cortex from patients with schizophrenia. *J Neurosci Res* 2004; **77**(6)**:** 858-866.

217. Rousseau A, McEwen AG, Poussin-Courmontagne P, Rognan D, Nomine Y, Rio MC*, et al*. TRAF4 is a novel phosphoinositide-binding protein modulating tight junctions and favoring cell migration. *PLoS Biol* 2013; **11**(12)**:** e1001726.

218. Mathew SJ, Rembold M, Leptin M. Role for Traf4 in polarizing adherens junctions as a prerequisite for efficient cell shape changes. *Mol Cell Biol* 2011; **31**(24)**:** 4978-4993.

219. Eom DS, Amarnath S, Agarwala S. Apicobasal polarity and neural tube closure. *Dev Growth Differ* 2013; **55**(1)**:** 164-172.

220. Kalkan T, Iwasaki Y, Park CY, Thomsen GH. Tumor necrosis factor-receptor-associated factor-4 is a positive regulator of transforming growth factor-beta signaling that affects neural crest formation. *Mol Biol Cell* 2009; **20**(14)**:** 3436-3450.

221. Valerio A, Ferrario M, Martinez FO, Locati M, Ghisi V, Bresciani LG*, et al*. Gene expression profile activated by the chemokine CCL5/RANTES in human neuronal cells. *J Neurosci Res* 2004; **78**(3)**:** 371-382.

222. Park MH, Lee YK, Lee YH, Kim YB, Yun YW, Nam SY*, et al*. Chemokines released from astrocytes promote chemokine receptor 5-mediated neuronal cell differentiation. *Exp Cell Res* 2009; **315**(16)**:** 2715-2726.

223. Schwarz E, Guest PC, Rahmoune H, Harris LW, Wang L, Leweke FM*, et al*. Identification of a biological signature for schizophrenia in serum. *Mol Psychiatry* 2012; **17**(5)**:** 494-502.

224. Reale M, Patruno A, De Lutiis MA, Pesce M, Felaco M, Di Giannantonio M*, et al*. Dysregulation of chemo-cytokine production in schizophrenic patients versus healthy controls. *BMC Neurosci* 2011; **12:** 13.

225. Stuart MJ, Baune BT. Chemokines and chemokine receptors in mood disorders, schizophrenia, and cognitive impairment: a systematic review of biomarker studies. *Neurosci Biobehav Rev* 2014; **42:** 93-115.

226. Seeman P, Ko F, Jack E, Greenstein R, Dean B. Consistent with dopamine supersensitivity, RGS9 expression is diminished in the amphetamine-treated animal model of schizophrenia and in postmortem schizophrenia brain. *Synapse* 2007; **61**(5)**:** 303-309.

227. Rahman Z, Schwarz J, Gold SJ, Zachariou V, Wein MN, Choi KH*, et al*. RGS9 modulates dopamine signaling in the basal ganglia. *Neuron* 2003; **38**(6)**:** 941-952.

228. Matsuoka T, Tsunoda M, Sumiyoshi T, Takasaki I, Tabuchi Y, Seo T*, et al*. Effect of MK-801 on gene expressions in the amygdala of rats. *Synapse* 2008; **62**(1)**:** 1-7.

229. Beneyto M, Morris HM, Rovensky KC, Lewis DA. Lamina- and cell-specific alterations in cortical somatostatin receptor 2 mRNA expression in schizophrenia. *Neuropharmacology* 2012; **62**(3)**:** 1598-1605.

230. Merenlender-Wagner A, Malishkevich A, Shemer Z, Udawela M, Gibbons A, Scarr E*, et al*. Autophagy has a key role in the pathophysiology of schizophrenia. *Mol Psychiatry* 2015; **20**(1)**:** 126-132.

231. Alkelai A, Lupoli S, Greenbaum L, Kohn Y, Kanyas-Sarner K, Ben-Asher E*, et al*. DOCK4 and CEACAM21 as novel schizophrenia candidate genes in the Jewish population. *Int J Neuropsychopharmacol* 2012; **15**(4)**:** 459-469.

232. Stohr H, Berger C, Frohlich S, Weber BH. A novel gene encoding a putative transmembrane protein with two extracellular CUB domains and a low-density lipoprotein class A module: isolation of alternatively spliced isoforms in retina and brain. *Gene* 2002; **286**(2)**:** 223-231.

233. Li F, Tsien JZ. Memory and the NMDA receptors. *N Engl J Med* 2009; **361**(3)**:** 302-303.

234. O'Donnell L, Soileau B, Heard P, Carter E, Sebold C, Gelfond J*, et al*. Genetic determinants of autism in individuals with deletions of 18q. *Hum Genet* 2010; **128**(2)**:** 155-164.

235. Mayilyan KR, Weinberger DR, Sim RB. The complement system in schizophrenia. *Drug News Perspect* 2008; **21**(4)**:** 200-210.

236. Fourgeaud L, Boulanger LM. Synapse remodeling, compliments of the complement system. *Cell* 2007; **131**(6)**:** 1034-1036.

237. Michailidou I, Willems JG, Kooi EJ, van Eden C, Gold SM, Geurts JJ*, et al*. Complement C1q-C3-associated synaptic changes in multiple sclerosis hippocampus. *Ann Neurol* 2015; **77**(6)**:** 1007-1026.

238. Hakobyan S, Boyajyan A, Sim RB. Classical pathway complement activity in schizophrenia. *Neurosci Lett* 2005; **374**(1)**:** 35-37.

239. Ni J, Hu S, Zhang J, Tang W, Lu W, Zhang C. A Preliminary Genetic Analysis of Complement 3 Gene and Schizophrenia. *PLoS One* 2015; **10**(8)**:** e0136372.

240. Kim YK, Myint AM, Lee BH, Han CS, Lee HJ, Kim DJ*, et al*. Th1, Th2 and Th3 cytokine alteration in schizophrenia. *Prog Neuropsychopharmacol Biol Psychiatry* 2004; **28**(7)**:** 1129-1134.

241. Zhang L, Yi H, Xia XP, Zhao Y. Transforming growth factor-beta: an important role in CD4+CD25+ regulatory T cells and immune tolerance. *Autoimmunity* 2006; **39**(4)**:** 269-276.

242. Krieglstein K, Suter-Crazzolara C, Fischer WH, Unsicker K. TGF-beta superfamily members promote survival of midbrain dopaminergic neurons and protect them against MPP+ toxicity. *EMBO J* 1995; **14**(4)**:** 736-742.

243. Krieglstein K, Unsicker K. Transforming growth factor-beta promotes survival of midbrain dopaminergic neurons and protects them against N-methyl-4-phenylpyridinium ion toxicity. *Neuroscience* 1994; **63**(4)**:** 1189-1196.

244. Awad MR, El-Gamel A, Hasleton P, Turner DM, Sinnott PJ, Hutchinson IV. Genotypic variation in the transforming growth factor-beta1 gene: association with transforming growth factor-beta1 production, fibrotic lung disease, and graft fibrosis after lung transplantation. *Transplantation* 1998; **66**(8)**:** 1014-1020.

245. Frydecka D, Misiak B, Pawlak-Adamska E, Karabon L, Tomkiewicz A, Sedlaczek P*, et al*. Sex differences in TGFB-beta signaling with respect to age of onset and cognitive functioning in schizophrenia. *Neuropsychiatr Dis Treat* 2015; **11:** 575-584.

246. Boise LH, Gonzalez-Garcia M, Postema CE, Ding L, Lindsten T, Turka LA*, et al*. bcl-x, a bcl-2-related gene that functions as a dominant regulator of apoptotic cell death. *Cell* 1993; **74**(4)**:** 597-608.

247. Fatemi SH, Folsom TD, Reutiman TJ, Novak J, Engel RH. Comparative gene expression study of the chronic exposure to clozapine and haloperidol in rat frontal cortex. *Schizophr Res* 2012; **134**(2-3)**:** 211-218.

248. He J, Xu H, Yang Y, Rajakumar D, Li X, Li XM. The effects of chronic administration of quetiapine on the phencyclidine-induced reference memory impairment and decrease of Bcl-XL/Bax ratio in the posterior cingulate cortex in rats. *Behav Brain Res* 2006; **168**(2)**:** 236-242.

249. Wang C, McInnis J, Ross-Sanchez M, Shinnick-Gallagher P, Wiley JL, Johnson KM. Long-term behavioral and neurodegenerative effects of perinatal phencyclidine administration: implications for schizophrenia. *Neuroscience* 2001; **107**(4)**:** 535-550.

250. Ghahramani Seno MM, Gwadry FG, Hu P, Scherer SW. Neuregulin 1-alpha regulates phosphorylation, acetylation, and alternative splicing in lymphoblastoid cells. *Genome* 2013; **56**(10)**:** 619-625.

251. Sei Y, Ren-Patterson R, Li Z, Tunbridge EM, Egan MF, Kolachana BS*, et al*. Neuregulin1-induced cell migration is impaired in schizophrenia: association with neuregulin1 and catechol-o-methyltransferase gene polymorphisms. *Mol Psychiatry* 2007; **12**(10)**:** 946-957.

252. Bradford M, Law MH, Stewart AD, Shaw DJ, Megson IL, Wei J. The TGM2 gene is associated with schizophrenia in a British population. *Am J Med Genet B Neuropsychiatr Genet* 2009; **150B**(3)**:** 335-340.

253. Sollid LM. Coeliac disease: dissecting a complex inflammatory disorder. *Nat Rev Immunol* 2002; **2**(9)**:** 647-655.

254. Wang J, Liu Y, Wang Z, Du W, Hui L, Zhao X*, et al*. Lack of genetic association of the TGM2 gene with schizophrenia in a Chinese population. *Psychiatr Genet* 2015; **25**(6)**:** 259-262.

255. Hope S, Hoseth E, Dieset I, Morch RH, Aas M, Aukrust P*, et al*. Inflammatory markers are associated with general cognitive abilities in schizophrenia and bipolar disorder patients and healthy controls. *Schizophr Res* 2015; **165**(2-3)**:** 188-194.

256. Doecke JD, Laws SM, Faux NG, Wilson W, Burnham SC, Lam CP*, et al*. Blood-based protein biomarkers for diagnosis of Alzheimer disease. *Arch Neurol* 2012; **69**(10)**:** 1318-1325.

257. Giunta B, Rezai-Zadeh K, Tan J. Impact of the CD40-CD40L dyad in Alzheimer's disease. *CNS Neurol Disord Drug Targets* 2010; **9**(2)**:** 149-155.

258. Verhelst J, Hulpiau P, Saelens X. Mx proteins: antiviral gatekeepers that restrain the uninvited. *Microbiol Mol Biol Rev* 2013; **77**(4)**:** 551-566.

259. Haller O, Staeheli P, Schwemmle M, Kochs G. Mx GTPases: dynamin-like antiviral machines of innate immunity. *Trends Microbiol* 2015; **23**(3)**:** 154-163.

260. Dick A, Graf L, Olal D, von der Malsburg A, Gao S, Kochs G*, et al*. Role of nucleotide binding and GTPase domain dimerization in dynamin-like myxovirus resistance protein A for GTPase activation and antiviral activity. *J Biol Chem* 2015; **290**(20)**:** 12779-12792.

261. He Y, Sun S, Sha H, Liu Z, Yang L, Xue Z*, et al*. Emerging roles for XBP1, a sUPeR transcription factor. *Gene Expr* 2010; **15**(1)**:** 13-25.

262. Chen W, Duan S, Zhou J, Sun Y, Zheng Y, Gu N*, et al*. A case-control study provides evidence of association for a functional polymorphism -197C/G in XBP1 to schizophrenia and suggests a sex-dependent effect. *Biochem Biophys Res Commun* 2004; **319**(3)**:** 866-870.

263. Kakiuchi C, Ishiwata M, Umekage T, Tochigi M, Kohda K, Sasaki T*, et al*. Association of the XBP1-116C/G polymorphism with schizophrenia in the Japanese population. *Psychiatry Clin Neurosci* 2004; **58**(4)**:** 438-440.

264. Jonsson EG, Cichon S, Schumacher J, Abou Jamra R, Schulze TG, Deschner M*, et al*. Association study of a functional promoter polymorphism in the XBP1 gene and schizophrenia. *Am J Med Genet B Neuropsychiatr Genet* 2006; **141B**(1)**:** 71-75.

265. Watanabe Y, Fukui N, Muratake T, Amagane H, Kaneko N, Nunokawa A*, et al*. Association study of a functional promoter polymorphism of the X-box binding protein 1 gene in Japanese patients with schizophrenia. *Psychiatry Clin Neurosci* 2006; **60**(5)**:** 633-635.

266. Carter CJ. eIF2B and oligodendrocyte survival: where nature and nurture meet in bipolar disorder and schizophrenia? *Schizophr Bull* 2007; **33**(6)**:** 1343-1353.

267. Mitchell ES, Conus N, Kaput J. B vitamin polymorphisms and behavior: evidence of associations with neurodevelopment, depression, schizophrenia, bipolar disorder and cognitive decline. *Neurosci Biobehav Rev* 2014; **47:** 307-320.

268. Brown HE, Roffman JL. Vitamin supplementation in the treatment of schizophrenia. *CNS Drugs* 2014; **28**(7)**:** 611-622.

269. Cesca F, Baldelli P, Valtorta F, Benfenati F. The synapsins: key actors of synapse function and plasticity. *Prog Neurobiol* 2010; **91**(4)**:** 313-348.

270. Fassio A, Raimondi A, Lignani G, Benfenati F, Baldelli P. Synapsins: from synapse to network hyperexcitability and epilepsy. *Semin Cell Dev Biol* 2011; **22**(4)**:** 408-415.

271. Yu GI, Kim SK, Park HJ, Kim JW, Chung JH, Shin DH. The C allele of synonymous SNP (rs1142636, Asn170Asn) in SYN1 is a risk factor for the susceptibility of Korean female schizophrenia. *Synapse* 2012; **66**(11)**:** 979-983.

272. Greco B, Manago F, Tucci V, Kao HT, Valtorta F, Benfenati F. Autism-related behavioral abnormalities in synapsin knockout mice. *Behav Brain Res* 2013; **251:** 65-74.

273. Garcia CC, Blair HJ, Seager M, Coulthard A, Tennant S, Buddles M*, et al*. Identification of a mutation in synapsin I, a synaptic vesicle protein, in a family with epilepsy. *J Med Genet* 2004; **41**(3)**:** 183-186.

274. Fassio A, Patry L, Congia S, Onofri F, Piton A, Gauthier J*, et al*. SYN1 loss-of-function mutations in autism and partial epilepsy cause impaired synaptic function. *Hum Mol Genet* 2011; **20**(12)**:** 2297-2307.

275. Wesseling H, Rahmoune H, Tricklebank M, Guest PC, Bahn S. A targeted multiplexed proteomic investigation identifies ketamine-induced changes in immune markers in rat serum and expression changes in protein kinases/phosphatases in rat brain. *J Proteome Res* 2015; **14**(1)**:** 411-421.

276. Martin MV, Mirnics K, Nisenbaum LK, Vawter MP. Olanzapine Reversed Brain Gene Expression Changes Induced by Phencyclidine Treatment in Non-Human Primates. *Mol Neuropsychiatry* 2015; **1**(2)**:** 82-93.
