# Supplementary material for: Transcriptome sequencing study implicates immune-related genes differentially expressed in schizophrenia: new data and a meta-analysis
Source: Transl Psychiatry. 2017 Apr 18;7(4):e1093–. doi: 10.1038/tp.2017.47 (PMC5416689; doi:10.1038/tp.2017.47)
Supplement: Supplementary Table 3 [file tp201747x4.docx]

| **Table S3. Pathway analysis findings for 361 downregulated genes differentially expressed by affection status** | | | | |
| --- | --- | --- | --- | --- |
| **Category** | **GO ID** | **GO Term** | **Fold Enrichment** | **FDR** |
| Biological Process | GO:0048193 | Golgi vesicle transport | 7.18 | 9.42E-06 |
| Biological Process | GO:0015031 | protein transport | 2.78 | 1.04E-04 |
| Biological Process | GO:0046907 | intracellular transport | 2.95 | 1.08E-04 |
| Biological Process | GO:0045184 | establishment of protein localization | 2.75 | 1.30E-04 |
| Biological Process | GO:0006486 | protein amino acid glycosylation | 6.43 | 4.18E-04 |
| Biological Process | GO:0070085 | glycosylation | 6.43 | 4.18E-04 |
| Biological Process | GO:0043413 | biopolymer glycosylation | 6.43 | 4.18E-04 |
| Biological Process | GO:0018279 | protein amino acid N-linked glycosylation via asparagine | 35.29 | 5.17E-04 |
| Biological Process | GO:0018196 | peptidyl-asparagine modification | 35.29 | 5.17E-04 |
| Biological Process | GO:0009100 | glycoprotein metabolic process | 4.95 | 5.47E-04 |
| Biological Process | GO:0008104 | protein localization | 2.47 | 1.19E-03 |
| Biological Process | GO:0048199 | vesicle targeting, to, from or within Golgi | 29.41 | 1.58E-03 |
| Biological Process | GO:0006613 | cotranslational protein targeting to membrane | 29.41 | 1.58E-03 |
| Biological Process | GO:0006886 | intracellular protein transport | 3.46 | 2.54E-03 |
| Biological Process | GO:0009101 | glycoprotein biosynthetic process | 5.21 | 4.67E-03 |
| Biological Process | GO:0006986 | response to unfolded protein | 8.28 | 4.82E-03 |
| Biological Process | GO:0034613 | cellular protein localization | 3.15 | 1.10E-02 |
| Biological Process | GO:0070727 | cellular macromolecule localization | 3.13 | 1.24E-02 |
| Biological Process | GO:0006487 | protein amino acid N-linked glycosylation | 10.69 | 1.35E-02 |
| Biological Process | GO:0051789 | response to protein stimulus | 6.05 | 2.20E-02 |
| Biological Process | GO:0016192 | vesicle-mediated transport | 2.65 | 2.51E-02 |
| Biological Process | GO:0006612 | protein targeting to membrane | 12.48 | 2.75E-02 |
| Biological Process | GO:0006903 | vesicle targeting | 16.04 | 4.57E-02 |
| Cellular Component | GO:0005783 | endoplasmic reticulum | 4.42 | 4.89E-28 |
| Cellular Component | GO:0044432 | endoplasmic reticulum part | 7.03 | 2.80E-22 |
| Cellular Component | GO:0012505 | endomembrane system | 4.14 | 1.43E-18 |
| Cellular Component | GO:0005789 | endoplasmic reticulum membrane | 6.90 | 7.84E-16 |
| Cellular Component | GO:0042175 | nuclear envelope-endoplasmic reticulum network | 6.54 | 4.36E-15 |
| Cellular Component | GO:0031090 | organelle membrane | 3.15 | 6.23E-14 |
| Cellular Component | GO:0044431 | Golgi apparatus part | 4.87 | 6.37E-08 |
| Cellular Component | GO:0000139 | Golgi membrane | 5.99 | 3.92E-07 |
| Cellular Component | GO:0005794 | Golgi apparatus | 2.68 | 5.50E-06 |
| Cellular Component | GO:0005793 | ER-Golgi intermediate compartment | 12.94 | 6.85E-05 |
| Cellular Component | GO:0005788 | endoplasmic reticulum lumen | 7.96 | 3.60E-04 |
| Cellular Component | GO:0016021 | integral to membrane | 1.39 | 4.08E-04 |
| Cellular Component | GO:0030120 | vesicle coat | 12.56 | 5.46E-04 |
| Cellular Component | GO:0008250 | oligosaccharyltransferase complex | 31.82 | 6.90E-04 |
| Cellular Component | GO:0031224 | intrinsic to membrane | 1.35 | 2.24E-03 |
| Cellular Component | GO:0030135 | coated vesicle | 5.00 | 2.30E-03 |
| Cellular Component | GO:0031301 | integral to organelle membrane | 5.61 | 4.40E-03 |
| Cellular Component | GO:0031300 | intrinsic to organelle membrane | 5.09 | 4.79E-03 |
| Cellular Component | GO:0031410 | cytoplasmic vesicle | 2.56 | 4.83E-03 |
| Cellular Component | GO:0016023 | cytoplasmic membrane-bounded vesicle | 2.70 | 6.29E-03 |
| Cellular Component | GO:0031982 | vesicle | 2.45 | 1.12E-02 |
| Cellular Component | GO:0030662 | coated vesicle membrane | 7.27 | 1.13E-02 |
| Cellular Component | GO:0031988 | membrane-bounded vesicle | 2.61 | 1.13E-02 |
| Cellular Component | GO:0030117 | membrane coat | 7.58 | 2.89E-02 |
| Cellular Component | GO:0048475 | coated membrane | 7.58 | 2.89E-02 |
| Cellular Component | GO:0030176 | integral to endoplasmic reticulum membrane | 9.03 | 3.30E-02 |
| Cellular Component | GO:0030134 | ER to Golgi transport vesicle | 24.11 | 4.76E-02 |
| Molecular Function | GO:0004579 | dolichyl-diphosphooligosaccharide-protein glycotransferase activity | 41.41 | 1.69E-04 |
| Molecular Function | GO:0004576 | oligosaccharyl transferase activity | 37.27 | 3.34E-04 |
| Molecular Function | GO:0003756 | protein disulfide isomerase activity | 34.51 | 1.07E-02 |
| Molecular Function | GO:0016864 | intramolecular oxidoreductase activity, transposing S-S bonds | 34.51 | 1.07E-02 |
| Molecular Function | GO:0016862 | intramolecular oxidoreductase activity, interconverting keto- and enol-groups | 31.06 | 1.75E-02 |
| KEGG Pathway | hsa00510 | N-Glycan biosynthesis | 13.98 | 3.00E-06 |
| Note: GO Terms are tabulated only for those showing FDR<0.05 for fold enrichment. The input into the DAVID tools analysis was the list of 361 genes differentially expressed by affection status (Bonferroni *P*<0.05) that were expressed at lower levels in the schizophrenia cases. | | | | |
