# Supplementary material for: Transcriptome sequencing study implicates immune-related genes differentially expressed in schizophrenia: new data and a meta-analysis
Source: Transl Psychiatry. 2017 Apr 18;7(4):e1093–. doi: 10.1038/tp.2017.47 (PMC5416689; doi:10.1038/tp.2017.47)
Supplement: Supplementary Table 4 [file tp201747x5.docx]

| **Table S4. Genes differentially expressed by affection status across two technologies and two datasets** | | | | | |
| --- | --- | --- | --- | --- | --- |
| **Differential expression** | | **Array results** | | **RNAseq results** | |
| **Gene** | **Chromosome** | **Beta** | **FDR** | **Beta** | **Bonferroni** |
| *GBP4* | 1p22.2 | 0.160 | 1.71E-02 | 0.654 | 4.13E-14 |
| *GBP2* | 1p22.2 | 0.197 | 3.39E-03 | 0.362 | 1.31E-08 |
| *FAM69A* | 1p22.1 | -0.180 | 3.48E-03 | -0.170 | 1.78E-07 |
| *SYT11* | 1q22 | 0.155 | 2.79E-02 | 0.157 | 1.64E-08 |
| *STX6* | 1q25.3 | 0.175 | 1.23E-02 | 0.050 | 3.06E-06 |
| *ST6GAL1* | 3q27.3 | -0.143 | 1.73E-02 | -0.528 | 1.59E-16 |
| *LNPEP* | 5q15 | 0.154 | 2.07E-02 | 0.140 | 4.70E-08 |
| *GLO1* | 6p21.2 | 0.149 | 2.26E-02 | 0.154 | 2.21E-06 |
| *SGK1* | 6q23.2 | -0.138 | 4.97E-02 | -0.213 | 7.35E-07 |
| *FBXO32* | 8q24.13 | -0.133 | 3.87E-02 | -0.074 | 5.13E-06 |
| *TJP2* | 9q21.11 | 0.149 | 2.46E-02 | 0.226 | 4.38E-09 |
| *CASP1* | 11q22.3 | 0.143 | 4.91E-02 | 0.371 | 2.96E-07 |
| *ZNF821* | 16q22.3 | -0.152 | 2.31E-02 | -0.084 | 4.88E-06 |
| *GNG7* | 19p13.3 | -0.135 | 4.89E-02 | -0.213 | 9.41E-08 |
| *XBP1* | 22q12.1 | -0.146 | 2.39E-02 | -0.870 | 3.21E-07 |
| Note: The previously reported array results are on a sample of 268 schizophrenia cases and 446 controls, ^1^ and the current RNAseq results are on a fully independent (no shared subjects) sample of 529 schizophrenia cases and 660 controls. | | | | | |

**References.**

1. Sanders AR, Goring HH, Duan J, Drigalenko EI, Moy W, Freda J*, et al*. Transcriptome study of differential expression in schizophrenia. *Hum Mol Genet* 2013; **22**(24)**:** 5001-5014.
