# Supplementary material for: Transcriptome sequencing study implicates immune-related genes differentially expressed in schizophrenia: new data and a meta-analysis
Source: Transl Psychiatry. 2017 Apr 18;7(4):e1093–. doi: 10.1038/tp.2017.47 (PMC5416689; doi:10.1038/tp.2017.47)
Supplement: Supplementary Table 5 [file tp201747x6.docx]

| **Table S5. 647 Transcripts Differentially Expressed (Bonferroni *P*≤0.05) by Affection Status in Meta-analysis of array and RNAseq dataset** | | | | | | |
| --- | --- | --- | --- | --- | --- | --- |
| **ensGene** | **Gene Abbreviation** | **Location (hg38)** | **Brain Expression** | **Immune Function** | ***P*-value** | **Bonferroni** |
| ENSG00000188290.7 | *HES4* | chr1:998961-1000172 | yes | no | 3.59E-07 | 2.92E-03 |
| ENSG00000188157.10 | *AGRN* | chr1:1020122-1056118 | NA | no | 9.04E-08 | 7.36E-04 |
| ENSG00000171608.12 | *PIK3CD* | chr1:9651731-9729114 | yes | yes | 1.04E-09 | 8.47E-06 |
| ENSG00000116663.7 | *FBXO6* | chr1:11664123-11674354 | yes | yes | 9.53E-08 | 7.76E-04 |
| ENSG00000116685.12 | *KIAA2013* | chr1:11919590-11926428 | NA | no | 1.52E-06 | 1.24E-02 |
| ENSG00000142634.9 | *EFHD2* | chr1:15409894-15430343 | yes | yes | 6.68E-07 | 5.44E-03 |
| ENSG00000077549.14 | *CAPZB* | chr1:19338775-19485539 | yes | no | 2.61E-06 | 2.13E-02 |
| ENSG00000244038.6 | *DDOST* | chr1:20651766-20661544 | yes | yes | 1.05E-08 | 8.57E-05 |
| ENSG00000007968.6 | *E2F2* | chr1:23506429-23531220 | yes | yes | 3.01E-07 | 2.45E-03 |
| ENSG00000169504.11 | *CLIC4* | chr1:24745356-24844324 | yes | no | 1.41E-06 | 1.14E-02 |
| ENSG00000020633.15 | *RUNX3* | chr1:24899510-24965121 | yes | yes | 1.73E-11 | 1.41E-07 |
| ENSG00000157978.8 | *LDLRAP1* | chr1:25543579-25568886 | yes | no | 1.20E-09 | 9.73E-06 |
| ENSG00000117676.10 | *RPS6KA1* | chr1:26529760-26575030 | yes | yes | 1.81E-06 | 1.47E-02 |
| ENSG00000120656.8 | *TAF12* | chr1:28589322-28643085 | yes | no | 3.14E-07 | 2.56E-03 |
| ENSG00000159023.15 | *EPB41* | chr1:28887090-29120046 | yes | yes | 6.88E-09 | 5.60E-05 |
| ENSG00000160050.11 | *CCDC28B* | chr1:32200385-32205387 | yes | no | 1.11E-06 | 9.00E-03 |
| ENSG00000175130.6 | *MARCKSL1* | chr1:32333831-32336379 | yes | no | 2.93E-09 | 2.39E-05 |
| ENSG00000116514.13 | *RNF19B* | chr1:32936444-32964685 | yes | no | 5.27E-06 | 4.29E-02 |
| ENSG00000004455.13 | *AK2* | chr1:33007983-33080996 | yes | no | 1.50E-06 | 1.22E-02 |
| ENSG00000126067.8 | *PSMB2* | chr1:35599543-35641844 | yes | yes | 6.43E-11 | 5.23E-07 |
| ENSG00000163874.8 | *ZC3H12A* | chr1:37474551-37484379 | no | yes | 2.36E-09 | 1.92E-05 |
| ENSG00000196449.3 | *YRDC* | chr1:37802943-37808185 | NA | no | 8.50E-09 | 6.92E-05 |
| ENSG00000090621.10 | *PABPC4* | chr1:39560815-39576790 | yes | yes | 1.14E-09 | 9.29E-06 |
| ENSG00000159596.6 | *TMEM69* | chr1:45687213-45694443 | yes | no | 1.08E-07 | 8.77E-04 |
| ENSG00000117862.8 | *TXNDC12* | chr1:52020130-52056171 | yes | no | 5.68E-07 | 4.63E-03 |
| ENSG00000134717.14 | *BTF3L4* | chr1:52056124-52090716 | yes | no | 9.17E-09 | 7.46E-05 |
| ENSG00000162385.7 | *MAGOH* | chr1:53226891-53238610 | yes | no | 7.11E-08 | 5.79E-04 |
| ENSG00000058799.10 | *YIPF1* | chr1:53851718-53889834 | yes | no | 1.95E-06 | 1.59E-02 |
| ENSG00000162402.9 | *USP24* | chr1:55066358-55215113 | yes | no | 1.04E-07 | 8.49E-04 |
| ENSG00000184588.14 | *PDE4B* | chr1:65792513-66374579 | yes | yes | 2.48E-09 | 2.02E-05 |
| ENSG00000116791.10 | *CRYZ* | chr1:74705481-74733408 | yes | no | 9.04E-11 | 7.36E-07 |
| ENSG00000137959.12 | *IFI44L* | chr1:78619921-78646145 | yes | yes | 2.88E-08 | 2.35E-04 |
| ENSG00000137965.7 | *IFI44* | chr1:78649795-78664078 | yes | no | 2.89E-08 | 2.35E-04 |
| ENSG00000117151.9 | *CTBS* | chr1:84549605-84574480 | yes | no | 9.50E-07 | 7.73E-03 |
| ENSG00000153898.9 | *MCOLN2* | chr1:84925582-84997113 | no | no | 1.72E-10 | 1.40E-06 |
| ENSG00000055732.9 | *MCOLN3* | chr1:85018081-85048499 | no | no | 7.26E-08 | 5.91E-04 |
| ENSG00000117174.7 | *ZNHIT6* | chr1:85649422-85708433 | yes | yes | 3.88E-06 | 3.16E-02 |
| ENSG00000171502.11 | *COL24A1* | chr1:85729232-86156943 | yes | no | 6.88E-08 | 5.60E-04 |
| ENSG00000153936.13 | *HS2ST1* | chr1:86914647-87109998 | yes | yes | 2.24E-06 | 1.83E-02 |
| ENSG00000117228.9 | *GBP1* | chr1:89052318-89065360 | yes | yes | 2.10E-14 | 1.71E-10 |
| ENSG00000162645.9 | *GBP2* | chr1:89106131-89150456 | NA | yes | 6.45E-14 | 5.25E-10 |
| ENSG00000162654.8 | *GBP4* | chr1:89181147-89198932 | yes | yes | 1.85E-17 | 1.50E-13 |
| ENSG00000154511.8 | *FAM69A* | chr1:92832736-92961522 | yes | no | 1.87E-12 | 1.52E-08 |
| ENSG00000079335.14 | *CDC14A* | chr1:100351733-100520277 | yes | no | 1.03E-09 | 8.40E-06 |
| ENSG00000198890.7 | *PRMT6* | chr1:107056678-107059294 | yes | yes | 1.26E-09 | 1.02E-05 |
| ENSG00000065135.8 | *GNAI3* | chr1:109548610-109618321 | yes | yes | 2.98E-06 | 2.43E-02 |
| ENSG00000155363.15 | *MOV10* | chr1:112673140-112700746 | yes | yes | 1.64E-09 | 1.33E-05 |
| ENSG00000163399.12 | *ATP1A1* | chr1:116372667-116410261 | yes | no | 1.67E-06 | 1.36E-02 |
| ENSG00000183508.4 | *FAM46C* | chr1:117605933-117628372 | yes | no | 1.92E-09 | 1.56E-05 |
| ENSG00000213190.3 | *MLLT11* | chr1:151057757-151068497 | yes | no | 2.73E-06 | 2.22E-02 |
| ENSG00000197747.5 | *S100A10* | chr1:151982914-151994390 | yes | no | 4.61E-06 | 3.75E-02 |
| ENSG00000143570.14 | *SLC39A1* | chr1:153959098-153968184 | yes | no | 1.75E-08 | 1.42E-04 |
| ENSG00000143515.13 | *ATP8B2* | chr1:154325552-154351307 | yes | no | 5.11E-12 | 4.16E-08 |
| ENSG00000179085.7 | *DPM3* | chr1:155139890-155140595 | yes | no | 3.07E-07 | 2.50E-03 |
| ENSG00000177628.12 | *GBA* | chr1:155234451-155244699 | yes | yes | 2.66E-08 | 2.16E-04 |
| ENSG00000132718.8 | *SYT11* | chr1:155859508-155885199 | yes | no | 8.46E-12 | 6.88E-08 |
| ENSG00000163479.10 | *SSR2* | chr1:156009047-156020959 | yes | yes | 6.81E-07 | 5.54E-03 |
| ENSG00000160803.7 | *UBQLN4* | chr1:156035300-156053794 | NA | no | 3.46E-10 | 2.82E-06 |
| ENSG00000160789.16 | *LMNA* | chr1:156082572-156140089 | yes | yes | 1.19E-08 | 9.70E-05 |
| ENSG00000163565.15 | *IFI16* | chr1:158999967-159055155 | yes | yes | 6.96E-07 | 5.66E-03 |
| ENSG00000158710.11 | *TAGLN2* | chr1:159918106-159925732 | yes | no | 5.77E-07 | 4.70E-03 |
| ENSG00000162734.9 | *PEA15* | chr1:160205336-160215376 | yes | no | 2.25E-08 | 1.83E-04 |
| ENSG00000117090.11 | *SLAMF1* | chr1:160608099-160647295 | no | yes | 1.33E-06 | 1.09E-02 |
| ENSG00000158850.11 | *B4GALT3* | chr1:161171309-161177968 | yes | no | 3.42E-10 | 2.78E-06 |
| ENSG00000143153.9 | *ATP1B1* | chr1:169105696-169132722 | yes | no | 3.53E-07 | 2.88E-03 |
| ENSG00000143156.10 | *NME7* | chr1:169132530-169367967 | yes | no | 4.04E-07 | 3.29E-03 |
| ENSG00000188404.5 | *SELL* | chr1:169690666-169711698 | yes | yes | 1.92E-06 | 1.56E-02 |
| ENSG00000075945.9 | *KIFAP3* | chr1:169921325-170085208 | yes | yes | 7.46E-08 | 6.07E-04 |
| ENSG00000117533.11 | *VAMP4* | chr1:171700159-171742247 | yes | no | 4.03E-08 | 3.28E-04 |
| ENSG00000135823.10 | *STX6* | chr1:180972711-181023121 | yes | no | 1.86E-10 | 1.51E-06 |
| ENSG00000143333.6 | *RGS16* | chr1:182598622-182604408 | yes | no | 7.04E-10 | 5.73E-06 |
| ENSG00000135838.10 | *NPL* | chr1:182789292-182830384 | yes | no | 3.62E-06 | 2.95E-02 |
| ENSG00000135829.13 | *DHX9* | chr1:182839368-182887751 | yes | yes | 5.64E-08 | 4.59E-04 |
| ENSG00000116406.15 | *EDEM3* | chr1:184690230-184754913 | yes | no | 3.44E-06 | 2.80E-02 |
| ENSG00000116679.12 | *IVNS1ABP* | chr1:185296387-185317329 | yes | yes | 4.65E-06 | 3.78E-02 |
| ENSG00000090104.8 | *RGS1* | chr1:192575726-192580031 | yes | yes | 8.47E-12 | 6.89E-08 |
| ENSG00000122188.9 | *LAX1* | chr1:203765175-203776233 | no | yes | 1.46E-07 | 1.19E-03 |
| ENSG00000158715.5 | *SLC45A3* | chr1:205657850-205680459 | yes | no | 7.76E-13 | 6.32E-09 |
| ENSG00000266028.4 | *SRGAP2* | chr1:206203344-206464443 | NA | no | 9.46E-07 | 7.70E-03 |
| ENSG00000117322.13 | *CR2* | chr1:207454229-207489895 | no | yes | 6.82E-11 | 5.55E-07 |
| ENSG00000009790.11 | *TRAF3IP3* | chr1:209756031-209782320 | no | no | 3.97E-07 | 3.23E-03 |
| ENSG00000162772.13 | *ATF3* | chr1:212565333-212620777 | yes | yes | 1.59E-15 | 1.29E-11 |
| ENSG00000123685.5 | *BATF3* | chr1:212686417-212699985 | yes | yes | 9.47E-10 | 7.71E-06 |
| ENSG00000143494.12 | *VASH2* | chr1:212950519-212992037 | yes | no | 8.93E-07 | 7.27E-03 |
| ENSG00000136643.8 | *RPS6KC1* | chr1:213051232-213274773 | yes | no | 7.11E-08 | 5.79E-04 |
| ENSG00000182827.8 | *ACBD3* | chr1:226144678-226186730 | yes | no | 2.20E-06 | 1.79E-02 |
| ENSG00000116962.11 | *NID1* | chr1:235975829-236065162 | yes | no | 7.68E-10 | 6.25E-06 |
| ENSG00000134321.8 | *RSAD2* | chr2:6865805-6898239 | yes | yes | 5.32E-06 | 4.33E-02 |
| ENSG00000115884.7 | *SDC1* | chr2:20200796-20225433 | yes | no | 1.98E-07 | 1.62E-03 |
| ENSG00000084733.7 | *RAB10* | chr2:26034106-26137454 | yes | no | 5.34E-06 | 4.34E-02 |
| ENSG00000119777.15 | *TMEM214* | chr2:27032909-27041695 | yes | no | 4.72E-07 | 3.85E-03 |
| ENSG00000138074.11 | *SLC5A6* | chr2:27199586-27212958 | yes | no | 1.09E-07 | 8.85E-04 |
| ENSG00000162959.10 | *MEMO1* | chr2:31865059-32011230 | yes | no | 7.23E-07 | 5.89E-03 |
| ENSG00000008869.8 | *HEATR5B* | chr2:36968382-37084342 | NA | no | 2.61E-08 | 2.13E-04 |
| ENSG00000055332.13 | *EIF2AK2* | chr2:37099209-37157065 | yes | yes | 4.13E-07 | 3.37E-03 |
| ENSG00000119729.7 | *RHOQ* | chr2:46541805-46583121 | NA | no | 2.11E-11 | 1.72E-07 |
| ENSG00000151665.9 | *PIGF* | chr2:46580936-46617119 | no | no | 3.64E-09 | 2.97E-05 |
| ENSG00000184261.4 | *KCNK12* | chr2:47516580-47570939 | yes | no | 2.55E-06 | 2.08E-02 |
| ENSG00000028116.13 | *VRK2* | chr2:57907650-58159920 | yes | no | 4.52E-10 | 3.68E-06 |
| ENSG00000115392.8 | *FANCL* | chr2:58159242-58241372 | yes | no | 7.89E-12 | 6.42E-08 |
| ENSG00000170340.10 | *B3GNT2* | chr2:62196112-62224731 | yes | no | 1.68E-08 | 1.37E-04 |
| ENSG00000115504.11 | *EHBP1* | chr2:62673850-63046487 | yes | no | 8.27E-10 | 6.73E-06 |
| ENSG00000115956.9 | *PLEK* | chr2:68365172-68397453 | yes | yes | 1.39E-07 | 1.13E-03 |
| ENSG00000163219.8 | *ARHGAP25* | chr2:68679600-68826833 | no | no | 8.44E-07 | 6.87E-03 |
| ENSG00000169564.6 | *PCBP1* | chr2:70087453-70089203 | yes | no | 4.54E-07 | 3.70E-03 |
| ENSG00000135638.10 | *EMX1* | chr2:72916259-72936071 | yes | yes | 5.90E-11 | 4.81E-07 |
| ENSG00000239779.3 | *WBP1* | chr2:74458328-74460891 | NA | no | 8.18E-10 | 6.66E-06 |
| ENSG00000239305.3 | *RNF103* | chr2:86603392-86623866 | yes | no | 7.33E-07 | 5.97E-03 |
| ENSG00000172071.8 | *EIF2AK3* | chr2:88556740-88627576 | yes | yes | 7.15E-11 | 5.82E-07 |
| ENSG00000196843.12 | *ARID5A* | chr2:96536742-96552638 | yes | no | 7.55E-07 | 6.14E-03 |
| ENSG00000115604.7 | *IL18R1* | chr2:102311528-102398775 | no | yes | 2.38E-08 | 1.94E-04 |
| ENSG00000071051.10 | *NCK2* | chr2:105744896-105894274 | yes | no | 3.87E-08 | 3.15E-04 |
| ENSG00000115109.10 | *EPB41L5* | chr2:120013004-120179119 | yes | no | 6.45E-07 | 5.25E-03 |
| ENSG00000150540.10 | *HNMT* | chr2:137964019-138016364 | yes | yes | 6.26E-07 | 5.10E-03 |
| ENSG00000150556.13 | *LYPD6B* | chr2:149038106-149215262 | yes | no | 5.29E-07 | 4.31E-03 |
| ENSG00000123609.7 | *NMI* | chr2:151270464-151290057 | no | no | 1.57E-09 | 1.28E-05 |
| ENSG00000123610.4 | *TNFAIP6* | chr2:151357591-151380048 | yes | yes | 4.46E-07 | 3.63E-03 |
| ENSG00000115267.5 | *IFIH1* | chr2:162267078-162318703 | yes | yes | 3.38E-13 | 2.75E-09 |
| ENSG00000073737.13 | *DHRS9* | chr2:169064788-169096167 | no | no | 3.30E-08 | 2.69E-04 |
| ENSG00000077380.12 | *DYNC1I2* | chr2:171687408-171748420 | yes | no | 3.14E-07 | 2.56E-03 |
| ENSG00000115368.6 | *WDR75* | chr2:189441432-189475565 | yes | no | 1.45E-10 | 1.18E-06 |
| ENSG00000115415.15 | *STAT1* | chr2:190964357-191020960 | yes | yes | 3.28E-11 | 2.67E-07 |
| ENSG00000138442.6 | *WDR12* | chr2:202874781-203014798 | yes | no | 1.91E-07 | 1.55E-03 |
| ENSG00000119004.11 | *CYP20A1* | chr2:203238448-203305840 | yes | no | 4.61E-06 | 3.76E-02 |
| ENSG00000118246.10 | *FASTKD2* | chr2:206765356-206792509 | yes | no | 4.74E-07 | 3.86E-03 |
| ENSG00000118242.12 | *MREG* | chr2:215942804-216034096 | yes | no | 2.76E-08 | 2.25E-04 |
| ENSG00000135929.5 | *CYP27A1* | chr2:218781748-218815293 | yes | no | 4.99E-08 | 4.06E-04 |
| ENSG00000123992.15 | *DNPEP* | chr2:219373545-219400022 | yes | no | 1.63E-07 | 1.33E-03 |
| ENSG00000144591.14 | *GMPPA* | chr2:219498866-219506988 | yes | no | 1.79E-07 | 1.45E-03 |
| ENSG00000163082.9 | *SGPP2* | chr2:222424516-222560948 | yes | no | 1.31E-06 | 1.06E-02 |
| ENSG00000135932.7 | *CAB39* | chr2:230712844-230821075 | yes | no | 4.29E-06 | 3.49E-02 |
| ENSG00000173692.9 | *PSMD1* | chr2:231056863-231172827 | yes | yes | 1.96E-08 | 1.60E-04 |
| ENSG00000134109.7 | *EDEM1* | chr3:5187645-5219957 | yes | no | 1.28E-06 | 1.04E-02 |
| ENSG00000180914.7 | *OXTR* | chr3:8750407-8769628 | yes | no | 4.90E-11 | 3.99E-07 |
| ENSG00000214021.12 | *TTLL3* | chr3:9808085-9855138 | yes | no | 8.33E-07 | 6.78E-03 |
| ENSG00000163704.8 | *PRRT3* | chr3:9945541-9952394 | yes | no | 4.63E-11 | 3.77E-07 |
| ENSG00000206560.7 | *ANKRD28* | chr3:15667235-15859771 | yes | no | 8.83E-09 | 7.19E-05 |
| ENSG00000172936.9 | *MYD88* | chr3:38138477-38143022 | yes | yes | 2.32E-08 | 1.89E-04 |
| ENSG00000172939.5 | *OXSR1* | chr3:38165088-38255488 | yes | no | 1.96E-09 | 1.59E-05 |
| ENSG00000121807.5 | *CCR2* | chr3:46353733-46360928 | no | yes | 4.89E-06 | 3.98E-02 |
| ENSG00000173540.9 | *GMPPB* | chr3:49716843-49723951 | yes | no | 1.82E-08 | 1.48E-04 |
| ENSG00000016864.13 | *GLT8D1* | chr3:52694484-52706083 | yes | no | 7.43E-12 | 6.05E-08 |
| ENSG00000114902.10 | *SPCS1* | chr3:52704954-52711146 | yes | no | 7.07E-11 | 5.76E-07 |
| ENSG00000168297.12 | *PXK* | chr3:58332879-58426126 | yes | no | 1.99E-06 | 1.62E-02 |
| ENSG00000168301.9 | *KCTD6* | chr3:58492113-58502360 | yes | no | 2.69E-07 | 2.19E-03 |
| ENSG00000144744.13 | *UBA3* | chr3:69054729-69080408 | yes | yes | 5.96E-08 | 4.85E-04 |
| ENSG00000114480.9 | *GBE1* | chr3:81489698-81762161 | yes | yes | 5.64E-07 | 4.59E-03 |
| ENSG00000168386.15 | *FILIP1L* | chr3:99830140-100114513 | no | no | 5.03E-07 | 4.09E-03 |
| ENSG00000114021.8 | *NIT2* | chr3:100334700-100356866 | yes | no | 1.17E-09 | 9.55E-06 |
| ENSG00000114354.9 | *TFG* | chr3:100709330-100748966 | yes | no | 1.59E-06 | 1.30E-02 |
| ENSG00000186265.6 | *BTLA* | chr3:112463967-112499561 | no | yes | 1.42E-06 | 1.16E-02 |
| ENSG00000176142.9 | *TMEM39A* | chr3:119429499-119468830 | yes | no | 2.93E-07 | 2.38E-03 |
| ENSG00000121594.8 | *CD80* | chr3:119524292-119559602 | no | yes | 4.10E-11 | 3.33E-07 |
| ENSG00000144837.5 | *PLA1A* | chr3:119597841-119629811 | yes | no | 2.08E-10 | 1.69E-06 |
| ENSG00000145088.5 | *EAF2* | chr3:121835182-121886526 | yes | no | 1.78E-06 | 1.45E-02 |
| ENSG00000138496.13 | *PARP9* | chr3:122527923-122564577 | yes | no | 4.48E-11 | 3.65E-07 |
| ENSG00000163840.6 | *DTX3L* | chr3:122564237-122575203 | yes | yes | 1.56E-07 | 1.27E-03 |
| ENSG00000173193.10 | *PARP14* | chr3:122680617-122730840 | yes | no | 3.74E-08 | 3.04E-04 |
| ENSG00000058262.6 | *SEC61A1* | chr3:128051640-128071683 | yes | yes | 6.25E-08 | 5.09E-04 |
| ENSG00000184897.5 | *H1FX* | chr3:129314770-129316277 | yes | no | 1.01E-06 | 8.24E-03 |
| ENSG00000091527.12 | *CDV3* | chr3:133573729-133590261 | yes | no | 4.43E-06 | 3.61E-02 |
| ENSG00000177311.7 | *ZBTB38* | chr3:141324212-141449792 | NA | no | 1.11E-10 | 9.01E-07 |
| ENSG00000175040.4 | *CHST2* | chr3:143119330-143122958 | yes | yes | 1.78E-06 | 1.45E-02 |
| ENSG00000188313.9 | *PLSCR1* | chr3:146515179-146544864 | yes | yes | 2.65E-13 | 2.16E-09 |
| ENSG00000070087.10 | *PFN2* | chr3:149964903-150050788 | yes | yes | 2.29E-07 | 1.86E-03 |
| ENSG00000114850.3 | *SSR3* | chr3:156540139-156555184 | yes | no | 1.21E-07 | 9.87E-04 |
| ENSG00000008952.13 | *SEC62* | chr3:169966634-169998373 | yes | no | 1.50E-09 | 1.22E-05 |
| ENSG00000121858.7 | *TNFSF10* | chr3:172505507-172523507 | yes | yes | 7.81E-09 | 6.36E-05 |
| ENSG00000172578.8 | *KLHL6* | chr3:183487530-183555689 | no | yes | 7.96E-07 | 6.48E-03 |
| ENSG00000114796.12 | *KLHL24* | chr3:183635567-183684477 | yes | no | 6.94E-08 | 5.65E-04 |
| ENSG00000073849.11 | *ST6GAL1* | chr3:186930484-187078553 | yes | yes | 1.39E-19 | 1.13E-15 |
| ENSG00000136514.2 | *RTP4* | chr3:187368331-187372076 | yes | no | 3.43E-08 | 2.79E-04 |
| ENSG00000184203.4 | *PPP1R2* | chr3:195514424-195543386 | yes | no | 7.92E-07 | 6.45E-03 |
| ENSG00000061938.13 | *TNK2* | chr3:195863363-195911945 | yes | no | 3.31E-06 | 2.70E-02 |
| ENSG00000163132.6 | *MSX1* | chr4:4859665-4863936 | yes | no | 1.01E-06 | 8.19E-03 |
| ENSG00000109689.11 | *STIM2* | chr4:26857677-27025381 | yes | yes | 2.10E-06 | 1.71E-02 |
| ENSG00000163694.11 | *RBM47* | chr4:40423266-40630875 | yes | no | 8.29E-10 | 6.75E-06 |
| ENSG00000188848.12 | *BEND4* | chr4:42110937-42152878 | yes | no | 7.07E-09 | 5.75E-05 |
| ENSG00000169019.10 | *COMMD8* | chr4:47450795-47463719 | yes | no | 2.21E-07 | 1.80E-03 |
| ENSG00000035720.4 | *STAP1* | chr4:67558727-67607337 | yes | no | 4.18E-06 | 3.40E-02 |
| ENSG00000138764.10 | *CCNG2* | chr4:77157150-77433388 | yes | no | 6.37E-08 | 5.19E-04 |
| ENSG00000145293.11 | *ENOPH1* | chr4:82430561-82461091 | yes | no | 1.70E-07 | 1.38E-03 |
| ENSG00000145287.7 | *PLAC8* | chr4:83090047-83137075 | no | no | 4.88E-07 | 3.97E-03 |
| ENSG00000173085.10 | *COQ2* | chr4:83261535-83284914 | yes | no | 2.85E-06 | 2.32E-02 |
| ENSG00000173083.11 | *HPSE* | chr4:83292460-83335153 | no | yes | 2.50E-08 | 2.03E-04 |
| ENSG00000138642.11 | *HERC6* | chr4:88378738-88443111 | yes | no | 7.56E-08 | 6.16E-04 |
| ENSG00000138646.5 | *HERC5* | chr4:88457116-88506163 | yes | yes | 9.22E-11 | 7.51E-07 |
| ENSG00000109320.8 | *NFKB1* | chr4:102501328-102617302 | yes | yes | 4.00E-13 | 3.25E-09 |
| ENSG00000178403.3 | *NEUROG2* | chr4:112513515-112516172 | yes | no | 4.58E-06 | 3.73E-02 |
| ENSG00000150961.11 | *SEC24D* | chr4:118722822-118838683 | yes | yes | 6.77E-07 | 5.51E-03 |
| ENSG00000164070.8 | *HSPA4L* | chr4:127781820-127840733 | yes | no | 2.04E-06 | 1.66E-02 |
| ENSG00000179387.6 | *ELMOD2* | chr4:140524157-140553770 | yes | no | 4.62E-09 | 3.76E-05 |
| ENSG00000164136.13 | *IL15* | chr4:141636598-141733987 | no | yes | 1.30E-06 | 1.06E-02 |
| ENSG00000164164.12 | *OTUD4* | chr4:145110837-145180161 | yes | no | 3.78E-08 | 3.08E-04 |
| ENSG00000170006.8 | *TMEM154* | chr4:152618631-152680165 | yes | no | 2.82E-06 | 2.30E-02 |
| ENSG00000121210.12 | *KIAA0922* | chr4:153466345-153636711 | yes | no | 1.32E-07 | 1.08E-03 |
| ENSG00000137628.13 | *DDX60* | chr4:168216292-168318807 | yes | no | 1.05E-09 | 8.55E-06 |
| ENSG00000129128.9 | *SPCS3* | chr4:176319963-176332245 | yes | no | 1.67E-08 | 1.36E-04 |
| ENSG00000164305.14 | *CASP3* | chr4:184627695-184649509 | yes | yes | 2.83E-07 | 2.30E-03 |
| ENSG00000186352.5 | *ANKRD37* | chr4:185396020-185400628 | yes | no | 5.08E-06 | 4.13E-02 |
| ENSG00000049656.10 | *CLPTM1L* | chr5:1317743-1345099 | yes | yes | 2.21E-10 | 1.80E-06 |
| ENSG00000164237.5 | *CMBL* | chr5:10275874-10308026 | yes | no | 2.05E-07 | 1.67E-03 |
| ENSG00000113387.8 | *SUB1* | chr5:32531632-32604079 | yes | no | 3.17E-11 | 2.58E-07 |
| ENSG00000168724.11 | *DNAJC21* | chr5:34929592-34958964 | yes | no | 1.64E-07 | 1.34E-03 |
| ENSG00000123213.19 | *NLN* | chr5:65722195-65871725 | yes | no | 2.78E-06 | 2.26E-02 |
| ENSG00000152942.15 | *RAD17* | chr5:69369292-69414801 | yes | no | 7.03E-07 | 5.72E-03 |
| ENSG00000081189.10 | *MEF2C* | chr5:88718157-88904105 | yes | yes | 3.10E-06 | 2.52E-02 |
| ENSG00000175471.16 | *MCTP1* | chr5:94703740-95284575 | yes | no | 2.46E-06 | 2.01E-02 |
| ENSG00000118985.11 | *ELL2* | chr5:95885097-95962071 | yes | no | 1.64E-07 | 1.34E-03 |
| ENSG00000113441.12 | *LNPEP* | chr5:96935393-97037515 | yes | yes | 1.08E-11 | 8.81E-08 |
| ENSG00000064651.10 | *SLC12A2* | chr5:128083765-128189688 | yes | no | 1.93E-06 | 1.57E-02 |
| ENSG00000066583.8 | *ISOC1* | chr5:129094750-129114028 | yes | no | 1.11E-06 | 9.03E-03 |
| ENSG00000125347.10 | *IRF1* | chr5:132481608-132490798 | yes | yes | 9.70E-07 | 7.89E-03 |
| ENSG00000113558.15 | *SKP1* | chr5:134148934-134177038 | yes | yes | 2.97E-07 | 2.42E-03 |
| ENSG00000113615.9 | *SEC24A* | chr5:134648788-134727823 | yes | no | 3.36E-07 | 2.73E-03 |
| ENSG00000031003.7 | *FAM13B* | chr5:137937959-138051961 | yes | no | 4.65E-06 | 3.79E-02 |
| ENSG00000145901.11 | *TNIP1* | chr5:151029944-151093577 | yes | yes | 6.04E-11 | 4.92E-07 |
| ENSG00000113282.10 | *CLINT1* | chr5:157785742-157859175 | yes | no | 1.24E-11 | 1.01E-07 |
| ENSG00000169220.14 | *RGS14* | chr5:177357836-177372601 | yes | yes | 5.18E-07 | 4.22E-03 |
| ENSG00000146094.10 | *DOK3* | chr5:177501906-177511274 | yes | yes | 3.31E-07 | 2.69E-03 |
| ENSG00000131446.12 | *MGAT1* | chr5:180790540-180815652 | yes | no | 1.90E-06 | 1.54E-02 |
| ENSG00000124535.12 | *WRNIP1* | chr6:2765413-2786952 | yes | yes | 1.48E-07 | 1.20E-03 |
| ENSG00000124783.9 | *SSR1* | chr6:7268305-7347446 | yes | no | 2.65E-10 | 2.16E-06 |
| ENSG00000239264.5 | *TXNDC5* | chr6:7881516-7910814 | yes | no | 6.47E-12 | 5.27E-08 |
| ENSG00000205269.5 | *TMEM170B* | chr6:11538277-11583524 | NA | no | 2.16E-11 | 1.76E-07 |
| ENSG00000137414.5 | *FAM8A1* | chr6:17600354-17611719 | yes | no | 1.08E-06 | 8.77E-03 |
| ENSG00000137364.4 | *TPMT* | chr6:18128310-18155074 | yes | no | 1.17E-07 | 9.56E-04 |
| ENSG00000111913.12 | *FAM65B* | chr6:24797372-25042168 | yes | no | 1.01E-06 | 8.21E-03 |
| ENSG00000213886.3 | *UBD* | chr6:29555514-29559925 | no | no | 6.29E-15 | 5.12E-11 |
| ENSG00000204642.10 | *HLA-F* | chr6:29722774-29738528 | yes | yes | 1.00E-10 | 8.18E-07 |
| ENSG00000206503.8 | *HLA-A* | chr6:29941259-29945884 | NA | yes | 5.50E-08 | 4.48E-04 |
| ENSG00000204619.4 | *PPP1R11* | chr6:30066708-30070333 | yes | no | 3.43E-06 | 2.79E-02 |
| ENSG00000137331.11 | *IER3* | chr6:30743198-30744554 | yes | yes | 8.71E-09 | 7.09E-05 |
| ENSG00000204386.7 | *NEU1* | chr6:31857658-31862906 | yes | yes | 5.34E-10 | 4.35E-06 |
| ENSG00000213676.7 | *ATF6B* | chr6:32098175-32128253 | NA | no | 2.21E-07 | 1.80E-03 |
| ENSG00000204310.7 | *AGPAT1* | chr6:32168211-32178096 | yes | no | 1.79E-06 | 1.45E-02 |
| ENSG00000204264.5 | *PSMB8* | chr6:32840716-32844703 | no | yes | 1.24E-06 | 1.01E-02 |
| ENSG00000240065.4 | *PSMB9* | chr6:32844135-32859585 | yes | yes | 4.75E-07 | 3.87E-03 |
| ENSG00000168394.10 | *TAP1* | chr6:32845208-32853978 | yes | yes | 1.40E-08 | 1.14E-04 |
| ENSG00000112473.13 | *SLC39A7* | chr6:33200444-33204439 | yes | no | 1.90E-07 | 1.55E-03 |
| ENSG00000096060.11 | *FKBP5* | chr6:35573584-35728583 | yes | yes | 4.86E-08 | 3.95E-04 |
| ENSG00000112079.8 | *STK38* | chr6:36493891-36547470 | yes | no | 2.12E-08 | 1.72E-04 |
| ENSG00000124767.6 | *GLO1* | chr6:38675924-38703141 | yes | no | 6.64E-10 | 5.40E-06 |
| ENSG00000112561.14 | *TFEB* | chr6:41683977-41736259 | yes | yes | 1.84E-09 | 1.50E-05 |
| ENSG00000171467.12 | *ZNF318* | chr6:43307133-43369478 | yes | no | 4.34E-11 | 3.54E-07 |
| ENSG00000096384.16 | *HSP90AB1* | chr6:44246165-44253888 | yes | yes | 3.28E-09 | 2.67E-05 |
| ENSG00000146232.11 | *NFKBIE* | chr6:44258165-44265788 | yes | yes | 4.42E-09 | 3.60E-05 |
| ENSG00000065308.4 | *TRAM2* | chr6:52497401-52576915 | yes | no | 1.77E-07 | 1.44E-03 |
| ENSG00000082269.13 | *FAM135A* | chr6:70412940-70561174 | yes | no | 9.40E-11 | 7.65E-07 |
| ENSG00000083097.11 | *DOPEY1* | chr6:83067665-83171350 | yes | no | 3.76E-06 | 3.06E-02 |
| ENSG00000013392.7 | *RWDD2A* | chr6:83193378-83198932 | yes | no | 1.18E-09 | 9.58E-06 |
| ENSG00000198833.6 | *UBE2J1* | chr6:89326624-89352848 | yes | yes | 2.92E-12 | 2.37E-08 |
| ENSG00000112249.10 | *ASCC3* | chr6:100508193-100881372 | yes | yes | 3.81E-06 | 3.10E-02 |
| ENSG00000057657.11 | *PRDM1* | chr6:106086319-106109939 | yes | yes | 2.60E-06 | 2.11E-02 |
| ENSG00000025796.10 | *SEC63* | chr6:107867755-107958189 | yes | yes | 1.55E-06 | 1.26E-02 |
| ENSG00000056972.15 | *TRAF3IP2* | chr6:111556453-111606278 | yes | yes | 4.48E-06 | 3.65E-02 |
| ENSG00000010810.14 | *FYN* | chr6:111660331-111873452 | yes | yes | 5.28E-06 | 4.30E-02 |
| ENSG00000152894.11 | *PTPRK* | chr6:127968778-128520674 | yes | yes | 2.69E-08 | 2.19E-04 |
| ENSG00000079931.11 | *MOXD1* | chr6:132296054-132401545 | yes | no | 3.32E-07 | 2.70E-03 |
| ENSG00000118515.8 | *SGK1* | chr6:134169245-134318112 | yes | no | 9.07E-10 | 7.38E-06 |
| ENSG00000118503.11 | *TNFAIP3* | chr6:137867187-137883312 | no | yes | 1.53E-08 | 1.25E-04 |
| ENSG00000164442.9 | *CITED2* | chr6:139371806-139374620 | yes | yes | 4.10E-09 | 3.34E-05 |
| ENSG00000146425.7 | *DYNLT1* | chr6:158636473-158644739 | yes | no | 8.63E-12 | 7.02E-08 |
| ENSG00000106266.5 | *SNX8* | chr7:2251769-2354318 | yes | no | 2.23E-06 | 1.82E-02 |
| ENSG00000075624.10 | *ACTB* | chr7:5527150-5563784 | yes | yes | 2.41E-06 | 1.96E-02 |
| ENSG00000075618.14 | *FSCN1* | chr7:5592822-5606655 | yes | no | 1.84E-12 | 1.50E-08 |
| ENSG00000136240.6 | *KDELR2* | chr7:6445952-6484242 | yes | no | 4.55E-07 | 3.71E-03 |
| ENSG00000106415.9 | *GLCCI1* | chr7:7968793-8094272 | yes | no | 9.36E-08 | 7.62E-04 |
| ENSG00000106460.15 | *TMEM106B* | chr7:12211240-12243367 | yes | no | 6.07E-07 | 4.94E-03 |
| ENSG00000136261.11 | *BZW2* | chr7:16646130-16706523 | yes | no | 1.26E-08 | 1.03E-04 |
| ENSG00000050344.8 | *NFE2L3* | chr7:26152239-26187125 | yes | no | 5.21E-07 | 4.24E-03 |
| ENSG00000086300.12 | *SNX10* | chr7:26291894-26374329 | yes | no | 7.51E-07 | 6.12E-03 |
| ENSG00000010270.10 | *STARD3NL* | chr7:38178221-38230671 | yes | no | 6.11E-06 | 4.97E-02 |
| ENSG00000106605.7 | *BLVRA* | chr7:43758679-43807342 | yes | no | 1.35E-06 | 1.10E-02 |
| ENSG00000183696.10 | *UPP1* | chr7:48088627-48108733 | yes | yes | 6.35E-10 | 5.17E-06 |
| ENSG00000127951.5 | *FGL2* | chr7:77193370-77199826 | yes | yes | 8.13E-07 | 6.62E-03 |
| ENSG00000005469.8 | *CROT* | chr7:87345680-87399795 | yes | no | 2.31E-08 | 1.88E-04 |
| ENSG00000164715.5 | *LMTK2* | chr7:98106884-98209633 | yes | no | 9.24E-08 | 7.52E-04 |
| ENSG00000241685.5 | *ARPC1A* | chr7:99325897-99388164 | yes | yes | 6.99E-10 | 5.69E-06 |
| ENSG00000205277.6 | *MUC12* | chr7:100969622-101018949 | NA | no | 2.14E-06 | 1.75E-02 |
| ENSG00000106367.10 | *AP1S1* | chr7:101154396-101161596 | yes | no | 4.54E-08 | 3.69E-04 |
| ENSG00000160999.9 | *SH2B2* | chr7:102285090-102321711 | yes | yes | 1.52E-06 | 1.24E-02 |
| ENSG00000105835.8 | *NAMPT* | chr7:106248284-106286326 | yes | yes | 2.36E-10 | 1.92E-06 |
| ENSG00000105974.8 | *CAV1* | chr7:116524784-116561184 | yes | yes | 2.67E-06 | 2.18E-02 |
| ENSG00000128595.13 | *CALU* | chr7:128739291-128771807 | yes | no | 5.03E-07 | 4.09E-03 |
| ENSG00000186591.8 | *UBE2H* | chr7:129830731-129952949 | yes | yes | 4.97E-06 | 4.04E-02 |
| ENSG00000172331.8 | *BPGM* | chr7:134646807-134679813 | yes | no | 4.81E-07 | 3.92E-03 |
| ENSG00000182158.11 | *CREB3L2* | chr7:137874978-138002067 | yes | no | 1.91E-11 | 1.55E-07 |
| ENSG00000064393.12 | *HIPK2* | chr7:139561569-139777778 | yes | no | 7.77E-08 | 6.33E-04 |
| ENSG00000059378.9 | *PARP12* | chr7:140023743-140063721 | yes | no | 8.00E-07 | 6.51E-03 |
| ENSG00000257093.3 | *KIAA1147* | chr7:141656727-141702153 | yes | no | 4.56E-06 | 3.71E-02 |
| ENSG00000155660.7 | *PDIA4* | chr7:149003061-149028641 | yes | no | 9.23E-10 | 7.52E-06 |
| ENSG00000127399.11 | *LRRC61* | chr7:150322638-150338150 | yes | no | 8.68E-08 | 7.06E-04 |
| ENSG00000133627.14 | *ACTR3B* | chr7:152759748-152855378 | yes | yes | 5.72E-09 | 4.66E-05 |
| ENSG00000186480.9 | *INSIG1* | chr7:155297775-155310235 | yes | no | 8.00E-09 | 6.51E-05 |
| ENSG00000136573.9 | *BLK* | chr8:11494000-11564604 | no | yes | 2.80E-06 | 2.28E-02 |
| ENSG00000154328.12 | *NEIL2* | chr8:11769638-11787346 | yes | no | 1.08E-06 | 8.80E-03 |
| ENSG00000104763.14 | *ASAH1* | chr8:18056424-18084985 | yes | no | 2.48E-06 | 2.02E-02 |
| ENSG00000120910.11 | *PPP3CC* | chr8:22440818-22541142 | yes | yes | 8.44E-08 | 6.87E-04 |
| ENSG00000120889.9 | *TNFRSF10B* | chr8:23020132-23069179 | no | yes | 8.20E-09 | 6.68E-05 |
| ENSG00000197217.9 | *ENTPD4* | chr8:23385782-23457695 | yes | no | 7.96E-08 | 6.48E-04 |
| ENSG00000168081.5 | *PNOC* | chr8:28316985-28343355 | yes | yes | 2.34E-08 | 1.90E-04 |
| ENSG00000133874.1 | *RNF122* | chr8:33547754-33567125 | yes | no | 1.07E-09 | 8.69E-06 |
| ENSG00000147535.13 | *PPAPDC1B* | chr8:38263129-38269243 | yes | no | 7.45E-07 | 6.07E-03 |
| ENSG00000147526.16 | *TACC1* | chr8:38728185-38853028 | yes | no | 4.81E-09 | 3.91E-05 |
| ENSG00000168300.10 | *PCMTD1* | chr8:51817574-51899186 | yes | no | 8.38E-08 | 6.82E-04 |
| ENSG00000147509.10 | *RGS20* | chr8:53851807-53959303 | yes | no | 1.58E-06 | 1.29E-02 |
| ENSG00000180828.2 | *BHLHE22* | chr8:64580366-64583628 | yes | no | 6.24E-09 | 5.08E-05 |
| ENSG00000067167.4 | *TRAM1* | chr8:70573441-70608387 | yes | yes | 5.11E-06 | 4.16E-02 |
| ENSG00000178860.8 | *MSC* | chr8:71841548-71844468 | yes | no | 8.59E-11 | 6.99E-07 |
| ENSG00000104432.9 | *IL7* | chr8:78675742-78805523 | no | yes | 3.92E-10 | 3.19E-06 |
| ENSG00000164683.13 | *HEY1* | chr8:79764009-79767863 | yes | no | 2.06E-08 | 1.67E-04 |
| ENSG00000076554.12 | *TPD52* | chr8:80034867-80231232 | yes | yes | 1.73E-10 | 1.41E-06 |
| ENSG00000104312.7 | *RIPK2* | chr8:89757746-89791063 | yes | yes | 2.53E-10 | 2.06E-06 |
| ENSG00000164823.6 | *OSGIN2* | chr8:89901858-89927888 | yes | no | 2.04E-07 | 1.66E-03 |
| ENSG00000104320.10 | *NBN* | chr8:89933335-90003228 | yes | yes | 3.65E-09 | 2.97E-05 |
| ENSG00000164938.10 | *TP53INP1* | chr8:94925971-94949411 | yes | yes | 2.91E-09 | 2.37E-05 |
| ENSG00000147649.6 | *MTDH* | chr8:97644178-97728770 | yes | no | 1.79E-09 | 1.46E-05 |
| ENSG00000132541.7 | *HRSP12* | chr8:98102343-98117241 | yes | no | 2.85E-06 | 2.32E-02 |
| ENSG00000104356.7 | *POP1* | chr8:98117296-98159834 | yes | no | 2.14E-06 | 1.74E-02 |
| ENSG00000164920.6 | *OSR2* | chr8:98944402-98952104 | yes | no | 2.71E-06 | 2.21E-02 |
| ENSG00000104450.9 | *SPAG1* | chr8:100157905-100259278 | yes | no | 8.41E-07 | 6.85E-03 |
| ENSG00000156804.4 | *FBXO32* | chr8:123497888-123541206 | yes | no | 3.94E-09 | 3.21E-05 |
| ENSG00000173334.3 | *TRIB1* | chr8:125430320-125438405 | yes | yes | 8.21E-07 | 6.69E-03 |
| ENSG00000104419.11 | *NDRG1* | chr8:133237170-133302022 | yes | yes | 4.19E-08 | 3.41E-04 |
| ENSG00000160932.7 | *LY6E* | chr8:143017981-143023832 | yes | no | 9.09E-07 | 7.40E-03 |
| ENSG00000178685.10 | *PARP10* | chr8:143977152-144012772 | yes | no | 4.52E-10 | 3.68E-06 |
| ENSG00000178719.13 | *GRINA* | chr8:143990057-143993415 | yes | no | 3.01E-08 | 2.45E-04 |
| ENSG00000196922.7 | *ZNF252P* | chr8:144973588-145002895 | NA | no | 1.29E-07 | 1.05E-03 |
| ENSG00000198642.6 | *KLHL9* | chr9:21329670-21335380 | yes | yes | 8.72E-07 | 7.10E-03 |
| ENSG00000107201.6 | *DDX58* | chr9:32455704-32526324 | yes | yes | 1.43E-08 | 1.17E-04 |
| ENSG00000086061.12 | *DNAJA1* | chr9:33025210-33039907 | yes | no | 5.65E-15 | 4.60E-11 |
| ENSG00000086065.10 | *CHMP5* | chr9:33264878-33282069 | yes | no | 5.01E-09 | 4.08E-05 |
| ENSG00000137100.12 | *DCTN3* | chr9:34613544-34620523 | yes | no | 6.29E-07 | 5.12E-03 |
| ENSG00000159921.11 | *GNE* | chr9:36214440-36277056 | yes | yes | 2.32E-07 | 1.89E-03 |
| ENSG00000137106.14 | *GRHPR* | chr9:37422665-37436990 | yes | no | 2.84E-13 | 2.31E-09 |
| ENSG00000119139.13 | *TJP2* | chr9:69121263-69255208 | yes | no | 1.98E-12 | 1.61E-08 |
| ENSG00000131669.6 | *NINJ1* | chr9:93121488-93134288 | yes | yes | 1.14E-06 | 9.25E-03 |
| ENSG00000095380.10 | *NANS* | chr9:98056738-98083075 | yes | no | 3.48E-06 | 2.84E-02 |
| ENSG00000119523.9 | *ALG2* | chr9:99216425-99221956 | yes | no | 9.95E-07 | 8.10E-03 |
| ENSG00000023318.7 | *ERP44* | chr9:99979178-100099040 | yes | no | 1.13E-09 | 9.21E-06 |
| ENSG00000106701.8 | *FSD1L* | chr9:105447795-105552433 | yes | no | 1.23E-10 | 1.00E-06 |
| ENSG00000136810.9 | *TXN* | chr9:110243810-110256640 | NA | yes | 3.18E-07 | 2.59E-03 |
| ENSG00000136888.6 | *ATP6V1G1* | chr9:114587745-114598373 | yes | no | 1.31E-06 | 1.07E-02 |
| ENSG00000056558.7 | *TRAF1* | chr9:120902392-120929173 | no | yes | 1.28E-10 | 1.04E-06 |
| ENSG00000165209.15 | *STRBP* | chr9:123109499-123268576 | yes | no | 8.00E-09 | 6.51E-05 |
| ENSG00000119487.13 | *MAPKAP1* | chr9:125437392-125707234 | yes | no | 4.20E-09 | 3.42E-05 |
| ENSG00000136840.15 | *ST6GALNAC4* | chr9:127907885-127917038 | yes | no | 7.47E-07 | 6.08E-03 |
| ENSG00000167106.8 | *FAM102A* | chr9:127940578-127980513 | yes | no | 1.86E-06 | 1.52E-02 |
| ENSG00000125485.14 | *DDX31* | chr9:132592996-132670401 | yes | no | 7.26E-10 | 5.91E-06 |
| ENSG00000067082.11 | *KLF6* | chr10:3775995-3785281 | yes | no | 4.48E-07 | 3.65E-03 |
| ENSG00000134452.16 | *FBXO18* | chr10:5890202-5937594 | yes | no | 2.05E-07 | 1.67E-03 |
| ENSG00000134460.12 | *IL2RA* | chr10:6010688-6062325 | no | yes | 4.69E-06 | 3.82E-02 |
| ENSG00000134453.12 | *RBM17* | chr10:6088986-6117457 | yes | no | 7.85E-07 | 6.39E-03 |
| ENSG00000152465.14 | *NMT2* | chr10:15102583-15168693 | yes | no | 1.94E-06 | 1.58E-02 |
| ENSG00000148484.14 | *RSU1* | chr10:16590610-16817528 | yes | no | 1.56E-06 | 1.27E-02 |
| ENSG00000165996.10 | *PTPLA* | chr10:17589031-17617377 | yes | no | 1.68E-07 | 1.37E-03 |
| ENSG00000168283.10 | *BMI1* | chr10:22321210-22331484 | yes | yes | 5.36E-07 | 4.37E-03 |
| ENSG00000185875.9 | *THNSL1* | chr10:25016657-25026664 | yes | no | 1.85E-06 | 1.51E-02 |
| ENSG00000128815.14 | *WDFY4* | chr10:48684875-48982956 | no | no | 7.87E-17 | 6.41E-13 |
| ENSG00000122873.8 | *CISD1* | chr10:58269057-58289586 | yes | no | 9.80E-07 | 7.98E-03 |
| ENSG00000108091.10 | *CCDC6* | chr10:59788762-59906656 | yes | no | 1.78E-06 | 1.45E-02 |
| ENSG00000150347.11 | *ARID5B* | chr10:61901299-62096944 | NA | yes | 6.55E-09 | 5.33E-05 |
| ENSG00000122862.4 | *SRGN* | chr10:69088105-69104811 | yes | no | 7.23E-11 | 5.88E-07 |
| ENSG00000180817.8 | *PPA1* | chr10:70202829-70233911 | yes | no | 2.39E-07 | 1.94E-03 |
| ENSG00000138279.12 | *ANXA7* | chr10:73375100-73414076 | yes | yes | 8.19E-15 | 6.67E-11 |
| ENSG00000108219.11 | *TSPAN14* | chr10:80454165-80533123 | yes | no | 5.09E-06 | 4.15E-02 |
| ENSG00000198682.9 | *PAPSS2* | chr10:87659612-87747705 | yes | yes | 1.64E-09 | 1.34E-05 |
| ENSG00000026103.16 | *FAS* | chr10:88990530-89015785 | yes | yes | 1.24E-08 | 1.01E-04 |
| ENSG00000119922.8 | *IFIT2* | chr10:89301954-89309276 | yes | yes | 4.20E-13 | 3.42E-09 |
| ENSG00000119917.10 | *IFIT3* | chr10:89327893-89340971 | yes | yes | 6.10E-16 | 4.97E-12 |
| ENSG00000185745.9 | *IFIT1* | chr10:89392545-89406486 | yes | no | 3.36E-07 | 2.74E-03 |
| ENSG00000173145.8 | *NOC3L* | chr10:94333225-94362959 | yes | yes | 5.24E-07 | 4.26E-03 |
| ENSG00000077147.11 | *TM9SF3* | chr10:96518108-96587452 | yes | yes | 1.42E-06 | 1.16E-02 |
| ENSG00000077150.14 | *NFKB2* | chr10:102394109-102402529 | yes | yes | 2.07E-13 | 1.68E-09 |
| ENSG00000165806.16 | *CASP7* | chr10:113679161-113730907 | yes | yes | 1.19E-06 | 9.68E-03 |
| ENSG00000148908.11 | *RGS10* | chr10:119499827-119542708 | yes | no | 3.04E-06 | 2.47E-02 |
| ENSG00000068383.15 | *INPP5A* | chr10:132537819-132783480 | yes | no | 4.06E-07 | 3.30E-03 |
| ENSG00000151651.12 | *ADAM8* | chr10:133262402-133276868 | yes | yes | 1.85E-06 | 1.51E-02 |
| ENSG00000142089.12 | *IFITM3* | chr11:319668-327537 | no | yes | 1.01E-06 | 8.25E-03 |
| ENSG00000177105.9 | *RHOG* | chr11:3826977-3840983 | yes | yes | 2.57E-13 | 2.09E-09 |
| ENSG00000132109.9 | *TRIM21* | chr11:4384896-4393696 | no | yes | 2.66E-10 | 2.17E-06 |
| ENSG00000132256.15 | *TRIM5* | chr11:5663556-5938619 | no | yes | 3.24E-06 | 2.64E-02 |
| ENSG00000132274.12 | *TRIM22* | chr11:5689688-5737089 | yes | yes | 5.23E-14 | 4.26E-10 |
| ENSG00000166311.6 | *SMPD1* | chr11:6390430-6394998 | yes | yes | 1.65E-07 | 1.35E-03 |
| ENSG00000166333.10 | *ILK* | chr11:6603707-6610874 | yes | yes | 3.69E-06 | 3.00E-02 |
| ENSG00000148925.7 | *BTBD10* | chr11:13388000-13463297 | yes | no | 1.83E-07 | 1.49E-03 |
| ENSG00000129084.14 | *PSMA1* | chr11:14504873-14643635 | yes | yes | 3.44E-07 | 2.80E-03 |
| ENSG00000049449.5 | *RCN1* | chr11:31812390-32105755 | NA | no | 2.81E-06 | 2.28E-02 |
| ENSG00000135378.3 | *PRRG4* | chr11:32829942-32858123 | no | no | 4.41E-08 | 3.59E-04 |
| ENSG00000179431.6 | *FJX1* | chr11:35618418-35620868 | yes | no | 2.09E-08 | 1.70E-04 |
| ENSG00000085117.8 | *CD82* | chr11:44564426-44620363 | yes | no | 5.92E-18 | 4.82E-14 |
| ENSG00000156587.12 | *UBE2L6* | chr11:57551655-57568284 | yes | yes | 1.77E-14 | 1.44E-10 |
| ENSG00000166889.13 | *PATL1* | chr11:59636715-59668980 | yes | no | 8.46E-07 | 6.88E-03 |
| ENSG00000089597.13 | *GANAB* | chr11:62624825-62646726 | yes | no | 1.17E-06 | 9.53E-03 |
| ENSG00000162298.13 | *SYVN1* | chr11:65121779-65134533 | yes | yes | 2.42E-09 | 1.97E-05 |
| ENSG00000175550.4 | *DRAP1* | chr11:65919256-65921561 | yes | no | 2.63E-06 | 2.14E-02 |
| ENSG00000175463.8 | *TBC1D10C* | chr11:67403914-67410089 | NA | no | 8.61E-09 | 7.01E-05 |
| ENSG00000175634.11 | *RPS6KB2* | chr11:67428459-67435408 | yes | no | 9.02E-08 | 7.34E-04 |
| ENSG00000214530.4 | *STARD10* | chr11:72754728-72793681 | yes | no | 3.05E-06 | 2.48E-02 |
| ENSG00000214517.5 | *PPME1* | chr11:74171098-74254703 | yes | no | 3.73E-09 | 3.04E-05 |
| ENSG00000118363.8 | *SPCS2* | chr11:74949246-74979031 | NA | no | 5.88E-07 | 4.79E-03 |
| ENSG00000137491.11 | *SLCO2B1* | chr11:75100562-75206549 | yes | no | 7.57E-07 | 6.16E-03 |
| ENSG00000123892.8 | *RAB38* | chr11:88113241-88175467 | no | yes | 6.42E-11 | 5.23E-07 |
| ENSG00000109861.12 | *CTSC* | chr11:88293591-88337787 | yes | yes | 3.29E-07 | 2.68E-03 |
| ENSG00000196371.3 | *FUT4* | chr11:94543839-94549898 | yes | yes | 3.32E-06 | 2.70E-02 |
| ENSG00000149218.4 | *ENDOD1* | chr11:95089809-95132645 | yes | no | 3.35E-09 | 2.72E-05 |
| ENSG00000023445.10 | *BIRC3* | chr11:102317449-102339403 | no | yes | 4.87E-17 | 3.96E-13 |
| ENSG00000137673.5 | *MMP7* | chr11:102520507-102530753 | no | yes | 6.67E-12 | 5.43E-08 |
| ENSG00000137752.19 | *CASP1* | chr11:105025442-105035250 | no | yes | 3.91E-10 | 3.18E-06 |
| ENSG00000137710.11 | *RDX* | chr11:110174879-110296722 | yes | no | 1.16E-06 | 9.46E-03 |
| ENSG00000110777.8 | *POU2AF1* | chr11:111352251-111455630 | no | yes | 2.98E-06 | 2.42E-02 |
| ENSG00000172269.13 | *DPAGT1* | chr11:119096502-119108331 | yes | no | 1.10E-08 | 8.96E-05 |
| ENSG00000134910.9 | *STT3A* | chr11:125591711-125625215 | yes | yes | 1.12E-07 | 9.09E-04 |
| ENSG00000182934.8 | *SRPR* | chr11:126262918-126269144 | yes | no | 2.81E-09 | 2.29E-05 |
| ENSG00000151702.13 | *FLI1* | chr11:128686534-128813267 | yes | yes | 3.14E-06 | 2.56E-02 |
| ENSG00000151502.7 | *VPS26B* | chr11:134224644-134247792 | yes | no | 2.31E-10 | 1.88E-06 |
| ENSG00000110848.5 | *CD69* | chr12:9752485-9760901 | no | yes | 5.09E-07 | 4.15E-03 |
| ENSG00000111276.7 | *CDKN1B* | chr12:12715057-12722371 | yes | yes | 3.14E-10 | 2.56E-06 |
| ENSG00000213782.4 | *DDX47* | chr12:12813315-12829981 | yes | no | 4.83E-10 | 3.93E-06 |
| ENSG00000246705.4 | *H2AFJ* | chr12:14774382-14778002 | yes | no | 2.25E-06 | 1.83E-02 |
| ENSG00000111348.5 | *ARHGDIB* | chr12:14942016-14961728 | yes | yes | 3.01E-06 | 2.45E-02 |
| ENSG00000118308.11 | *LRMP* | chr12:25021001-25108334 | yes | no | 1.74E-06 | 1.42E-02 |
| ENSG00000170456.11 | *DENND5B* | chr12:31382222-31591097 | yes | no | 1.06E-07 | 8.63E-04 |
| ENSG00000177119.12 | *ANO6* | chr12:45215986-45440404 | NA | no | 1.25E-06 | 1.02E-02 |
| ENSG00000111424.7 | *VDR* | chr12:47841536-47943048 | no | yes | 6.91E-11 | 5.63E-07 |
| ENSG00000134285.7 | *FKBP11* | chr12:48921517-48926474 | yes | yes | 1.23E-06 | 1.00E-02 |
| ENSG00000161791.10 | *FMNL3* | chr12:49636498-49708165 | yes | no | 4.29E-07 | 3.49E-03 |
| ENSG00000135457.6 | *TFCP2* | chr12:51093662-51173134 | yes | no | 1.12E-06 | 9.15E-03 |
| ENSG00000135473.11 | *PAN2* | chr12:56316222-56334053 | yes | no | 7.36E-08 | 5.99E-04 |
| ENSG00000123329.14 | *ARHGAP9* | chr12:57472254-57488814 | yes | no | 5.38E-06 | 4.38E-02 |
| ENSG00000135446.13 | *CDK4* | chr12:57747726-57756013 | yes | yes | 1.23E-08 | 1.00E-04 |
| ENSG00000174106.2 | *LEMD3* | chr12:65169570-65248327 | yes | no | 2.06E-06 | 1.68E-02 |
| ENSG00000166226.9 | *CCT2* | chr12:69585333-69601570 | yes | no | 8.29E-08 | 6.75E-04 |
| ENSG00000127328.18 | *RAB3IP* | chr12:69738680-69823204 | yes | no | 6.88E-10 | 5.60E-06 |
| ENSG00000139291.10 | *TMEM19* | chr12:71686086-71705046 | yes | no | 3.90E-08 | 3.18E-04 |
| ENSG00000136026.10 | *CKAP4* | chr12:106237876-106304279 | yes | no | 1.87E-10 | 1.52E-06 |
| ENSG00000084112.11 | *SSH1* | chr12:108782693-108857590 | yes | no | 9.11E-07 | 7.41E-03 |
| ENSG00000135148.8 | *TRAFD1* | chr12:112125500-112153609 | yes | yes | 5.19E-10 | 4.22E-06 |
| ENSG00000111331.9 | *OAS3* | chr12:112938351-112973249 | yes | yes | 5.41E-08 | 4.40E-04 |
| ENSG00000170855.3 | *TRIAP1* | chr12:120443960-120446412 | no | no | 3.38E-06 | 2.75E-02 |
| ENSG00000139725.4 | *RHOF* | chr12:121777753-121803403 | NA | no | 1.77E-08 | 1.44E-04 |
| ENSG00000150977.10 | *RILPL2* | chr12:123410682-123436717 | NA | no | 5.32E-12 | 4.33E-08 |
| ENSG00000073060.12 | *SCARB1* | chr12:124776855-124882668 | yes | yes | 2.21E-06 | 1.80E-02 |
| ENSG00000184992.10 | *BRI3BP* | chr12:124993699-125031231 | yes | no | 8.69E-07 | 7.07E-03 |
| ENSG00000111450.10 | *STX2* | chr12:130789599-130839266 | yes | yes | 3.29E-08 | 2.68E-04 |
| ENSG00000176915.11 | *ANKLE2* | chr12:132725502-132761888 | NA | no | 1.14E-06 | 9.24E-03 |
| ENSG00000165474.5 | *GJB2* | chr13:20187469-20192898 | yes | no | 4.32E-07 | 3.52E-03 |
| ENSG00000152484.10 | *USP12* | chr13:27066141-27171896 | yes | no | 2.21E-06 | 1.80E-02 |
| ENSG00000132963.7 | *POMP* | chr13:28659103-28678925 | yes | yes | 9.18E-07 | 7.47E-03 |
| ENSG00000120697.5 | *ALG5* | chr13:36949774-37000261 | yes | no | 4.70E-06 | 3.83E-02 |
| ENSG00000120688.8 | *WBP4* | chr13:41061273-41084006 | yes | no | 4.01E-10 | 3.26E-06 |
| ENSG00000133106.11 | *EPSTI1* | chr13:42886387-42992271 | yes | no | 1.38E-06 | 1.12E-02 |
| ENSG00000083635.7 | *NUFIP1* | chr13:44939248-44989483 | yes | no | 1.22E-06 | 9.95E-03 |
| ENSG00000136152.11 | *COG3* | chr13:45464897-45536630 | yes | no | 2.89E-06 | 2.35E-02 |
| ENSG00000102531.13 | *FNDC3A* | chr13:48975911-49209779 | yes | no | 1.90E-06 | 1.54E-02 |
| ENSG00000005810.14 | *MYCBP2* | chr13:77044654-77327050 | yes | no | 3.68E-08 | 2.99E-04 |
| ENSG00000102580.11 | *DNAJC3* | chr13:95677138-95794989 | yes | no | 8.88E-07 | 7.23E-03 |
| ENSG00000125304.8 | *TM9SF2* | chr13:99501416-99564006 | yes | yes | 1.82E-07 | 1.48E-03 |
| ENSG00000102524.8 | *TNFSF13B* | chr13:108251239-108308484 | yes | yes | 4.19E-12 | 3.41E-08 |
| ENSG00000092010.11 | *PSME1* | chr14:24136157-24138967 | yes | yes | 1.02E-08 | 8.27E-05 |
| ENSG00000100911.10 | *PSME2* | chr14:24143361-24147570 | yes | yes | 2.28E-12 | 1.85E-08 |
| ENSG00000213928.5 | *IRF9* | chr14:24161052-24166565 | yes | yes | 9.56E-09 | 7.78E-05 |
| ENSG00000139899.7 | *CBLN3* | chr14:24426531-24430954 | yes | no | 2.51E-07 | 2.05E-03 |
| ENSG00000100906.7 | *NFKBIA* | chr14:35401510-35404749 | yes | yes | 1.31E-08 | 1.07E-04 |
| ENSG00000186469.5 | *GNG2* | chr14:51826194-51979342 | yes | yes | 1.68E-07 | 1.37E-03 |
| ENSG00000126777.14 | *KTN1* | chr14:55559071-55701526 | yes | no | 1.82E-06 | 1.48E-02 |
| ENSG00000213463.4 | *SYNJ2BP* | chr14:70366495-70417061 | yes | no | 1.83E-07 | 1.49E-03 |
| ENSG00000170348.5 | *TMED10* | chr14:75131469-75176631 | yes | no | 3.01E-07 | 2.45E-03 |
| ENSG00000183576.9 | *SETD3* | chr14:99397745-99480879 | yes | no | 8.84E-07 | 7.19E-03 |
| ENSG00000183484.8 | *GPR132* | chr14:105049388-105065445 | no | yes | 5.08E-10 | 4.13E-06 |
| ENSG00000137880.5 | *GCHFR* | chr15:40764019-40767710 | yes | no | 5.50E-06 | 4.47E-02 |
| ENSG00000140280.10 | *LYSMD2* | chr15:51723010-51751585 | yes | no | 1.88E-13 | 1.53E-09 |
| ENSG00000128872.6 | *TMOD2* | chr15:51751560-51816368 | yes | no | 7.10E-08 | 5.78E-04 |
| ENSG00000069956.8 | *MAPK6* | chr15:51952105-52067372 | yes | no | 1.12E-09 | 9.11E-06 |
| ENSG00000103642.8 | *LACTB* | chr15:63121799-63142061 | yes | yes | 3.25E-09 | 2.64E-05 |
| ENSG00000166794.4 | *PPIB* | chr15:64155811-64163205 | yes | no | 1.06E-06 | 8.64E-03 |
| ENSG00000241839.6 | *PLEKHO2* | chr15:64841882-64868007 | yes | no | 3.94E-06 | 3.20E-02 |
| ENSG00000103769.6 | *RAB11A* | chr15:65726053-65891991 | yes | no | 3.59E-11 | 2.92E-07 |
| ENSG00000129028.5 | *THAP10* | chr15:70881341-70892785 | yes | no | 4.03E-07 | 3.28E-03 |
| ENSG00000140464.16 | *PML* | chr15:73994672-74047812 | yes | yes | 2.16E-06 | 1.76E-02 |
| ENSG00000138623.6 | *SEMA7A* | chr15:74409288-74434467 | yes | yes | 1.67E-07 | 1.36E-03 |
| ENSG00000140497.13 | *SCAMP2* | chr15:74843729-74873365 | yes | no | 1.53E-06 | 1.25E-02 |
| ENSG00000178802.14 | *MPI* | chr15:74890004-74902219 | yes | no | 9.57E-07 | 7.79E-03 |
| ENSG00000167196.10 | *FBXO22* | chr15:75903858-75942510 | yes | no | 3.35E-09 | 2.73E-05 |
| ENSG00000140374.12 | *ETFA* | chr15:76215354-76311472 | yes | no | 1.63E-06 | 1.32E-02 |
| ENSG00000117906.10 | *RCN2* | chr15:76931618-76954392 | yes | no | 2.78E-08 | 2.26E-04 |
| ENSG00000041357.12 | *PSMA4* | chr15:78540404-78552419 | yes | yes | 1.19E-06 | 9.68E-03 |
| ENSG00000140379.7 | *BCL2A1* | chr15:79960888-79971446 | no | yes | 2.90E-07 | 2.36E-03 |
| ENSG00000086666.15 | *ZFAND6* | chr15:80059567-80138393 | yes | no | 5.73E-10 | 4.66E-06 |
| ENSG00000140612.10 | *SEC11A* | chr15:84669537-84716716 | yes | no | 8.46E-07 | 6.89E-03 |
| ENSG00000182768.8 | *NGRN* | chr15:90265658-90278141 | NA | no | 2.69E-08 | 2.19E-04 |
| ENSG00000167962.9 | *ZNF598* | chr16:1997653-2009823 | yes | no | 1.11E-06 | 9.01E-03 |
| ENSG00000185338.4 | *SOCS1* | chr16:11254404-11256179 | no | yes | 2.20E-08 | 1.79E-04 |
| ENSG00000184602.5 | *SNN* | chr16:11668413-11679159 | yes | no | 3.17E-16 | 2.58E-12 |
| ENSG00000153066.9 | *TXNDC11* | chr16:11679079-11742878 | yes | no | 5.54E-09 | 4.51E-05 |
| ENSG00000048462.7 | *TNFRSF17* | chr16:11965106-11968068 | no | yes | 1.39E-12 | 1.13E-08 |
| ENSG00000072864.9 | *NDE1* | chr16:15643266-15726353 | yes | no | 1.50E-07 | 1.22E-03 |
| ENSG00000013364.15 | *MVP* | chr16:29820393-29848039 | yes | no | 5.27E-06 | 4.29E-02 |
| ENSG00000090238.8 | *YPEL3* | chr16:30092313-30096915 | yes | no | 1.22E-07 | 9.93E-04 |
| ENSG00000005844.14 | *ITGAL* | chr16:30472657-30523185 | no | yes | 1.97E-08 | 1.61E-04 |
| ENSG00000125148.6 | *MT2A* | chr16:56608198-56609497 | NA | yes | 1.29E-09 | 1.05E-05 |
| ENSG00000102962.4 | *CCL22* | chr16:57358771-57366190 | no | yes | 2.41E-08 | 1.96E-04 |
| ENSG00000103018.13 | *CYB5B* | chr16:69424524-69466266 | yes | no | 8.54E-09 | 6.96E-05 |
| ENSG00000198373.9 | *WWP2* | chr16:69762305-69941741 | yes | no | 4.12E-06 | 3.35E-02 |
| ENSG00000102984.11 | *ZNF821* | chr16:71859679-71895336 | yes | no | 1.64E-09 | 1.33E-05 |
| ENSG00000090863.8 | *GLG1* | chr16:74451957-74607114 | yes | no | 3.69E-08 | 3.01E-04 |
| ENSG00000003249.10 | *DBNDD1* | chr16:90004864-90020128 | yes | no | 7.27E-09 | 5.92E-05 |
| ENSG00000183688.4 | *FAM101B* | chr17:439977-445939 | NA | no | 3.98E-08 | 3.24E-04 |
| ENSG00000108953.13 | *YWHAE* | chr17:1344271-1400378 | yes | yes | 1.95E-08 | 1.59E-04 |
| ENSG00000197879.11 | *MYO1C* | chr17:1464097-1492812 | yes | no | 2.77E-06 | 2.25E-02 |
| ENSG00000167721.7 | *TSR1* | chr17:2322502-2337507 | yes | no | 1.50E-06 | 1.22E-02 |
| ENSG00000132388.9 | *UBE2G1* | chr17:4269258-4366628 | yes | no | 6.21E-12 | 5.06E-08 |
| ENSG00000108518.7 | *PFN1* | chr17:4945651-4949061 | yes | yes | 6.38E-08 | 5.19E-04 |
| ENSG00000108515.14 | *ENO3* | chr17:4948091-4957131 | no | no | 2.06E-08 | 1.68E-04 |
| ENSG00000091592.12 | *NLRP1* | chr17:5499426-5619424 | yes | yes | 2.80E-07 | 2.28E-03 |
| ENSG00000132530.13 | *XAF1* | chr17:6755446-6775647 | yes | yes | 3.69E-08 | 3.00E-04 |
| ENSG00000129226.10 | *CD68* | chr17:7579466-7582113 | yes | yes | 7.01E-09 | 5.70E-05 |
| ENSG00000109103.8 | *UNC119* | chr17:28546706-28552668 | yes | no | 1.59E-14 | 1.29E-10 |
| ENSG00000109107.10 | *ALDOC* | chr17:28573114-28577264 | yes | no | 8.61E-07 | 7.01E-03 |
| ENSG00000076604.11 | *TRAF4* | chr17:28743983-28750958 | yes | yes | 9.62E-13 | 7.83E-09 |
| ENSG00000132589.12 | *FLOT2* | chr17:28879334-28897679 | yes | no | 1.56E-08 | 1.27E-04 |
| ENSG00000167543.12 | *TP53I13* | chr17:29566051-29573157 | yes | no | 6.63E-07 | 5.40E-03 |
| ENSG00000185862.6 | *EVI2B* | chr17:31303765-31314112 | yes | no | 3.33E-07 | 2.71E-03 |
| ENSG00000271503.2 | *CCL5* | chr17:35871490-35880793 | yes | yes | 1.36E-06 | 1.11E-02 |
| ENSG00000275832.1 | *ARHGAP23* | chr17:38428417-38512392 | NA | no | 3.51E-06 | 2.85E-02 |
| ENSG00000131759.14 | *RARA* | chr17:40309191-40357643 | yes | yes | 1.07E-09 | 8.70E-06 |
| ENSG00000126561.13 | *STAT5A* | chr17:42287546-42311943 | yes | yes | 3.70E-11 | 3.01E-07 |
| ENSG00000168610.11 | *STAT3* | chr17:42313323-42388568 | yes | yes | 5.08E-06 | 4.13E-02 |
| ENSG00000175832.9 | *ETV4* | chr17:43527843-43579620 | no | no | 5.22E-09 | 4.25E-05 |
| ENSG00000002919.11 | *SNX11* | chr17:48103356-48123074 | yes | no | 4.47E-14 | 3.64E-10 |
| ENSG00000159202.14 | *UBE2Z* | chr17:48908368-48929056 | yes | yes | 6.73E-11 | 5.48E-07 |
| ENSG00000108798.5 | *ABI3* | chr17:49210226-49223225 | yes | no | 4.33E-11 | 3.53E-07 |
| ENSG00000167085.8 | *PHB* | chr17:49404048-49414905 | yes | yes | 4.89E-06 | 3.98E-02 |
| ENSG00000121104.4 | *FAM117A* | chr17:49710331-49789180 | yes | no | 1.10E-06 | 8.95E-03 |
| ENSG00000108819.10 | *PPP1R9B* | chr17:50133734-50150630 | yes | yes | 2.35E-06 | 1.91E-02 |
| ENSG00000108829.9 | *LRRC59* | chr17:50375058-50397553 | yes | no | 2.13E-09 | 1.74E-05 |
| ENSG00000108960.4 | *MMD* | chr17:55392612-55421992 | yes | no | 1.13E-11 | 9.18E-08 |
| ENSG00000108389.6 | *MTMR4* | chr17:58489528-58517905 | yes | no | 3.69E-07 | 3.01E-03 |
| ENSG00000008283.12 | *CYB561* | chr17:63432303-63446378 | yes | no | 3.45E-09 | 2.81E-05 |
| ENSG00000178607.12 | *ERN1* | chr17:64039141-64130819 | yes | yes | 3.88E-09 | 3.16E-05 |
| ENSG00000070540.9 | *WIPI1* | chr17:68420947-68457513 | yes | no | 8.91E-09 | 7.26E-05 |
| ENSG00000125398.5 | *SOX9* | chr17:72121019-72126420 | yes | yes | 6.92E-09 | 5.63E-05 |
| ENSG00000180616.5 | *SSTR2* | chr17:73165011-73171046 | yes | no | 2.32E-11 | 1.89E-07 |
| ENSG00000167851.10 | *CD300A* | chr17:74466415-74484796 | yes | no | 1.41E-06 | 1.15E-02 |
| ENSG00000129657.11 | *SEC14L1* | chr17:77086715-77217101 | yes | no | 1.01E-10 | 8.23E-07 |
| ENSG00000141582.11 | *CBX4* | chr17:79833155-79839429 | yes | no | 1.26E-08 | 1.02E-04 |
| ENSG00000225663.4 | *FAM195B* | chr17:81822360-81833302 | NA | no | 4.57E-07 | 3.72E-03 |
| ENSG00000185624.11 | *P4HB* | chr17:81843158-81860694 | NA | yes | 7.76E-09 | 6.32E-05 |
| ENSG00000167088.7 | *SNRPD1* | chr18:21612266-21630456 | yes | no | 8.68E-07 | 7.06E-03 |
| ENSG00000118276.8 | *B4GALT6* | chr18:31622246-31685836 | yes | no | 8.42E-07 | 6.86E-03 |
| ENSG00000166974.9 | *MAPRE2* | chr18:34976927-35143470 | yes | no | 8.51E-08 | 6.93E-04 |
| ENSG00000152229.15 | *PSTPIP2* | chr18:45983535-46072272 | no | yes | 1.14E-06 | 9.32E-03 |
| ENSG00000172175.9 | *MALT1* | chr18:58671385-58750139 | yes | yes | 7.31E-07 | 5.95E-03 |
| ENSG00000141682.11 | *PMAIP1* | chr18:59899947-59904306 | no | yes | 8.38E-08 | 6.82E-04 |
| ENSG00000166347.15 | *CYB5A* | chr18:74250846-74292016 | yes | no | 5.17E-14 | 4.21E-10 |
| ENSG00000197971.11 | *MBP* | chr18:76978826-77133683 | yes | yes | 1.73E-06 | 1.41E-02 |
| ENSG00000167468.13 | *GPX4* | chr19:1103925-1106791 | yes | yes | 3.31E-06 | 2.70E-02 |
| ENSG00000130005.8 | *GAMT* | chr19:1397083-1401570 | yes | no | 5.45E-09 | 4.44E-05 |
| ENSG00000071564.11 | *TCF3* | chr19:1609289-1652605 | yes | no | 1.80E-08 | 1.47E-04 |
| ENSG00000099875.11 | *MKNK2* | chr19:2037464-2051244 | yes | no | 3.84E-13 | 3.13E-09 |
| ENSG00000176533.9 | *GNG7* | chr19:2511218-2702709 | yes | yes | 1.30E-10 | 1.06E-06 |
| ENSG00000125910.5 | *S1PR4* | chr19:3172345-3180332 | yes | yes | 9.96E-09 | 8.11E-05 |
| ENSG00000105246.5 | *EBI3* | chr19:4229497-4237531 | yes | yes | 1.88E-12 | 1.53E-08 |
| ENSG00000125657.4 | *TNFSF9* | chr19:6530998-6535928 | yes | yes | 5.44E-10 | 4.43E-06 |
| ENSG00000125733.14 | *TRIP10* | chr19:6737924-6751526 | yes | yes | 3.66E-09 | 2.98E-05 |
| ENSG00000090339.5 | *ICAM1* | chr19:10270834-10286615 | yes | yes | 3.13E-10 | 2.55E-06 |
| ENSG00000142453.8 | *CARM1* | chr19:10871512-10923070 | yes | yes | 4.36E-07 | 3.55E-03 |
| ENSG00000130175.6 | *PRKCSH* | chr19:11435287-11450968 | yes | yes | 2.25E-07 | 1.83E-03 |
| ENSG00000171223.5 | *JUNB* | chr19:12791495-12793315 | yes | yes | 1.55E-12 | 1.26E-08 |
| ENSG00000099797.8 | *TECR* | chr19:14517084-14565980 | yes | no | 1.29E-06 | 1.05E-02 |
| ENSG00000187912.8 | *CLEC17A* | chr19:14583083-14611157 | yes | no | 3.25E-07 | 2.64E-03 |
| ENSG00000127528.5 | *KLF2* | chr19:16324816-16327874 | yes | no | 5.71E-09 | 4.65E-05 |
| ENSG00000216490.3 | *IFI30* | chr19:18173161-18178117 | yes | yes | 5.40E-12 | 4.39E-08 |
| ENSG00000105656.9 | *ELL* | chr19:18442662-18522127 | yes | no | 3.75E-06 | 3.05E-02 |
| ENSG00000181035.10 | *SLC25A42* | chr19:19063998-19112888 | yes | no | 3.08E-08 | 2.51E-04 |
| ENSG00000166289.5 | *PLEKHF1* | chr19:29665055-29675457 | yes | no | 2.64E-07 | 2.15E-03 |
| ENSG00000167645.13 | *YIF1B* | chr19:38305103-38317273 | yes | no | 3.39E-06 | 2.76E-02 |
| ENSG00000130755.9 | *GMFG* | chr19:39328352-39342372 | yes | no | 2.32E-07 | 1.89E-03 |
| ENSG00000128016.5 | *ZFP36* | chr19:39406812-39409412 | yes | yes | 5.11E-06 | 4.16E-02 |
| ENSG00000105223.15 | *PLD3* | chr19:40348455-40380439 | yes | no | 8.70E-08 | 7.09E-04 |
| ENSG00000105404.7 | *RABAC1* | chr19:41956680-41959390 | yes | no | 1.73E-10 | 1.40E-06 |
| ENSG00000069399.9 | *BCL3* | chr19:44747704-44760044 | yes | yes | 1.23E-07 | 1.00E-03 |
| ENSG00000104856.10 | *RELB* | chr19:45001429-45038194 | yes | yes | 1.48E-08 | 1.20E-04 |
| ENSG00000125753.10 | *VASP* | chr19:45506578-45526983 | yes | yes | 6.61E-07 | 5.38E-03 |
| ENSG00000126457.17 | *PRMT1* | chr19:49675785-49689029 | yes | yes | 2.76E-08 | 2.25E-04 |
| ENSG00000105379.6 | *ETFB* | chr19:51345168-51366418 | yes | no | 3.53E-06 | 2.88E-02 |
| ENSG00000125826.16 | *RBCK1* | chr20:407497-430966 | yes | yes | 6.31E-08 | 5.14E-04 |
| ENSG00000101224.14 | *CDC25B* | chr20:3786771-3806121 | yes | yes | 2.96E-07 | 2.41E-03 |
| ENSG00000089050.11 | *RBBP9* | chr20:18486539-18497243 | yes | no | 3.74E-10 | 3.05E-06 |
| ENSG00000101294.13 | *HM13* | chr20:31514427-31577923 | yes | yes | 2.82E-08 | 2.29E-04 |
| ENSG00000171552.9 | *BCL2L1* | chr20:31664451-31723989 | yes | yes | 6.61E-07 | 5.38E-03 |
| ENSG00000126003.6 | *PLAGL2* | chr20:32192502-32207791 | yes | no | 2.81E-06 | 2.28E-02 |
| ENSG00000131069.16 | *ACSS2* | chr20:34872145-34927962 | yes | no | 5.92E-07 | 4.82E-03 |
| ENSG00000088298.9 | *EDEM2* | chr20:35115356-35147364 | yes | no | 4.98E-07 | 4.05E-03 |
| ENSG00000118705.13 | *RPN2* | chr20:37178409-37241623 | yes | no | 7.70E-08 | 6.27E-04 |
| ENSG00000132824.10 | *SERINC3* | chr20:44496220-44522109 | yes | no | 3.53E-07 | 2.88E-03 |
| ENSG00000244274.4 | *DBNDD2* | chr20:45406056-45410610 | yes | no | 6.69E-07 | 5.44E-03 |
| ENSG00000101017.10 | *CD40* | chr20:46118271-46129863 | no | yes | 1.80E-15 | 1.47E-11 |
| ENSG00000158470.5 | *B4GALT5* | chr20:49632944-49713878 | yes | no | 1.03E-07 | 8.34E-04 |
| ENSG00000171940.10 | *ZNF217* | chr20:53567064-53609907 | yes | no | 5.67E-07 | 4.62E-03 |
| ENSG00000124256.11 | *ZBP1* | chr20:57603845-57620576 | no | yes | 1.09E-08 | 8.84E-05 |
| ENSG00000203896.6 | *LIME1* | chr20:63736282-63739103 | yes | yes | 5.91E-06 | 4.81E-02 |
| ENSG00000234883.3 | *MIR155HG* | chr21:25561908-25575168 | NA | no | 1.50E-18 | 1.22E-14 |
| ENSG00000154719.10 | *MRPL39* | chr21:25585655-25607517 | yes | no | 1.94E-06 | 1.58E-02 |
| ENSG00000142192.17 | *APP* | chr21:25880549-26171128 | yes | yes | 5.73E-07 | 4.66E-03 |
| ENSG00000159110.16 | *IFNAR2* | chr21:33229900-33265675 | yes | yes | 5.26E-13 | 4.28E-09 |
| ENSG00000243646.5 | *IL10RB* | chr21:33266357-33297234 | yes | yes | 5.23E-07 | 4.26E-03 |
| ENSG00000142166.9 | *IFNAR1* | chr21:33324476-33359862 | yes | yes | 1.28E-12 | 1.04E-08 |
| ENSG00000185658.10 | *BRWD1* | chr21:39184175-39321559 | yes | no | 9.62E-08 | 7.83E-04 |
| ENSG00000183486.9 | *MX2* | chr21:41361942-41409390 | yes | no | 5.05E-10 | 4.11E-06 |
| ENSG00000157601.10 | *MX1* | chr21:41420303-41459214 | yes | yes | 2.43E-06 | 1.98E-02 |
| ENSG00000160216.15 | *AGPAT3* | chr21:43865185-43986536 | yes | no | 1.10E-06 | 8.97E-03 |
| ENSG00000184979.9 | *USP18* | chr22:18149898-18177397 | yes | yes | 2.76E-12 | 2.24E-08 |
| ENSG00000100219.13 | *XBP1* | chr22:28794554-28800597 | yes | yes | 1.14E-10 | 9.31E-07 |
| ENSG00000185339.5 | *TCN2* | chr22:30606837-30627278 | yes | no | 5.06E-08 | 4.12E-04 |
| ENSG00000213923.7 | *CSNK1E* | chr22:38290690-38398522 | yes | no | 3.52E-06 | 2.86E-02 |
| ENSG00000239713.4 | *APOBEC3G* | chr22:39040960-39087743 | yes | yes | 9.68E-08 | 7.88E-04 |
| ENSG00000100307.9 | *CBX7* | chr22:39120166-39152674 | yes | no | 1.79E-07 | 1.45E-03 |
| ENSG00000167077.9 | *MEI1* | chr22:41699498-41799456 | no | no | 2.78E-07 | 2.26E-03 |
| ENSG00000100266.14 | *PACSIN2* | chr22:42835411-43015145 | yes | no | 1.14E-08 | 9.32E-05 |
| ENSG00000100422.10 | *CERK* | chr22:46684410-46738261 | yes | yes | 3.52E-11 | 2.87E-07 |
| ENSG00000198355.4 | *PIM3* | chr22:49960512-49964080 | NA | yes | 2.22E-06 | 1.81E-02 |
| ENSG00000185386.11 | *MAPK11* | chr22:50263712-50270767 | yes | yes | 5.08E-10 | 4.14E-06 |
| ENSG00000025708.9 | *TYMP* | chr22:50525751-50530085 | yes | yes | 1.90E-08 | 1.55E-04 |
| ENSG00000177989.10 | *ODF3B* | chr22:50529709-50532580 | NA | no | 7.70E-07 | 6.26E-03 |
| ENSG00000123595.6 | *RAB9A* | chrX:13689120-13710506 | yes | no | 2.62E-10 | 2.13E-06 |
| ENSG00000046651.11 | *OFD1* | chrX:13734744-13769353 | yes | no | 1.53E-07 | 1.25E-03 |
| ENSG00000102172.12 | *SMS* | chrX:21940572-21994835 | NA | no | 3.90E-09 | 3.18E-05 |
| ENSG00000123130.13 | *ACOT9* | chrX:23702252-23766475 | yes | no | 7.99E-07 | 6.51E-03 |
| ENSG00000198814.9 | *GK* | chrX:30653358-30731456 | yes | no | 2.08E-06 | 1.69E-02 |
| ENSG00000165168.7 | *CYBB* | chrX:37780010-37813461 | yes | yes | 8.31E-07 | 6.76E-03 |
| ENSG00000180182.7 | *MED14* | chrX:40648305-40735858 | yes | no | 1.58E-07 | 1.29E-03 |
| ENSG00000102265.8 | *TIMP1* | chrX:47582312-47586789 | yes | yes | 3.54E-06 | 2.88E-02 |
| ENSG00000126767.14 | *ELK1* | chrX:47635520-47650604 | yes | yes | 6.04E-08 | 4.92E-04 |
| ENSG00000102100.11 | *SLC35A2* | chrX:48903181-48911958 | yes | no | 2.67E-08 | 2.17E-04 |
| ENSG00000196998.12 | *WDR45* | chrX:49074432-49101170 | yes | no | 1.39E-06 | 1.13E-02 |
| ENSG00000158526.7 | *TSR2* | chrX:54440400-54445487 | yes | no | 2.85E-06 | 2.32E-02 |
| ENSG00000010671.12 | *BTK* | chrX:101349446-101390796 | no | yes | 1.03E-06 | 8.39E-03 |
| ENSG00000126945.8 | *HNRNPH2* | chrX:101408294-101414133 | yes | no | 4.58E-07 | 3.73E-03 |
| ENSG00000125351.7 | *UPF3B* | chrX:119834021-119852998 | yes | no | 2.38E-06 | 1.94E-02 |
| ENSG00000171155.7 | *C1GALT1C1* | chrX:120625792-120630150 | yes | no | 2.70E-06 | 2.20E-02 |
| ENSG00000165704.11 | *HPRT1* | chrX:134460152-134520513 | yes | no | 1.94E-06 | 1.58E-02 |
| ENSG00000063587.13 | *ZNF275* | chrX:153334154-153360110 | NA | no | 8.67E-09 | 7.06E-05 |
| ENSG00000180879.10 | *SSR4* | chrX:153793515-153798505 | yes | no | 4.84E-08 | 3.94E-04 |
| ENSG00000196924.11 | *FLNA* | chrX:154348523-154374638 | yes | no | 4.83E-07 | 3.93E-03 |
| ENSG00000155959.7 | *VBP1* | chrX:155197006-155239817 | yes | no | 5.41E-06 | 4.41E-02 |
| The analyses tabulated here are for the 647 transcripts with differential expression by schizophrenia status from the meta-analysis of 1 189 RNAseq-studied subjects (529 cases and 660 controls) and 714 array-studied subjects (268 cases and 446 controls) using the same measured covariates, and restricted to the 8 141 genes detected in 80% or more subjects for each technology (Bonferroni *P*-values are adjusted for 8 141 genes analyzed). Immune-related, i.e., protein-coding gene function containing “immune” from genecards.org, is noted (yes, no, or NA for those unlisted). Genes are also tabulated for brain expression (yes, no, or NA for those unlisted) in the adult (hbatlas.org, ^1^). | | | | | | |

**References.**

1. Kang HJ, Kawasawa YI, Cheng F, Zhu Y, Xu X, Li M*, et al*. Spatio-temporal transcriptome of the human brain. *Nature* 2011; **478**(7370)**:** 483-489.
